# Supplementary material for: Design, Synthesis, In Silico and In Vitro Studies of New Immunomodulatory Anticancer Nicotinamide Derivatives Targeting VEGFR-2
Source: Molecules. 2022 Jun 24;27(13):4079. doi: 10.3390/molecules27134079 (PMC9268560; doi:10.3390/molecules27134079)
Supplement: Supplementary file 1 [file molecules-27-04079-s001.zip › molecules-1734805-supplementary.pdf]

Design, Synthesis, In Silico and In Vitro Studies of New Immunomodulatory Anticancer Nicotinamide Derivatives Targeting VEGFR-2

Reda G.Yousef [1], Wagdy M. Eldehna [2], Alaa Elwan [1], Abdelaziz S. Abdelaziz [1], Ahmed B. M. Mehany [3], Ibraheem M. M. Gobaara [3], Bshra A. Alsfouk [4], Eslam B. Elkaeed [5], Ahmed M. Metwaly [6,7,]* Ibrahim. H. Eissa [1,]*

[1] Pharmaceutical Medicinal Chemistry & Drug Design Department, Faculty of Pharmacy (Boys), Al-Azhar University, Cairo 11884, Egypt

[2] Department of Pharmaceutical Chemistry, Faculty of Pharmacy, Kafrelsheikh University, P.O. Box 33516, Kafrelsheikh, Egypt

[3] Zoology Department, Faculty of Science (Boys), Al-Azhar University, Cairo 11884, Egypt

[4] Department of Pharmaceutical Sciences, College of Pharmacy, Princess Nourah bint Abdulrahman University, P.O. Box 84428, Riyadh 11671, Saudi Arabia

[5] Department of Pharmaceutical Sciences, College of Pharmacy, AlMaarefa University, Riyadh 13713, Saudi Arabia

[6] Pharmacognosy and Medicinal Plants Department, Faculty of Pharmacy (Boys), Al-Azhar University, Cairo 11884, Egypt

[7] Biopharmaceutical Products Research Department, Genetic Engineering and Biotechnology Research Institute, City of Scientific Research and Technological Applications (SRTA-City), Alexandria, Egypt

*** Corresponding authors:**

Ibrahim H. Eissa

Medicinal Chemistry Department, Faculty of Pharmacy (Boys),
Al-Azhar University, Cairo 11884, Egypt. **Email:** Ibrahimeissa@azhar.edu.eg

Ahmed M. Metwaly

Pharmacognosy and Medicinal Plants Department, Faculty of Pharmacy (Boys), Al-Azhar University, Cairo 11884, Egypt. **Email:** ametwaly@azhar.edu.eg

Content

1	**4.2. Chemistry and materials**
2	**Spectral data of final target compounds 6,7,8,11, and 12**
3	**4.3. Experimental of Biological testing**<br>**4.3.1**. In vitro anti-proliferative activity<br>**4.3.2.** In vitro VEGFR-2 kinase assay<br>**4.3.3.** Flow cytometry analysis for cell cycle<br>**4.3.4.** Flow cytometry analysis for apoptosis<br>**4.3.5.** Quantitative Real Time Reverse-Transcriptase PCR technique
4	**4.4. Experimental of *in silico* studies**<br>**4.4.1.** Docking studies<br>**4.4.2.** ADMET studies<br>**4.4.3.** Toxicity studies
5	***In silico* toxicity data of final target compounds 6,7,8,11, and 12**

1- 4.2. Chemistry and material

All melting points were carried out by open capillary method on a Gallen kamp Melting point apparatus. The infrared spectra were recorded on pye Unicam SP 1000 IR spectrophotometer using potassium bromide disc technique. Proton magnetic resonance 1HNMR spectra were recorded on a Bruker 400 Megahertz-nuclear magnetic resonance (400 MHZ-NMR) spectrophotometer. Carbon-13 (C13) nuclear magnetic resonance (13CNMR) spectra were recorded on a Bruker 100 Megahertz-nuclear magnetic resonance (100 MHZ-NMR) spectrophotometer. Tetramethylsilane (TMS) was used as internal standard and chemical shifts were measured in δ scale one part per million (ppm). All compounds were within ±0.4 of the theoretical values. The reactions were monitored by thin-layer chromatography (TLC) using TLC sheets precoated with UV fluorescent silica gel Merck 60 F254 plates and were visualized using ultraviolet (UV) lamp and different solvents as mobile phases.

2- Spectral data of final target compounds 6,7,8,11, and 12

IR of compound 6

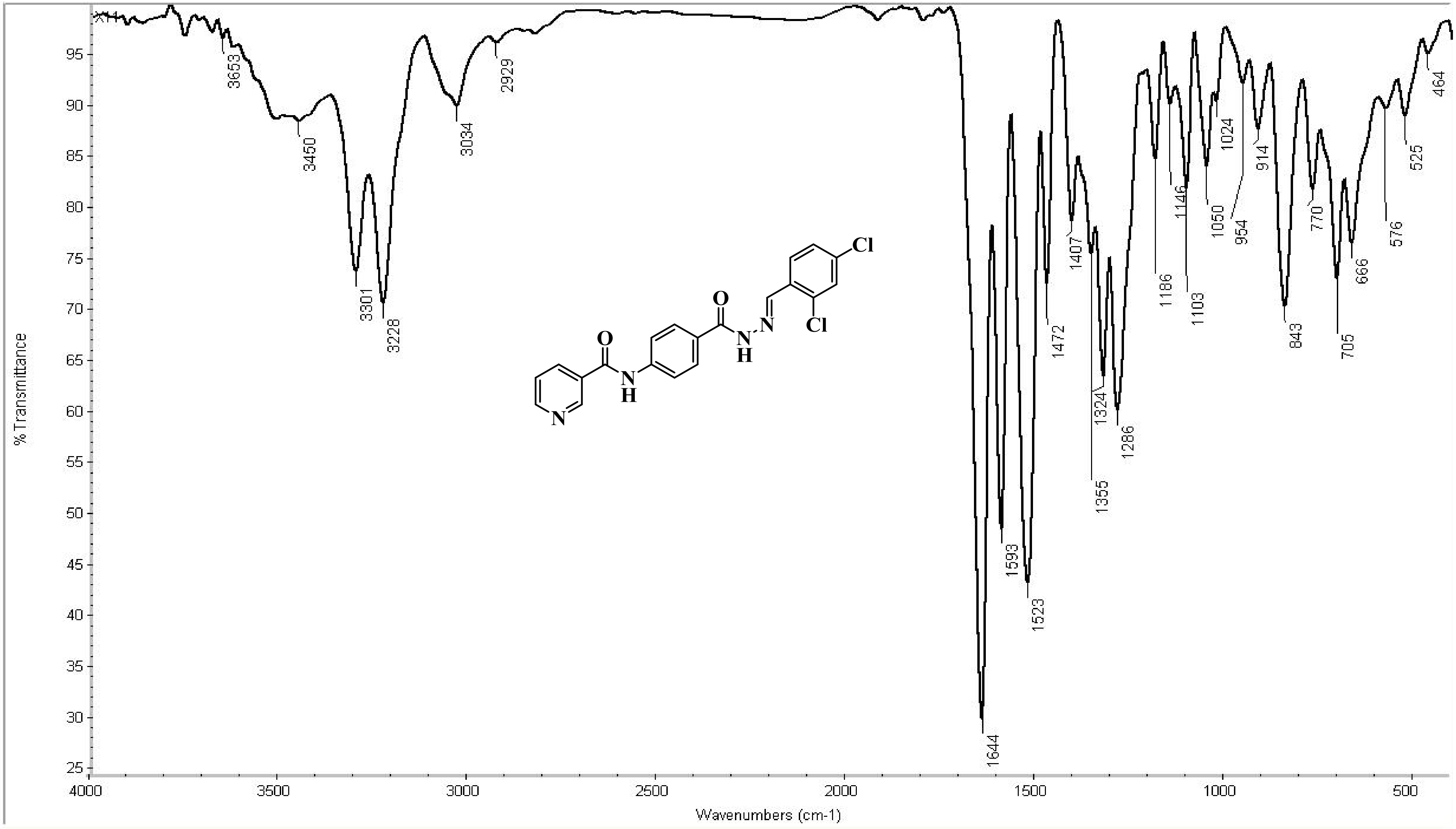

1H NMR of compound 6

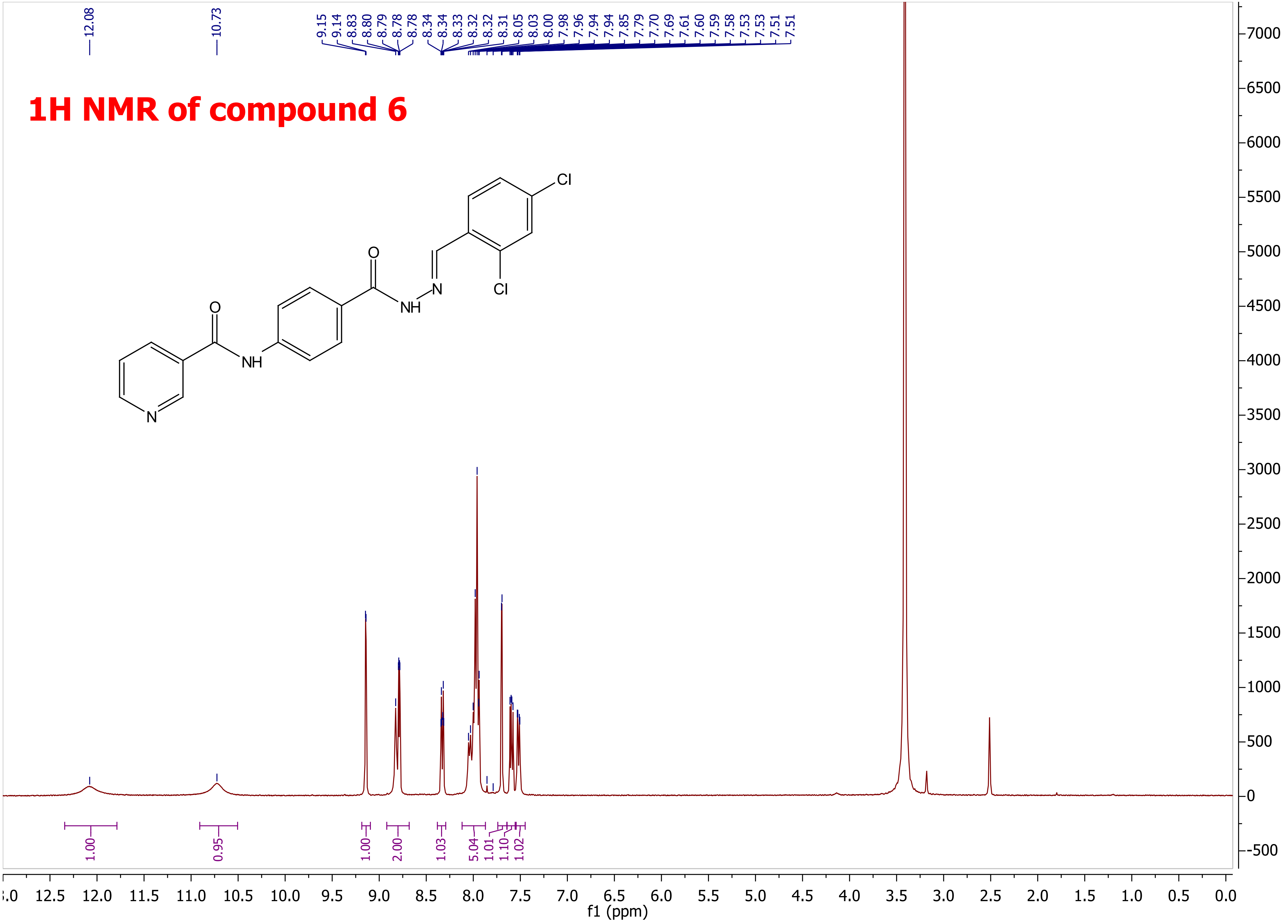

1H NMR of compound 6

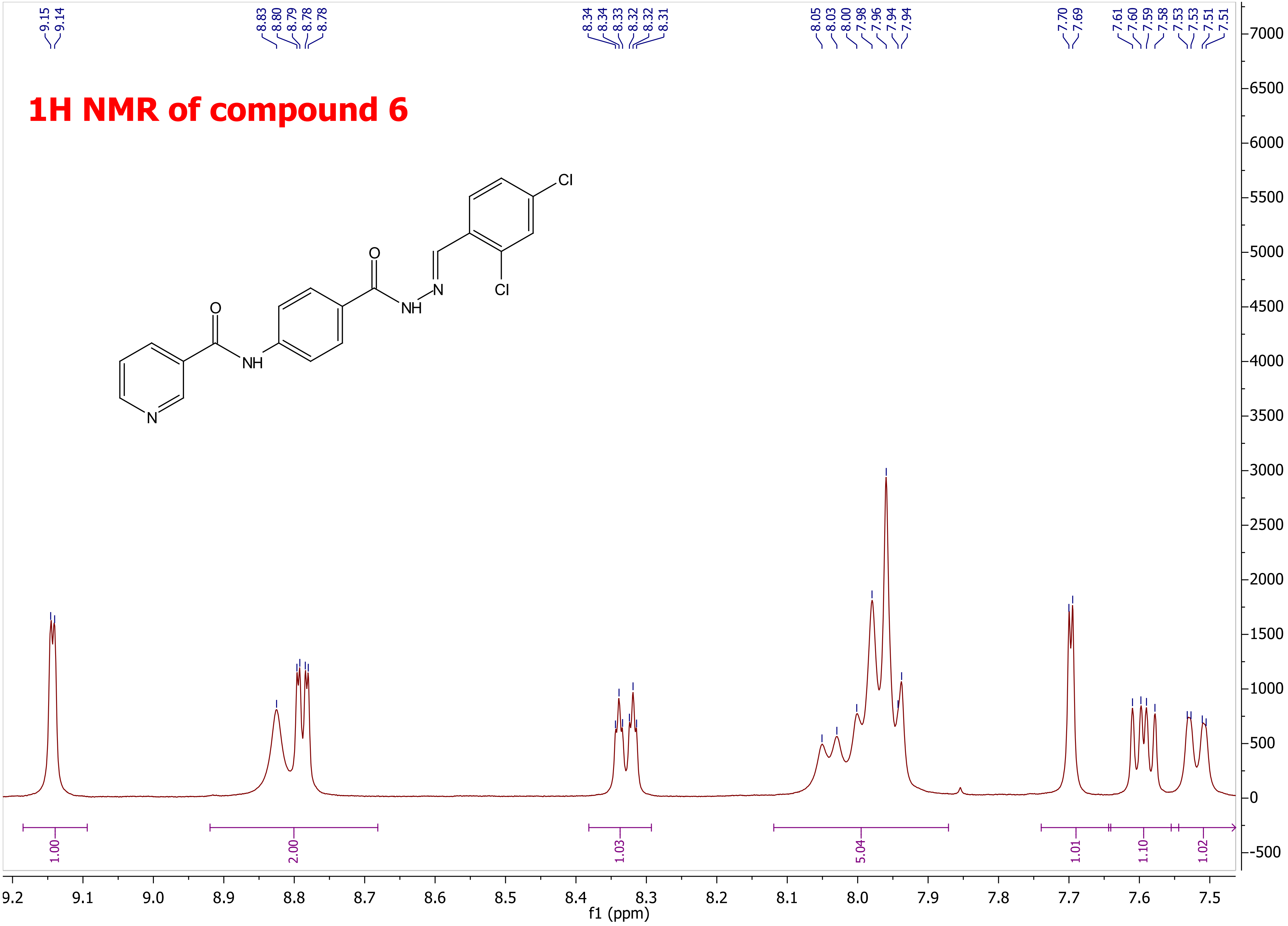

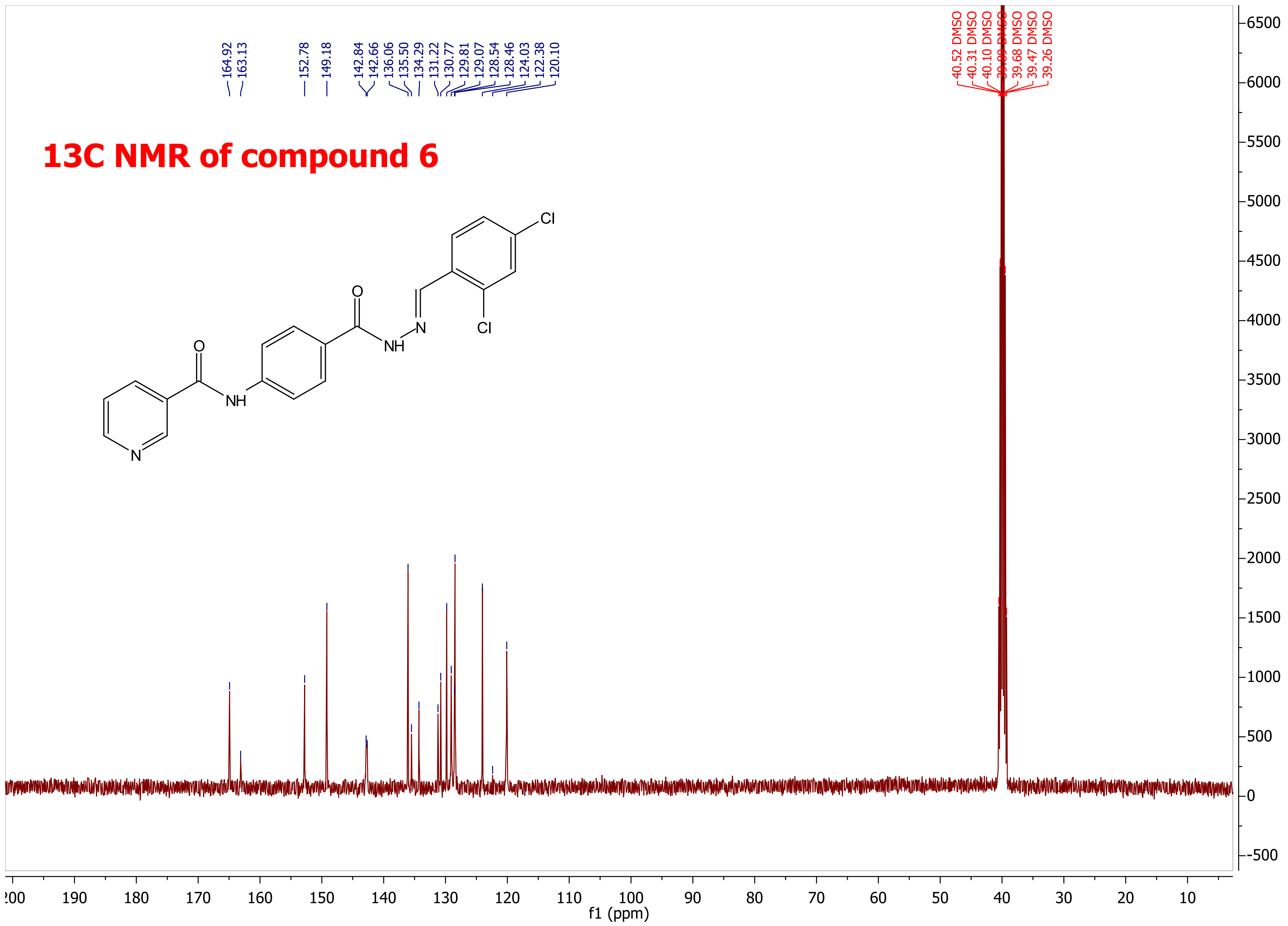

13C NMR of compound 6
164.92
163.13
152.78
149.18
142.84
142.66
136.06
135.50
134.29
131.22
130.77
129.81
129.07
128.54
128.46
124.03
122.38
120.10
40.52 DMSO
40.31 DMSO
40.10 DMSO
39.89 DMSO
39.68 DMSO
39.47 DMSO
39.26 DMSO
O
NH
N
O
NH
N
Cl
Cl
f1 (ppm)

IR of compound 7

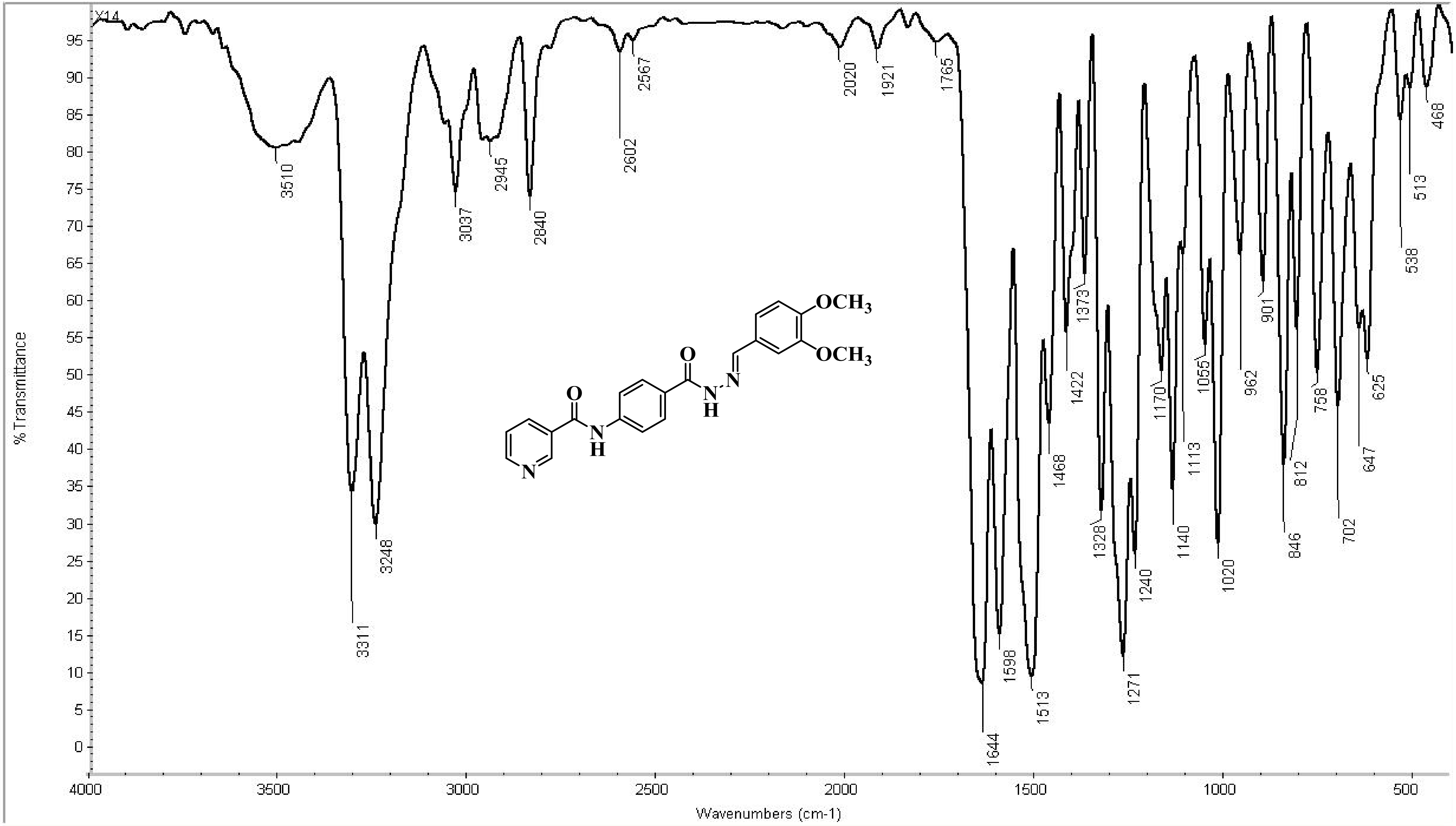

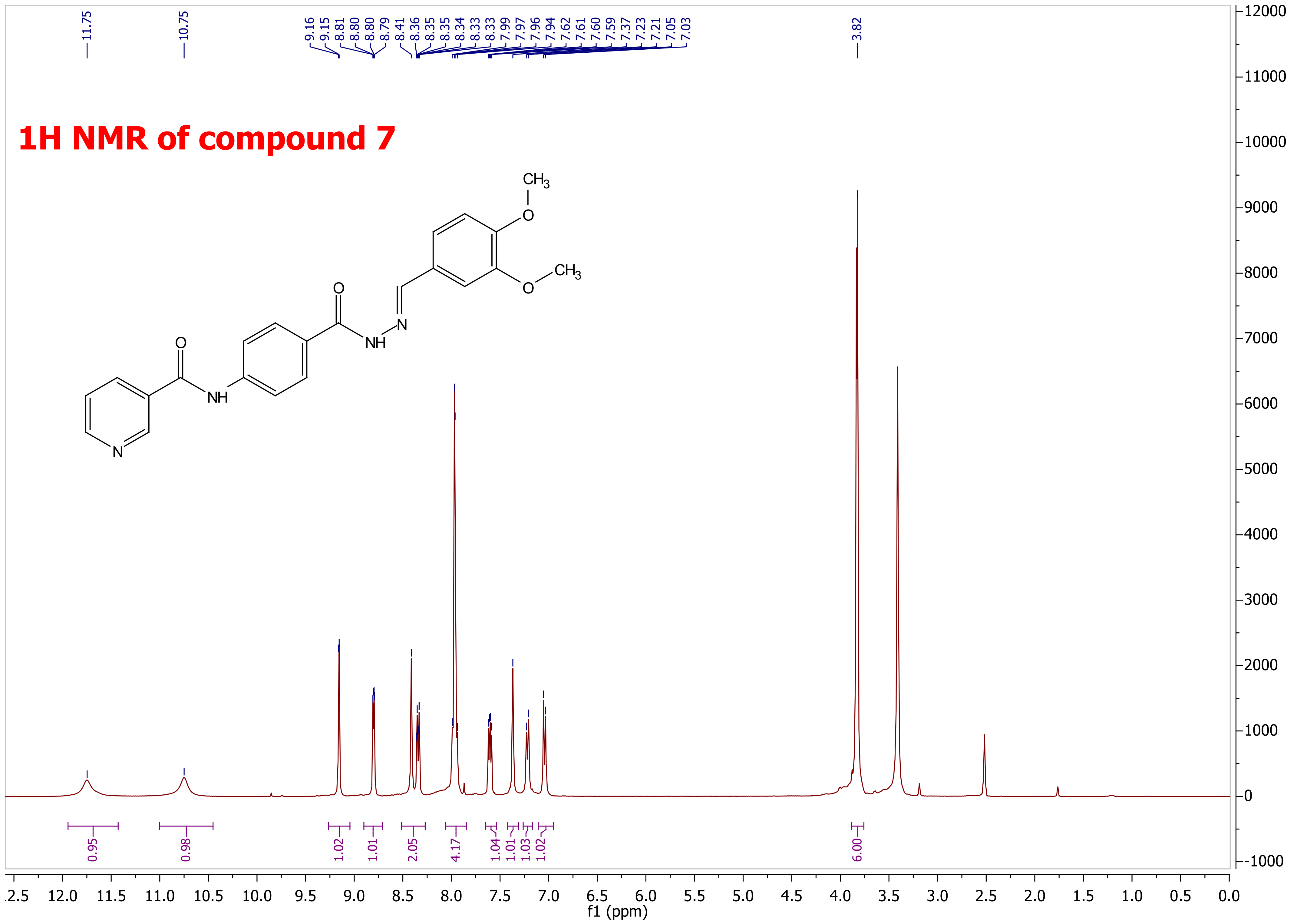
1H NMR of compound 7
11.75
10.75
9.16
9.15
8.81
8.80
8.80
8.79
8.41
8.36
8.35
8.35
8.34
8.33
8.33
7.99
7.97
7.96
7.94
7.62
7.61
7.60
7.59
7.37
7.23
7.21
7.05
7.03
3.82
CH3
O
O
CH3
O
N
NH
O
NH
N
0.95
0.98
1.02
1.01
2.05
4.17
1.04
1.01
1.03
1.02
6.00
f1 (ppm)

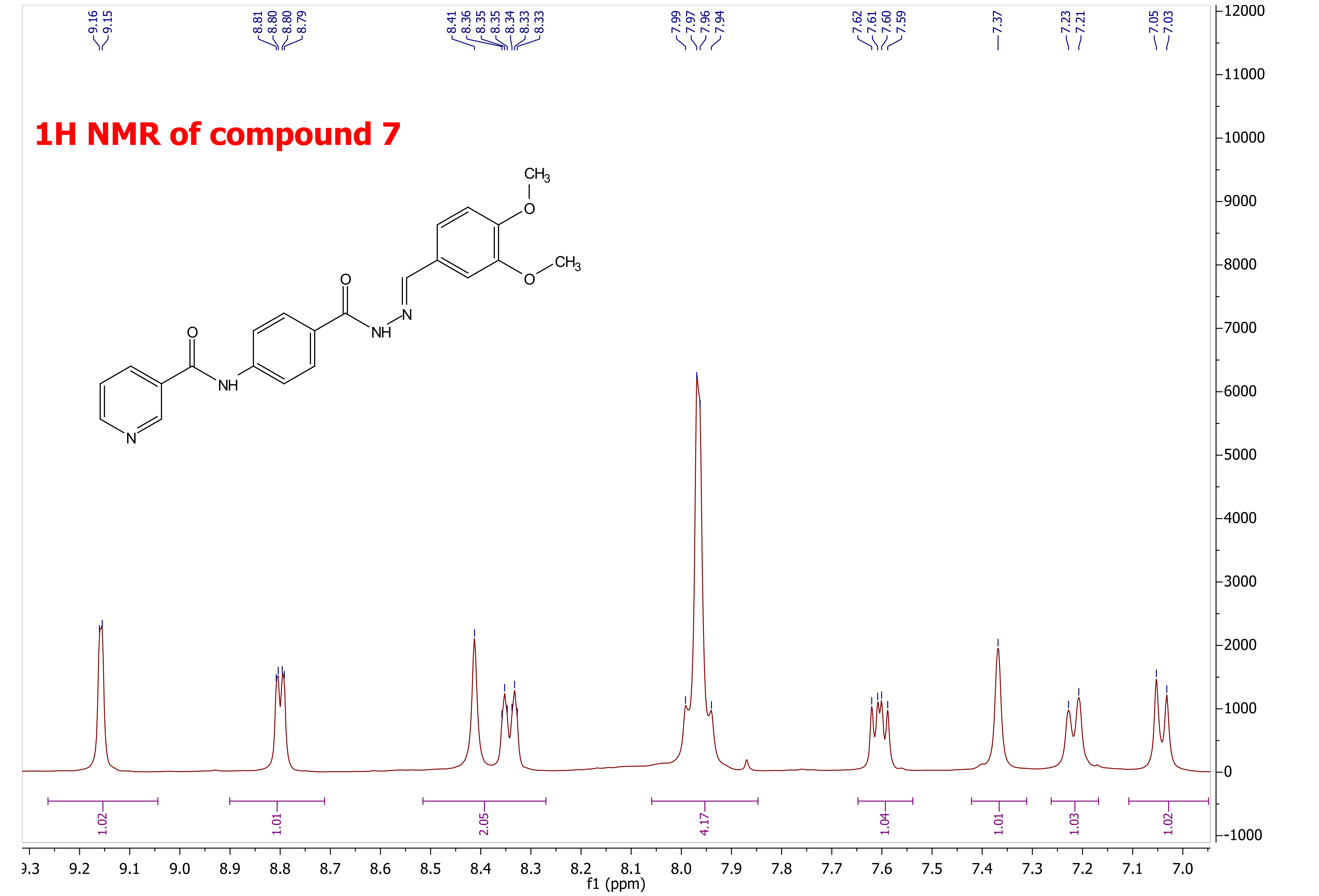
1H NMR of compound 7
9.16
9.15
8.81
8.80
8.80
8.79
8.41
8.36
8.35
8.35
8.34
8.33
8.33
7.99
7.97
7.96
7.94
7.62
7.61
7.60
7.59
7.37
7.23
7.21
7.05
7.03
1.02
1.01
2.05
4.17
1.04
1.01
1.03
1.02
CH3
O
O
CH3
O
N
NH
O
NH
N
f1 (ppm)

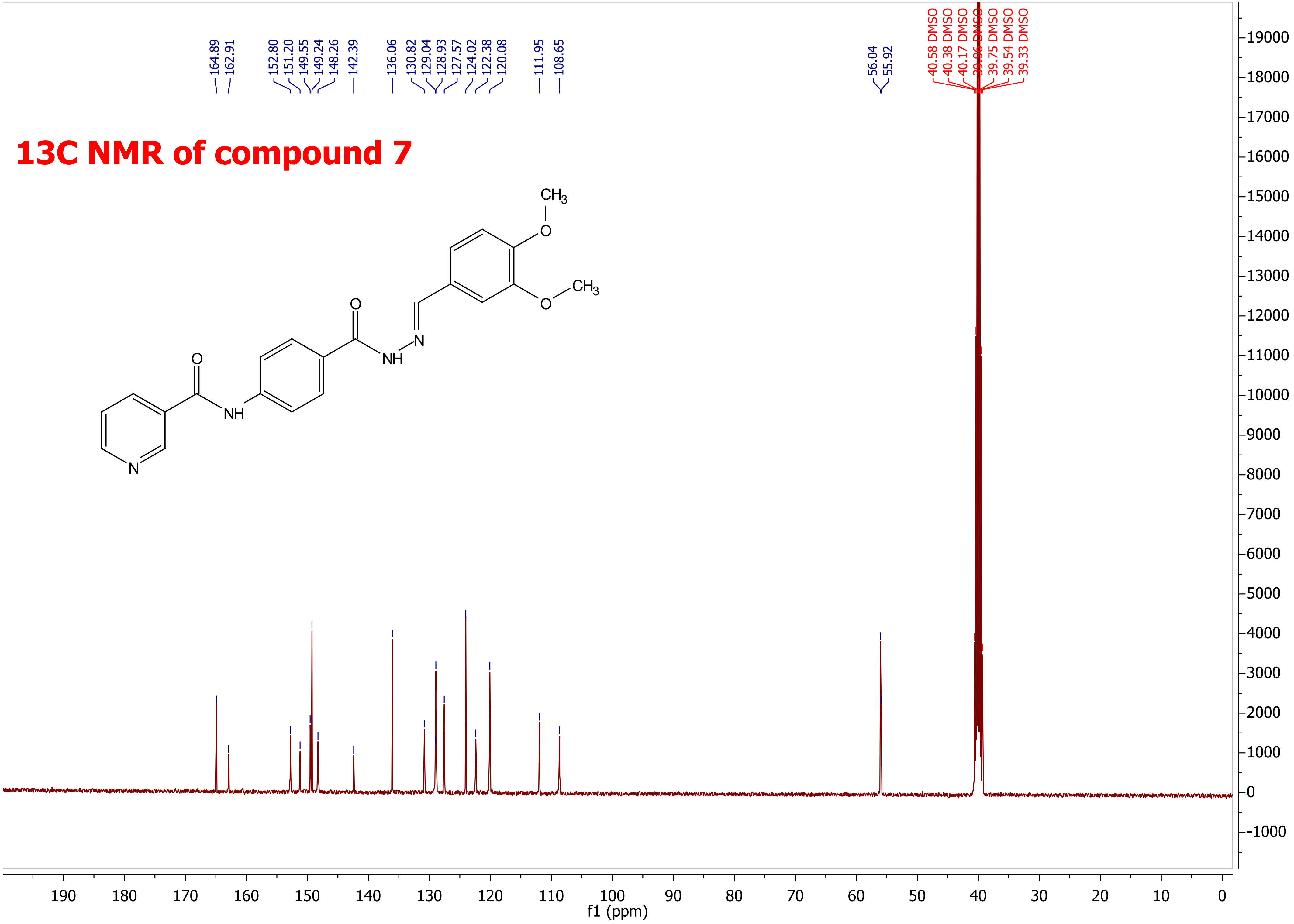

13C NMR of compound 7
164.89
162.91
152.80
151.20
149.55
149.24
148.26
142.39
136.06
130.82
129.04
128.93
127.57
124.02
122.38
120.08
111.95
108.65
56.04
55.92
40.58 DMSO
40.38 DMSO
40.17 DMSO
39.96 DMSO
39.75 DMSO
39.54 DMSO
39.33 DMSO
f1 (ppm)

IR of compound 8

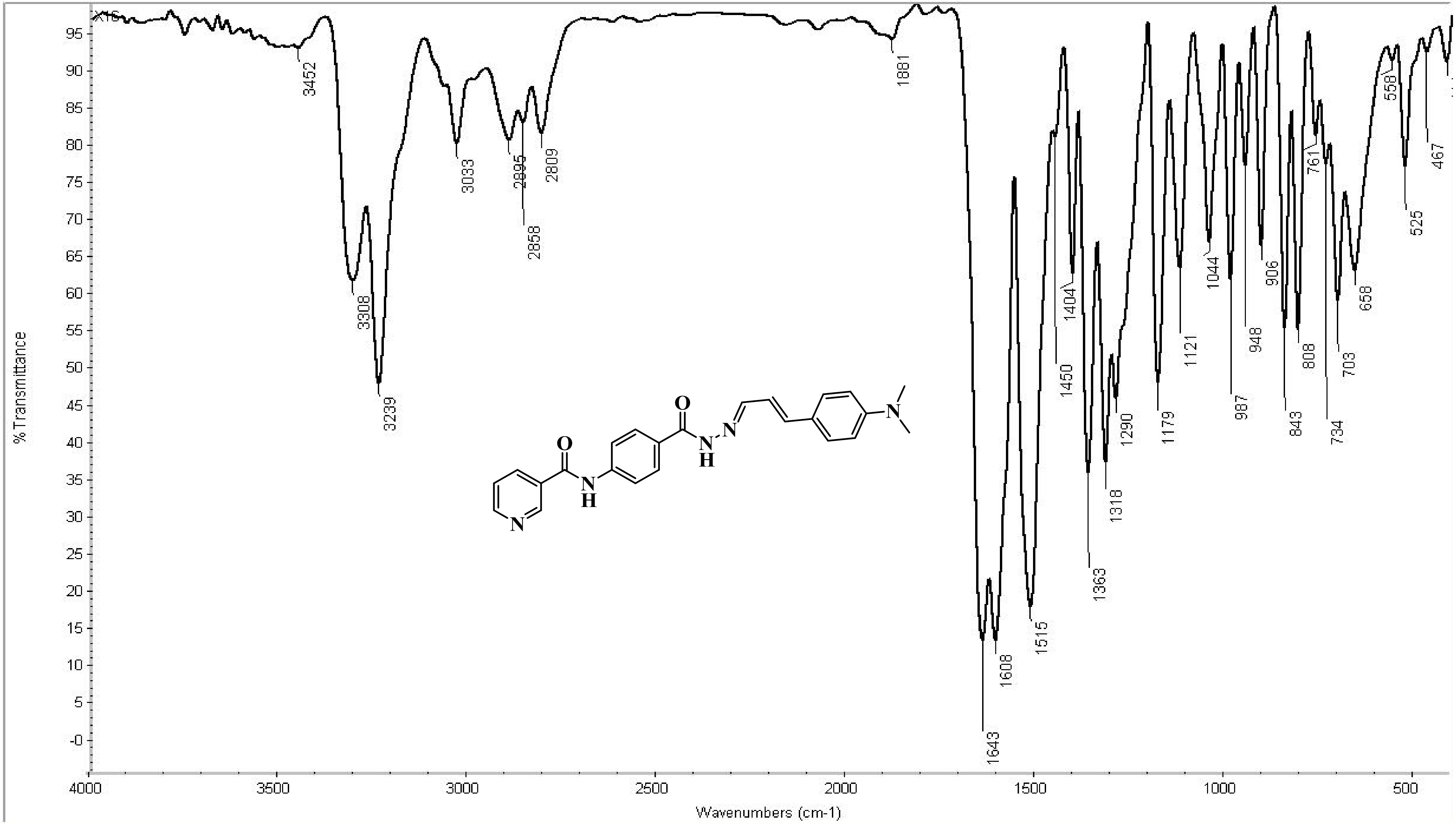

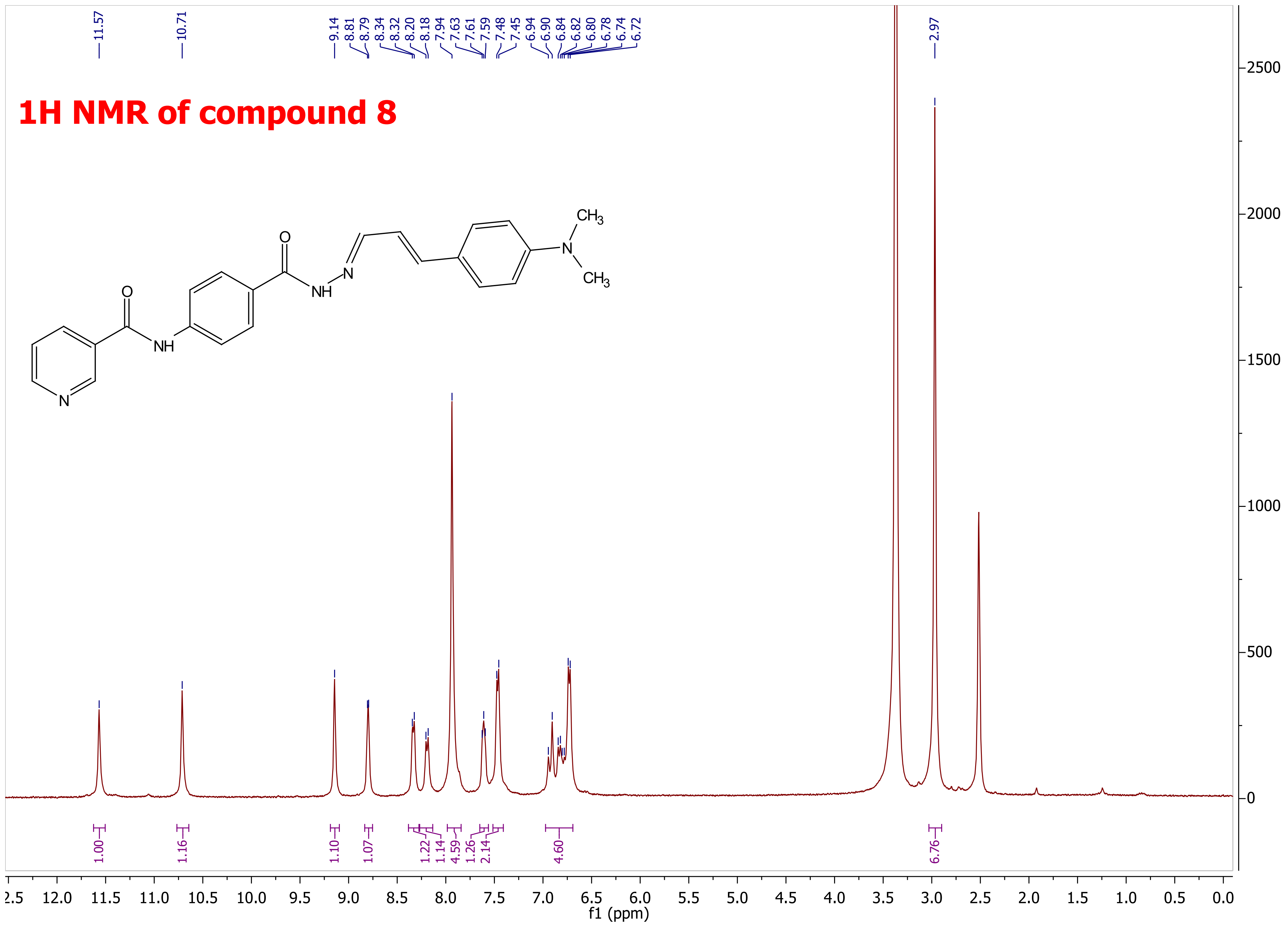
1H NMR of compound 8
11.57
10.71
9.14
8.81
8.79
8.34
8.32
8.20
8.18
7.94
7.63
7.61
7.59
7.48
7.45
6.94
6.90
6.84
6.82
6.80
6.78
6.74
6.72
2.97
O
O
NH
NH
N
N
N
CH3
CH3
1.00
1.16
1.10
1.07
1.22
1.14
4.59
1.26
2.14
4.60
6.76
f1 (ppm)

1H NMR of compound 8

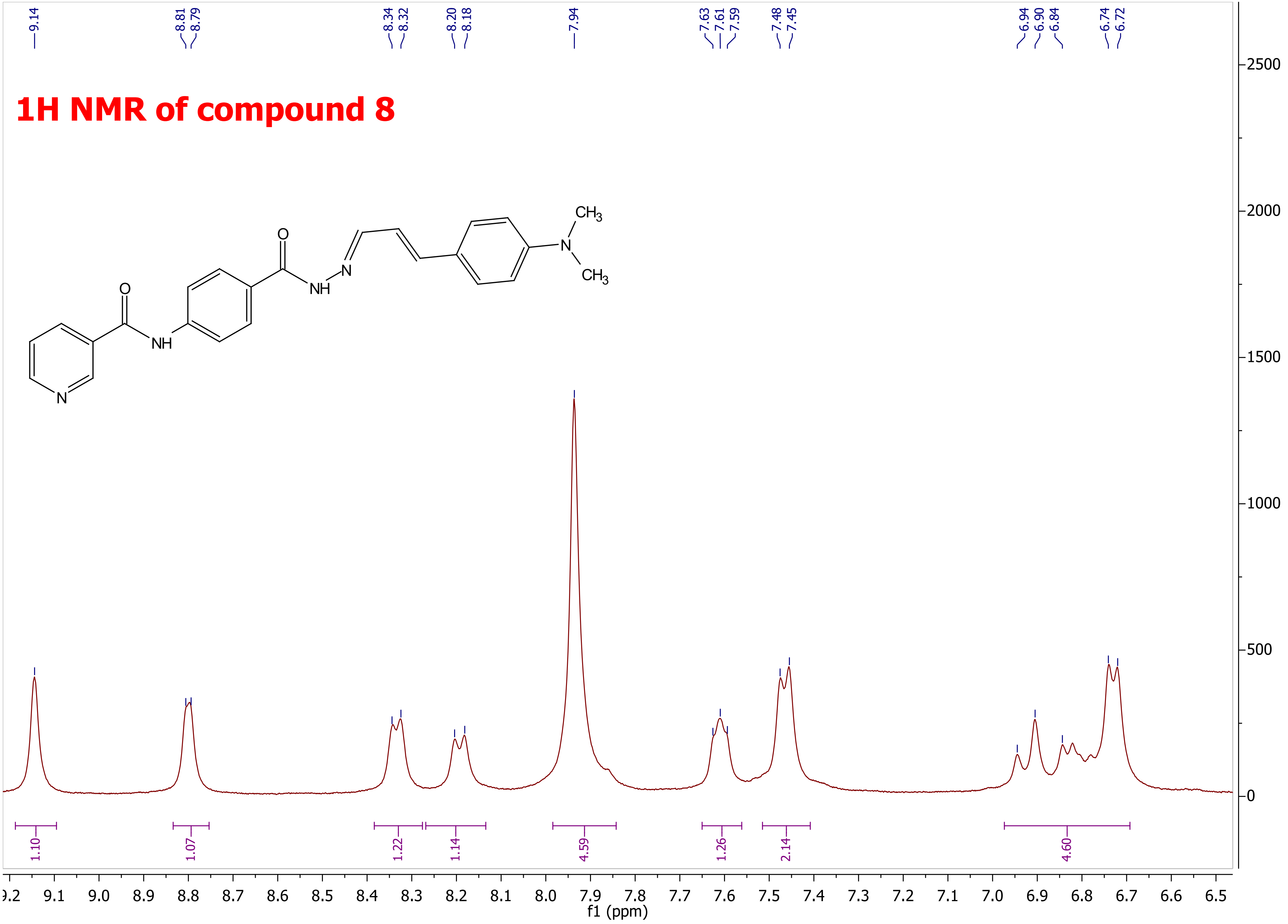

13C NMR of compound 8

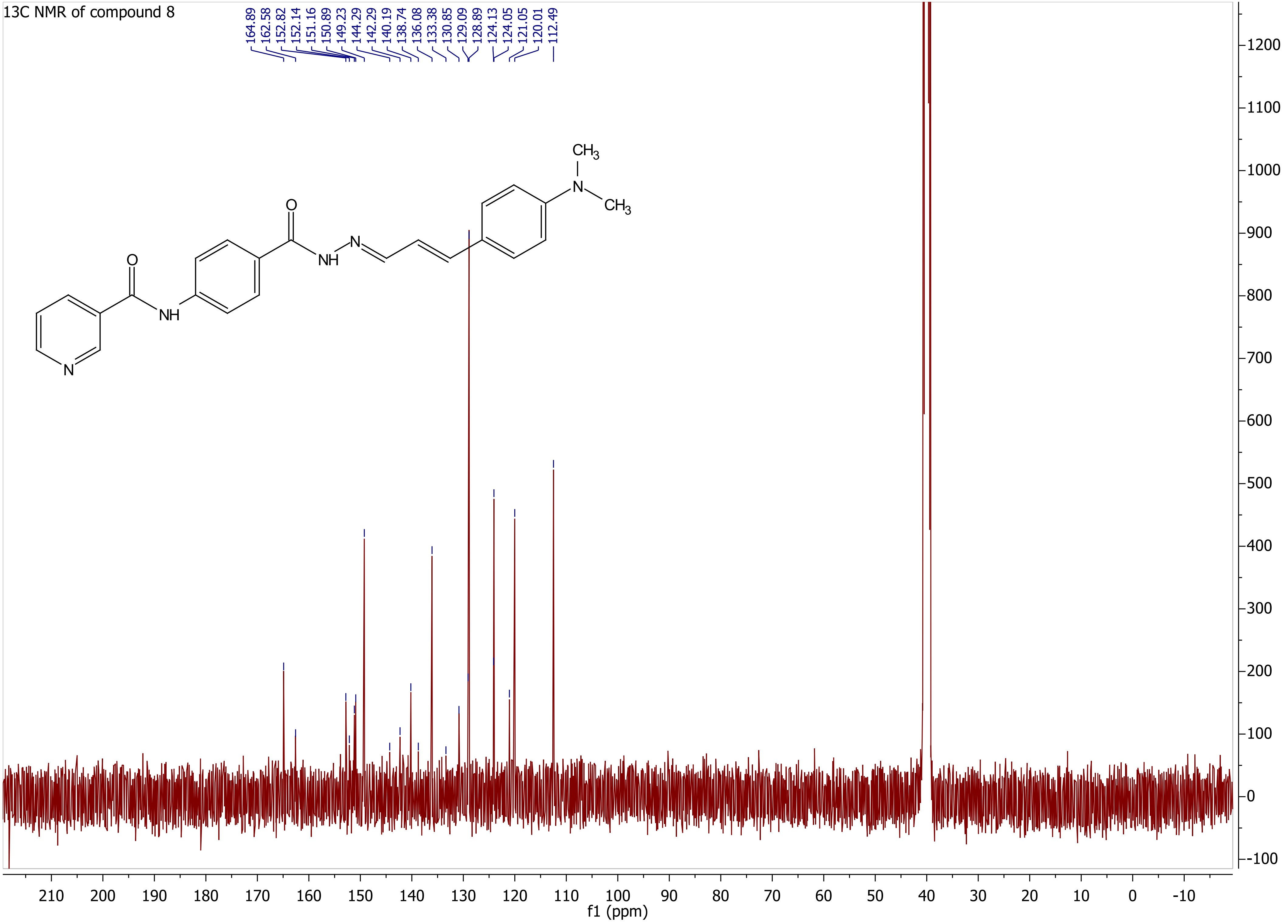

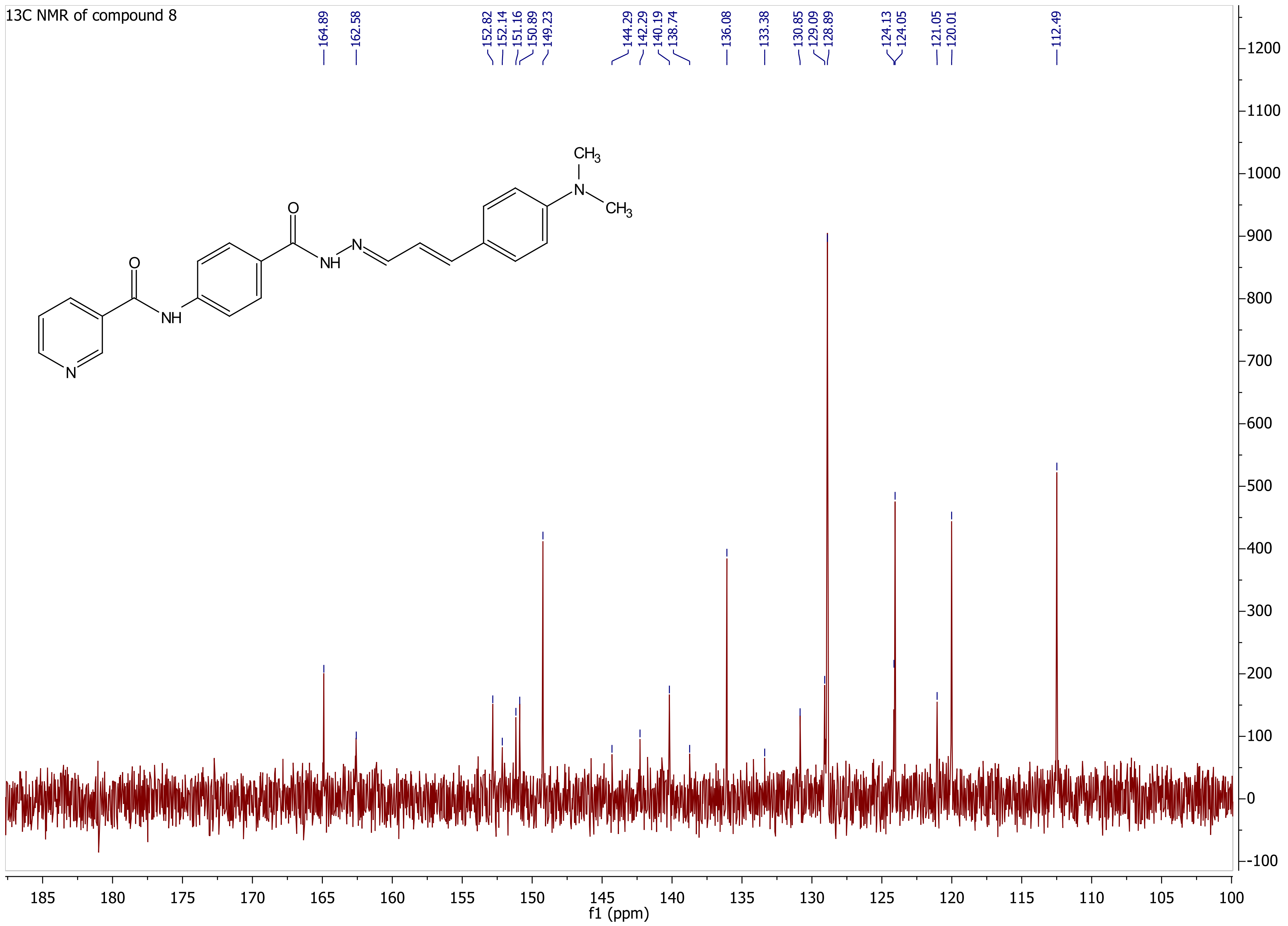
13C NMR of compound 8
164.89
162.58
152.82
152.14
151.16
150.89
149.23
144.29
142.29
140.19
138.74
136.08
133.38
130.85
129.09
128.89
124.13
124.05
121.05
120.01
112.49
f1 (ppm)

IR of compound 11

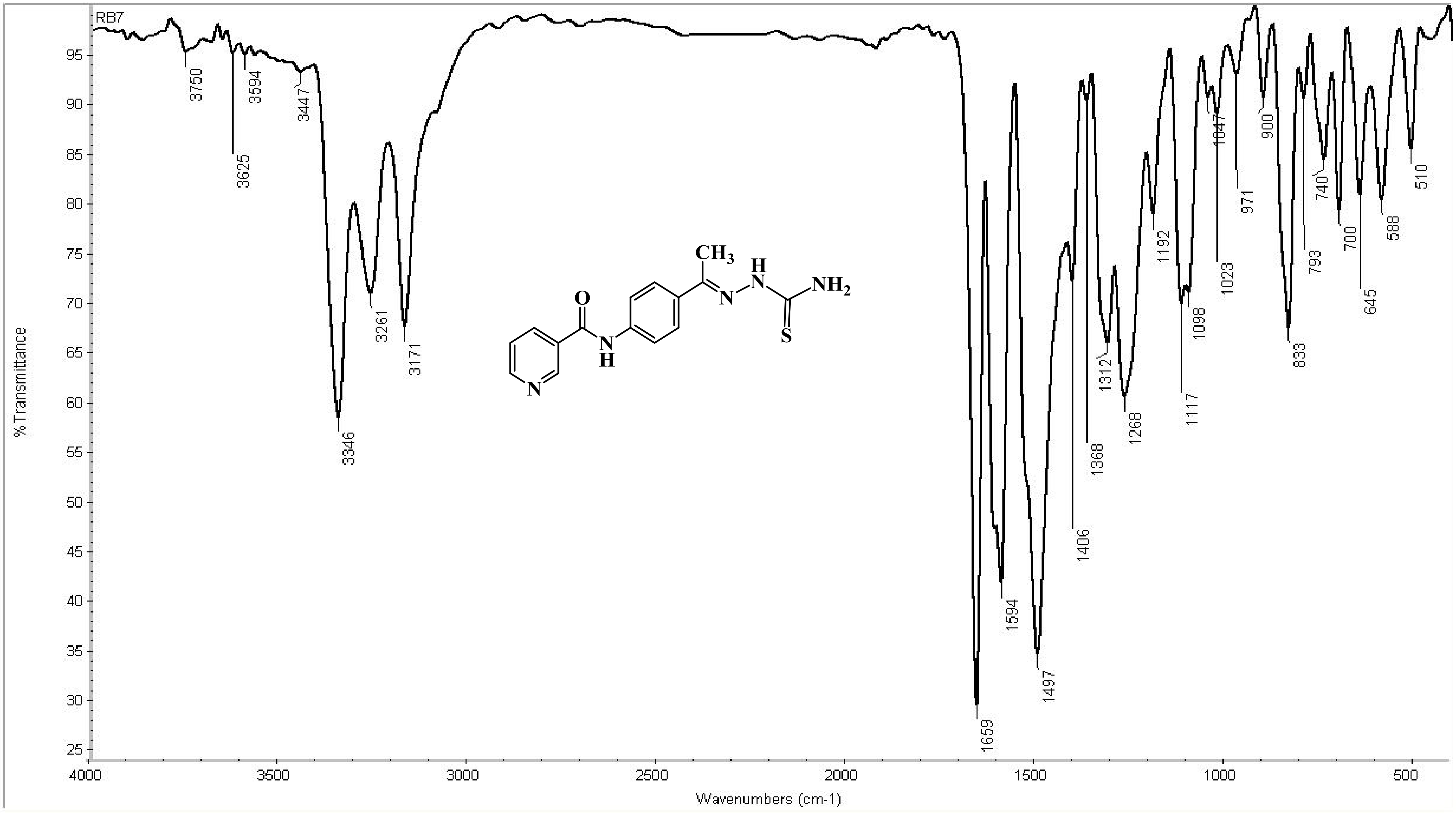

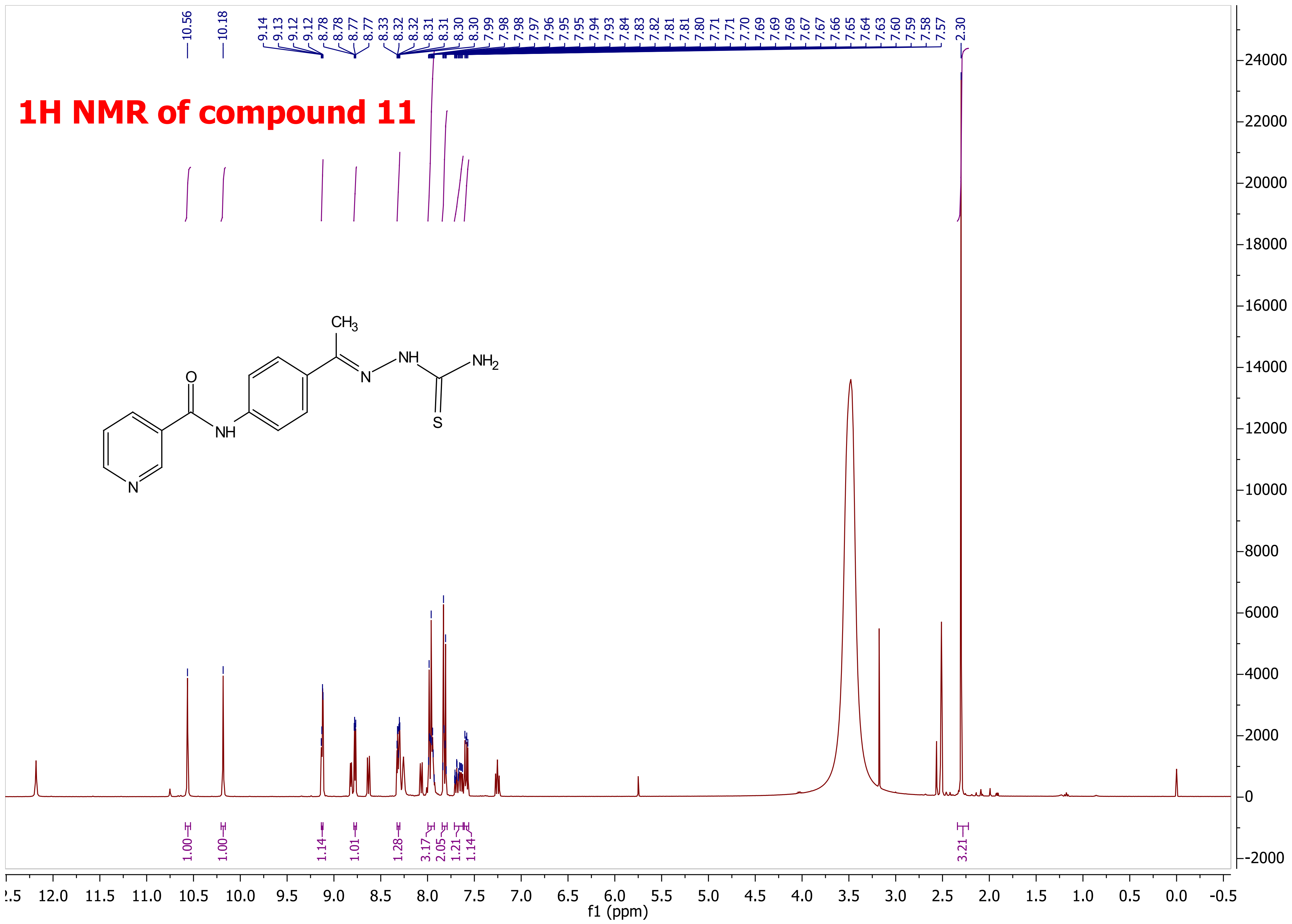
1H NMR of compound 11
10.56
10.18
9.14
9.13
9.12
9.12
8.78
8.78
8.77
8.77
8.33
8.32
8.32
8.31
8.31
8.30
8.30
7.99
7.98
7.98
7.97
7.96
7.95
7.95
7.94
7.93
7.84
7.83
7.82
7.81
7.81
7.80
7.71
7.71
7.70
7.69
7.69
7.69
7.67
7.67
7.66
7.65
7.64
7.63
7.60
7.59
7.58
7.57
2.30
1.00
1.00
1.14
1.01
1.28
3.17
2.05
1.21
1.14
3.21
CH3
NH
NH2
O
N
NH
S
N
f1 (ppm)

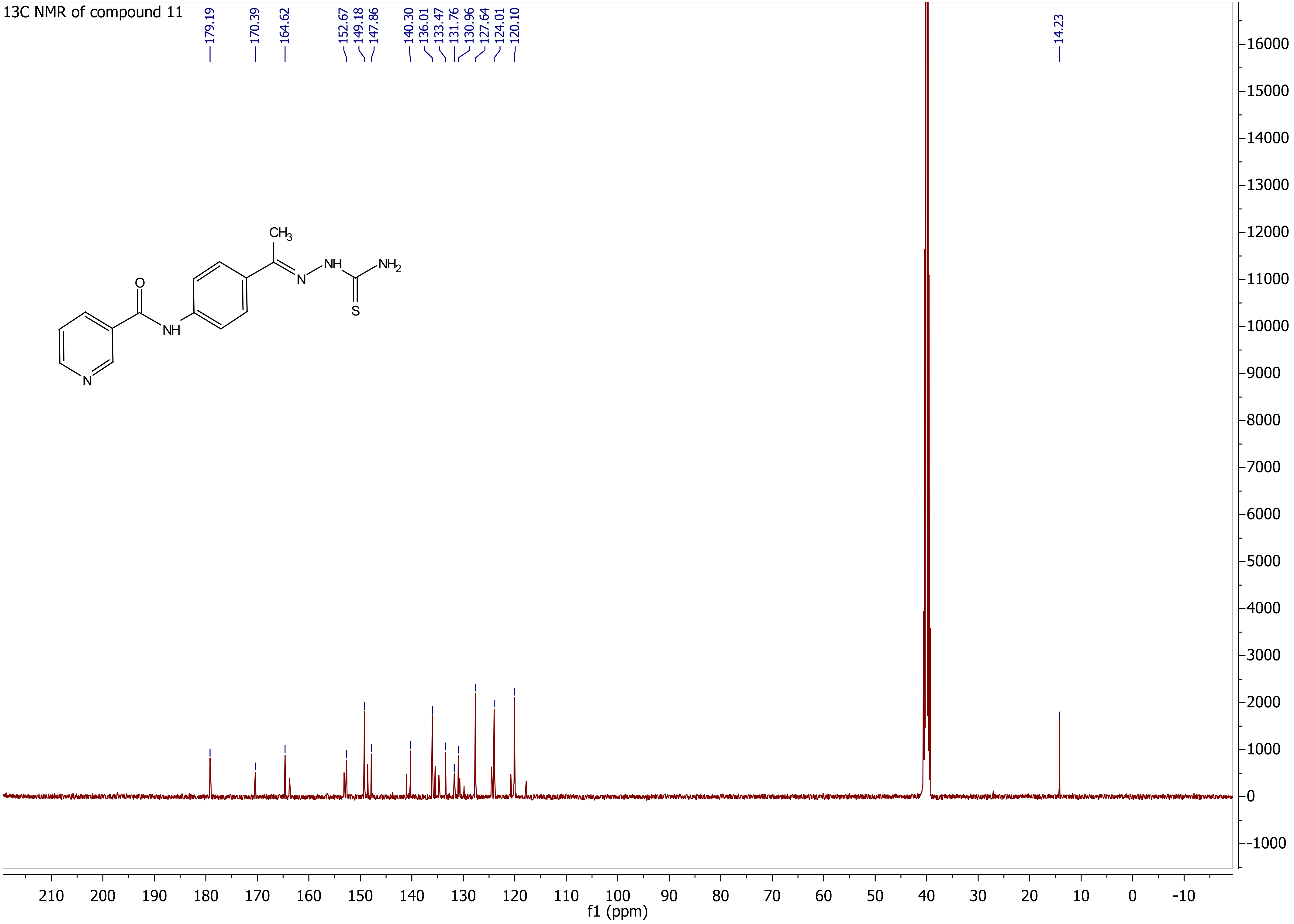
13C NMR of compound 11
179.19
170.39
164.62
152.67
149.18
147.86
140.30
136.01
133.47
131.76
130.96
127.64
124.01
120.10
14.23
CH3
NH
NH2
N
O
NH
S
N
f1 (ppm)

IR of compound 12

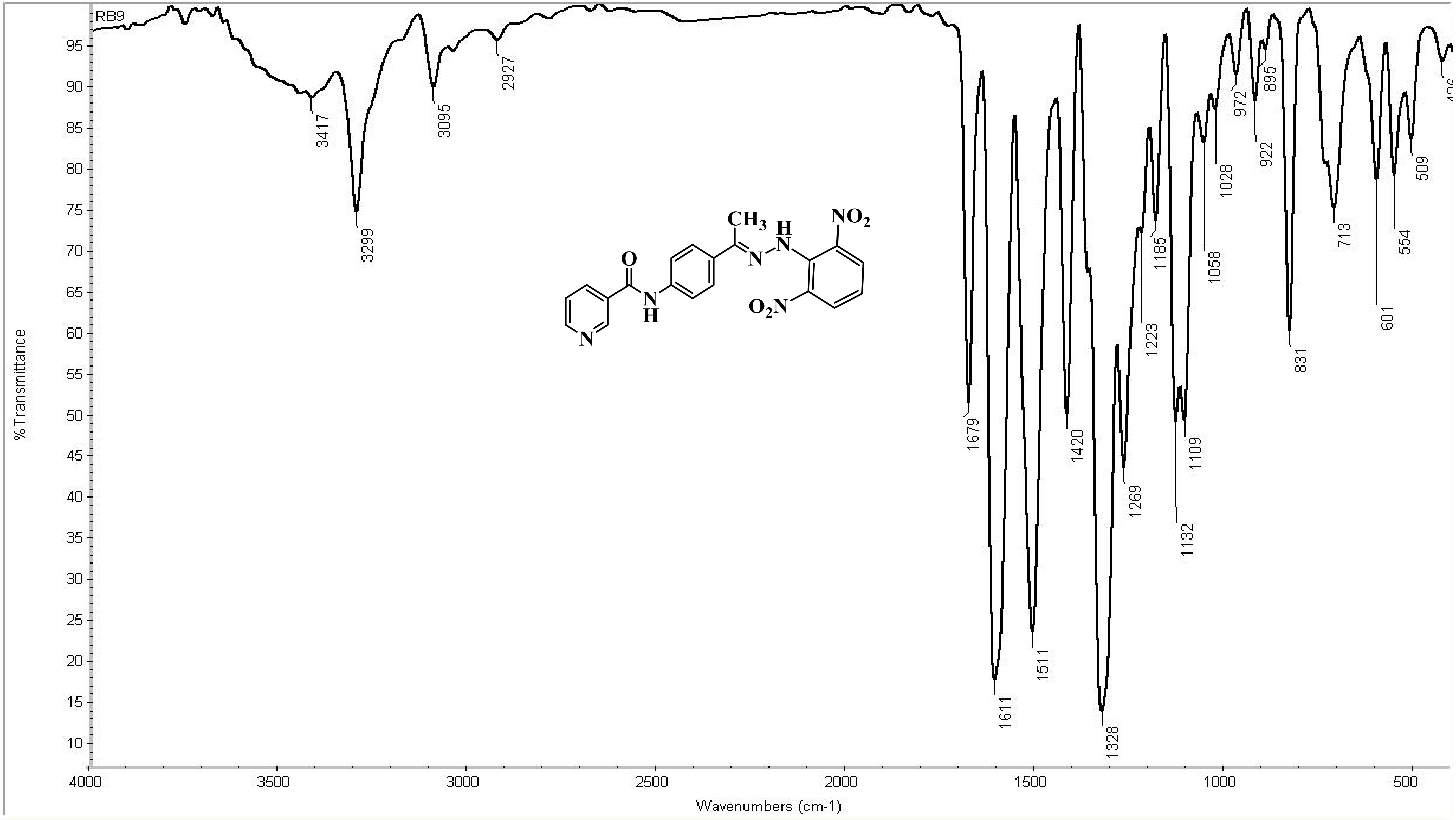

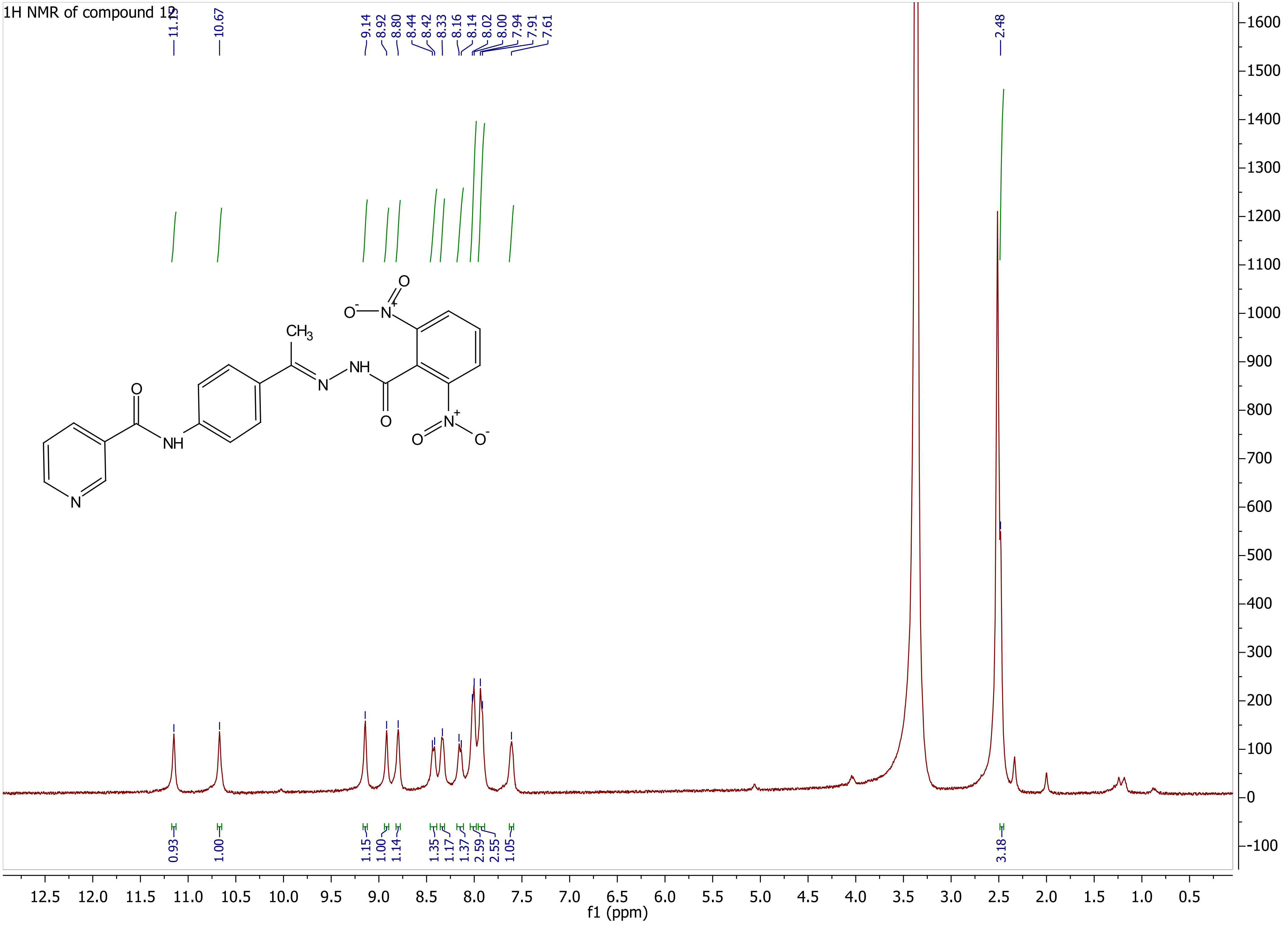
1H NMR of compound 12
11.15
10.67
9.14
8.92
8.80
8.44
8.42
8.33
8.16
8.14
8.02
8.00
7.94
7.91
7.61
2.48
CH3
O
NH
N
NH
O
O
N+
O-
N+
O
O-
N
0.93
1.00
1.15
1.00
1.14
1.35
1.17
1.37
2.59
2.55
1.05
3.18
12.5
12.0
11.5
11.0
10.5
10.0
9.5
9.0
8.5
8.0
7.5
7.0
6.5
6.0
5.5
5.0
4.5
4.0
3.5
3.0
2.5
2.0
1.5
1.0
0.5
f1 (ppm)
1600
1500
1400
1300
1200
1100
1000
900
800
700
600
500
400
300
200
100
0
-100

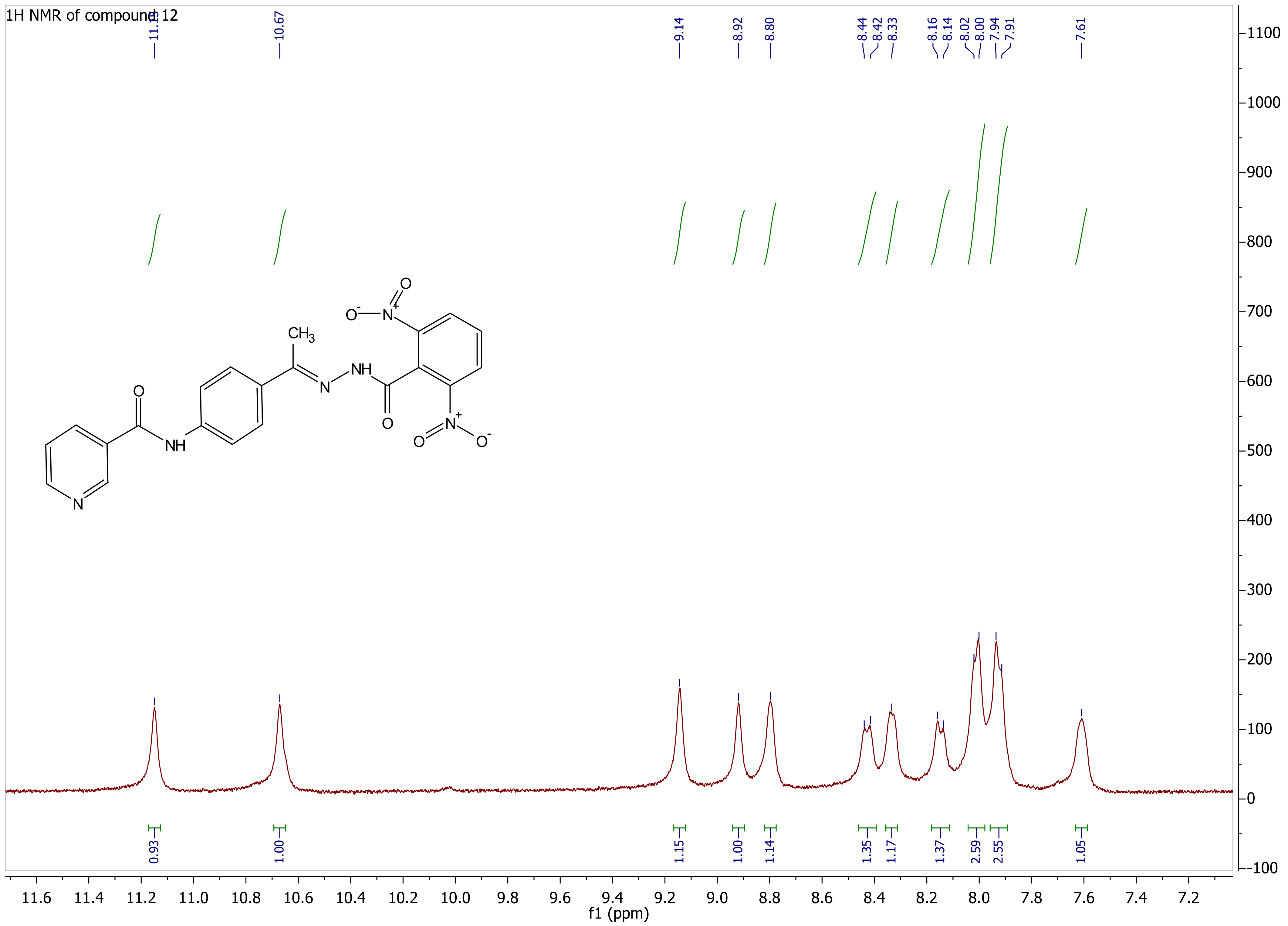
1H NMR of compound 12
10.67
9.14
8.92
8.80
8.44
8.42
8.33
8.16
8.14
8.02
8.00
7.94
7.91
7.61
0.93
1.00
1.15
1.00
1.14
1.35
1.17
1.37
2.59
2.55
1.05
f1 (ppm)
CH3
NH
N
O
NH
N
O
N+
O-
O
N+
O
O-

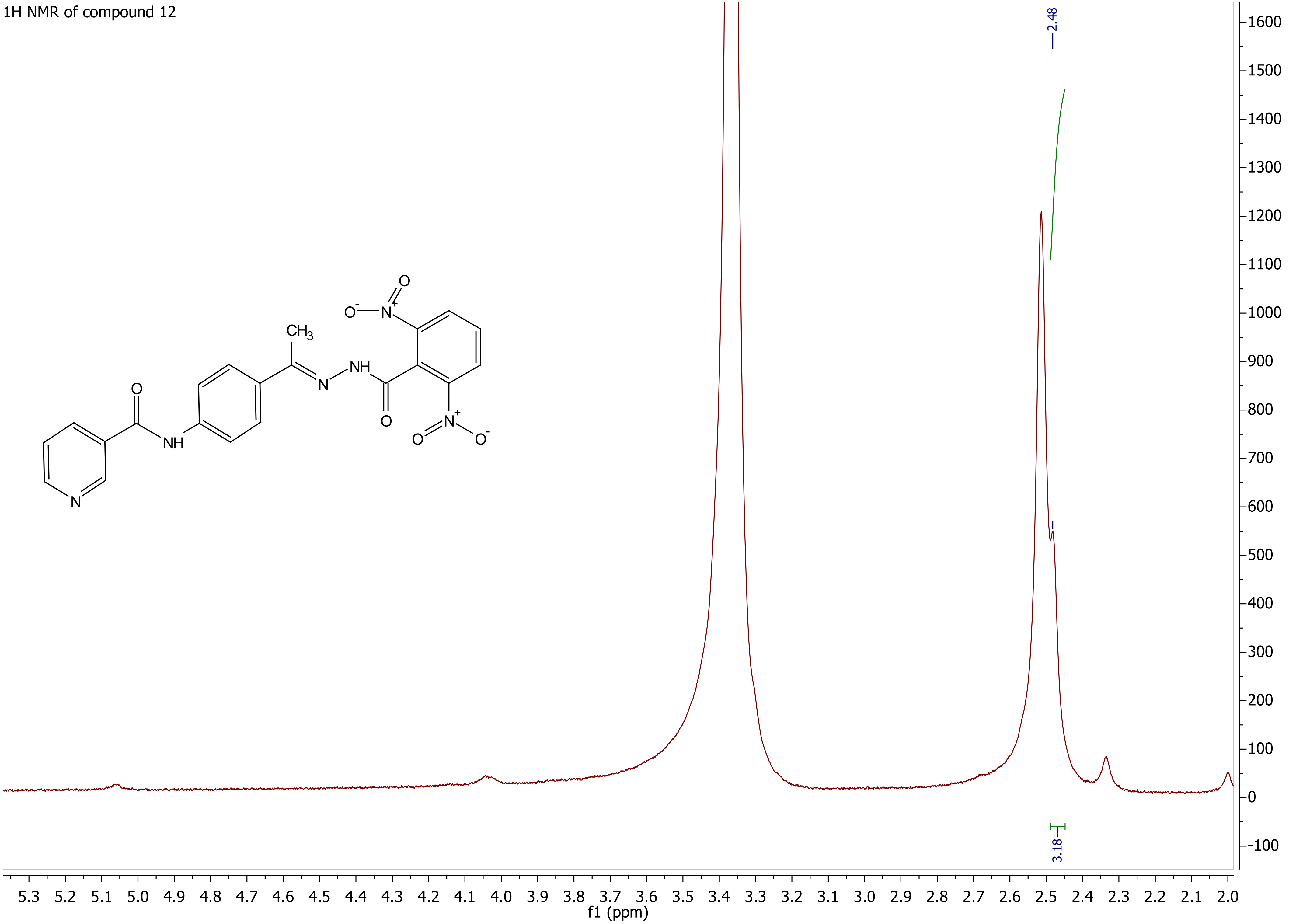

1H NMR of compound 12
2.48
3.18
f1 (ppm)
5.3 5.2 5.1 5.0 4.9 4.8 4.7 4.6 4.5 4.4 4.3 4.2 4.1 4.0 3.9 3.8 3.7 3.6 3.5 3.4 3.3 3.2 3.1 3.0 2.9 2.8 2.7 2.6 2.5 2.4 2.3 2.2 2.1 2.0
1600 1500 1400 1300 1200 1100 1000 900 800 700 600 500 400 300 200 100 0 -100

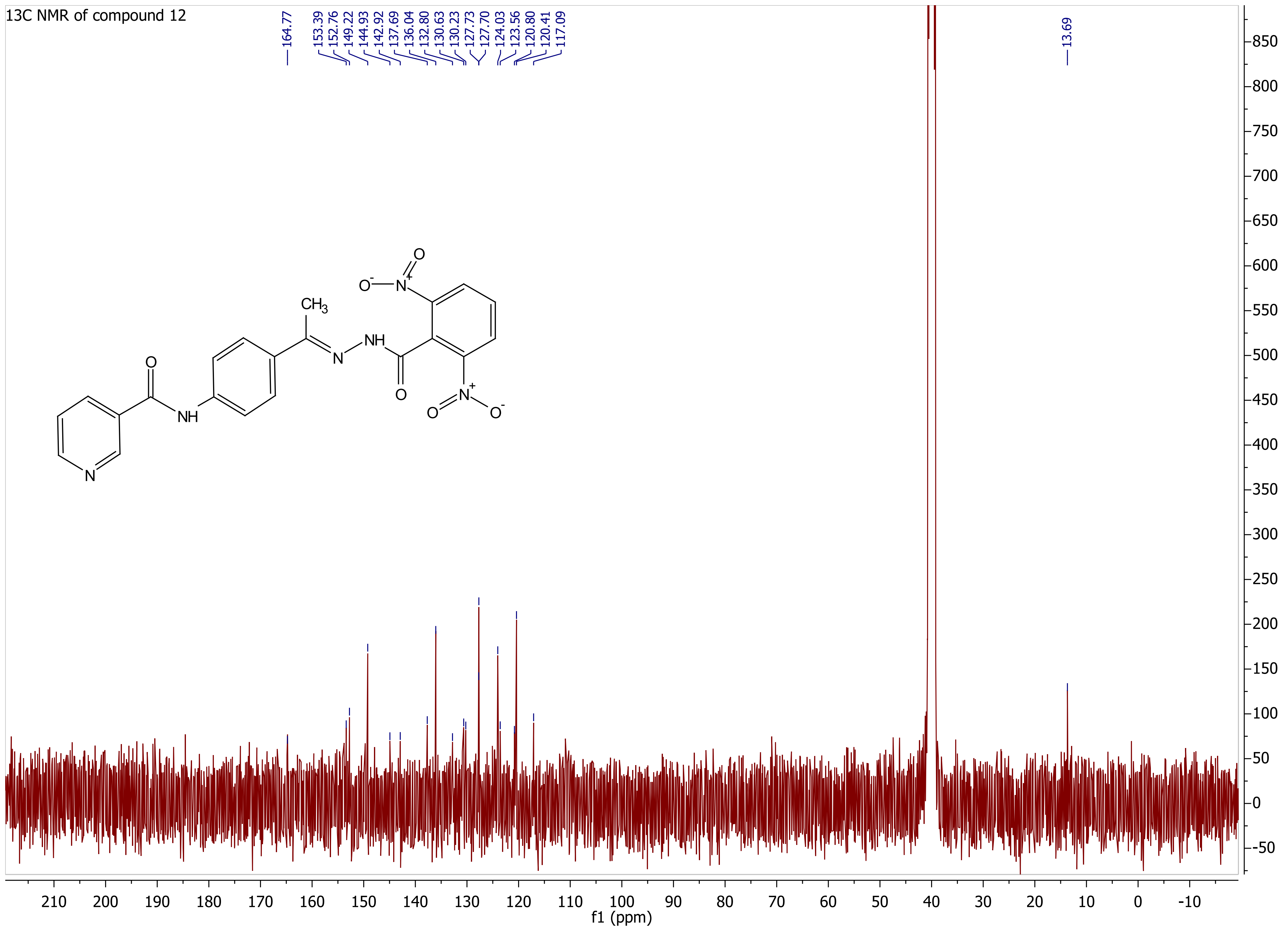
13C NMR of compound 12
164.77
153.39
152.76
149.22
144.93
142.92
137.69
136.04
132.80
130.63
130.23
127.73
127.70
124.03
123.56
120.80
120.41
117.09
13.69
f1 (ppm)
CH3
NH
N
O

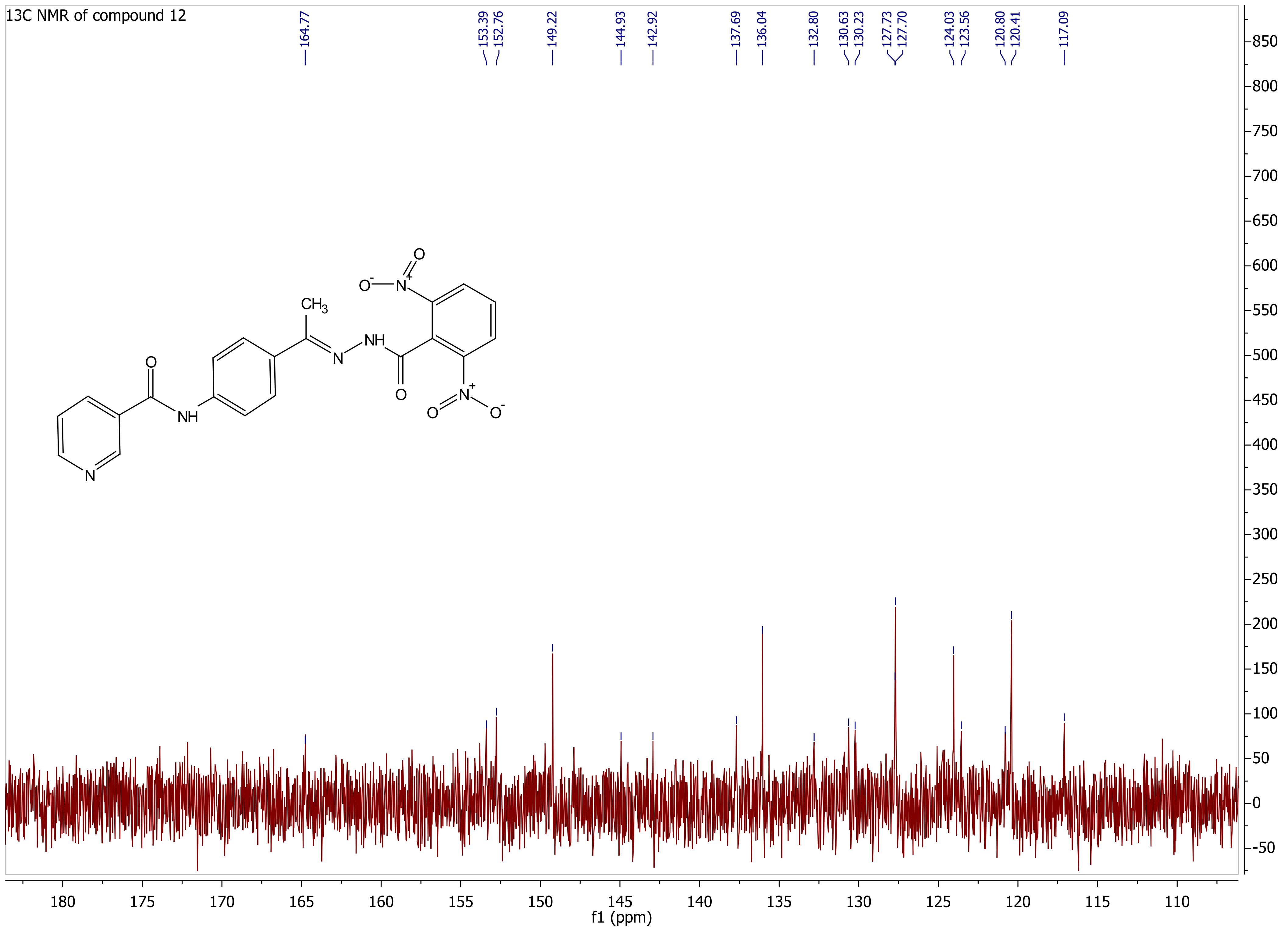
13C NMR of compound 12
164.77
153.39
152.76
149.22
144.93
142.92
137.69
136.04
132.80
130.63
130.23
127.73
127.70
124.03
123.56
120.80
120.41
117.09
CH3
NH
N
O
O-
N+
NH
O
N+
O
O-
O
f1 (ppm)

3- 4.3. Experimental of biological testing

4.3.1. In vitro anti-proliferative activity

The in vitro antiproliferative activities of the synthesized compounds were evaluated against two human tumor cell lines: hepatocellular carcinoma (HepG2) and breast cancer (MCF-7) using MTT assay protocol The commercially available sorafenib were used in this test as a positive control. The tested cell lines were purchased and dropped on the appropriate growth medium. The growth medium was supplemented with 100 mg/mL of streptomycin, 100 units/mL of penicillin and 10% of heat-inactivated fetal bovine serum in a humidified 5% (v/v) CO2 atmosphere at 37 ◦C. Then the cells from the two cancer cell lines were seeded at the appropriate densities into 96-well microtiter plates. After incubation for 24 h, the growth medium of each cell was treated with graded concentrations (1, 5, 10, 25, 50 µM) of the test compounds and incubated for three days. The viability of treated cells was determined using 3-[4,5-dimethylthiazole-2-yl]- 2,5-diphenyltetrazolium bromide (MTT) technique as cells were stained with 5% MTT solution and allowed to break down the dye into colored-insoluble formazan crystals for 4 h. The formazan crystals were dissolved in acidified isopropanol for 30 min with continuous shaking at room temperature. The colorimetric assay was measured and recorded at absorbance of 570 nm. The cell viability was expressed as percentage of control and the concentration that induces 50% of maximum inhibition of cell proliferation considering control group as 100% viability and group treated with a mixture of toxic compounds as 0% viability. (IC50) were determined for each compound using Graph Pad Prism version 5 software by plotting of log C against % viability.

4.3.2. In vitro VEGFR-2 kinase assay

The synthesized compounds were estimated for their in vitro inhibition on human VEGFR-2 in HepG2 cell line; using ELISA kit. Firstly, a plate was used for the assay had been coated by an antibody specific for human VEGFR-2 enzyme, Sorafenib was nominated as a standard VEGFR-2 inhibitor. Both standard and samples were added to the wells and incubated overnight at 4 ◦C, then washed. The biotinylated antibody was

supplemented and further incubated for 1 h at room temperature. The unreacted, liberated antibody was then washed; followed by addition of HRP-conjugated streptavidin and incubated for 45 min at room temperature. Wells were washed and a TMB substrate solution was added and kept at room temperature for 30 min. Finally, the stop solution was added, and the intensity of the color produced was measured at 450 nm . Concentration-inhibition response curve was established by GraphPad Prism 5.0. The IC50 value was calculated as the concentration at which 50% of the cells could survive in comparison to sorafenib.

4.3.3. Flow cytometry analysis for cell cycle

Cell cycle analysis for the most potent candidate **27a** was carried out through Flow cytometric analysis. In this test HepG2 cells were supplemented with the test compound at its cytotoxic concentration, seeded and subjected for incubation for 24 h at 37 ◦C and 5% CO2. Cells were washed twice with phosphate buffer saline, then the centrifugation of cell pellets had been completed, followed by preservation with ice-cold 70% ethanol for 15 min. Pellets were collected again and incubated with propidium iodide (PI) staining solution. After incubation for one hour at room temperature, it was analyzed by flowcytometry on an FC500 cytometer (Beckman Coulter) and the cell cycle distributions were calculated.

4.3.4. Flow cytometry analysis for apoptosis

Flow cytometry cell apoptosis analysis was used to investigate the apoptotic effect of the most active compound **27a** in HepG2 cells, the examination depends on that AnnexinV-fluorescein isothiocyanate (Annexin V-FITC) is a protein that has high affinity to phosphatidyl serine PS, which can be detected by staining with Annexin V-(FITC) and counter staining with propidium iodide (PI). In such procedure, HepG2 cells were incubated together with the tested compound (100 mL) for 24 h. Then, the cells were centrifuged, collected by trypsin, washed with PBS two successive times, and suspended in 500 mL of binding buffer. The double staining Annexin V-FITC (5 mL) and PI (5 mL) were added to the cells and incubated in the dark for 5 min at room temperature. The stained cells were analyzed for flow cytometry using FACS caliber flow cytometer and apoptosis detection kit (BD Biosciences, San Jose, CA)

4.3.5. Quantitative Real Time Reverse-Transcriptase PCR technique

The quantity of BAX, Bcl-2, and caspase3 mRNA in control and Ay17 (at the IC_{50} concentration)-treated HepG2 cells was assessed by qRT-PCR (reference). Total RNA from vehicle-treated control (0.01% DMSO) and 10k-treated HepG2 cells were extracted as-per the manufacturer instructions (RNeasy mini kit, Qiagen, Germany). After RNA extraction, cDNA was prepared using the Revert Aid First Strand cDNA Synthesis kit (Thermo Scientific, USA). Amplification of target cDNA for apoptosis markers and GAPDH [as a normalization (housekeeping) gene] was done using one-step RT-PCR SYBR® Green kit Master Mix (Bio-Rad Laboratories, USA) on Rotor-Gene Q real-time PCR thermal cycler instrument. cDNA (2 µl aliquots) was mixed with 1 µl of forward primer, 1 µl reverse primer, 10 µl master mixture, and the reaction volume was completed to 20 µl with nuclease-free water. All experiments were performed in triplicates

The sequences for primers used in quantitative Real Time Reverse-Transcriptase PCR (qRT-PCR)

**Gene**	**Primer sequence**
**BAX**	F: 5'- CCCGAGAGGTCTTTTTCCGAG -3'
	R: 5'- CCAGCCCATGATGGTTCTGAT -3'
**Bcl-2**	F: 5'- TTGTGGCCTTCTTTGAGTTCGGTG -3'
	R: 5'- GGTGCCGGTTCAGGTACTCAGTCA -3'
**Caspase-3**	F: 5'- ACATGGAAGCGAATCAATGGACTC -3'
	R: 5'- AAGGACTCAAATTCTGTTGCCACC -3'
**GABDH**	F: 5'- GACCCCTTCAT GACCTCAAC -3'
	R: 5'- CTTCTCCATGGTGGTGAAGA -3'

4- 4.4.Experimental of *in silico* studies

4.4.1. Docking studies

The docking studies were performed utilizing MOE.14 software to explore the binding mode of the synthesized compounds towards VEGFR-2. The 3D crystal structures of the target macromolecules VEGFR-2 were downloaded from the protein databank, http://www.pdb.org (PDB ID; 2OH4 and 4ASD). Sorafenib was used as reference ligand. To prepare the target protein, water molecules were removed, and the valances of atoms were corrected through protonation of the whole molecule. Then energy minimization was carried out by applying CHARMM and MMFF94 force fields. After that, the active binding site was defined and prepared for docking. The validation process was performed by redocking the co-crystallized ligand. The designed compounds together with sorafenib were drawn using ChemBioDraw Ultra 14.0 and saved as MDL-SD format. The sketched compounds were constructed from fragment libraries in MOE program, protonated, followed by energy minimization then prepared for docking. Docking process was carried through Triangle matcher placement inserted in compute window, and the scoring function was London dG. Ten conformers (poses) for each molecule were generated using genetic algorithm searches. The free energies and binding modes of the designed molecules against VEGFR-2 were determined. The most ideal pose was selected according to its binding free energy as well as its binding mode with target molecule.

4.4.2. ADMET studies

ADMET descriptors (absorption, distribution, metabolism, excretion and toxicity) of the synthesized compounds were determined using Discovery studio 4.0. At first, the CHARMM force field was applied then the compounds were prepared and minimized according to the preparation of small molecule protocol. Then ADMET descriptors protocol was applied to carry out these studies.

4.4.3. Toxicity studies

The toxicity parameters of the synthesized compounds were calculated using Discovery studio 4.0. Sorafenib was used as a reference drug. At first, the CHARMM force field was applied then the compounds were prepared and minimized according to the preparation of small molecule protocol. Then different parameters were calculated from toxicity prediction (extensible) protocol.

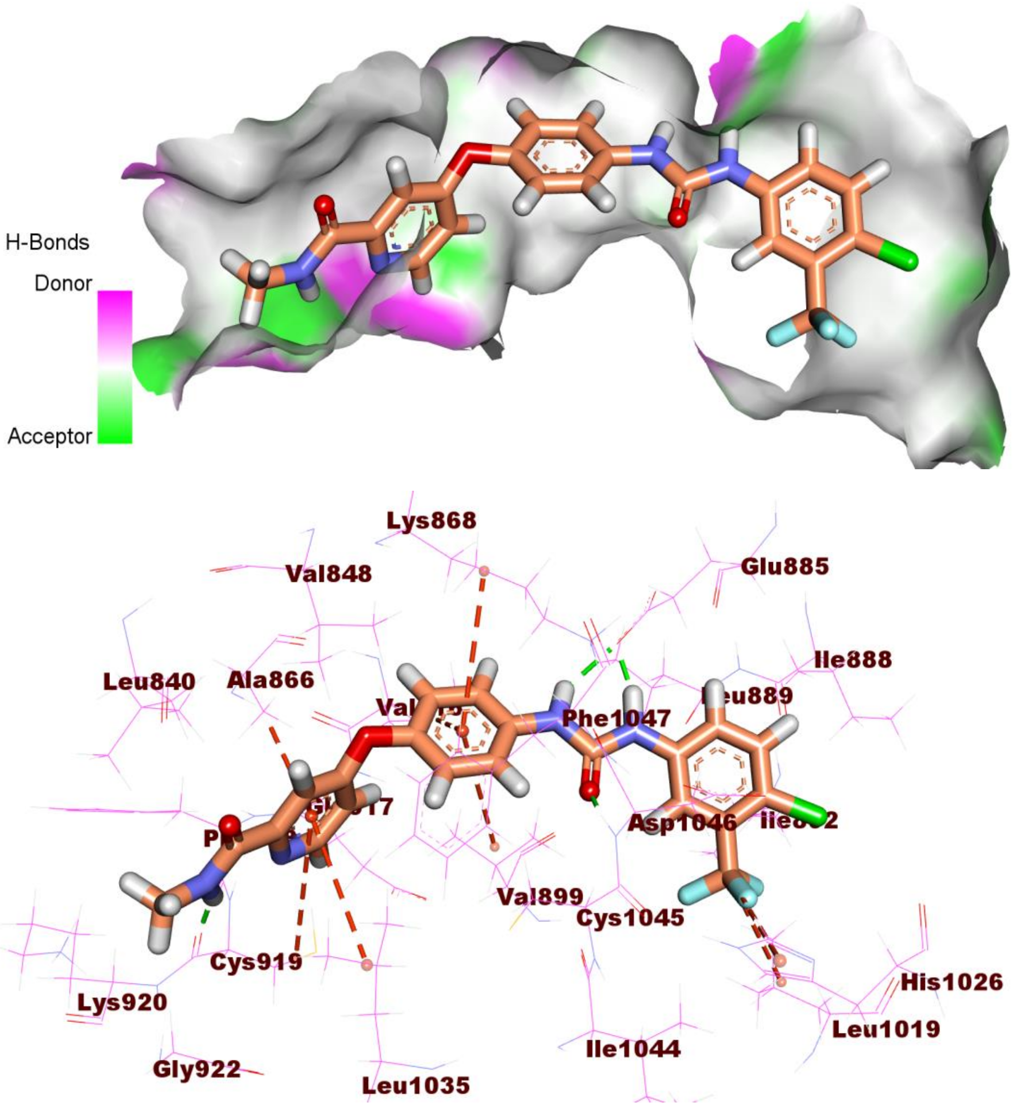

Binding mode of sorafenib into VEGFR-2 active site

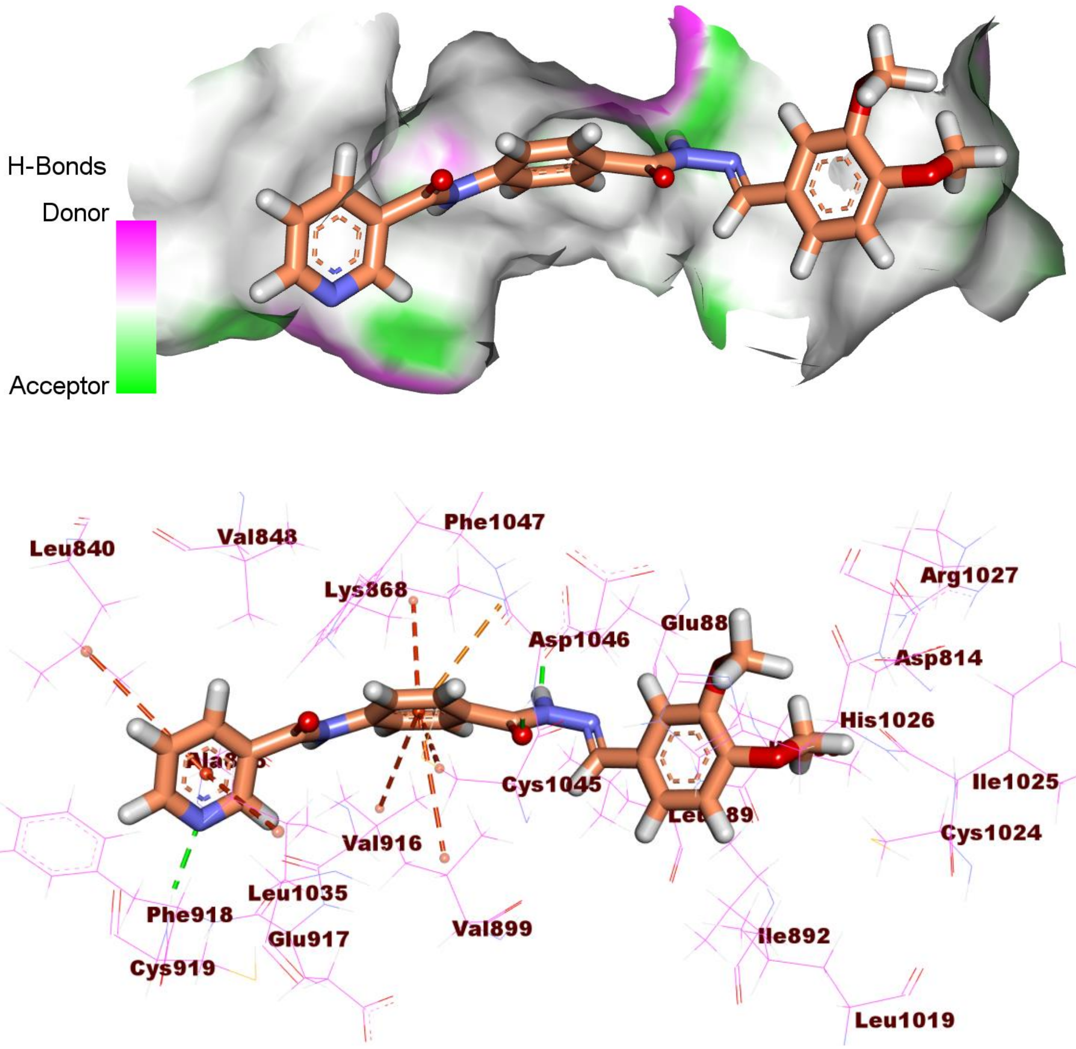

Binding mode of compound **7** into VEGFR-2 active site

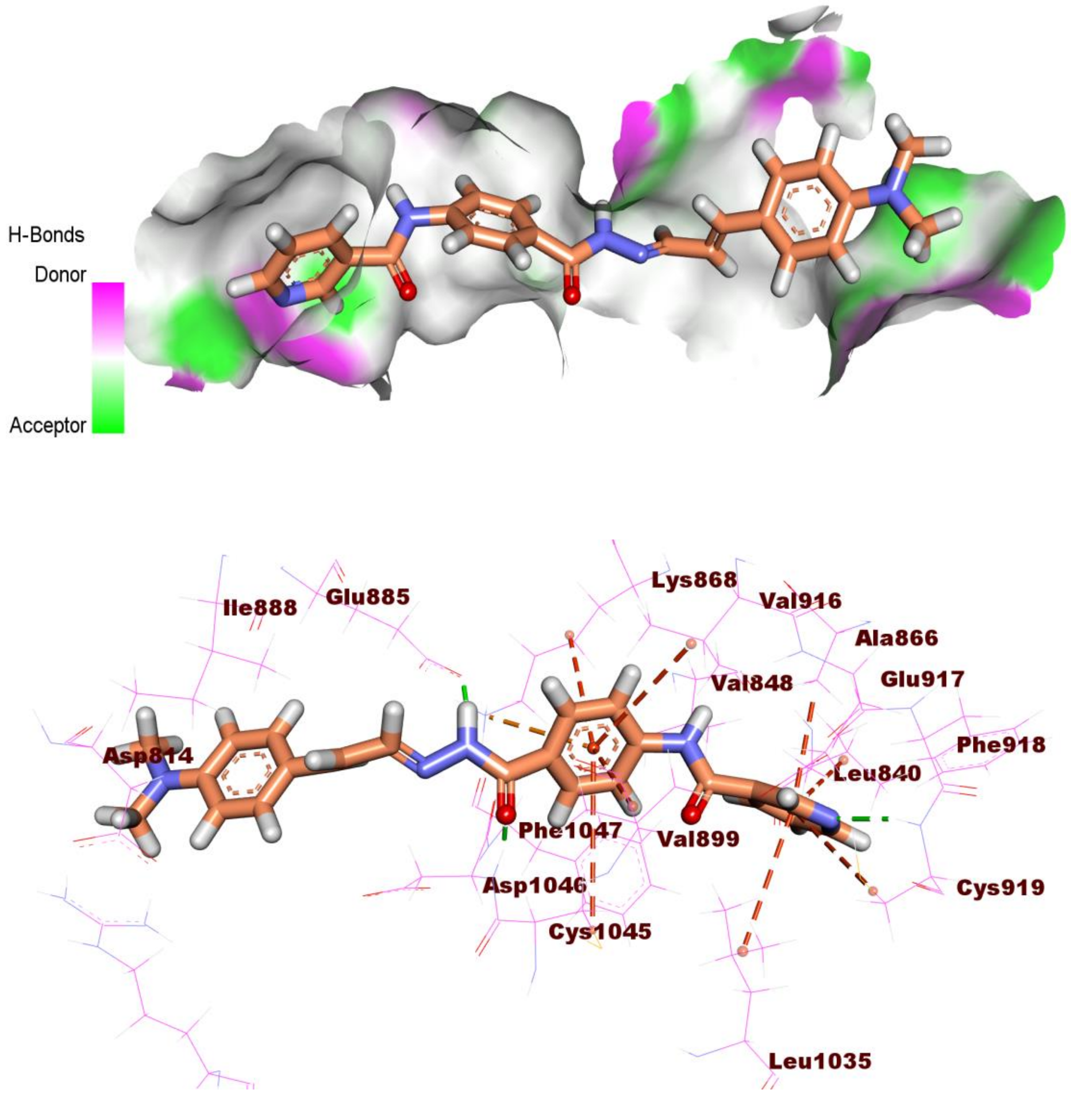

Binding mode of compound **8** into VEGFR-2 active site

1- Molecular Dynamics (MD) Simulations

The system was prepared using the web-based CHARMM-GUI[1-3] interface with the CHARMM36 force field[4]. All the simulations were done using the NAMD 2.13[5] package. The TIP3P explicit solvation model was used[6], and the periodic boundary conditions were set with a dimension of 82.65 Å, 82.36 Å, and 82.64 Å in x, y, and z, respectively. The parameters for the top docking results were generated using the CHARMM general force field[7] Afterward, the system was neutralized using required number of Cl^-/Na^+ ions. The MD protocols involved minimization, equilibration, and production. a 2 *fs* time step of integration was chosen for all MD simulations, the equilibration was carried in the canonical (*NVT*) ensemble, while the isothermal–isobaric (*NPT*) ensemble was for the production. Through the 100 *ns* of MD production, the pressure was set at 1 atm using the Nose´–Hoover Langevin piston barostat [8, 9] with a Langevin piston decay of 0.05 *ps* and a period of 0.1 *ps*. The temperature was set at 298.15 K using the Langevin thermostat[10]. A distance cutoff of 12.0 Å was applied to short-range nonbonded interactions with a pair list distance of 16 Å, and Lennard Jones interactions were smoothly truncated at 8.0 Å. Long-range electrostatic interactions were treated using the particle-mesh Ewald (PME) method[11, 12], where a grid spacing of 1.0 Å was used for all simulation cells. All covalent bonds involving hydrogen atoms were constrained using the SHAKE algorithm[13]. For consistency, we have applied the same protocol for all MD simulations.

2- Molecular Mechanics Poisson–Boltzmann Surface Area (MM-PBSA)

The Molecular Mechanics Poisson–Boltzmann surface area (MM-PBSA) [14, 15] approach implemented in the MOLAICAL code[16] was used for the relative binding energy calculations, in which the ligand (L) binds to the protein receptor (R) to form the complex (RL),

$$\Delta G_{bind} = \Delta G_{RL} - \Delta G_{R} - \Delta G_{L}$$

which can be represented by contributions of different interactions,

$$\Delta G_{bind} = \Delta H - T\Delta S = \Delta E_{MM} + \Delta G_{Sol} - T\Delta S$$

where the changes in the gas phase molecular mechanics $\left(\Delta E_{MM}\right)$, solvation Gibbs energy $\left(\Delta G_{Sol}\right)$, and conformational entropy $\left(-T\Delta S\right)$ are determined as follows: ΔE_{MM} is the sum of the changes in the electrostatic energies ΔE_{ele}, the van der Waals energies ΔE_{vdW}, and the internal energies ΔE_{int} (bonded interactions); ΔG_{Sol} is the total of both the polar solvation (calculated using the generalized Born model) and the nonpolar solvation (calculated using the solvent-accessible surface area) and $-T\Delta S$ is calculated by the normal mode analysis. The solvent dielectric constant of 78.5 and the surface tension constant of 0.03012 kJ mol^{-1} $Å^2$ were used for MM/GBSA calculations.

[1] S. Jo, T. Kim, V.G. Iyer, W. Im, CHARMM-GUI: A web-based graphical user interface for CHARMM, Journal of computational chemistry 29(11) (2008) 1859-1865.

[2] B.R. Brooks, C.L. Brooks III, A.D. Mackerell Jr., L. Nilsson, R.J. Petrella, B. Roux, Y. Won, G. Archontis, C. Bartels, S. Boresch, A. Caflisch, L. Caves, Q. Cui, A.R. Dinner, M. Feig, S. Fischer, J. Gao, M. Hodoscek, W. Im, K. Kuczera, T. Lazaridis, J. Ma, V. Ovchinnikov, E. Paci, R.W. Pastor, C.B. Post, J.Z. Pu, M. Schaefer, B. Tidor, R.M. Venable, H.L. Woodcock, X. Wu, W. Yang, D.M. York, M. Karplus, CHARMM: The biomolecular simulation program, Journal of computational chemistry 30(10) (2009) 1545-1614.

[3] J. Lee, X. Cheng, J.M. Swails, M.S. Yeom, P.K. Eastman, J.A. Lemkul, S. Wei, J. Buckner, J.C. Jeong, Y. Qi, S. Jo, V.S. Pande, D.A. Case, C.L. Brooks, A.D. MacKerell, J.B. Klauda, W. Im, CHARMM-GUI Input Generator for NAMD, GROMACS, AMBER, OpenMM, and CHARMM/OpenMM Simulations Using the CHARMM36 Additive Force Field, Journal of chemical theory and computation 12(1) (2016) 405-413.

[4] R.B. Best, X. Zhu, J. Shim, P.E. Lopes, J. Mittal, M. Feig, A.D. Mackerell, Jr., Optimization of the additive CHARMM all-atom protein force field targeting improved sampling of the backbone phi, psi and side-chain chi(1) and chi(2) dihedral angles, Journal of chemical theory and computation 8(9) (2012) 3257-3273.

[5] J.C. Phillips, R. Braun, W. Wang, J. Gumbart, E. Tajkhorshid, E. Villa, C. Chipot, R.D. Skeel, L. Kale, K. Schulten, Scalable molecular dynamics with NAMD, Journal of computational chemistry 26(16) (2005) 1781-802.

[6] W.L. Jorgensen, J. Chandrasekhar, J.D. Madura, R.W. Impey, M.L. Klein, Comparison of simple potential functions for simulating liquid water, The Journal of Chemical Physics 79(2) (1983) 926-935.

[7] W. Yu, X. He, K. Vanommeslaeghe, A.D. MacKerell, Jr., Extension of the CHARMM General Force Field to sulfonyl-containing compounds and its utility in biomolecular simulations, Journal of computational chemistry 33(31) (2012) 2451-68.

[8] S. Nosé, M.L. Klein, Constant pressure molecular dynamics for molecular systems, Molecular Physics 50(5) (1983) 1055-1076.

[9] S. Nosé, A molecular dynamics method for simulations in the canonical ensemble, Molecular Physics 52(2) (1984) 255-268.

[10] G.S. Grest, K. Kremer, Molecular dynamics simulation for polymers in the presence of a heat bath, Physical review. A, General physics 33(5) (1986) 3628-3631.
[11] T. Darden, D. York, L. Pedersen, Particle mesh Ewald: AnN·log(N) method for Ewald sums in large systems, The Journal of Chemical Physics 98(12) (1993) 10089-10092.
[12] U. Essmann, L. Perera, M.L. Berkowitz, T. Darden, H. Lee, L.G. Pedersen, A smooth particle mesh Ewald method, The Journal of Chemical Physics 103(19) (1995) 8577-8593.
[13] J.-P. Ryckaert, G. Ciccotti, H.J.C. Berendsen, Numerical integration of the cartesian equations of motion of a system with constraints: molecular dynamics of n-alkanes, Journal of Computational Physics 23(3) (1977) 327-341.
[14] S. Genheden, U. Ryde, Comparison of end-point continuum-solvation methods for the calculation of protein-ligand binding free energies, Proteins 80(5) (2012) 1326-42.
[15] E. Wang, H. Sun, J. Wang, Z. Wang, H. Liu, J.Z.H. Zhang, T. Hou, End-Point Binding Free Energy Calculation with MM/PBSA and MM/GBSA: Strategies and Applications in Drug Design, Chemical reviews 119(16) (2019) 9478-9508.
[16] Q. Bai, S. Tan, T. Xu, H. Liu, J. Huang, X. Yao, MolAICal: a soft tool for 3D drug design of protein targets by artificial intelligence and classical algorithm, Briefings in bioinformatics 22(3) (2021) bbaa161.

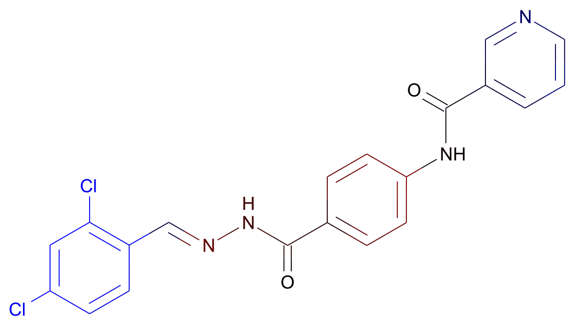

$C_{20}H_{14}Cl_2N_4O_2$
Molecular Weight: 413.25676
ALogP: 3.646
Rotatable Bonds: 5
Acceptors: 4
Donors: 2

Model Prediction

Prediction: Non-Mutagen
Probability: 0.587
Enrichment: 1.05
Bayesian Score: -5.39
Mahalanobis Distance: 7.53
Mahalanobis Distance p-value: 0.999

Prediction: Positive if the Bayesian score is above the estimated best cutoff value from minimizing the false positive and false negative rate.
Probability: The esimated probability that the sample is in the positive category. This assumes that the Bayesian score follows a normal distribution and is different from the prediction using a cutoff.
Enrichment: An estimate of enrichment, that is, the increased likelihood (versus random) of this sample being in the category.
Bayesian Score: The standard Laplacian-modified Bayesian score.
Mahalanobis Distance: The Mahalanobis distance (MD) is the distance to the center of the training data. The larger the MD, the less trustworthy the prediction.
Mahalanobis Distance p-value: The p-value gives the fraction of training data with an MD greater than or equal to the one for the given sample, assuming normally distributed data. The smaller the p-value, the less trustworthy the prediciton. For highly non-normal X properties (e.g., fingerprints), the MD p-value is wildly inaccurate.

Structural Similar Compounds

Name	N/A	N/A	4651-67-6
Structure	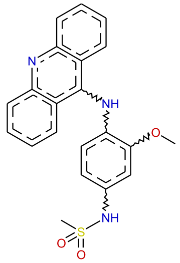	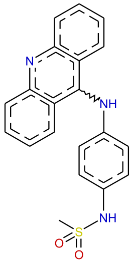	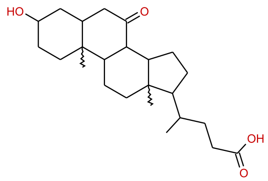
Actual Endpoint	Mutagen	Mutagen	Non-Mutagen
Predicted Endpoint	Mutagen	Mutagen	Non-Mutagen
Distance	0.550	0.562	0.568
Reference	Mut. Res. 280:233-244; 1992	Mut. Res. 280:233-244; 1992	Kazius et. al., J. Med. Chem. (2005) 48, 312-320

Model Applicability

Unknown features are fingerprint features in the query molecule, but not found or appearing too infrequently in the training set.

1. All properties and OPS components are within expected ranges.

Feature Contribution

Top features for positive contribution

Fingerprint	Bit/Smiles	Feature Structure	Score	Mutagen in training set
SCFP_12	555539852	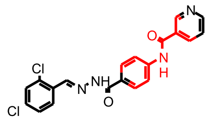 [*]:[cH]:[c](:[cH]:[*])C(=O)N[c]1:[cH]:[c H]:[*]:[cH]:[cH]:1	0.447	22 out of 24

SCFP_12	818445224	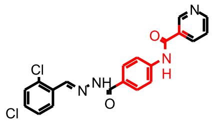 [*][c]1:[cH]:[cH]:[c](NC(=O)[c](:[*]):[*]):[cH]:[cH]:1	0.434	12 out of 13
SCFP_12	124026986	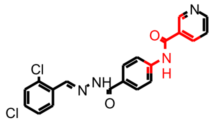 [*]:[cH]:[c](:[cH]:[*])C(=O)N[c](:[*]):[*]	0.429	33 out of 37
**Top Features for negative contribution**				
**Fingerprint**	**Bit/Smiles**	**Feature Structure**	**Score**	**Mutagen in training set**
SCFP_12	1809645840	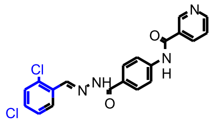 [*][c]1:[*]:[cH]:[c](Cl):[cH]:[c]:1Cl	-1.33	12 out of 84
SCFP_12	-274643719	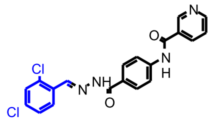 [*]=C[c]1:[cH]:[cH]:[c](Cl):[cH]:[c]:1Cl	-0.916	1 out of 7
SCFP_12	1908972582	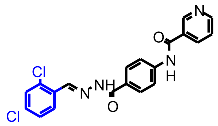 [*][c]1:[cH]:[cH]:[c](Cl):[cH]:[c]:1Cl	-0.821	10 out of 42

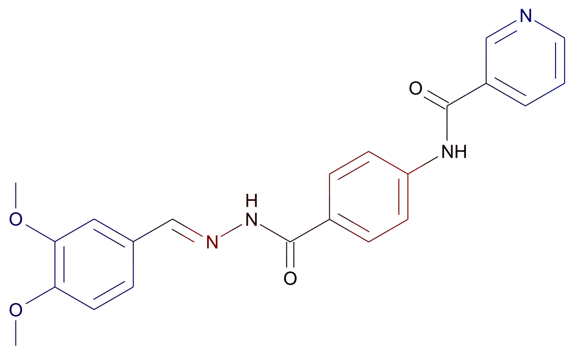

$C_{22}H_{20}N_4O_4$
Molecular Weight: 404.4186
ALogP: 2.284
Rotatable Bonds: 7
Acceptors: 6
Donors: 2

Structural Similar Compounds

Name	HYCANTHONE N-OXIDE	Delavirdine	458-37-7
Structure	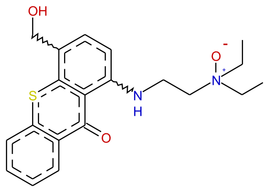	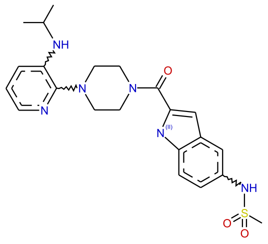	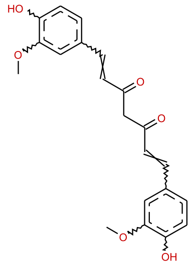
Actual Endpoint	Mutagen	Non-Mutagen	Non-Mutagen
Predicted Endpoint	Mutagen	Non-Mutagen	Non-Mutagen
Distance	0.564	0.573	0.578
Reference	EMIC	Contrera, J.F., Matthews, E.J., Kruhlak, N.L., and Benz, R.D., Regulatory Toxicology and Pharmacology 2005, 313-323.	Kazius et. al., J. Med. Chem. (2005) 48, 312-320

Model Prediction

Prediction: Non-Mutagen
Probability: 0.714
Enrichment: 1.28
Bayesian Score: -1.19
Mahalanobis Distance: 7.64
Mahalanobis Distance p-value: 0.998

Prediction: Positive if the Bayesian score is above the estimated best cutoff value from minimizing the false positive and false negative rate.
Probability: The esimated probability that the sample is in the positive category. This assumes that the Bayesian score follows a normal distribution and is different from the prediction using a cutoff.
Enrichment: An estimate of enrichment, that is, the increased likelihood (versus random) of this sample being in the category.
Bayesian Score: The standard Laplacian-modified Bayesian score.
Mahalanobis Distance: The Mahalanobis distance (MD) is the distance to the center of the training data. The larger the MD, the less trustworthy the prediction.
Mahalanobis Distance p-value: The p-value gives the fraction of training data with an MD greater than or equal to the one for the given sample, assuming normally distributed data. The smaller the p-value, the less trustworthy the prediciton. For highly non-normal X properties (e.g., fingerprints), the MD p-value is wildly inaccurate.

Model Applicability

Unknown features are fingerprint features in the query molecule, but not found or appearing too infrequently in the training set.

1. All properties and OPS components are within expected ranges.

Feature Contribution

Top features for positive contribution

Fingerprint	Bit/Smiles	Feature Structure	Score	Mutagen in training set
SCFP_12	555539852	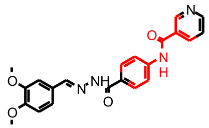 [*]:[cH]:[c](:[cH]:[*])C(=O)N[c]1:[cH]:[c H]:[*]:[cH]:[cH]:1	0.447	22 out of 24

SCFP_12	2037424534	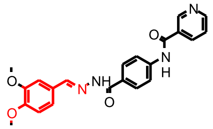<br>[*]O[c]1:[cH]:[cH]:[c<br>](\C=N\[*]):[cH]:[c]<br>:1[*]	0.442	13 out of 14
SCFP_12	818445224	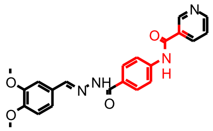<br>[*][c]1:[cH]:[cH]:[c]<br>(NC(=O)[c](:[*]):[*]<br>):[cH]:[cH]:1	0.434	12 out of 13

Top Features for negative contribution

Fingerprint	Bit/Smiles	Feature Structure	Score	Mutagen in training set
SCFP_12	1538617933	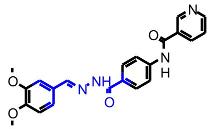<br>[*][c](:[*]):[c](\C=N<br>\NC(=O)[c](:[*]):[*]<br>):[cH]:[*]	-0.762	0 out of 2
SCFP_12	1165971455	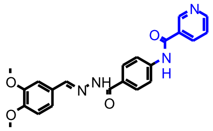<br>[*]NC(=O)[c]1:[cH]:[c<br>H]:[cH]:n:[cH]:1	-0.762	0 out of 2
SCFP_12	903335088	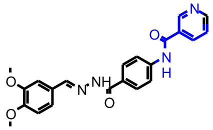<br>[*]NC(=O)[c]1:[cH]:[c<br>H]:[*]:n:[cH]:1	-0.762	0 out of 2

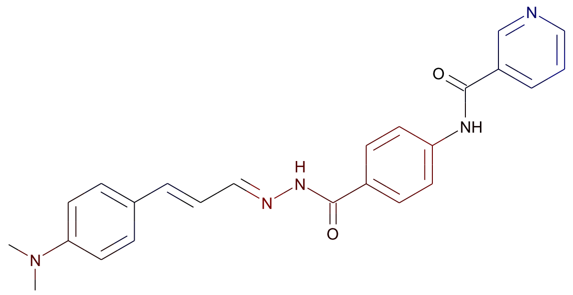

$C_{24}H_{23}N_5O_2$
Molecular Weight: 413.47172
ALogP: 2.839
Rotatable Bonds: 7
Acceptors: 5
Donors: 2

Model Prediction

Prediction: Mutagen
Probability: 0.765
Enrichment: 1.37
Bayesian Score: 1.46
Mahalanobis Distance: 9.67
Mahalanobis Distance p-value: 0.493

Prediction: Positive if the Bayesian score is above the estimated best cutoff value from minimizing the false positive and false negative rate.
Probability: The esimated probability that the sample is in the positive category. This assumes that the Bayesian score follows a normal distribution and is different from the prediction using a cutoff.
Enrichment: An estimate of enrichment, that is, the increased likelihood (versus random) of this sample being in the category.
Bayesian Score: The standard Laplacian-modified Bayesian score.
Mahalanobis Distance: The Mahalanobis distance (MD) is the distance to the center of the training data. The larger the MD, the less trustworthy the prediction.
Mahalanobis Distance p-value: The p-value gives the fraction of training data with an MD greater than or equal to the one for the given sample, assuming normally distributed data. The smaller the p-value, the less trustworthy the prediciton. For highly non-normal X properties (e.g., fingerprints), the MD p-value is wildly inaccurate.

Structural Similar Compounds

Name	HYCANTHONE N-OXIDE	54484-90-1	HYCANTHONE
Structure	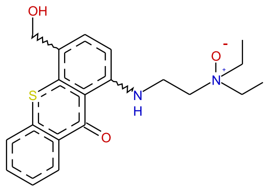	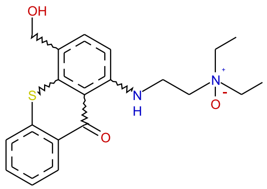	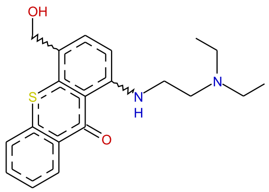
Actual Endpoint	Mutagen	Mutagen	Mutagen
Predicted Endpoint	Mutagen	Mutagen	Mutagen
Distance	0.541	0.546	0.572
Reference	EMIC	Kazius et. al., J. Med. Chem. (2005) 48, 312-320	EMIC

Model Applicability

Unknown features are fingerprint features in the query molecule, but not found or appearing too infrequently in the training set.

1. All properties and OPS components are within expected ranges.

Feature Contribution

Top features for positive contribution				
**Fingerprint**	**Bit/Smiles**	**Feature Structure**	**Score**	**Mutagen in training set**
SCFP_12	2016450626	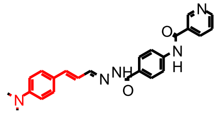 [*]\C=C\[c]1:[cH]:[cH]:[c](:[cH]:[cH]:1)N([*])[*]	0.474	41 out of 44

SCFP_12	555539852	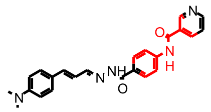 [*]:[cH]:[c](:[cH]:[*])C(=O)N[c]1:[cH]:[cH]:[*]:[cH]:[cH]:1	0.447	22 out of 24
SCFP_12	818445224	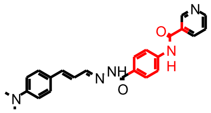 [*][c]1:[cH]:[cH]:[c](NC(=O)[c](:[*]):[*]):[cH]:[cH]:1	0.434	12 out of 13

Top Features for negative contribution

Fingerprint	Bit/Smiles	Feature Structure	Score	Mutagen in training set
SCFP_12	903335088	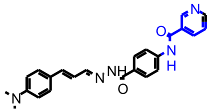 [*]NC(=O)[c]1:[cH]:[cH]:[*]:n:[cH]:1	-0.762	0 out of 2
SCFP_12	1165971455	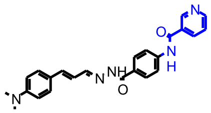 [*]NC(=O)[c]1:[cH]:[cH]:[cH]:n:[cH]:1	-0.762	0 out of 2
SCFP_12	-105808146	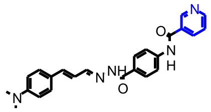 [*][c]1:[cH]:[cH]:[cH]:n:[cH]:1	-0.411	8 out of 22

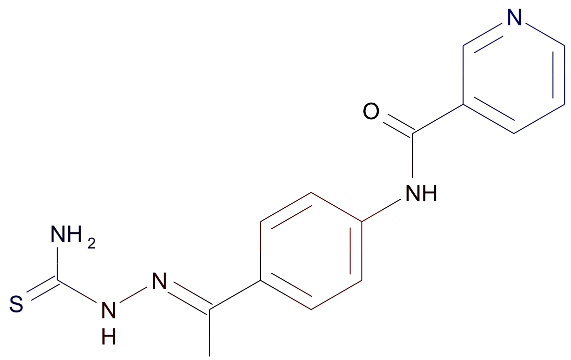

$C_{15}H_{15}N_5OS$
Molecular Weight: 313.3775
ALogP: 1.414
Rotatable Bonds: 5
Acceptors: 4
Donors: 3

Model Prediction

Prediction: Non-Mutagen
Probability: 0.7
Enrichment: 1.25
Bayesian Score: -1.77
Mahalanobis Distance: 13.8
Mahalanobis Distance p-value: 1.99e-008

Prediction: Positive if the Bayesian score is above the estimated best cutoff value from minimizing the false positive and false negative rate.
Probability: The esimated probability that the sample is in the positive category. This assumes that the Bayesian score follows a normal distribution and is different from the prediction using a cutoff.
Enrichment: An estimate of enrichment, that is, the increased likelihood (versus random) of this sample being in the category.
Bayesian Score: The standard Laplacian-modified Bayesian score.
Mahalanobis Distance: The Mahalanobis distance (MD) is the distance to the center of the training data. The larger the MD, the less trustworthy the prediction.
Mahalanobis Distance p-value: The p-value gives the fraction of training data with an MD greater than or equal to the one for the given sample, assuming normally distributed data. The smaller the p-value, the less trustworthy the prediciton. For highly non-normal X properties (e.g., fingerprints), the MD p-value is wildly inaccurate.

Structural Similar Compounds

Name	Furosemide	CHLORAMPHENICOL	134-90-7
Structure	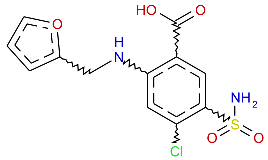	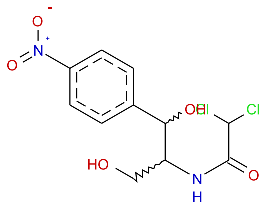	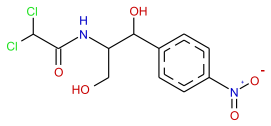
Actual Endpoint	Non-Mutagen	Non-Mutagen	Mutagen
Predicted Endpoint	Non-Mutagen	Non-Mutagen	Non-Mutagen
Distance	0.557	0.557	0.557
Reference	Environ. Mol. Mut. 16(Suppl 18):1-14;1990	EMIC	Kazius et. al., J. Med. Chem. (2005) 48, 312-320

Model Applicability

Unknown features are fingerprint features in the query molecule, but not found or appearing too infrequently in the training set.

1. All properties and OPS components are within expected ranges.

Feature Contribution

Top features for positive contribution				
**Fingerprint**	**Bit/Smiles**	**Feature Structure**	**Score**	**Mutagen in training set**
SCFP_12	555539852	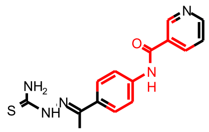 [*]:[cH]:[c](:[cH]:[*])C(=O)N[c]1:[cH]:[cH]:[*]:[cH]:[cH]:1	0.447	22 out of 24

SCFP_12	827404948	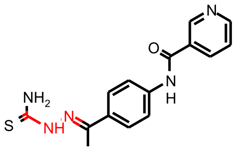<br>[*]C(=[*])NN=[*]	0.442	5 out of 5
SCFP_12	818445224	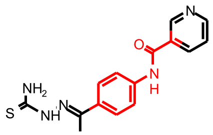<br>[*][c]1:[cH]:[cH]:[c]<br>(NC(=O)[c](:[*]):[*]<br>):[cH]:[cH]:1	0.434	12 out of 13
**Top Features for negative contribution**				
**Fingerprint**	**Bit/Smiles**	**Feature Structure**	**Score**	**Mutagen in training set**
SCFP_12	382734644	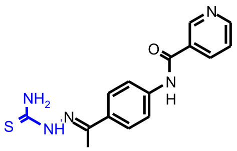<br>[*]NC(=S)N	-0.811	3 out of 14
SCFP_12	1165971455	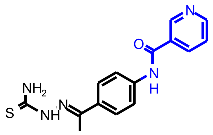<br>[*]NC(=O)[c]1:[cH]:[c<br>H]:[cH]:n:[cH]:1	-0.762	0 out of 2
SCFP_12	-331724199	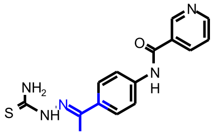<br>[*]\N=C(/C)\[c](:[*])<br>:[*]	-0.762	0 out of 2

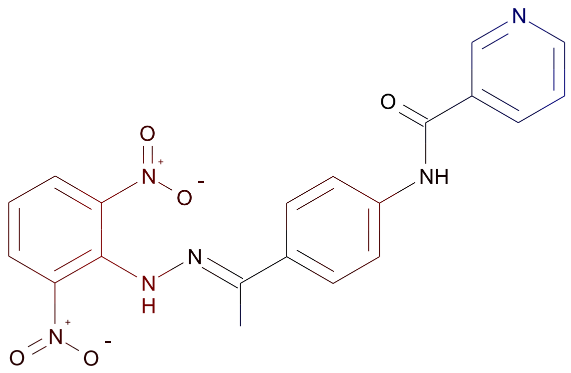

$C_{20}H_{16}N_6O_5$
Molecular Weight: 420.37824
ALogP: 2.852
Rotatable Bonds: 7
Acceptors: 8
Donors: 2

Model Prediction

Prediction: Mutagen
Probability: 0.782
Enrichment: 1.4
Bayesian Score: 2.71
Mahalanobis Distance: 8.16
Mahalanobis Distance p-value: 0.985

Prediction: Positive if the Bayesian score is above the estimated best cutoff value from minimizing the false positive and false negative rate.
Probability: The esimated probability that the sample is in the positive category. This assumes that the Bayesian score follows a normal distribution and is different from the prediction using a cutoff.
Enrichment: An estimate of enrichment, that is, the increased likelihood (versus random) of this sample being in the category.
Bayesian Score: The standard Laplacian-modified Bayesian score.
Mahalanobis Distance: The Mahalanobis distance (MD) is the distance to the center of the training data. The larger the MD, the less trustworthy the prediction.
Mahalanobis Distance p-value: The p-value gives the fraction of training data with an MD greater than or equal to the one for the given sample, assuming normally distributed data. The smaller the p-value, the less trustworthy the prediciton. For highly non-normal X properties (e.g., fingerprints), the MD p-value is wildly inaccurate.

Structural Similar Compounds

Name	33372-39-3	6358-31-2	C.I. Pigment Yellow 74
Structure	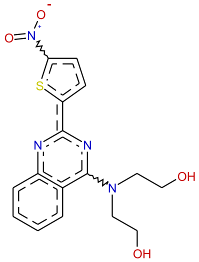	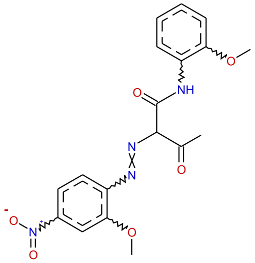	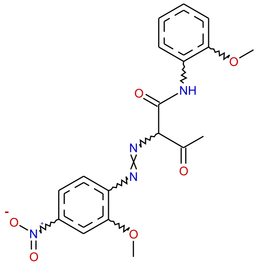
Actual Endpoint	Mutagen	Non-Mutagen	Non-Mutagen
Predicted Endpoint	Mutagen	Non-Mutagen	Non-Mutagen
Distance	0.573	0.575	0.585
Reference	Kazius et. al., J. Med. Chem. (2005) 48, 312-320	Kazius et. al., J. Med. Chem. (2005) 48, 312-320	EMIC

Model Applicability

Unknown features are fingerprint features in the query molecule, but not found or appearing too infrequently in the training set.

1. All properties and OPS components are within expected ranges.

Feature Contribution

Top features for positive contribution

Fingerprint	Bit/Smiles	Feature Structure	Score	Mutagen in training set
SCFP_12	-990000384	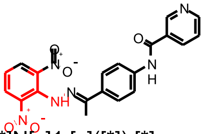 [*]N[c]1:[c]([*]):[*]:[cH]:[cH]:[c]:1[N+](=O)[O-]	0.507	50 out of 52

SCFP_12	555539852	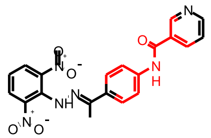 [*]:[cH]:[c](:[cH]:[*])C(=O)N[c]1:[cH]:[c H]:[*]:[cH]:[cH]:1	0.447	22 out of 24
SCFP_12	818445224	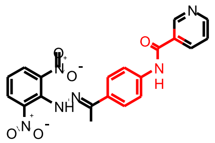 [*][c]1:[cH]:[cH]:[c] (NC(=O)[c](:[*]):[*]):[cH]:[cH]:1	0.434	12 out of 13
**Top Features for negative contribution**				
**Fingerprint**	**Bit/Smiles**	**Feature Structure**	**Score**	**Mutagen in training set**
SCFP_12	1165971455	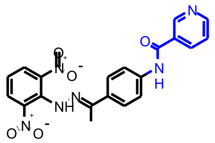 [*]NC(=O)[c]1:[cH]:[c H]:[cH]:n:[cH]:1	-0.762	0 out of 2
SCFP_12	-331724199	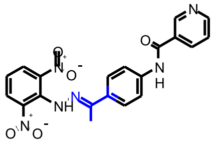 [*]\N=C(/C)\[c](:[*]) :[*]	-0.762	0 out of 2
SCFP_12	903335088	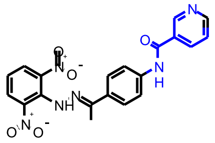 [*]NC(=O)[c]1:[cH]:[c H]:[*]:n:[cH]:1	-0.762	0 out of 2

Sorafenib

TOPKAT_Ames_Mutagenicity

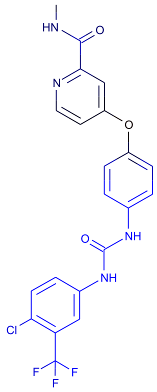

$C_{21}H_{16}ClF_3N_4O_3$

Molecular Weight: 464.82494

ALogP: 4.175

Rotatable Bonds: 6

Acceptors: 4

Donors: 3

Structural Similar Compounds

Name	GLYBURIDE	38914-96-4	93957-54-1
Structure	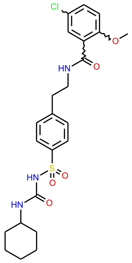	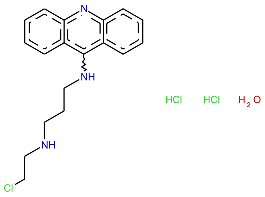	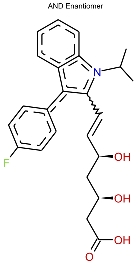
Actual Endpoint	Non-Mutagen	Mutagen	Non-Mutagen
Predicted Endpoint	Non-Mutagen	Mutagen	Non-Mutagen
Distance	0.590	0.592	0.600
Reference	PDR 1994	Kazius et. al., J. Med. Chem. (2005) 48, 312-320	US Environmental Protection Agency at http://www.epa.gov/NCCT/dsstox/sdf_isscan_external.html

Model Prediction

Prediction: Non-Mutagen

Probability: 0.0531

Enrichment: 0.0951

Bayesian Score: -19.7

Mahalanobis Distance: 13.1

Mahalanobis Distance p-value: 2.73e-006

Prediction: Positive if the Bayesian score is above the estimated best cutoff value from minimizing the false positive and false negative rate.
Probability: The esimated probability that the sample is in the positive category. This assumes that the Bayesian score follows a normal distribution and is different from the prediction using a cutoff.
Enrichment: An estimate of enrichment, that is, the increased likelihood (versus random) of this sample being in the category.
Bayesian Score: The standard Laplacian-modified Bayesian score.
Mahalanobis Distance: The Mahalanobis distance (MD) is the distance to the center of the training data. The larger the MD, the less trustworthy the prediction.
Mahalanobis Distance p-value: The p-value gives the fraction of training data with an MD greater than or equal to the one for the given sample, assuming normally distributed data. The smaller the p-value, the less trustworthy the prediciton. For highly non-normal X properties (e.g., fingerprints), the MD p-value is wildly inaccurate.

Model Applicability

Unknown features are fingerprint features in the query molecule, but not found or appearing too infrequently in the training set.

1. All properties and OPS components are within expected ranges.

Feature Contribution

Top features for positive contribution				
**Fingerprint**	**Bit/Smiles**	**Feature Structure**	**Score**	**Mutagen in training set**
SCFP_12	-347281112	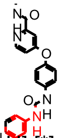 [*]N[c]1:[cH]:[*]:[c]([*]):[c](:[cH]:1)C([*])([*])[*]	0.337	18 out of 22

SCFP_12	1208843554	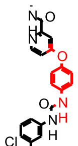 [*]N[c]1:[cH]:[cH]:[c](O[c](:[*]):[*]):[cH]:[cH]:1	0.337	6 out of 7
SCFP_12	-1943080297	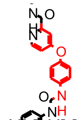 [*]N[c]1:[cH]:[cH]:[c](O[c]2:[cH]:[cH]:[*]:[c]([*]):[cH]:2):[cH]:[cH]:1	0.304	5 out of 6

Top Features for negative contribution

Fingerprint	Bit/Smiles	Feature Structure	Score	Mutagen in training set
SCFP_12	816802409	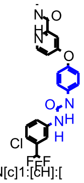 [*]NC(=O)N[c]1:[cH]:[cH]:[c]([*]):[cH]:[cH]:1	-1.82	0 out of 9
SCFP_12	-1903175541	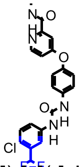 [*][c](:[*]):[c](:[cH]:[*])C(F)(F)F	-1.51	3 out of 30
SCFP_12	-300280774	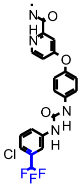 [*]:[c](:[*])C(F)(F)F	-1.51	3 out of 30

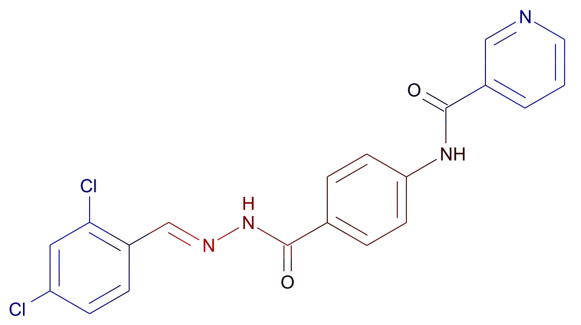

$C_{20}H_{14}Cl_2N_4O_2$

Molecular Weight: 413.25676

ALogP: 3.646

Rotatable Bonds: 5

Acceptors: 4

Donors: 2

Structural Similar Compounds

Name	Bicalutamide	Indomethacin	Indapamide
Structure	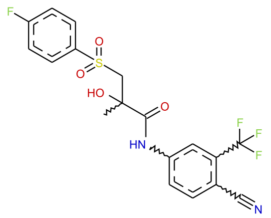	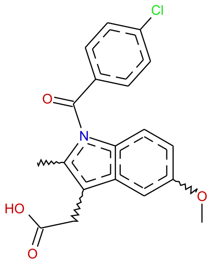	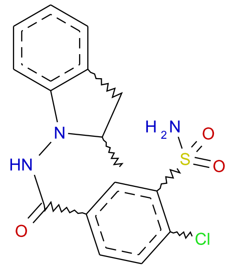
Actual Endpoint	Non-Carcinogen	Non-Carcinogen	Non-Carcinogen
Predicted Endpoint	Non-Carcinogen	Non-Carcinogen	Non-Carcinogen
Distance	0.610	0.634	0.642
Reference	US FDA (Centre for Drug Eval.& Res./Off. Testing & Res.) Sept. 1997	US FDA (Centre for Drug Eval.& Res./Off. Testing & Res.) Sept. 1997	US FDA (Centre for Drug Eval.& Res./Off. Testing & Res.) Sept. 1997

Model Prediction

Prediction: Non-Carcinogen

Probability: 0.221

Enrichment: 0.691

Bayesian Score: -2.83

Mahalanobis Distance: 13.2

Mahalanobis Distance p-value: 0.000234

Prediction: Positive if the Bayesian score is above the estimated best cutoff value from minimizing the false positive and false negative rate.
Probability: The esimated probability that the sample is in the positive category. This assumes that the Bayesian score follows a normal distribution and is different from the prediction using a cutoff.
Enrichment: An estimate of enrichment, that is, the increased likelihood (versus random) of this sample being in the category.
Bayesian Score: The standard Laplacian-modified Bayesian score.
Mahalanobis Distance: The Mahalanobis distance (MD) is the distance to the center of the training data. The larger the MD, the less trustworthy the prediction.
Mahalanobis Distance p-value: The p-value gives the fraction of training data with an MD greater than or equal to the one for the given sample, assuming normally distributed data. The smaller the p-value, the less trustworthy the prediciton. For highly non-normal X properties (e.g., fingerprints), the MD p-value is wildly inaccurate.

Model Applicability

Unknown features are fingerprint features in the query molecule, but not found or appearing too infrequently in the training set.

1. All properties and OPS components are within expected ranges.
2. Unknown ECFP_2 feature: 1335702447: [*][c](:[*]):[c](C=[*]):c:[*]

Feature Contribution

Top features for positive contribution				
Fingerprint	Bit/Smiles	Feature Structure	Score	Carcinogen in training set
ECFP_6	-1832102709	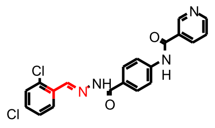 [*]\N=C\[c](:[*]):[*]	0.742	5 out of 6

ECFP_6	-1087070950	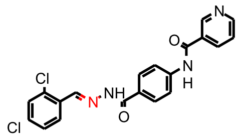 [*]N=[*]	0.724	10 out of 14
ECFP_6	738938915	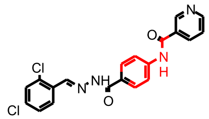 [*]C(=[*])N[c]1:[cH]:[cH]:[*]:[cH]:[cH]:1	0.617	2 out of 2
**Top Features for negative contribution**				
**Fingerprint**	**Bit/Smiles**	**Feature Structure**	**Score**	**Carcinogen in training set**
ECFP_6	2013347047	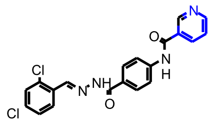 [*][c]1:[*]:n:[cH]:[cH]:[cH]:1	-0.805	0 out of 4
ECFP_6	1335691903	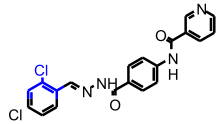 [*][c](:[*]):[c](Cl):[cH]:[*]	-0.669	3 out of 22
ECFP_6	577592657	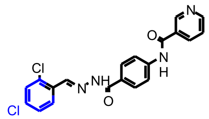 [*][c]1:[*]:[cH]:[cH]:[c](Cl):[cH]:1	-0.586	3 out of 20

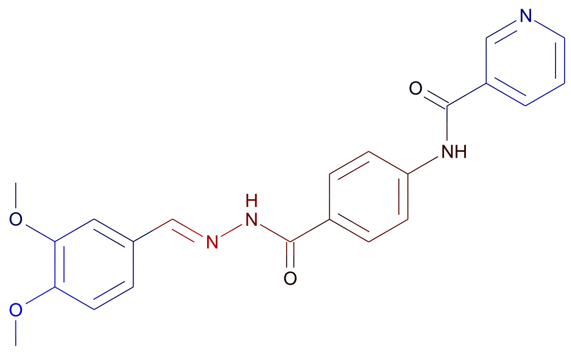

$C_{22}H_{20}N_4O_4$
Molecular Weight: 404.4186
ALogP: 2.284
Rotatable Bonds: 7
Acceptors: 6
Donors: 2

Model Prediction

Prediction: Non-Carcinogen
Probability: 0.223
Enrichment: 0.695
Bayesian Score: -2.68
Mahalanobis Distance: 12
Mahalanobis Distance p-value: 0.00953

Prediction: Positive if the Bayesian score is above the estimated best cutoff value from minimizing the false positive and false negative rate.
Probability: The esimated probability that the sample is in the positive category. This assumes that the Bayesian score follows a normal distribution and is different from the prediction using a cutoff.
Enrichment: An estimate of enrichment, that is, the increased likelihood (versus random) of this sample being in the category.
Bayesian Score: The standard Laplacian-modified Bayesian score.
Mahalanobis Distance: The Mahalanobis distance (MD) is the distance to the center of the training data. The larger the MD, the less trustworthy the prediction.
Mahalanobis Distance p-value: The p-value gives the fraction of training data with an MD greater than or equal to the one for the given sample, assuming normally distributed data. The smaller the p-value, the less trustworthy the prediciton. For highly non-normal X properties (e.g., fingerprints), the MD p-value is wildly inaccurate.

Structural Similar Compounds

Name	Bicalutamide	Moricizine	Alizapride
Structure	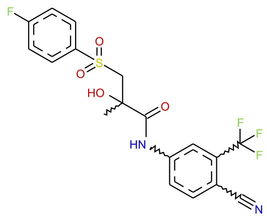	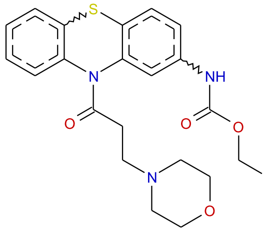	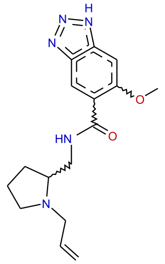
Actual Endpoint	Non-Carcinogen	Carcinogen	Carcinogen
Predicted Endpoint	Non-Carcinogen	Carcinogen	Carcinogen
Distance	0.589	0.624	0.627
Reference	US FDA (Centre for Drug Eval.& Res./Off. Testing & Res.) Sept. 1997	US FDA (Centre for Drug Eval.& Res./Off. Testing & Res.) Sept. 1997	US FDA (Centre for Drug Eval.& Res./Off. Testing & Res.) Sept. 1997

Model Applicability

Unknown features are fingerprint features in the query molecule, but not found or appearing too infrequently in the training set.

1. All properties and OPS components are within expected ranges.
2. Unknown ECFP_2 feature: -176483725: [*]=C[c](:c:[*]):c:[*]

Feature Contribution

Top features for positive contribution				
Fingerprint	Bit/Smiles	Feature Structure	Score	Carcinogen in training set
ECFP_6	-1832102709	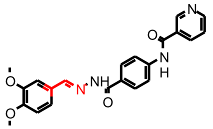 [*]\N=C\[c](:[*]):[*]	0.742	5 out of 6

ECFP_6	-1087070950	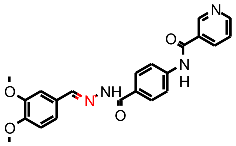 [*]N=[*]	0.724	10 out of 14
ECFP_6	544048674	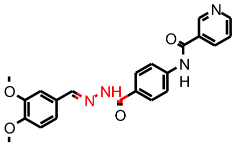 [*]C(=[*])NN=[*]	0.617	2 out of 2

Top Features for negative contribution

Fingerprint	Bit/Smiles	Feature Structure	Score	Carcinogen in training set
ECFP_6	2077607946	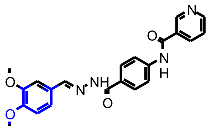 [*]O[c]1:[cH]:[cH]:[c](([*]):[*]:[c]:1[*]	-1.15	0 out of 7
ECFP_6	-468366781	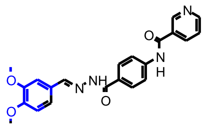 [*]O[c]1:[cH]:[*]:[c]([*]):[cH]:[c]:1OC	-0.805	0 out of 4
ECFP_6	2013347047	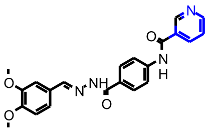 [*][c]1:[*]:n:[cH]:[cH]:[cH]:1	-0.805	0 out of 4

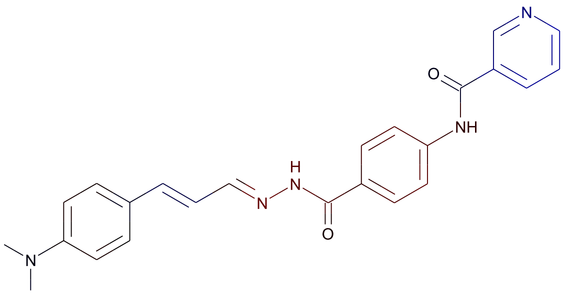

$C_{24}H_{23}N_5O_2$
Molecular Weight: 413.47172
ALogP: 2.839
Rotatable Bonds: 7
Acceptors: 5
Donors: 2

Model Prediction

Prediction: Non-Carcinogen
Probability: 0.245
Enrichment: 0.765
Bayesian Score: -0.99
Mahalanobis Distance: 12.3
Mahalanobis Distance p-value: 0.00439

Prediction: Positive if the Bayesian score is above the estimated best cutoff value from minimizing the false positive and false negative rate.
Probability: The esimated probability that the sample is in the positive category. This assumes that the Bayesian score follows a normal distribution and is different from the prediction using a cutoff.
Enrichment: An estimate of enrichment, that is, the increased likelihood (versus random) of this sample being in the category.
Bayesian Score: The standard Laplacian-modified Bayesian score.
Mahalanobis Distance: The Mahalanobis distance (MD) is the distance to the center of the training data. The larger the MD, the less trustworthy the prediction.
Mahalanobis Distance p-value: The p-value gives the fraction of training data with an MD greater than or equal to the one for the given sample, assuming normally distributed data. The smaller the p-value, the less trustworthy the prediciton. For highly non-normal X properties (e.g., fingerprints), the MD p-value is wildly inaccurate.

Structural Similar Compounds

Name	Bicalutamide	Moricizine	Flecainide
Structure	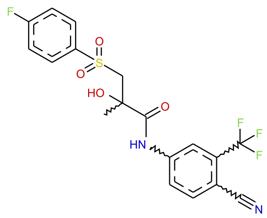	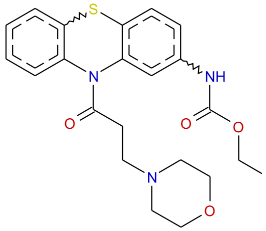	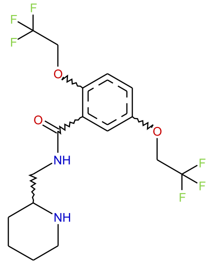
Actual Endpoint	Non-Carcinogen	Carcinogen	Non-Carcinogen
Predicted Endpoint	Non-Carcinogen	Carcinogen	Non-Carcinogen
Distance	0.587	0.638	0.642
Reference	US FDA (Centre for Drug Eval.& Res./Off. Testing & Res.) Sept. 1997	US FDA (Centre for Drug Eval.& Res./Off. Testing & Res.) Sept. 1997	US FDA (Centre for Drug Eval.& Res./Off. Testing & Res.) Sept. 1997

Model Applicability

Unknown features are fingerprint features in the query molecule, but not found or appearing too infrequently in the training set.

1. All properties and OPS components are within expected ranges.
2. Unknown ECFP_2 feature: -984423246: [*]\N=C\C=[*]
3. Unknown ECFP_2 feature: -176483725: [*]=C[c](:c:[*]):c:[*]

Feature Contribution

Top features for positive contribution

Fingerprint	Bit/Smiles	Feature Structure	Score	Carcinogen in training set
ECFP_6	-1087070950	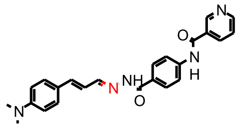 [*]N=[*]	0.724	10 out of 14

ECFP_6	1814278164	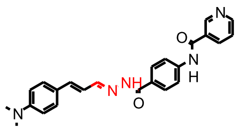 [*]N\N=C\[*]	0.617	2 out of 2
ECFP_6	738938915	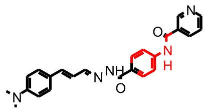 [*]C(=[*])N[c]1:[cH]:[cH]:[*]:[cH]:[cH]:1	0.617	2 out of 2

Top Features for negative contribution

Fingerprint	Bit/Smiles	Feature Structure	Score	Carcinogen in training set
ECFP_6	-1831055759	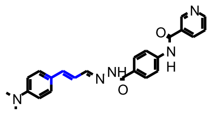 [*]\C=C\[c](:[*]):[*]	-0.805	0 out of 4
ECFP_6	2013347047	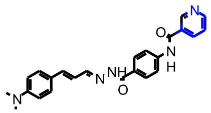 [*][c]1:[*]:n:[cH]:[cH]:[cH]:1	-0.805	0 out of 4
ECFP_6	-471753896	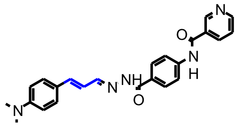 [*]\C=C\C=[*]	-0.482	0 out of 2

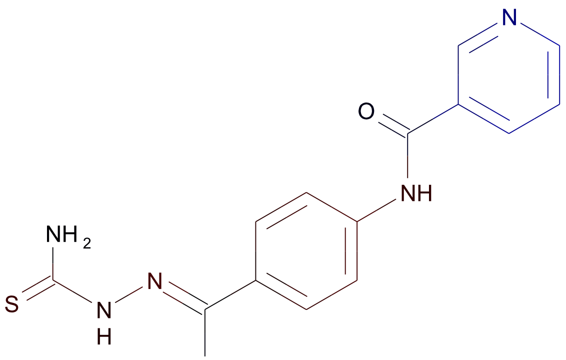

$C_{15}H_{15}N_5OS$
Molecular Weight: 313.3775
ALogP: 1.414
Rotatable Bonds: 5
Acceptors: 4
Donors: 3

Model Prediction

Prediction: Carcinogen
Probability: 0.248
Enrichment: 0.774
Bayesian Score: -0.815
Mahalanobis Distance: 12.4
Mahalanobis Distance p-value: 0.00324

Prediction: Positive if the Bayesian score is above the estimated best cutoff value from minimizing the false positive and false negative rate.
Probability: The esimated probability that the sample is in the positive category. This assumes that the Bayesian score follows a normal distribution and is different from the prediction using a cutoff.
Enrichment: An estimate of enrichment, that is, the increased likelihood (versus random) of this sample being in the category.
Bayesian Score: The standard Laplacian-modified Bayesian score.
Mahalanobis Distance: The Mahalanobis distance (MD) is the distance to the center of the training data. The larger the MD, the less trustworthy the prediction.
Mahalanobis Distance p-value: The p-value gives the fraction of training data with an MD greater than or equal to the one for the given sample, assuming normally distributed data. The smaller the p-value, the less trustworthy the prediciton. For highly non-normal X properties (e.g., fingerprints), the MD p-value is wildly inaccurate.

Structural Similar Compounds

Name	Furosemide	Tolbutamide	Sotalol
Structure	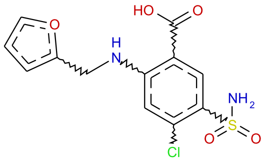	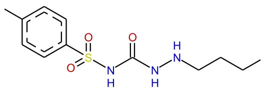	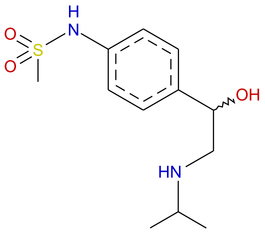
Actual Endpoint	Carcinogen	Non-Carcinogen	Non-Carcinogen
Predicted Endpoint	Carcinogen	Non-Carcinogen	Non-Carcinogen
Distance	0.568	0.573	0.621
Reference	US FDA (Centre for Drug Eval.& Res./Off. Testing & Res.) Sept. 1997	US FDA (Centre for Drug Eval.& Res./Off. Testing & Res.) Sept. 1997	US FDA (Centre for Drug Eval.& Res./Off. Testing & Res.) Sept. 1997

Model Applicability

Unknown features are fingerprint features in the query molecule, but not found or appearing too infrequently in the training set.

1. All properties and OPS components are within expected ranges.
2. Unknown ECFP_2 feature: 128986386: [*]\N=C(/C)\[c](:[*]):[*]
3. Unknown ECFP_2 feature: 560380707: [*]NN=C([*])[*]
4. Unknown ECFP_2 feature: -571028867: [*]NC(=S)N

Feature Contribution

Top features for positive contribution

Fingerprint	Bit/Smiles	Feature Structure	Score	Carcinogen in training set
ECFP_6	-1087070950	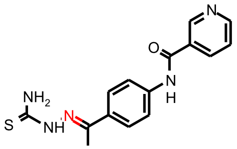 [*]N=[*]	0.724	10 out of 14

ECFP_6	-845108448	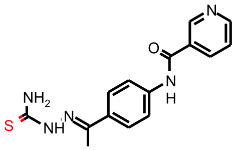 [*]=S	0.675	4 out of 5
ECFP_6	738938915	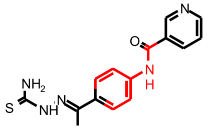 [*]C(=[*])N[c]1:[cH]:[cH]:[*]:[cH]:[cH]:1	0.617	2 out of 2

Top Features for negative contribution

Fingerprint	Bit/Smiles	Feature Structure	Score	Carcinogen in training set
ECFP_6	2013347047	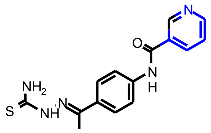 [*][c]1:[*]:n:[cH]:[cH]:[cH]:1	-0.805	0 out of 4
ECFP_6	764951226	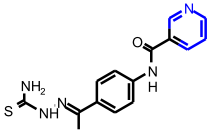 [*]1:[cH]:[cH]:[cH]:n:[cH]:1	-0.482	0 out of 2
ECFP_6	-1818873508	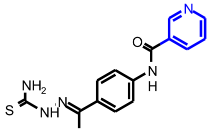 [*][c]1:[cH]:[cH]:[cH]:n:[cH]:1	-0.482	0 out of 2

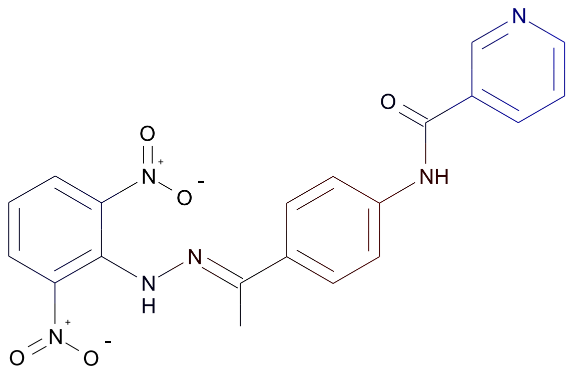

$C_{20}H_{16}N_6O_5$

Molecular Weight: 420.37824

ALogP: 2.852

Rotatable Bonds: 7

Acceptors: 8

Donors: 2

Model Prediction

Prediction: Non-Carcinogen

Probability: 0.217

Enrichment: 0.676

Bayesian Score: -3.36

Mahalanobis Distance: 12

Mahalanobis Distance p-value: 0.011

Prediction: Positive if the Bayesian score is above the estimated best cutoff value from minimizing the false positive and false negative rate.
Probability: The esimated probability that the sample is in the positive category. This assumes that the Bayesian score follows a normal distribution and is different from the prediction using a cutoff.
Enrichment: An estimate of enrichment, that is, the increased likelihood (versus random) of this sample being in the category.
Bayesian Score: The standard Laplacian-modified Bayesian score.
Mahalanobis Distance: The Mahalanobis distance (MD) is the distance to the center of the training data. The larger the MD, the less trustworthy the prediction.
Mahalanobis Distance p-value: The p-value gives the fraction of training data with an MD greater than or equal to the one for the given sample, assuming normally distributed data. The smaller the p-value, the less trustworthy the prediciton. For highly non-normal X properties (e.g., fingerprints), the MD p-value is wildly inaccurate.

Structural Similar Compounds

Name	Sulfasalazine	Nedocromil	Nisoldipine
Structure	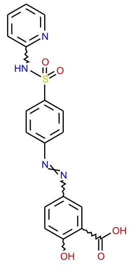	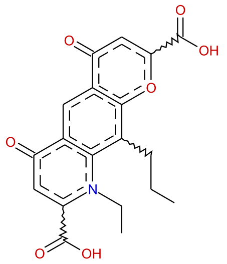	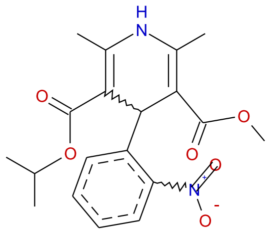
Actual Endpoint	Carcinogen	Non-Carcinogen	Non-Carcinogen
Predicted Endpoint	Carcinogen	Non-Carcinogen	Non-Carcinogen
Distance	0.624	0.635	0.691
Reference	US FDA (Centre for Drug Eval.& Res./Off. Testing & Res.) Sept. 1997	US FDA (Centre for Drug Eval.& Res./Off. Testing & Res.) Sept. 1997	US FDA (Centre for Drug Eval.& Res./Off. Testing & Res.) Sept. 1997

Model Applicability

Unknown features are fingerprint features in the query molecule, but not found or appearing too infrequently in the training set.

1. All properties and OPS components are within expected ranges.
2. Unknown ECFP_2 feature: 1043790491: [*][N+](=[*])[*]
3. Unknown ECFP_2 feature: 781519895: [*][O-]
4. Unknown ECFP_2 feature: 128986386: [*]\N=C(/C)\[c](:[*]):[*]
5. Unknown ECFP_2 feature: 560380707: [*]NN=C([*])[*]
6. Unknown ECFP_2 feature: -1236714312: [*]=NN[c](:[*]):[*]
7. Unknown ECFP_2 feature: -1956535100: [*][c](:[*]):[c]([N+](=[*])[*]):c:[*]
8. Unknown ECFP_2 feature: -215026467: [*]:[c](:[*])[N+](=O)[O-]
9. Unknown ECFP_2 feature: 2104376220: [*][N+](=O)[*]
10. Unknown ECFP_2 feature: -659271057: [*][N+](=[*])[O-]

Feature Contribution

Top features for positive contribution

Fingerprint	Bit/Smiles	Feature Structure	Score	Carcinogen in training set

ECFP_6	-1087070950	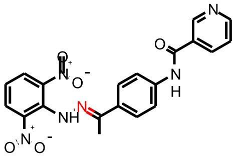 [*]N=[*]	0.724	10 out of 14
ECFP_6	738938915	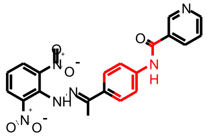 [*]C(=[*])N[c]1:[cH]:[cH]:[*]:[cH]:[cH]:1	0.617	2 out of 2
ECFP_6	-177077903	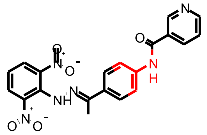 [*]N[c](:[cH]:[*]):[cH]:[*]	0.279	4 out of 9

Top Features for negative contribution

Fingerprint	Bit/Smiles	Feature Structure	Score	Carcinogen in training set
ECFP_6	2013347047	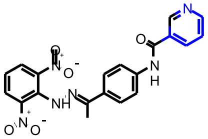 [*][c]1:[*]:n:[cH]:[cH]:[cH]:1	-0.805	0 out of 4
ECFP_6	2007300961	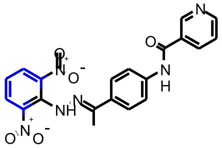 [*][c]1:[*]:[c]([*]):[cH]:[cH]:[cH]:1	-0.652	5 out of 34

ECFP_6	764951226	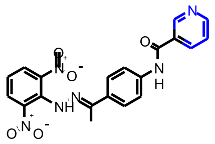 [*]1:[cH]:[cH]:[cH]:n :[cH]:1	-0.482	0 out of 2

Sorafenib

TOPKAT_Mouse_Female_FDA_None_vs_Carcinogen

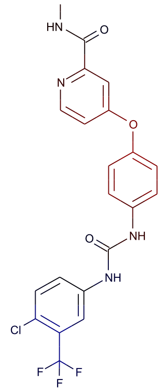

$C_{21}H_{16}ClF_3N_4O_3$
Molecular Weight: 464.82494
ALogP: 4.175
Rotatable Bonds: 6
Acceptors: 4
Donors: 3

Model Prediction

Prediction: Carcinogen
Probability: 0.257
Enrichment: 0.801
Bayesian Score: -0.321
Mahalanobis Distance: 14.9
Mahalanobis Distance p-value: 4.21e-007

Prediction: Positive if the Bayesian score is above the estimated best cutoff value from minimizing the false positive and false negative rate.
Probability: The esimated probability that the sample is in the positive category. This assumes that the Bayesian score follows a normal distribution and is different from the prediction using a cutoff.
Enrichment: An estimate of enrichment, that is, the increased likelihood (versus random) of this sample being in the category.
Bayesian Score: The standard Laplacian-modified Bayesian score.
Mahalanobis Distance: The Mahalanobis distance (MD) is the distance to the center of the training data. The larger the MD, the less trustworthy the prediction.
Mahalanobis Distance p-value: The p-value gives the fraction of training data with an MD greater than or equal to the one for the given sample, assuming normally distributed data. The smaller the p-value, the less trustworthy the prediciton. For highly non-normal X properties (e.g., fingerprints), the MD p-value is wildly inaccurate.

Structural Similar Compounds

Name	Glimepride	Glyburide	Fluvastatin
Structure	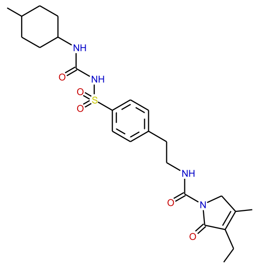	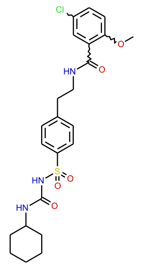	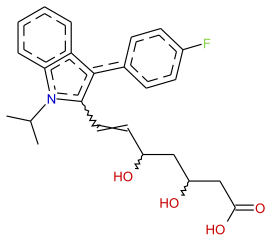
Actual Endpoint	Carcinogen	Non-Carcinogen	Non-Carcinogen
Predicted Endpoint	Carcinogen	Non-Carcinogen	Non-Carcinogen
Distance	0.605	0.615	0.625
Reference	US FDA (Centre for Drug Eval.& Res./Off. Testing & Res.) Sept. 1997	US FDA (Centre for Drug Eval.& Res./Off. Testing & Res.) Sept. 1997	US FDA (Centre for Drug Eval.& Res./Off. Testing & Res.) Sept. 1997

Model Applicability

Unknown features are fingerprint features in the query molecule, but not found or appearing too infrequently in the training set.

1. OPS PC20 out of range. Value: -3.3309. Training min, max, SD, explained variance: -3.1862, 4.4571, 1.28, 0.0167.

Feature Contribution

Top features for positive contribution

Fingerprint	Bit/Smiles	Feature Structure	Score	Carcinogen in training set
ECFP_6	738938915	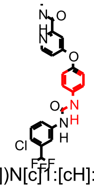 [*]C(=[*])N[c]1:[cH]:[cH]:[*]:[cH]:[cH]:1	0.617	2 out of 2

ECFP_6	1338334141	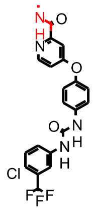<br>[*]C(=[*])NC	0.442	2 out of 3
ECFP_6	-335167981	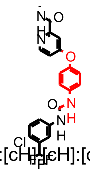<br>[*]O[c]1:[cH]:[cH]:[c](NC(=[*])[*]):[cH]: [cH]:1	0.424	1 out of 1
**Top Features for negative contribution**				
**Fingerprint**	**Bit/Smiles**	**Feature Structure**	**Score**	**Carcinogen in training set**
ECFP_6	1335691903	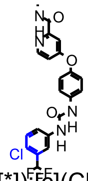<br>[*][c](:[*]):[c](Cl): [cH]:[*]	-0.669	3 out of 22
ECFP_6	1336678434	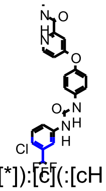<br>[*][c](:[*]):[c](:[cH]:[*])C([*])([*])[*]	-0.657	0 out of 3
ECFP_6	-1952889961	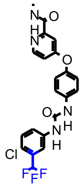<br>[*]:[c](:[*])C(F)(F)F	-0.657	0 out of 3

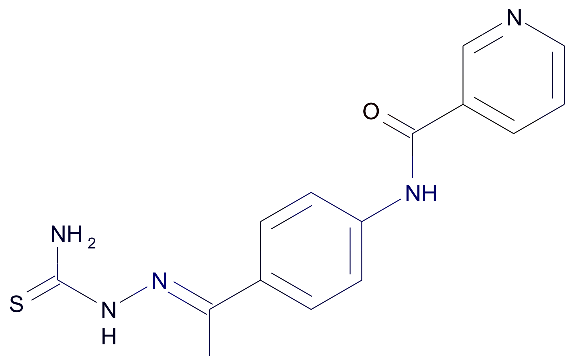

$C_{15}H_{15}N_5OS$

Molecular Weight: 313.3775

ALogP: 1.414

Rotatable Bonds: 5

Acceptors: 4

Donors: 3

Structural Similar Compounds

Name	Furosemide	Sulfamethazine	Guanabenz
Structure	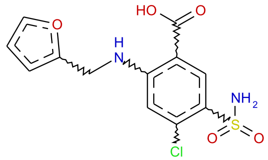	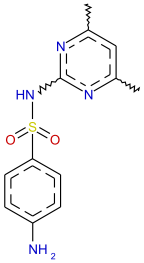	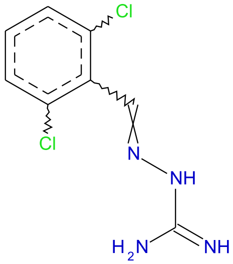
Actual Endpoint	Single-Carcinogen	Single-Carcinogen	Single-Carcinogen
Predicted Endpoint	Single-Carcinogen	Single-Carcinogen	Single-Carcinogen
Distance	0.564	0.683	0.697
Reference	US FDA (Centre for Drug Eval.& Res./Off. Testing & Res.) Sept. 1997	US FDA (Centre for Drug Eval.& Res./Off. Testing & Res.) Sept. 1997	US FDA (Centre for Drug Eval.& Res./Off. Testing & Res.) Sept. 1997

Model Prediction

Prediction: Single-Carcinogen

Probability: 0.221

Enrichment: 0.538

Bayesian Score: -8.6

Mahalanobis Distance: 11.8

Mahalanobis Distance p-value: 0.000561

Prediction: Positive if the Bayesian score is above the estimated best cutoff value from minimizing the false positive and false negative rate.
Probability: The esimated probability that the sample is in the positive category. This assumes that the Bayesian score follows a normal distribution and is different from the prediction using a cutoff.
Enrichment: An estimate of enrichment, that is, the increased likelihood (versus random) of this sample being in the category.
Bayesian Score: The standard Laplacian-modified Bayesian score.
Mahalanobis Distance: The Mahalanobis distance (MD) is the distance to the center of the training data. The larger the MD, the less trustworthy the prediction.
Mahalanobis Distance p-value: The p-value gives the fraction of training data with an MD greater than or equal to the one for the given sample, assuming normally distributed data. The smaller the p-value, the less trustworthy the prediciton. For highly non-normal X properties (e.g., fingerprints), the MD p-value is wildly inaccurate.

Model Applicability

Unknown features are fingerprint features in the query molecule, but not found or appearing too infrequently in the training set.

1. All properties and OPS components are within expected ranges.
2. Unknown ECFP_2 feature: 128986386: [*]\N=C(/C)\[c](:[*]):[*]
3. Unknown ECFP_2 feature: 560380707: [*]NN=C([*])[*]
4. Unknown ECFP_2 feature: -571028867: [*]NC(=S)N

Feature Contribution

Top features for positive contribution				
Fingerprint	Bit/Smiles	Feature Structure	Score	Multiple-Carcinogen in training set
ECFP_4	-175146122	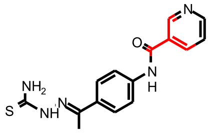 [*]C(=[*])[c](:[cH]:[*]):[cH]:[*]	0.403	6 out of 9

ECFP_4	1430169877	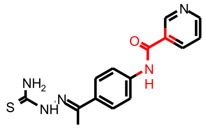<br>[*]NC(=O)[c](:[*]):[*]]	0.299	2 out of 3
ECFP_4	1444581947	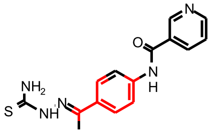<br>[*]C(=[*])[c]1:[cH]:[*]:[c]([*]):[cH]:[cH]:1	0.259	4 out of 7

Top Features for negative contribution

Fingerprint	Bit/Smiles	Feature Structure	Score	Multiple-Carcinogen in training set
ECFP_4	-1087070950	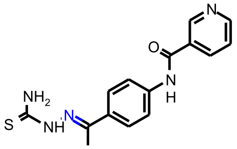<br>[*]N=[*]	-1.63	0 out of 10
ECFP_4	866218936	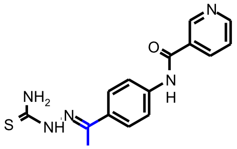<br>[*]C(=[*])C	-1.24	0 out of 6
ECFP_4	888054369	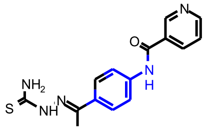<br>[*]N[c]1:[cH]:[*]:[c]([*]):[cH]:[cH]:1	-0.8	0 out of 3

Sorafenib

TOPKAT_Mouse_Female_FDA_Single_vs_Multiple

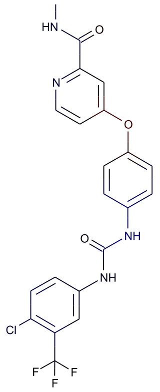

$C_{21}H_{16}ClF_3N_4O_3$

Molecular Weight: 464.82494

ALogP: 4.175

Rotatable Bonds: 6

Acceptors: 4

Donors: 3

Model Prediction

Prediction: Single-Carcinogen

Probability: 0.283

Enrichment: 0.691

Bayesian Score: -3.89

Mahalanobis Distance: 11.1

Mahalanobis Distance p-value: 0.00221

Prediction: Positive if the Bayesian score is above the estimated best cutoff value from minimizing the false positive and false negative rate.
Probability: The esimated probability that the sample is in the positive category. This assumes that the Bayesian score follows a normal distribution and is different from the prediction using a cutoff.
Enrichment: An estimate of enrichment, that is, the increased likelihood (versus random) of this sample being in the category.
Bayesian Score: The standard Laplacian-modified Bayesian score.
Mahalanobis Distance: The Mahalanobis distance (MD) is the distance to the center of the training data. The larger the MD, the less trustworthy the prediction.
Mahalanobis Distance p-value: The p-value gives the fraction of training data with an MD greater than or equal to the one for the given sample, assuming normally distributed data. The smaller the p-value, the less trustworthy the prediciton. For highly non-normal X properties (e.g., fingerprints), the MD p-value is wildly inaccurate.

Structural Similar Compounds

Name	Glimepride	Labetalol	Lansoprazole
Structure	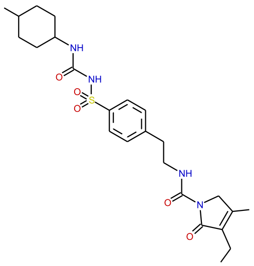	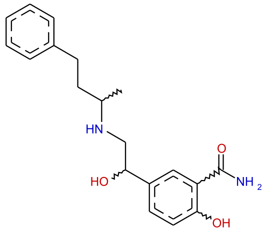	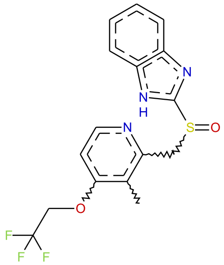
Actual Endpoint	Single-Carcinogen	Single-Carcinogen	Single-Carcinogen
Predicted Endpoint	Single-Carcinogen	Single-Carcinogen	Single-Carcinogen
Distance	0.599	0.808	0.820
Reference	US FDA (Centre for Drug Eval.& Res./Off. Testing & Res.) Sept. 1997	US FDA (Centre for Drug Eval.& Res./Off. Testing & Res.) Sept. 1997	US FDA (Centre for Drug Eval.& Res./Off. Testing & Res.) Sept. 1997

Model Applicability

Unknown features are fingerprint features in the query molecule, but not found or appearing too infrequently in the training set.

1. All properties and OPS components are within expected ranges.
2. Unknown ECFP_2 feature: 1336678434: [*][c](:[*]):[c](C([*])([*])[*]):c:[*]
3. Unknown ECFP_2 feature: -1952889961: [*]:[c](:[*])C(F)(F)F

Feature Contribution

Top features for positive contribution

Fingerprint	Bit/Smiles	Feature Structure	Score	Multiple-Carcinogen in training set
ECFP_4	-834094296	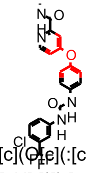 [*]:[cH]:[c](O[c](:[cH]:[*]):[cH]:[*]):[cH]:[*]	0.351	1 out of 1

ECFP_4	143734695	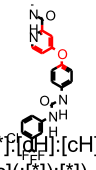 [*][c]1:[*]:[cH]:[cH]:[c](O[c](:[*]):[*]):[cH]:1	0.351	1 out of 1
ECFP_4	1407472008	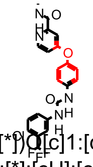 [*]:[c](:[*])O[c]1:[cH]:[cH]:[*]:[cH]:[cH]:1	0.351	1 out of 1

Top Features for negative contribution

Fingerprint	Bit/Smiles	Feature Structure	Score	Multiple-Carcinogen in training set
ECFP_4	1335691903	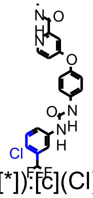 [*][c](:[*]):[c](Cl):[cH]:[*]	-0.8	0 out of 3
ECFP_4	888054369	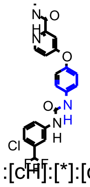 [*]N[c]1:[cH]:[*]:[c]([*]):[cH]:[cH]:1	-0.8	0 out of 3
ECFP_4	738938915	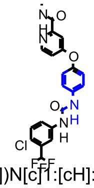 [*]C(=[*])N[c]1:[cH]:[cH]:[*]:[cH]:[cH]:1	-0.597	0 out of 2

7a TOPKAT_Mouse_Male_FDA_None_vs_Carcinogen

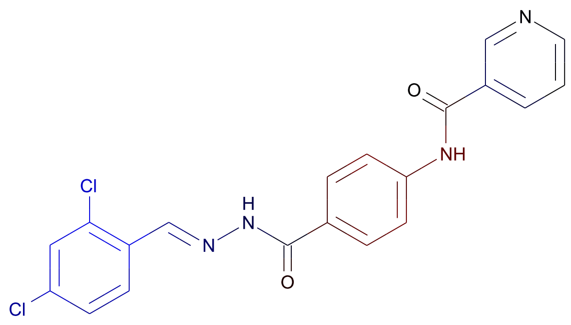

$C_{20}H_{14}Cl_2N_4O_2$
Molecular Weight: 413.25676
ALogP: 3.646
Rotatable Bonds: 5
Acceptors: 4
Donors: 2

Model Prediction

Prediction: Non-Carcinogen
Probability: 0.198
Enrichment: 0.672
Bayesian Score: -5.12
Mahalanobis Distance: 12.1
Mahalanobis Distance p-value: 0.00432

Prediction: Positive if the Bayesian score is above the estimated best cutoff value from minimizing the false positive and false negative rate.
Probability: The esimated probability that the sample is in the positive category. This assumes that the Bayesian score follows a normal distribution and is different from the prediction using a cutoff.
Enrichment: An estimate of enrichment, that is, the increased likelihood (versus random) of this sample being in the category.
Bayesian Score: The standard Laplacian-modified Bayesian score.
Mahalanobis Distance: The Mahalanobis distance (MD) is the distance to the center of the training data. The larger the MD, the less trustworthy the prediction.
Mahalanobis Distance p-value: The p-value gives the fraction of training data with an MD greater than or equal to the one for the given sample, assuming normally distributed data. The smaller the p-value, the less trustworthy the prediciton. For highly non-normal X properties (e.g., fingerprints), the MD p-value is wildly inaccurate.

Structural Similar Compounds

Name	Bicalutamide	Indapamide	Indomethacin
Structure	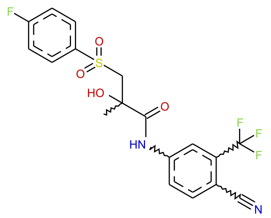	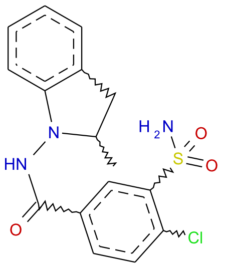	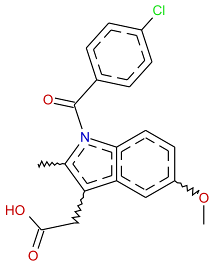
Actual Endpoint	Carcinogen	Non-Carcinogen	Non-Carcinogen
Predicted Endpoint	Carcinogen	Non-Carcinogen	Non-Carcinogen
Distance	0.552	0.614	0.620
Reference	US FDA (Centre for Drug Eval.& Res./Off. Testing & Res.) Sept. 1997	US FDA (Centre for Drug Eval.& Res./Off. Testing & Res.) Sept. 1997	US FDA (Centre for Drug Eval.& Res./Off. Testing & Res.) Sept. 1997

Model Applicability

Unknown features are fingerprint features in the query molecule, but not found or appearing too infrequently in the training set.

1. OPS PC16 out of range. Value: -3.2803. Training min, max, SD, explained variance: -3.1219, 5.3717, 1.291, 0.0175.

Feature Contribution

Top features for positive contribution				
**Fingerprint**	**Bit/Smiles**	**Feature Structure**	**Score**	**Carcinogen in training set**
FCFP_6	-581879738	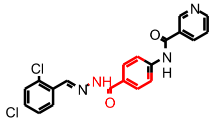 [*]NC(=O)[c]1:[cH]:[cH]:[*]:[cH]:[cH]:1	0.77	4 out of 5

FCFP_6	-1838187238	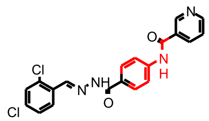 [*]C(=[*])N[c]1:[cH]:[cH]:[*]:[cH]:[cH]:1	0.565	4 out of 7
FCFP_6	1294255210	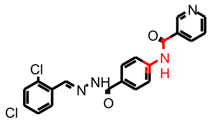 [*]C(=[*])N[c](:[*]):[*]	0.441	12 out of 28

Top Features for negative contribution

Fingerprint	Bit/Smiles	Feature Structure	Score	Carcinogen in training set
FCFP_6	2104062943	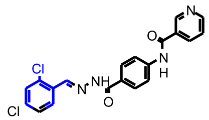 [*][c]1:[*]:[cH]:[c](C=[*]):[c](Cl):[cH]:1	-1.01	1 out of 17
FCFP_6	-885520711	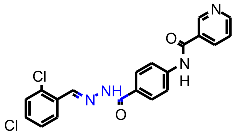 [*]C(=[*])NN=[*]	-0.839	0 out of 5
FCFP_6	555188808	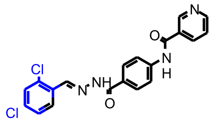 [*][c]1:[*]:[cH]:[c](Cl):[cH]:[c]:1Cl	-0.839	0 out of 5

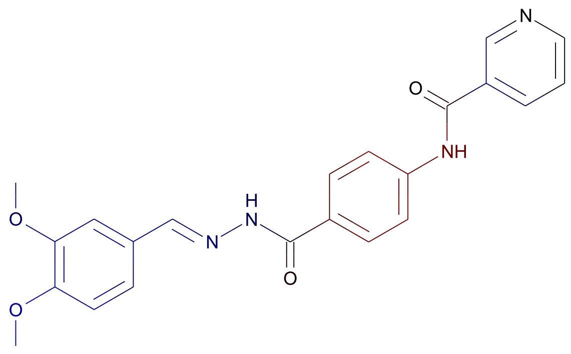

$C_{22}H_{20}N_4O_4$
Molecular Weight: 404.4186
ALogP: 2.284
Rotatable Bonds: 7
Acceptors: 6
Donors: 2

Model Prediction

Prediction: Non-Carcinogen
Probability: 0.223
Enrichment: 0.758
Bayesian Score: -3.64
Mahalanobis Distance: 13.5
Mahalanobis Distance p-value: 5.33e-005

Prediction: Positive if the Bayesian score is above the estimated best cutoff value from minimizing the false positive and false negative rate.
Probability: The esimated probability that the sample is in the positive category. This assumes that the Bayesian score follows a normal distribution and is different from the prediction using a cutoff.
Enrichment: An estimate of enrichment, that is, the increased likelihood (versus random) of this sample being in the category.
Bayesian Score: The standard Laplacian-modified Bayesian score.
Mahalanobis Distance: The Mahalanobis distance (MD) is the distance to the center of the training data. The larger the MD, the less trustworthy the prediction.
Mahalanobis Distance p-value: The p-value gives the fraction of training data with an MD greater than or equal to the one for the given sample, assuming normally distributed data. The smaller the p-value, the less trustworthy the prediciton. For highly non-normal X properties (e.g., fingerprints), the MD p-value is wildly inaccurate.

Structural Similar Compounds

Name	Bicalutamide	Moricizine	Cisapride
Structure	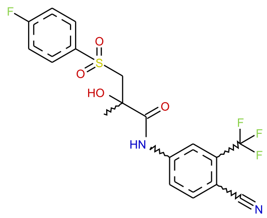	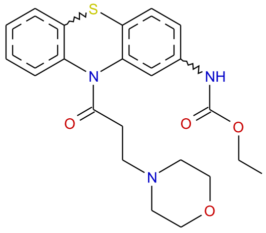	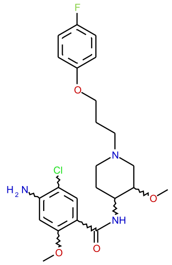
Actual Endpoint	Carcinogen	Non-Carcinogen	Non-Carcinogen
Predicted Endpoint	Carcinogen	Non-Carcinogen	Non-Carcinogen
Distance	0.573	0.621	0.626
Reference	US FDA (Centre for Drug Eval.& Res./Off. Testing & Res.) Sept. 1997	US FDA (Centre for Drug Eval.& Res./Off. Testing & Res.) Sept. 1997	US FDA (Centre for Drug Eval.& Res./Off. Testing & Res.) Sept. 1997

Model Applicability

Unknown features are fingerprint features in the query molecule, but not found or appearing too infrequently in the training set.

1. OPS PC16 out of range. Value: -3.8728. Training min, max, SD, explained variance: -3.1219, 5.3717, 1.291, 0.0175.

Feature Contribution

Top features for positive contribution				
**Fingerprint**	**Bit/Smiles**	**Feature Structure**	**Score**	**Carcinogen in training set**
FCFP_6	-581879738	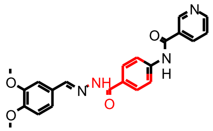 [*]NC(=O)[c]1:[cH]:[cH]:[*]:[cH]:[cH]:1	0.77	4 out of 5

FCFP_6	-1838187238	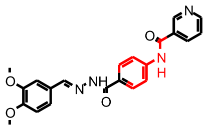 [*]C(=[*])N[c]1:[cH]:[cH]:[*]:[cH]:[cH]:1	0.565	4 out of 7
FCFP_6	1294255210	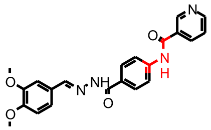 [*]C(=[*])N[c](:[*]):[*]	0.441	12 out of 28
**Top Features for negative contribution**				
**Fingerprint**	**Bit/Smiles**	**Feature Structure**	**Score**	**Carcinogen in training set**
FCFP_6	-885520711	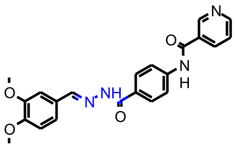 [*]C(=[*])NN=[*]	-0.839	0 out of 5
FCFP_6	-1038421835	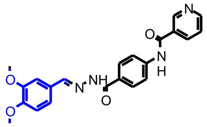 [*]O[c]1:[cH]:[c](C=[*]):[cH]:[cH]:[c]:1OC	-0.719	0 out of 4
FCFP_6	1028934530	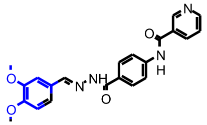 [*]O[c]1:[cH]:[*]:[c]([*]):[cH]:[c]:1OC	-0.596	1 out of 10

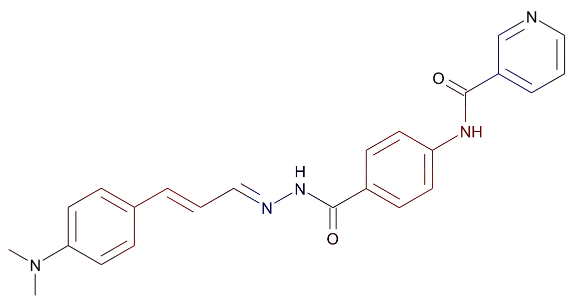

$C_{24}H_{23}N_5O_2$
Molecular Weight: 413.47172
ALogP: 2.839
Rotatable Bonds: 7
Acceptors: 5
Donors: 2

Model Prediction

Prediction: Carcinogen
Probability: 0.365
Enrichment: 1.24
Bayesian Score: 1.98
Mahalanobis Distance: 12.2
Mahalanobis Distance p-value: 0.00379

Prediction: Positive if the Bayesian score is above the estimated best cutoff value from minimizing the false positive and false negative rate.
Probability: The esimated probability that the sample is in the positive category. This assumes that the Bayesian score follows a normal distribution and is different from the prediction using a cutoff.
Enrichment: An estimate of enrichment, that is, the increased likelihood (versus random) of this sample being in the category.
Bayesian Score: The standard Laplacian-modified Bayesian score.
Mahalanobis Distance: The Mahalanobis distance (MD) is the distance to the center of the training data. The larger the MD, the less trustworthy the prediction.
Mahalanobis Distance p-value: The p-value gives the fraction of training data with an MD greater than or equal to the one for the given sample, assuming normally distributed data. The smaller the p-value, the less trustworthy the prediciton. For highly non-normal X properties (e.g., fingerprints), the MD p-value is wildly inaccurate.

Structural Similar Compounds

Name	Bicalutamide	Flecainide	Cisapride
Structure	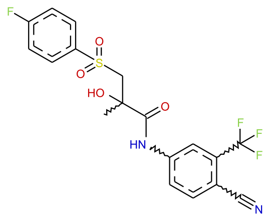	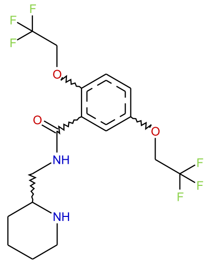	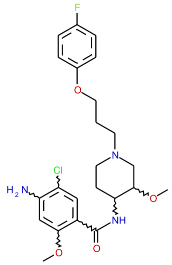
Actual Endpoint	Carcinogen	Non-Carcinogen	Non-Carcinogen
Predicted Endpoint	Carcinogen	Non-Carcinogen	Non-Carcinogen
Distance	0.568	0.625	0.625
Reference	US FDA (Centre for Drug Eval.& Res./Off. Testing & Res.) Sept. 1997	US FDA (Centre for Drug Eval.& Res./Off. Testing & Res.) Sept. 1997	US FDA (Centre for Drug Eval.& Res./Off. Testing & Res.) Sept. 1997

Model Applicability

Unknown features are fingerprint features in the query molecule, but not found or appearing too infrequently in the training set.

1. OPS PC16 out of range. Value: -3.3523. Training min, max, SD, explained variance: -3.1219, 5.3717, 1.291, 0.0175.
2. Unknown FCFP_2 feature: -2100309237: [*]\N=C\C=[*]

Feature Contribution

Top features for positive contribution

Fingerprint	Bit/Smiles	Feature Structure	Score	Carcinogen in training set
FCFP_6	-1033057683	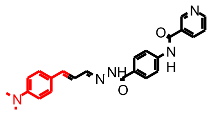 [*]=C[c]1:[cH]:[cH]:[c](:[cH]:[cH]:1)N(C)C	0.805	3 out of 3

FCFP_6	-581879738	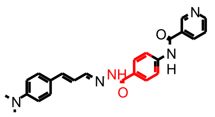 [*]NC(=O)[c]1:[cH]:[cH]:[*]:[cH]:[cH]:1	0.77	4 out of 5
FCFP_6	-146015125	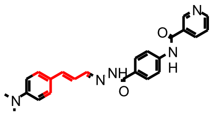 [*]=C\C=C\[c](:[cH]:[*]):[cH]:[*]	0.676	2 out of 2

Top Features for negative contribution

Fingerprint	Bit/Smiles	Feature Structure	Score	Carcinogen in training set
FCFP_6	-885520711	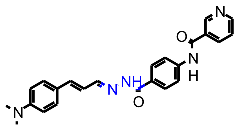 [*]C(=[*])NN=[*]	-0.839	0 out of 5
FCFP_6	1153798395	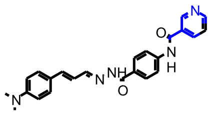 [*]C(=[*])[c]1:[cH]:[*]:[cH]:n:[cH]:1	-0.582	0 out of 3
FCFP_6	581019816	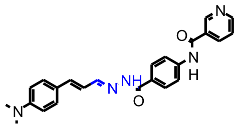 [*]N\N=C\[*]	-0.423	0 out of 2

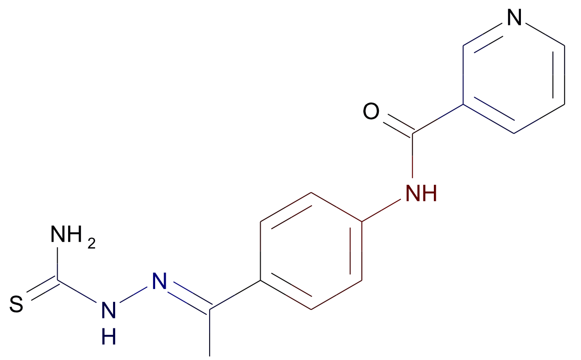

$C_{15}H_{15}N_5OS$
Molecular Weight: 313.3775
ALogP: 1.414
Rotatable Bonds: 5
Acceptors: 4
Donors: 3

Structural Similar Compounds

Name	Furosemide	Tolbutamide	Sotalol
Structure	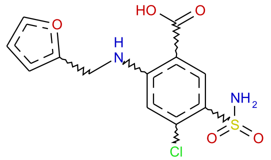	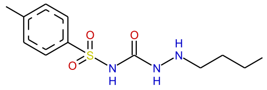	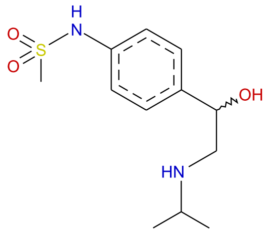
Actual Endpoint	Non-Carcinogen	Non-Carcinogen	Non-Carcinogen
Predicted Endpoint	Non-Carcinogen	Non-Carcinogen	Carcinogen
Distance	0.518	0.548	0.553
Reference	US FDA (Centre for Drug Eval.& Res./Off. Testing & Res.) Sept. 1997	US FDA (Centre for Drug Eval.& Res./Off. Testing & Res.) Sept. 1997	US FDA (Centre for Drug Eval.& Res./Off. Testing & Res.) Sept. 1997

Model Prediction

Prediction: Carcinogen
Probability: 0.294
Enrichment: 0.998
Bayesian Score: -0.458
Mahalanobis Distance: 13.3
Mahalanobis Distance p-value: 0.000102

Prediction: Positive if the Bayesian score is above the estimated best cutoff value from minimizing the false positive and false negative rate.
Probability: The esimated probability that the sample is in the positive category. This assumes that the Bayesian score follows a normal distribution and is different from the prediction using a cutoff.
Enrichment: An estimate of enrichment, that is, the increased likelihood (versus random) of this sample being in the category.
Bayesian Score: The standard Laplacian-modified Bayesian score.
Mahalanobis Distance: The Mahalanobis distance (MD) is the distance to the center of the training data. The larger the MD, the less trustworthy the prediction.
Mahalanobis Distance p-value: The p-value gives the fraction of training data with an MD greater than or equal to the one for the given sample, assuming normally distributed data. The smaller the p-value, the less trustworthy the prediciton. For highly non-normal X properties (e.g., fingerprints), the MD p-value is wildly inaccurate.

Model Applicability

Unknown features are fingerprint features in the query molecule, but not found or appearing too infrequently in the training set.

1. All properties and OPS components are within expected ranges.

Feature Contribution

Top features for positive contribution

Fingerprint	Bit/Smiles	Feature Structure	Score	Carcinogen in training set
FCFP_6	-1838187238	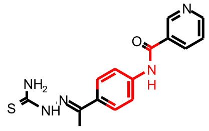 [*]C(=[*])N[c]1:[cH]:[cH]:[*]:[cH]:[cH]:1	0.565	4 out of 7

FCFP_6	1294255210	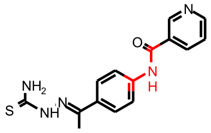 [*]C(=[*])N[c](:[*]):[*]	0.441	12 out of 28
FCFP_6	-773983804	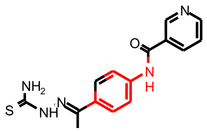 [*]N[c]1:[cH]:[*]:[c]([*]):[cH]:[cH]:1	0.409	10 out of 24

Top Features for negative contribution

Fingerprint	Bit/Smiles	Feature Structure	Score	Carcinogen in training set
FCFP_6	-885520711	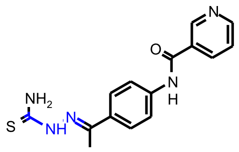 [*]C(=[*])NN=[*]	-0.839	0 out of 5
FCFP_6	1153798395	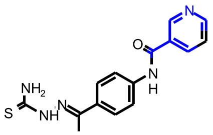 [*]C(=[*])[c]1:[cH]:[*]:[cH]:n:[cH]:1	-0.582	0 out of 3
FCFP_6	-1549192822	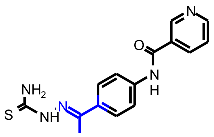 [*]\N=C(/C)\[c](:[*]):[*]	-0.489	3 out of 21

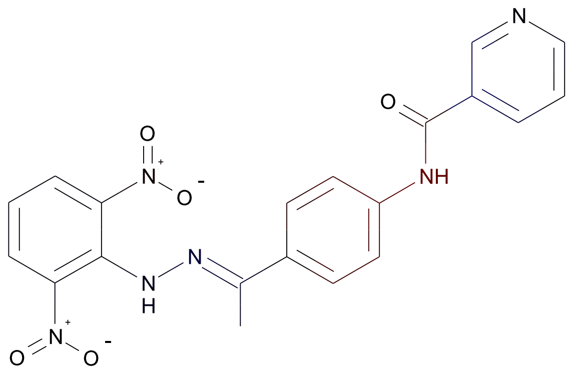

$C_{20}H_{16}N_6O_5$
Molecular Weight: 420.37824
ALogP: 2.852
Rotatable Bonds: 7
Acceptors: 8
Donors: 2

Model Prediction

Prediction: Carcinogen
Probability: 0.29
Enrichment: 0.984
Bayesian Score: -0.612
Mahalanobis Distance: 13.2
Mahalanobis Distance p-value: 0.000151

Prediction: Positive if the Bayesian score is above the estimated best cutoff value from minimizing the false positive and false negative rate.
Probability: The esimated probability that the sample is in the positive category. This assumes that the Bayesian score follows a normal distribution and is different from the prediction using a cutoff.
Enrichment: An estimate of enrichment, that is, the increased likelihood (versus random) of this sample being in the category.
Bayesian Score: The standard Laplacian-modified Bayesian score.
Mahalanobis Distance: The Mahalanobis distance (MD) is the distance to the center of the training data. The larger the MD, the less trustworthy the prediction.
Mahalanobis Distance p-value: The p-value gives the fraction of training data with an MD greater than or equal to the one for the given sample, assuming normally distributed data. The smaller the p-value, the less trustworthy the prediciton. For highly non-normal X properties (e.g., fingerprints), the MD p-value is wildly inaccurate.

Structural Similar Compounds

Name	Sulfasalazine	Nedocromil	Nisoldipine
Structure	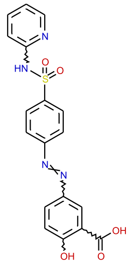	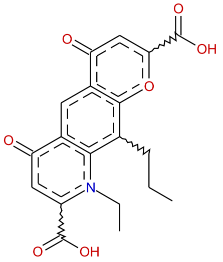	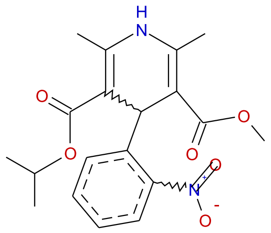
Actual Endpoint	Carcinogen	Non-Carcinogen	Non-Carcinogen
Predicted Endpoint	Carcinogen	Non-Carcinogen	Non-Carcinogen
Distance	0.596	0.619	0.672
Reference	US FDA (Centre for Drug Eval.& Res./Off. Testing & Res.) Sept. 1997	US FDA (Centre for Drug Eval.& Res./Off. Testing & Res.) Sept. 1997	US FDA (Centre for Drug Eval.& Res./Off. Testing & Res.) Sept. 1997

Model Applicability

Unknown features are fingerprint features in the query molecule, but not found or appearing too infrequently in the training set.

1. All properties and OPS components are within expected ranges.
2. Unknown FCFP_2 feature: 5: [*][O-]
3. Unknown FCFP_2 feature: 1294285001: [*]=NN[c](:[*]):[*]
4. Unknown FCFP_2 feature: -828984032: [*][c](:[*]):[c]([N+](=[*])[*]):c:[*]
5. Unknown FCFP_2 feature: -1338588315: [*]:[c](:[*])[N+](=O)[O-]
6. Unknown FCFP_2 feature: 1872392852: [*][N+](=O)[*]
7. Unknown FCFP_2 feature: 260476081: [*][N+](=[*])[O-]

Feature Contribution

Top features for positive contribution

Fingerprint	Bit/Smiles	Feature Structure	Score	Carcinogen in training set

FCFP_6	-1838187238	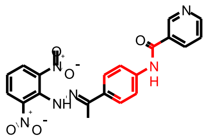 [*]C(=[*])N[c]1:[cH]:[cH]:[*]:[cH]:[cH]:1	0.565	4 out of 7
FCFP_6	1294255210	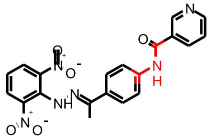 [*]C(=[*])N[c](:[*]):[*]	0.441	12 out of 28
FCFP_6	-773983804	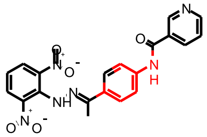 [*]N[c]1:[cH]:[*]:[c]([*]):[cH]:[cH]:1	0.409	10 out of 24

Top Features for negative contribution

**Fingerprint**	**Bit/Smiles**	**Feature Structure**	**Score**	**Carcinogen in training set**
FCFP_6	1153798395	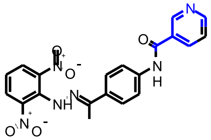 [*]C(=[*])[c]1:[cH]:[*]:[cH]:n:[cH]:1	-0.582	0 out of 3
FCFP_6	-1549192822	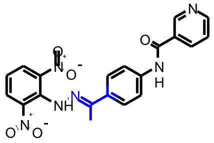 [*]\N=C(/C)\[c](:[*]):[*]	-0.489	3 out of 21

FCFP_6	581019816	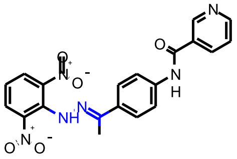 [*]N\N=C\[*]	-0.423	0 out of 2

Sorafenib

TOPKAT_Mouse_Male_FDA_None_vs_Carcinogen

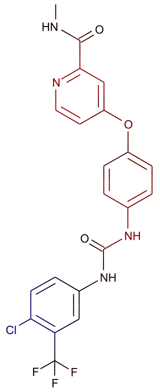

$C_{21}H_{16}ClF_3N_4O_3$
Molecular Weight: 464.82494
ALogP: 4.175
Rotatable Bonds: 6
Acceptors: 4
Donors: 3

Model Prediction

Prediction: Carcinogen
Probability: 0.444
Enrichment: 1.51
Bayesian Score: 4.21
Mahalanobis Distance: 20.3
Mahalanobis Distance p-value: 1.28e-019

Prediction: Positive if the Bayesian score is above the estimated best cutoff value from minimizing the false positive and false negative rate.
Probability: The esimated probability that the sample is in the positive category. This assumes that the Bayesian score follows a normal distribution and is different from the prediction using a cutoff.
Enrichment: An estimate of enrichment, that is, the increased likelihood (versus random) of this sample being in the category.
Bayesian Score: The standard Laplacian-modified Bayesian score.
Mahalanobis Distance: The Mahalanobis distance (MD) is the distance to the center of the training data. The larger the MD, the less trustworthy the prediction.
Mahalanobis Distance p-value: The p-value gives the fraction of training data with an MD greater than or equal to the one for the given sample, assuming normally distributed data. The smaller the p-value, the less trustworthy the prediciton. For highly non-normal X properties (e.g., fingerprints), the MD p-value is wildly inaccurate.

Structural Similar Compounds

Name	Glyburide	Glimepride	Fluvastatin
Structure	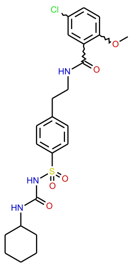	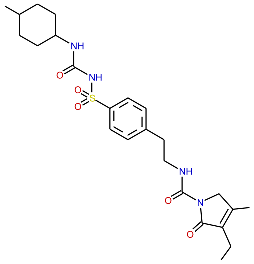	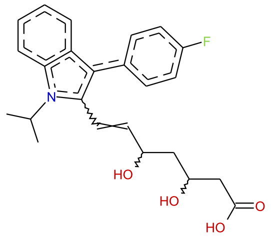
Actual Endpoint	Non-Carcinogen	Carcinogen	Non-Carcinogen
Predicted Endpoint	Non-Carcinogen	Carcinogen	Non-Carcinogen
Distance	0.594	0.599	0.603
Reference	US FDA (Centre for Drug Eval.& Res./Off. Testing & Res.) Sept. 1997	US FDA (Centre for Drug Eval.& Res./Off. Testing & Res.) Sept. 1997	US FDA (Centre for Drug Eval.& Res./Off. Testing & Res.) Sept. 1997

Model Applicability

Unknown features are fingerprint features in the query molecule, but not found or appearing too infrequently in the training set.

1. All properties and OPS components are within expected ranges.

Feature Contribution

Top features for positive contribution

Fingerprint	Bit/Smiles	Feature Structure	Score	Carcinogen in training set
FCFP_6	71953198	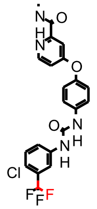 [*]C([*])([*])F	0.612	12 out of 23

FCFP_6	-1838187238	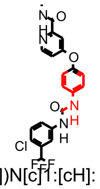 [*]C(=[*])N[c]1:[cH]:[cH]:[*]:[cH]:[cH]:1	0.565	4 out of 7
FCFP_6	859018953	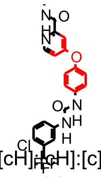 [*][c]1:[cH]:[cH]:[c](O[c](:[cH]:[*]):[cH]:[*]):[cH]:[cH]:1	0.46	1 out of 1

Top Features for negative contribution

Fingerprint	Bit/Smiles	Feature Structure	Score	Carcinogen in training set
FCFP_6	2104062943	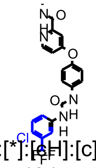 [*][c]1:[*]:[cH]:[c](C=[*]):[c](Cl):[cH]:1	-1.01	1 out of 17
FCFP_6	551850122	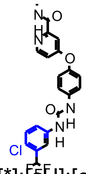 [*][c]1:[*]:[cH]:[c](Cl):[cH]:[cH]:1	-0.433	8 out of 49
FCFP_6	71476542	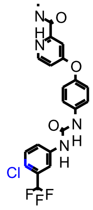 [*]:[c](:[*])Cl	-0.406	10 out of 59

TOPKAT_Mouse_Male_FDA_Single_vs_Multiple

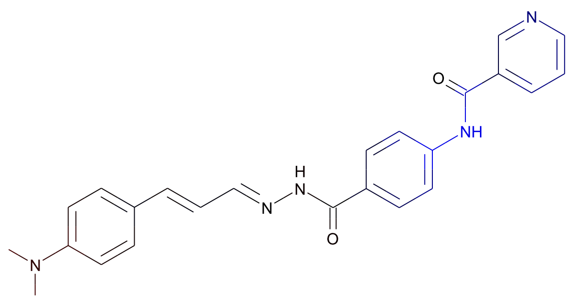

$C_{24}H_{23}N_5O_2$
Molecular Weight: 413.47172
ALogP: 2.839
Rotatable Bonds: 7
Acceptors: 5
Donors: 2

Model Prediction

Prediction: Single-Carcinogen
Probability: 0.146
Enrichment: 0.486
Bayesian Score: -8.65
Mahalanobis Distance: 15.7
Mahalanobis Distance p-value: 6.91e-007

Prediction: Positive if the Bayesian score is above the estimated best cutoff value from minimizing the false positive and false negative rate.
Probability: The esimated probability that the sample is in the positive category. This assumes that the Bayesian score follows a normal distribution and is different from the prediction using a cutoff.
Enrichment: An estimate of enrichment, that is, the increased likelihood (versus random) of this sample being in the category.
Bayesian Score: The standard Laplacian-modified Bayesian score.
Mahalanobis Distance: The Mahalanobis distance (MD) is the distance to the center of the training data. The larger the MD, the less trustworthy the prediction.
Mahalanobis Distance p-value: The p-value gives the fraction of training data with an MD greater than or equal to the one for the given sample, assuming normally distributed data. The smaller the p-value, the less trustworthy the prediciton. For highly non-normal X properties (e.g., fingerprints), the MD p-value is wildly inaccurate.

Structural Similar Compounds

Name	Bicalutamide	Lovastatin	Lansoprazole
Structure	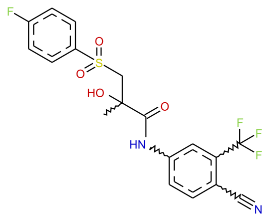	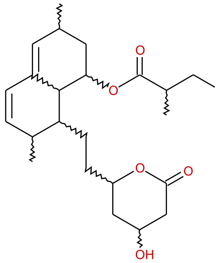	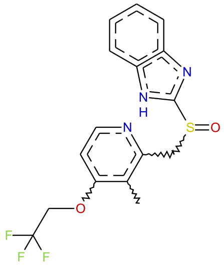
Actual Endpoint	Single-Carcinogen	Multiple-Carcinogen	Single-Carcinogen
Predicted Endpoint	Single-Carcinogen	Multiple-Carcinogen	Single-Carcinogen
Distance	0.590	0.709	0.718
Reference	US FDA (Centre for Drug Eval.& Res./Off. Testing & Res.) Sept. 1997	US FDA (Centre for Drug Eval.& Res./Off. Testing & Res.) Sept. 1997	US FDA (Centre for Drug Eval.& Res./Off. Testing & Res.) Sept. 1997

Model Applicability

Unknown features are fingerprint features in the query molecule, but not found or appearing too infrequently in the training set.

1. OPS PC15 out of range. Value: -2.782. Training min, max, SD, explained variance: -2.4461, 3.3002, 1.005, 0.0188.
2. Unknown FCFP_2 feature: -885520711: [*]C(=[*])NN=[*]
3. Unknown FCFP_2 feature: 581019816: [*]N\N=C\[*]
4. Unknown FCFP_2 feature: -2100309237: [*]\N=C\C=[*]

Feature Contribution

Top features for positive contribution

Fingerprint	Bit/Smiles	Feature Structure	Score	Multiple-Carcinogen in training set
FCFP_12	675769755	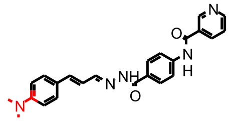 [*]:[c](:[*])N(C)C	0.573	5 out of 7

FCFP_12	547884906	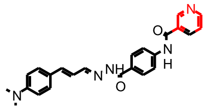 [*][c]1:[*]:[cH]:[cH]:n:[cH]:1	0.4	1 out of 1
FCFP_12	-1033057683	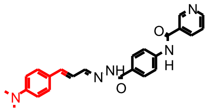 [*]=C[c]1:[cH]:[cH]:[c](:[cH]:[cH]:1)N(C)C	0.395	2 out of 3

Top Features for negative contribution

Fingerprint	Bit/Smiles	Feature Structure	Score	Multiple-Carcinogen in training set
FCFP_12	1294255210	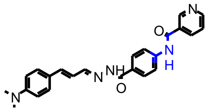 [*]C(=[*])N[c](:[*]):[*]	-1.63	0 out of 12
FCFP_12	590925877	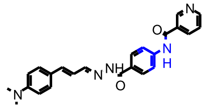 [*]N[c](:[cH]:[*]):[cH]:[*]	-0.998	1 out of 13
FCFP_12	-1838187238	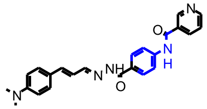 [*]C(=[*])N[c]1:[cH]:[cH]:[*]:[cH]:[cH]:1	-0.859	0 out of 4

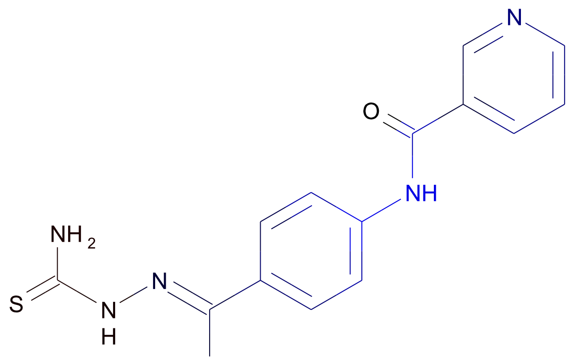

$C_{15}H_{15}N_5OS$
Molecular Weight: 313.3775
ALogP: 1.414
Rotatable Bonds: 5
Acceptors: 4
Donors: 3

Structural Similar Compounds

Name	Sulfamethazine	Primidolol	Procarbazine
Structure	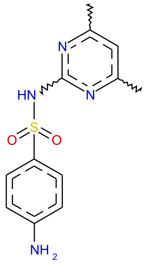	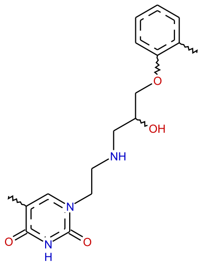	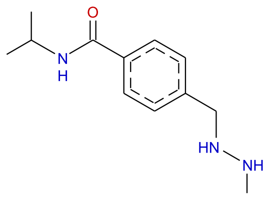
Actual Endpoint	Single-Carcinogen	Single-Carcinogen	Multiple-Carcinogen
Predicted Endpoint	Single-Carcinogen	Single-Carcinogen	Multiple-Carcinogen
Distance	0.698	0.745	0.746
Reference	US FDA (Centre for Drug Eval.& Res./Off. Testing & Res.) Sept. 1997	US FDA (Centre for Drug Eval.& Res./Off. Testing & Res.) Sept. 1997	US FDA (Centre for Drug Eval.& Res./Off. Testing & Res.) Sept. 1997

Model Prediction

Prediction: Single-Carcinogen
Probability: 0.146
Enrichment: 0.485
Bayesian Score: -8.89
Mahalanobis Distance: 17.3
Mahalanobis Distance p-value: 4.15e-008

Prediction: Positive if the Bayesian score is above the estimated best cutoff value from minimizing the false positive and false negative rate.
Probability: The esimated probability that the sample is in the positive category. This assumes that the Bayesian score follows a normal distribution and is different from the prediction using a cutoff.
Enrichment: An estimate of enrichment, that is, the increased likelihood (versus random) of this sample being in the category.
Bayesian Score: The standard Laplacian-modified Bayesian score.
Mahalanobis Distance: The Mahalanobis distance (MD) is the distance to the center of the training data. The larger the MD, the less trustworthy the prediction.
Mahalanobis Distance p-value: The p-value gives the fraction of training data with an MD greater than or equal to the one for the given sample, assuming normally distributed data. The smaller the p-value, the less trustworthy the prediciton. For highly non-normal X properties (e.g., fingerprints), the MD p-value is wildly inaccurate.

Model Applicability

Unknown features are fingerprint features in the query molecule, but not found or appearing too infrequently in the training set.

1. All properties and OPS components are within expected ranges.
2. Unknown FCFP_2 feature: 581019816: [*]N\N=C\[*]
3. Unknown FCFP_2 feature: -885520711: [*]C(=[*])NN=[*]

Feature Contribution

Top features for positive contribution

Fingerprint	Bit/Smiles	Feature Structure	Score	Multiple-Carcinogen in training set
FCFP_12	547884906	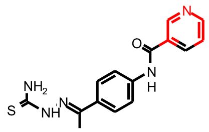 [*][c]1:[*]:[cH]:[cH]:n:[cH]:1	0.4	1 out of 1

FCFP_12	1499521844	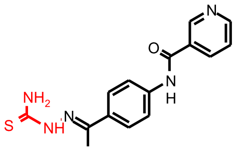 [*]NC(=S)N	0.39	5 out of 9
FCFP_12	-1549103449	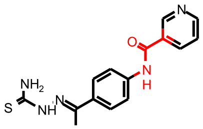 [*]NC(=O)[c](:[*]):[*]]	0.168	3 out of 7

Top Features for negative contribution

Fingerprint	Bit/Smiles	Feature Structure	Score	Multiple-Carcinogen in training set
FCFP_12	1294255210	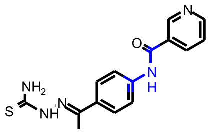 [*]C(=[*])N[c](:[*]): [*]	-1.63	0 out of 12
FCFP_12	590925877	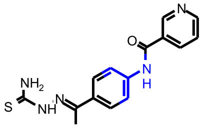 [*]N[c](:[cH]:[*]):[c H]:[*]	-0.998	1 out of 13
FCFP_12	-1838187238	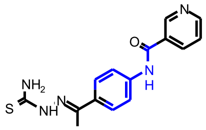 [*]C(=[*])N[c]1:[cH]: [cH]:[*]:[cH]:[cH]:1	-0.859	0 out of 4

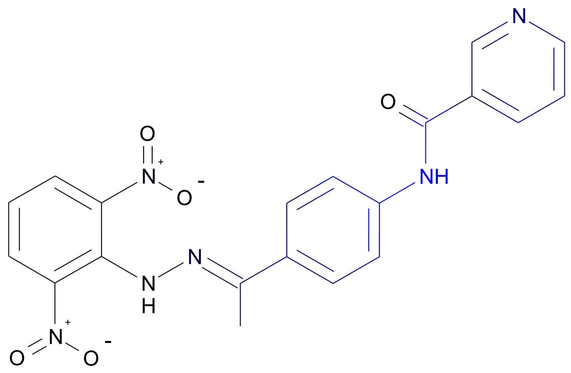

$C_{20}H_{16}N_6O_5$

Molecular Weight: 420.37824

ALogP: 2.852

Rotatable Bonds: 7

Acceptors: 8

Donors: 2

Structural Similar Compounds

Name	Sulfasalazine	Dihydroxymethylfuratrizine	Bicalutamide
Structure	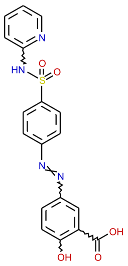	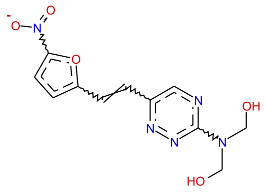	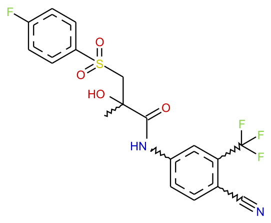
Actual Endpoint	Single-Carcinogen	Multiple-Carcinogen	Single-Carcinogen
Predicted Endpoint	Single-Carcinogen	Multiple-Carcinogen	Single-Carcinogen
Distance	0.663	0.774	0.787
Reference	US FDA (Centre for Drug Eval.& Res./Off. Testing & Res.) Sept. 1997	US FDA (Centre for Drug Eval.& Res./Off. Testing & Res.) Sept. 1997	US FDA (Centre for Drug Eval.& Res./Off. Testing & Res.) Sept. 1997

Model Prediction

Prediction: Single-Carcinogen

Probability: 0.148

Enrichment: 0.492

Bayesian Score: -11

Mahalanobis Distance: 14

Mahalanobis Distance p-value: 1.71e-005

Prediction: Positive if the Bayesian score is above the estimated best cutoff value from minimizing the false positive and false negative rate.
Probability: The esimated probability that the sample is in the positive category. This assumes that the Bayesian score follows a normal distribution and is different from the prediction using a cutoff.
Enrichment: An estimate of enrichment, that is, the increased likelihood (versus random) of this sample being in the category.
Bayesian Score: The standard Laplacian-modified Bayesian score.
Mahalanobis Distance: The Mahalanobis distance (MD) is the distance to the center of the training data. The larger the MD, the less trustworthy the prediction.
Mahalanobis Distance p-value: The p-value gives the fraction of training data with an MD greater than or equal to the one for the given sample, assuming normally distributed data. The smaller the p-value, the less trustworthy the prediciton. For highly non-normal X properties (e.g., fingerprints), the MD p-value is wildly inaccurate.

Model Applicability

Unknown features are fingerprint features in the query molecule, but not found or appearing too infrequently in the training set.

1. All properties and OPS components are within expected ranges.
2. Unknown FCFP_2 feature: 5: [*][O-]
3. Unknown FCFP_2 feature: 581019816: [*]N\N=C\[*]
4. Unknown FCFP_2 feature: 1294285001: [*]=NN[c](:[*]):[*]
5. Unknown FCFP_2 feature: -828984032: [*][c](:[*]):[c]([N+](=[*])[*]):c:[*]
6. Unknown FCFP_2 feature: -1338588315: [*]:[c](:[*])[N+](=O)[O-]
7. Unknown FCFP_2 feature: 1872392852: [*][N+](=O)[*]
8. Unknown FCFP_2 feature: 260476081: [*][N+](=[*])[O-]

Feature Contribution

Top features for positive contribution				
Fingerprint	Bit/Smiles	Feature Structure	Score	Multiple-Carcinogen in training set

FCFP_12	547884906	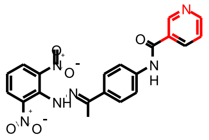 [*][c]1:[*]:[cH]:[cH]:n:[cH]:1	0.4	1 out of 1
FCFP_12	-1549103449	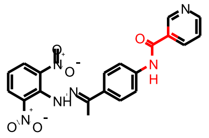 [*]NC(=O)[c](:[*]):[*]	0.168	3 out of 7
FCFP_12	136597326	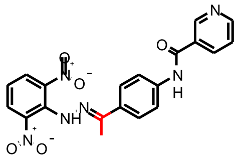 [*]C(=[*])C	0.0722	18 out of 49
**Top Features for negative contribution**				
**Fingerprint**	**Bit/Smiles**	**Feature Structure**	**Score**	**Multiple-Carcinogen in training set**
FCFP_12	1294255210	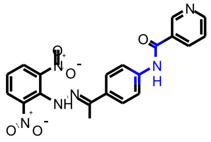 [*]C(=[*])N[c](:[*]):[*]	-1.63	0 out of 12
FCFP_12	590925877	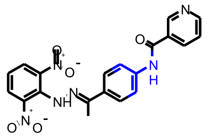 [*]N[c](:[cH]:[*]):[cH]:[*]	-0.998	1 out of 13

FCFP_12	-1838187238	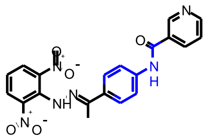 [*]C(=[*])N[c]1:[cH]:[cH]:[*]:[cH]:[cH]:1	-0.859	0 out of 4

Sorafenib

TOPKAT_Mouse_Male_FDA_Single_vs_Multiple

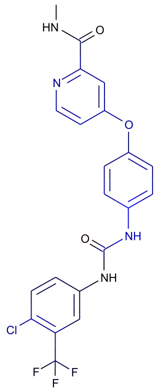

$C_{21}H_{16}ClF_3N_4O_3$
Molecular Weight: 464.82494
ALogP: 4.175
Rotatable Bonds: 6
Acceptors: 4
Donors: 3

Model Prediction

Prediction: Single-Carcinogen
Probability: 0.139
Enrichment: 0.461
Bayesian Score: -14.7
Mahalanobis Distance: 21.3
Mahalanobis Distance p-value: 4.93e-011

Prediction: Positive if the Bayesian score is above the estimated best cutoff value from minimizing the false positive and false negative rate.
Probability: The esimated probability that the sample is in the positive category. This assumes that the Bayesian score follows a normal distribution and is different from the prediction using a cutoff.
Enrichment: An estimate of enrichment, that is, the increased likelihood (versus random) of this sample being in the category.
Bayesian Score: The standard Laplacian-modified Bayesian score.
Mahalanobis Distance: The Mahalanobis distance (MD) is the distance to the center of the training data. The larger the MD, the less trustworthy the prediction.
Mahalanobis Distance p-value: The p-value gives the fraction of training data with an MD greater than or equal to the one for the given sample, assuming normally distributed data. The smaller the p-value, the less trustworthy the prediciton. For highly non-normal X properties (e.g., fingerprints), the MD p-value is wildly inaccurate.

Structural Similar Compounds

Name	Glimepride	Bicalutamide	Lansoprazole
Structure	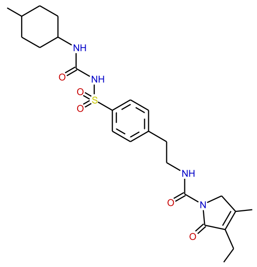	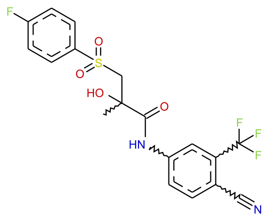	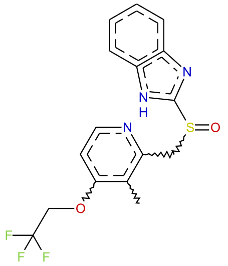
Actual Endpoint	Single-Carcinogen	Single-Carcinogen	Single-Carcinogen
Predicted Endpoint	Single-Carcinogen	Single-Carcinogen	Single-Carcinogen
Distance	0.626	0.700	0.866
Reference	US FDA (Centre for Drug Eval.& Res./Off. Testing & Res.) Sept. 1997	US FDA (Centre for Drug Eval.& Res./Off. Testing & Res.) Sept. 1997	US FDA (Centre for Drug Eval.& Res./Off. Testing & Res.) Sept. 1997

Model Applicability

Unknown features are fingerprint features in the query molecule, but not found or appearing too infrequently in the training set.

1. All properties and OPS components are within expected ranges.

Feature Contribution

Top features for positive contribution

Fingerprint	Bit/Smiles	Feature Structure	Score	Multiple-Carcinogen in training set
FCFP_12	1499521844	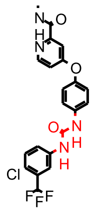 [*]NC(=S)N	0.39	5 out of 9

FCFP_12	-904785030	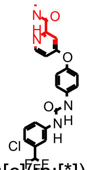 [*]:[cH]:[c](:n:[*])C(=O)NC	0.174	1 out of 2
FCFP_12	-1549103449	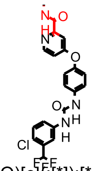 [*]NC(=O)[c](:[*]):[*]	0.168	3 out of 7

Top Features for negative contribution

Fingerprint	Bit/Smiles	Feature Structure	Score	Multiple-Carcinogen in training set
FCFP_12	1294255210	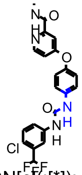 [*]C(=[*])N[c](:[*]):[*]	-1.63	0 out of 12
FCFP_12	590925877	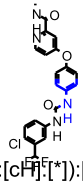 [*]N[c](:[cH]:[*]):[cH]:[*]	-0.998	1 out of 13
FCFP_12	-1462709112	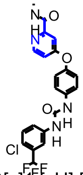 [*]C(=[*])[c]1:[cH]:[*]:[cH]:[cH]:n:1	-0.994	0 out of 5

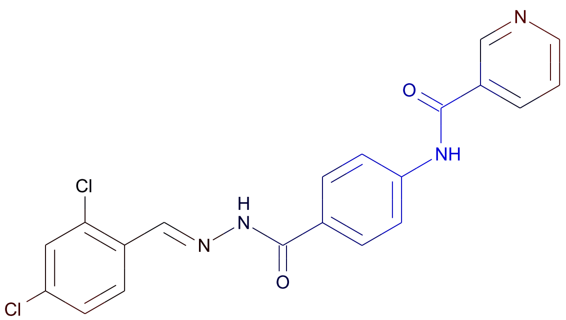

$C_{20}H_{14}Cl_2N_4O_2$
Molecular Weight: 413.25676
ALogP: 3.646
Rotatable Bonds: 5
Acceptors: 4
Donors: 2

Structural Similar Compounds

Name	1-AMINO-4-BENZOYLAMINO-ANTHRAQUINONE	5-NORBORNENE-2;3-DICARBOXYLIC ACID; 1;4;5;6;7;7-HEXACHLORO-	1-BENZOYLAMINO-4-METHOXY-5-CHLORANTHRAQUINONE
Structure	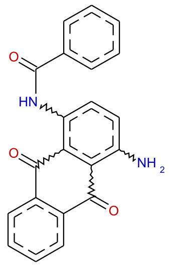	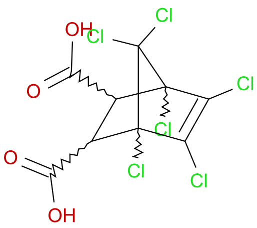	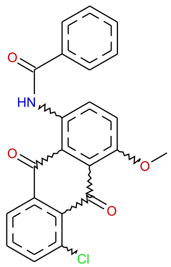
Actual Endpoint	Mild	Moderate_Severe	Mild
Predicted Endpoint	Mild	Moderate_Severe	Mild
Distance	0.629	0.641	0.674
Reference	28ZPAK-;124;72	28ZPAK-;92;72	28ZPAK-;90;72

Model Prediction

Prediction: Mild
Probability: 0.69
Enrichment: 1
Bayesian Score: -3.69
Mahalanobis Distance: 6.1
Mahalanobis Distance p-value: 1

Prediction: Positive if the Bayesian score is above the estimated best cutoff value from minimizing the false positive and false negative rate.
Probability: The esimated probability that the sample is in the positive category. This assumes that the Bayesian score follows a normal distribution and is different from the prediction using a cutoff.
Enrichment: An estimate of enrichment, that is, the increased likelihood (versus random) of this sample being in the category.
Bayesian Score: The standard Laplacian-modified Bayesian score.
Mahalanobis Distance: The Mahalanobis distance (MD) is the distance to the center of the training data. The larger the MD, the less trustworthy the prediction.
Mahalanobis Distance p-value: The p-value gives the fraction of training data with an MD greater than or equal to the one for the given sample, assuming normally distributed data. The smaller the p-value, the less trustworthy the prediciton. For highly non-normal X properties (e.g., fingerprints), the MD p-value is wildly inaccurate.

Model Applicability

Unknown features are fingerprint features in the query molecule, but not found or appearing too infrequently in the training set.

1. All properties and OPS components are within expected ranges.
2. Unknown FCFP_2 feature: 581019816: [*]N\N=C\[*]

Feature Contribution

Top features for positive contribution				
**Fingerprint**	**Bit/Smiles**	**Feature Structure**	**Score**	**Moderate_Severe in training set**
FCFP_10	-149636017	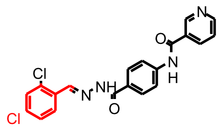 [*][c]1:[cH]:[c](Cl):[cH]:[cH]:[c]:1C=[*]	0.352	7 out of 7

FCFP_10	547884906	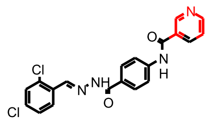<br>[*][c]1:[*]:[cH]:[cH]<br>:n:[cH]:1	0.317	4 out of 4
FCFP_10	73264552	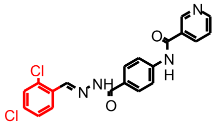<br>[*][c]1:[cH]:[cH]:[c]<br>(Cl):[cH]:[c]:1Cl	0.317	4 out of 4
**Top Features for negative contribution**				
**Fingerprint**	**Bit/Smiles**	**Feature Structure**	**Score**	**Moderate_Severe in training set**
FCFP_10	-1925475824	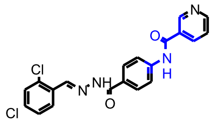<br>[*]:[cH]:[c](:[cH]:[*<br>])C(=O)N[c](:[*]):[*<br>]	-1.29	0 out of 4
FCFP_10	-581879738	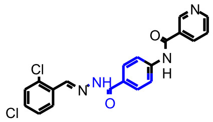<br>[*]NC(=O)[c]1:[cH]:[c<br>H]:[*]:[cH]:[cH]:1	-1.29	0 out of 4
FCFP_10	1175232969	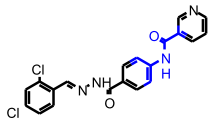<br>[*]:[cH]:[c](NC(=O)[c<br>](:[*]):[*]):[cH]:[*<br>]	-1.29	0 out of 4

TOPKAT_Ocular_Irritancy_Mild_vs_Moderate_Severe

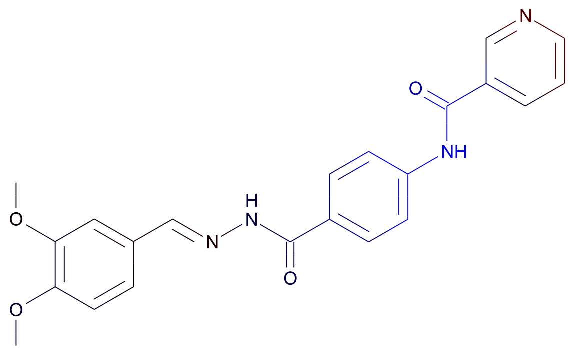

$C_{22}H_{20}N_4O_4$

Molecular Weight: 404.4186

ALogP: 2.284

Rotatable Bonds: 7

Acceptors: 6

Donors: 2

Model Prediction

Prediction: Mild

Probability: 0.604

Enrichment: 0.876

Bayesian Score: -4.96

Mahalanobis Distance: 6.31

Mahalanobis Distance p-value: 1

Prediction: Positive if the Bayesian score is above the estimated best cutoff value from minimizing the false positive and false negative rate.
Probability: The esimated probability that the sample is in the positive category. This assumes that the Bayesian score follows a normal distribution and is different from the prediction using a cutoff.
Enrichment: An estimate of enrichment, that is, the increased likelihood (versus random) of this sample being in the category.
Bayesian Score: The standard Laplacian-modified Bayesian score.
Mahalanobis Distance: The Mahalanobis distance (MD) is the distance to the center of the training data. The larger the MD, the less trustworthy the prediction.
Mahalanobis Distance p-value: The p-value gives the fraction of training data with an MD greater than or equal to the one for the given sample, assuming normally distributed data. The smaller the p-value, the less trustworthy the prediciton. For highly non-normal X properties (e.g., fingerprints), the MD p-value is wildly inaccurate.

Structural Similar Compounds

Name	1-AMINO-4-BENZOYLAMINO-ANTHRAQUINONE	ANTHRAQUINONE; 1-AMINO-4-HYDROXY-2-PHENOXY-	COLCHICINE
Structure	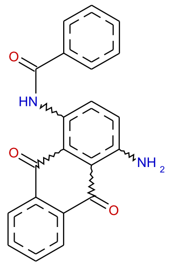	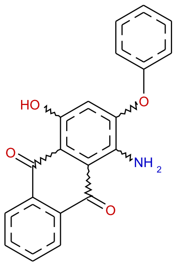	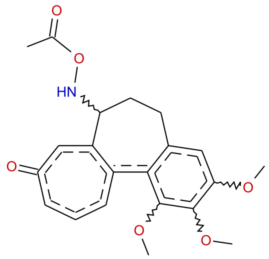
Actual Endpoint	Mild	Mild	Moderate_Severe
Predicted Endpoint	Mild	Mild	Moderate_Severe
Distance	0.708	0.709	0.711
Reference	28ZPAK-;124;72	28ZPAK 239;72	AJOPAA 31;837;48

Model Applicability

Unknown features are fingerprint features in the query molecule, but not found or appearing too infrequently in the training set.

1. All properties and OPS components are within expected ranges.
2. Unknown FCFP_2 feature: 581019816: [*]N\N=C\[*]

Feature Contribution

Top features for positive contribution				
**Fingerprint**	**Bit/Smiles**	**Feature Structure**	**Score**	**Moderate_Severe in training set**
FCFP_10	547884906	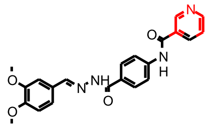 [*][c]1:[*]:[cH]:[cH]:n:[cH]:1	0.317	4 out of 4

FCFP_10	-1695756380	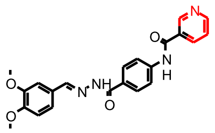 [*]1:[cH]:[cH]:[cH]:n :[cH]:1	0.285	10 out of 11
FCFP_10	-124655670	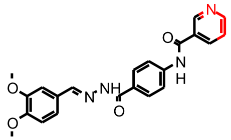 [*]:[cH]:[cH]:n:[*]	0.259	14 out of 16
**Top Features for negative contribution**				
**Fingerprint**	**Bit/Smiles**	**Feature Structure**	**Score**	**Moderate_Severe in training set**
FCFP_10	-581879738	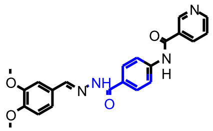 [*]NC(=O)[c]1:[cH]:[c H]:[*]:[cH]:[cH]:1	-1.29	0 out of 4
FCFP_10	-1925475824	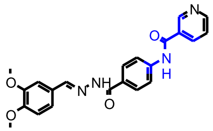 [*]:[cH]:[c](:[cH]:[*])C(=O)N[c](:[*]):[*]	-1.29	0 out of 4
FCFP_10	1175232969	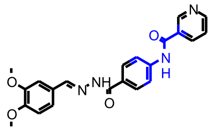 [*]:[cH]:[c](NC(=O)[c](:[*]):[*]):[cH]:[*]	-1.29	0 out of 4

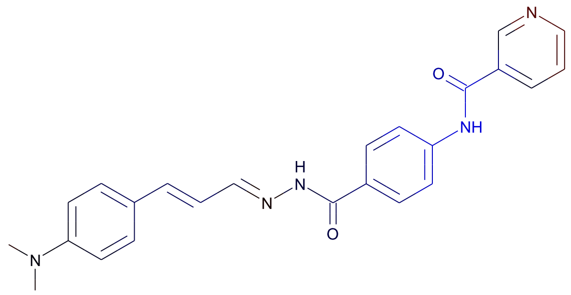

$C_{24}H_{23}N_5O_2$
Molecular Weight: 413.47172
ALogP: 2.839
Rotatable Bonds: 7
Acceptors: 5
Donors: 2

Structural Similar Compounds

Name	1-AMINO-4-BENZOYLAMINO-ANTHRAQUINONE	5-NORBORNENE-2;3-DICARBOXYLIC ACID; 1;4;5;6;7;7-HEXACHLORO-	ANTHRAQUINONE; 1-AMINO-4-HYDROXY-2-PHENOXY-
Structure	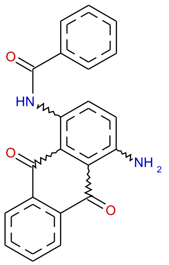	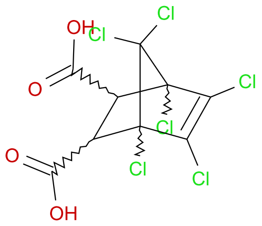	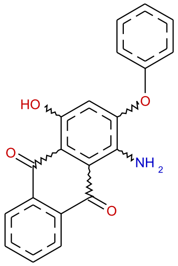
Actual Endpoint	Mild	Moderate_Severe	Mild
Predicted Endpoint	Mild	Moderate_Severe	Mild
Distance	0.680	0.683	0.708
Reference	28ZPAK-;124;72	28ZPAK-;92;72	28ZPAK 239;72

Model Prediction

Prediction: Mild
Probability: 0.546
Enrichment: 0.793
Bayesian Score: -5.66
Mahalanobis Distance: 9.87
Mahalanobis Distance p-value: 0.127

Prediction: Positive if the Bayesian score is above the estimated best cutoff value from minimizing the false positive and false negative rate.
Probability: The esimated probability that the sample is in the positive category. This assumes that the Bayesian score follows a normal distribution and is different from the prediction using a cutoff.
Enrichment: An estimate of enrichment, that is, the increased likelihood (versus random) of this sample being in the category.
Bayesian Score: The standard Laplacian-modified Bayesian score.
Mahalanobis Distance: The Mahalanobis distance (MD) is the distance to the center of the training data. The larger the MD, the less trustworthy the prediction.
Mahalanobis Distance p-value: The p-value gives the fraction of training data with an MD greater than or equal to the one for the given sample, assuming normally distributed data. The smaller the p-value, the less trustworthy the prediciton. For highly non-normal X properties (e.g., fingerprints), the MD p-value is wildly inaccurate.

Model Applicability

Unknown features are fingerprint features in the query molecule, but not found or appearing too infrequently in the training set.

1. All properties and OPS components are within expected ranges.
2. Unknown FCFP_2 feature: 581019816: [*]N\N=C\[*]

Feature Contribution

Top features for positive contribution

Fingerprint	Bit/Smiles	Feature Structure	Score	Moderate_Severe in training set
FCFP_10	547884906	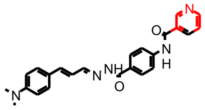 [*][c]1:[*]:[cH]:[cH]:n:[cH]:1	0.317	4 out of 4

FCFP_10	-1695756380	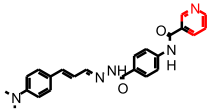 [*]1:[cH]:[cH]:[cH]:n :[cH]:1	0.285	10 out of 11
FCFP_10	-124655670	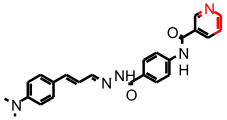 [*]:[cH]:[cH]:n:[*]	0.259	14 out of 16
**Top Features for negative contribution**				
**Fingerprint**	**Bit/Smiles**	**Feature Structure**	**Score**	**Moderate_Severe in training set**
FCFP_10	-581879738	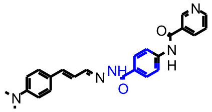 [*]NC(=O)[c]1:[cH]:[cH]:[*]:[cH]:[cH]:1	-1.29	0 out of 4
FCFP_10	1175232969	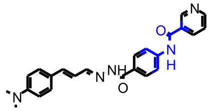 [*]:[cH]:[c](NC(=O)[c](:[*]):[*]):[cH]:[*]	-1.29	0 out of 4
FCFP_10	-1925475824	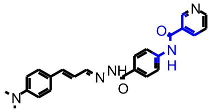 [*]:[cH]:[c](:[cH]:[*])C(=O)N[c](:[*]):[*]	-1.29	0 out of 4

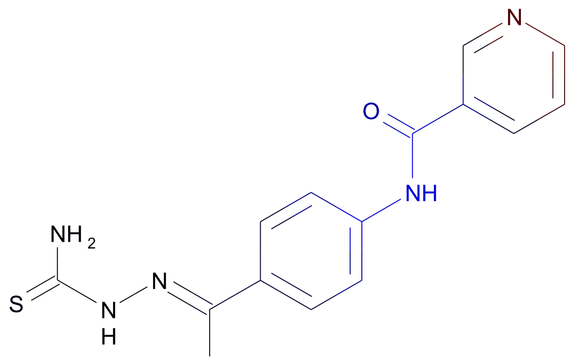

$C_{15}H_{15}N_5OS$
Molecular Weight: 313.3775
ALogP: 1.414
Rotatable Bonds: 5
Acceptors: 4
Donors: 3

Model Prediction

Prediction: Mild
Probability: 0.702
Enrichment: 1.02
Bayesian Score: -3.49
Mahalanobis Distance: 6.48
Mahalanobis Distance p-value: 1

Prediction: Positive if the Bayesian score is above the estimated best cutoff value from minimizing the false positive and false negative rate.
Probability: The esimated probability that the sample is in the positive category. This assumes that the Bayesian score follows a normal distribution and is different from the prediction using a cutoff.
Enrichment: An estimate of enrichment, that is, the increased likelihood (versus random) of this sample being in the category.
Bayesian Score: The standard Laplacian-modified Bayesian score.
Mahalanobis Distance: The Mahalanobis distance (MD) is the distance to the center of the training data. The larger the MD, the less trustworthy the prediction.
Mahalanobis Distance p-value: The p-value gives the fraction of training data with an MD greater than or equal to the one for the given sample, assuming normally distributed data. The smaller the p-value, the less trustworthy the prediciton. For highly non-normal X properties (e.g., fingerprints), the MD p-value is wildly inaccurate.

Structural Similar Compounds

Name	2-NAPHTHALENESULFONIC ACID; 4-HYDROXY-7-(METHYLAMINO)-	ANTHRAQUINONE; 1-((2-HYDROXYETHYL)AMINO)-4-(METHYLAMINO)-	O-TOLUENESULFONAMIDE; 4-AMINO-N-(2-HYDROXYETHYL)-
Structure	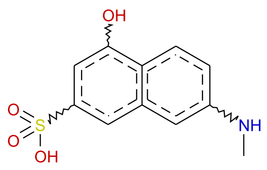	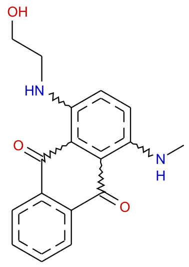	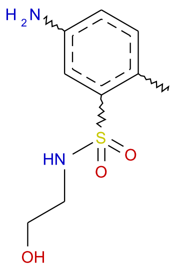
Actual Endpoint	Mild	Mild	Moderate_Severe
Predicted Endpoint	Mild	Mild	Moderate_Severe
Distance	0.606	0.637	0.656
Reference	28ZPAK 190;72	28ZPAK 245;72	28ZPAK-;200;72

Model Applicability

Unknown features are fingerprint features in the query molecule, but not found or appearing too infrequently in the training set.

1. All properties and OPS components are within expected ranges.
2. Unknown FCFP_2 feature: 581019816: [*]N\N=C\[*]

Feature Contribution

Top features for positive contribution				
**Fingerprint**	**Bit/Smiles**	**Feature Structure**	**Score**	**Moderate_Severe in training set**
FCFP_10	547884906	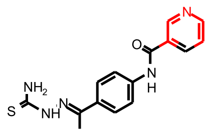 [*][c]1:[*]:[cH]:[cH]:n:[cH]:1	0.317	4 out of 4

FCFP_10	-1695756380	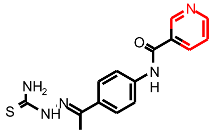 [*]1:[cH]:[cH]:[cH]:n :[cH]:1	0.285	10 out of 11
FCFP_10	-124655670	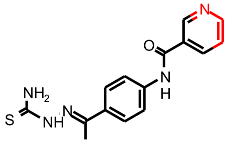 [*]:[cH]:[cH]:n:[*]	0.259	14 out of 16
**Top Features for negative contribution**				
**Fingerprint**	**Bit/Smiles**	**Feature Structure**	**Score**	**Moderate_Severe in training set**
FCFP_10	1175232969	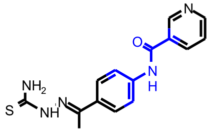 [*]:[cH]:[c](NC(=O)[c](:[*]):[*]):[cH]:[*]	-1.29	0 out of 4
FCFP_10	-1925475824	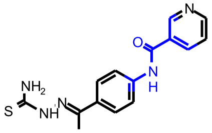 [*]:[cH]:[c](:[cH]:[*])C(=O)N[c](:[*]):[*]	-1.29	0 out of 4
FCFP_10	-790336137	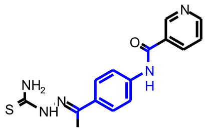 [*]C(=[*])N[c]1:[cH]: [cH]:[c](:[cH]:[cH]: 1)C(=[*])[*]	-0.507	0 out of 1

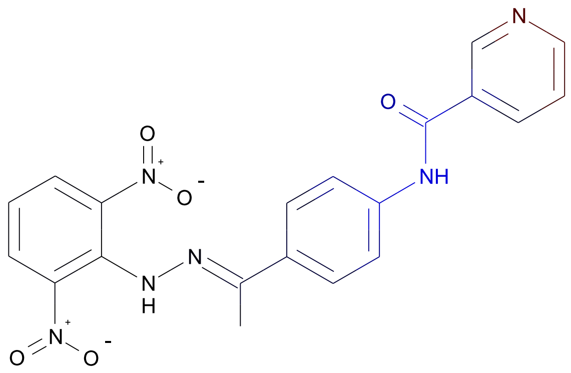

$C_{20}H_{16}N_6O_5$
Molecular Weight: 420.37824
ALogP: 2.852
Rotatable Bonds: 7
Acceptors: 8
Donors: 2

Structural Similar Compounds

Name	4;4'-DIAMINO-1;1'-DIANTHRIMIDE	2;2'-Stilbenedisulfonic acid; 4;4'-dinitro-	COLCHICINE
Structure	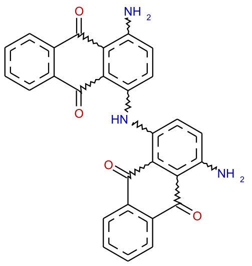	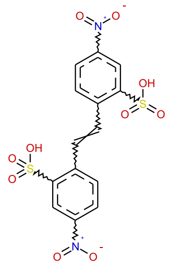	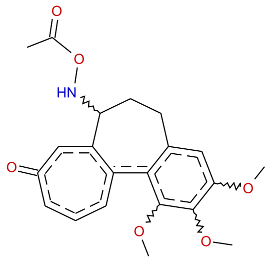
Actual Endpoint	Mild	Mild	Moderate_Severe
Predicted Endpoint	Mild	Mild	Moderate_Severe
Distance	0.774	0.797	0.826
Reference	28ZPAK-;125;72	Prehled Prumyslove Toxikologie; Organicke Latky; Marhold; J. pp 1062;86	AJOPAA 31;837;48

Model Prediction

Prediction: Mild
Probability: 0.692
Enrichment: 1
Bayesian Score: -3.66
Mahalanobis Distance: 5.51
Mahalanobis Distance p-value: 1

Prediction: Positive if the Bayesian score is above the estimated best cutoff value from minimizing the false positive and false negative rate.
Probability: The esimated probability that the sample is in the positive category. This assumes that the Bayesian score follows a normal distribution and is different from the prediction using a cutoff.
Enrichment: An estimate of enrichment, that is, the increased likelihood (versus random) of this sample being in the category.
Bayesian Score: The standard Laplacian-modified Bayesian score.
Mahalanobis Distance: The Mahalanobis distance (MD) is the distance to the center of the training data. The larger the MD, the less trustworthy the prediction.
Mahalanobis Distance p-value: The p-value gives the fraction of training data with an MD greater than or equal to the one for the given sample, assuming normally distributed data. The smaller the p-value, the less trustworthy the prediciton. For highly non-normal X properties (e.g., fingerprints), the MD p-value is wildly inaccurate.

Model Applicability

Unknown features are fingerprint features in the query molecule, but not found or appearing too infrequently in the training set.

1. All properties and OPS components are within expected ranges.
2. Unknown FCFP_2 feature: 581019816: [*]N\N=C\[*]
3. Unknown FCFP_2 feature: 1294285001: [*]=NN[c](:[*]):[*]
4. Unknown FCFP_2 feature: -828984032: [*][c](:[*]):[c]([N+](=[*])[*]):c:[*]
5. Unknown FCFP_2 feature: -1338588315: [*]:[c](:[*])[N+](=O)[O-]
6. Unknown FCFP_2 feature: 1872392852: [*][N+](=O)[*]
7. Unknown FCFP_2 feature: 260476081: [*][N+](=[*])[O-]

Feature Contribution

Top features for positive contribution

Fingerprint	Bit/Smiles	Feature Structure	Score	Moderate_Severe in training set

FCFP_10	547884906	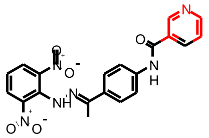 [*][c]1:[*]:[cH]:[cH]:n:[cH]:1	0.317	4 out of 4
FCFP_10	-1695756380	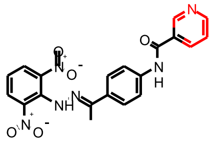 [*]1:[cH]:[cH]:[cH]:n:[cH]:1	0.285	10 out of 11
FCFP_10	-124655670	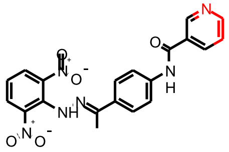 [*]:[cH]:[cH]:n:[*]	0.259	14 out of 16
**Top Features for negative contribution**				
**Fingerprint**	**Bit/Smiles**	**Feature Structure**	**Score**	**Moderate_Severe in training set**
FCFP_10	1175232969	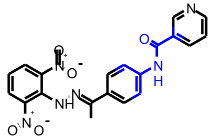 [*]:[cH]:[c](NC(=O)[c](:[*]):[*]):[cH]:[*]	-1.29	0 out of 4
FCFP_10	-1925475824	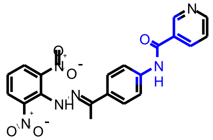 [*]:[cH]:[c](:[cH]:[*])C(=O)N[c](:[*]):[*]	-1.29	0 out of 4

FCFP_10	-790336137	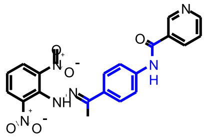 [*]C(=[*])N[c]1:[cH]:[cH]:[c](:[cH]:[cH]:1)C(=[*])[*]	-0.507	0 out of 1

Sorafenib

TOPKAT_Ocular_Irritancy_Mild_vs_Moderate_Severe

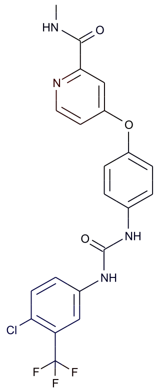

$C_{21}H_{16}ClF_3N_4O_3$
Molecular Weight: 464.82494
ALogP: 4.175
Rotatable Bonds: 6
Acceptors: 4
Donors: 3

Structural Similar Compounds

Name	4;4'-DIAMINO-1;1'-DIANTHRIMIDE	5-NORBORNENE-2;3-DICARBOXYLIC ACID; 1;4;5;6;7;7-HEXACHLORO-	METHANE;TRIS(4-AMINOPHENYL)-
Structure	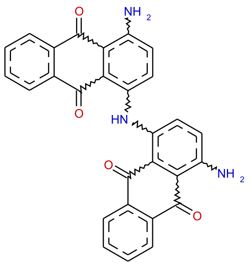	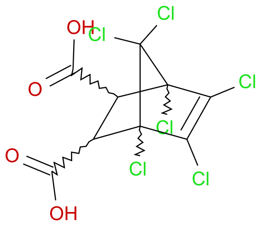	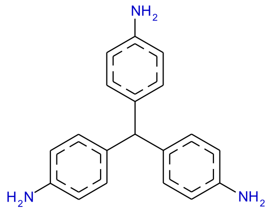
Actual Endpoint	Mild	Moderate_Severe	Moderate_Severe
Predicted Endpoint	Mild	Moderate_Severe	Moderate_Severe
Distance	0.799	0.816	0.827
Reference	28ZPAK-;125;72	28ZPAK-;92;72	28ZPAK-;73;72

Model Prediction

Prediction: Mild
Probability: 0.776
Enrichment: 1.13
Bayesian Score: -1.8
Mahalanobis Distance: 8.95
Mahalanobis Distance p-value: 0.537

Prediction: Positive if the Bayesian score is above the estimated best cutoff value from minimizing the false positive and false negative rate.
Probability: The esimated probability that the sample is in the positive category. This assumes that the Bayesian score follows a normal distribution and is different from the prediction using a cutoff.
Enrichment: An estimate of enrichment, that is, the increased likelihood (versus random) of this sample being in the category.
Bayesian Score: The standard Laplacian-modified Bayesian score.
Mahalanobis Distance: The Mahalanobis distance (MD) is the distance to the center of the training data. The larger the MD, the less trustworthy the prediction.
Mahalanobis Distance p-value: The p-value gives the fraction of training data with an MD greater than or equal to the one for the given sample, assuming normally distributed data. The smaller the p-value, the less trustworthy the prediciton. For highly non-normal X properties (e.g., fingerprints), the MD p-value is wildly inaccurate.

Model Applicability

Unknown features are fingerprint features in the query molecule, but not found or appearing too infrequently in the training set.

1. All properties and OPS components are within expected ranges.

Feature Contribution

Top features for positive contribution				
**Fingerprint**	**Bit/Smiles**	**Feature Structure**	**Score**	**Moderate_Severe in training set**
FCFP_10	-1695756380	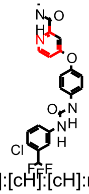 [*]1:[cH]:[cH]:[cH]:n:[cH]:1	0.285	10 out of 11

FCFP_10	-124655670	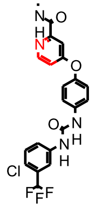 [*]:[cH]:[cH]:n:[*]	0.259	14 out of 16
FCFP_10	-885550502	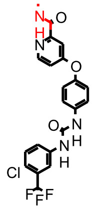 [*]C(=[*])NC	0.239	54 out of 64

Top Features for negative contribution

Fingerprint	Bit/Smiles	Feature Structure	Score	Moderate_Severe in training set
FCFP_10	2104062943	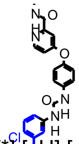 [*][c]1:[*]:[cH]:[c](C=[*]):[c](Cl):[cH]:1	-0.745	7 out of 24
FCFP_10	-174293376	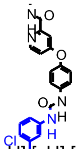 [*]N[c]1:[cH]:[cH]:[c](Cl):[c](:[cH]:1)C([*])([*])[*]	-0.507	0 out of 1
FCFP_10	-1549103449	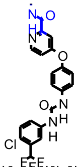 [*]NC(=O)[c](:[*]):[*]	-0.504	2 out of 6

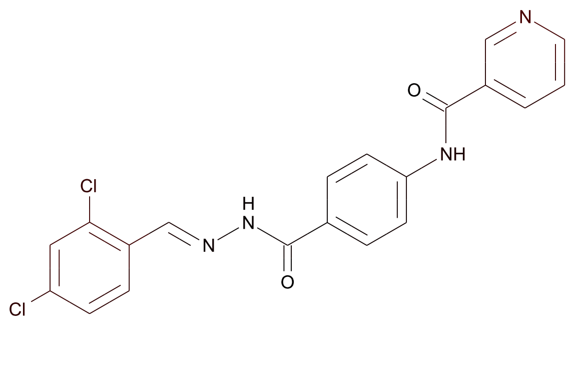

$C_{20}H_{14}Cl_2N_4O_2$
Molecular Weight: 413.25676
ALogP: 3.646
Rotatable Bonds: 5
Acceptors: 4
Donors: 2

Model Prediction

Prediction: Irritant
Probability: 1
Enrichment: 1.18
Bayesian Score: 3.46
Mahalanobis Distance: 4.71
Mahalanobis Distance p-value: 1

Prediction: Positive if the Bayesian score is above the estimated best cutoff value from minimizing the false positive and false negative rate.
Probability: The esimated probability that the sample is in the positive category. This assumes that the Bayesian score follows a normal distribution and is different from the prediction using a cutoff.
Enrichment: An estimate of enrichment, that is, the increased likelihood (versus random) of this sample being in the category.
Bayesian Score: The standard Laplacian-modified Bayesian score.
Mahalanobis Distance: The Mahalanobis distance (MD) is the distance to the center of the training data. The larger the MD, the less trustworthy the prediction.
Mahalanobis Distance p-value: The p-value gives the fraction of training data with an MD greater than or equal to the one for the given sample, assuming normally distributed data. The smaller the p-value, the less trustworthy the prediciton. For highly non-normal X properties (e.g., fingerprints), the MD p-value is wildly inaccurate.

Structural Similar Compounds

Name	1-AMINO-4-BENZOYLAMINO-ANTHRAQUINONE	5-NORBORNENE-2;3-DICARBOXYLIC ACID; 1;4;5;6;7;7-HEXACHLORO-	1-BENZOYLAMINO-4-METHOXY-5-CHLORANTHRAQUINONE
Structure	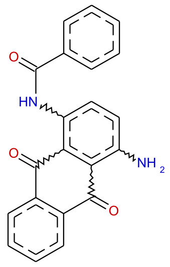	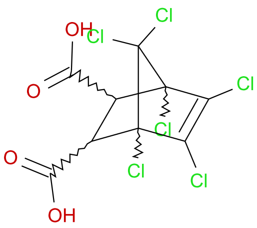	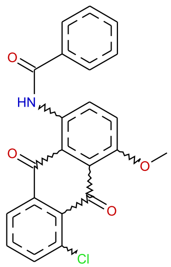
Actual Endpoint	Irritant	Irritant	Irritant
Predicted Endpoint	Irritant	Irritant	Irritant
Distance	0.623	0.627	0.677
Reference	28ZPAK-;124;72	28ZPAK-;92;72	28ZPAK-;90;72

Model Applicability

Unknown features are fingerprint features in the query molecule, but not found or appearing too infrequently in the training set.

1. All properties and OPS components are within expected ranges.
2. Unknown FCFP_2 feature: 581019816: [*]N\N=C\[*]

Feature Contribution

Top features for positive contribution				
**Fingerprint**	**Bit/Smiles**	**Feature Structure**	**Score**	**Irritant in training set**
FCFP_12	1747237384	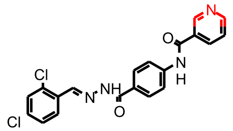 [*]:[cH]:n:[cH]:[*]	0.208	44 out of 44

FCFP_12	-124655670	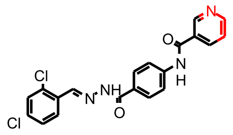 [*]:[cH]:[cH]:n:[*]	0.2	16 out of 16
FCFP_12	555188808	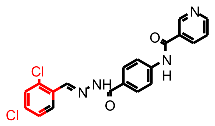 [*][c]1:[*]:[cH]:[c](Cl):[cH]:[c]:1Cl	0.195	12 out of 12
**Top Features for negative contribution**				
**Fingerprint**	**Bit/Smiles**	**Feature Structure**	**Score**	**Irritant in training set**
FCFP_12	0	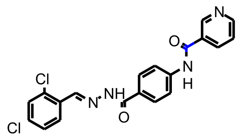 [*]C(=[*])[*]	0	1184 out of 1397
FCFP_12	1	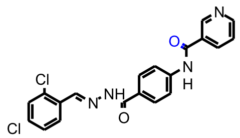 [*]=O	0	872 out of 1051
FCFP_12	203677720	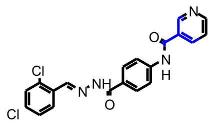 [*]C(=[*])[c](:[cH]:[*]):[cH]:[*]	0	319 out of 382

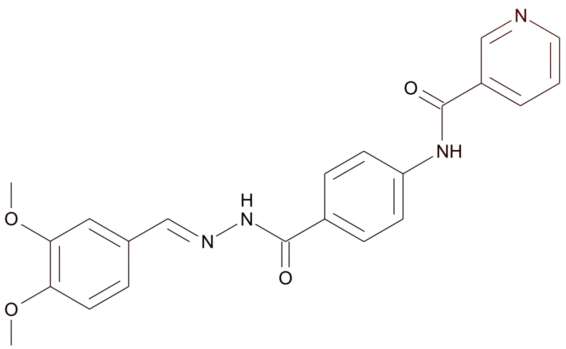

$C_{22}H_{20}N_4O_4$
Molecular Weight: 404.4186
ALogP: 2.284
Rotatable Bonds: 7
Acceptors: 6
Donors: 2

Structural Similar Compounds

Name	ANTHRAQUINONE; 1-AMINO-4-HYDROXY-2-PHENOXY-	1-AMINO-4-BENZOYLAMINO-ANTHRAQUINONE	2;2';-Dihydroxy-4;4'-dimethoxybenzophenone
Structure	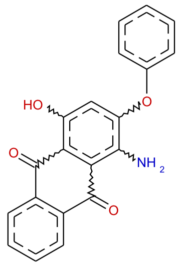	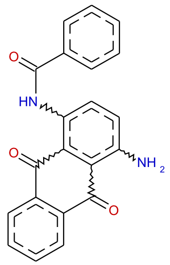	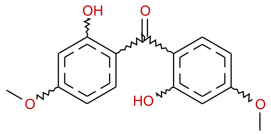
Actual Endpoint	Irritant	Irritant	Non-Irritant
Predicted Endpoint	Irritant	Irritant	Non-Irritant
Distance	0.694	0.699	0.699
Reference	28ZPAK 239;72	28ZPAK-;124;72	J. Am. Coll. Toxicol. 2(5):35;1983

Model Prediction

Prediction: Irritant
Probability: 1
Enrichment: 1.18
Bayesian Score: 2.61
Mahalanobis Distance: 5.78
Mahalanobis Distance p-value: 1

Prediction: Positive if the Bayesian score is above the estimated best cutoff value from minimizing the false positive and false negative rate.
Probability: The esimated probability that the sample is in the positive category. This assumes that the Bayesian score follows a normal distribution and is different from the prediction using a cutoff.
Enrichment: An estimate of enrichment, that is, the increased likelihood (versus random) of this sample being in the category.
Bayesian Score: The standard Laplacian-modified Bayesian score.
Mahalanobis Distance: The Mahalanobis distance (MD) is the distance to the center of the training data. The larger the MD, the less trustworthy the prediction.
Mahalanobis Distance p-value: The p-value gives the fraction of training data with an MD greater than or equal to the one for the given sample, assuming normally distributed data. The smaller the p-value, the less trustworthy the prediciton. For highly non-normal X properties (e.g., fingerprints), the MD p-value is wildly inaccurate.

Model Applicability

Unknown features are fingerprint features in the query molecule, but not found or appearing too infrequently in the training set.

1. All properties and OPS components are within expected ranges.
2. Unknown FCFP_2 feature: 581019816: [*]N\N=C\[*]

Feature Contribution

Top features for positive contribution

Fingerprint	Bit/Smiles	Feature Structure	Score	Irritant in training set
FCFP_12	1747237384	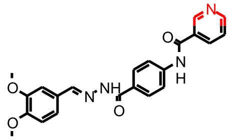 [*]:[cH]:n:[cH]:[*]	0.208	44 out of 44

FCFP_12	-124655670	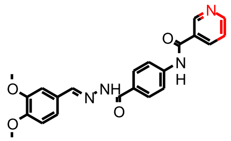<br>[*]:[cH]:[cH]:n:[*]	0.2	16 out of 16
FCFP_12	-1695756380	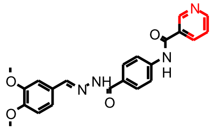<br>[*]1:[cH]:[cH]:[cH]:n:[cH]:1	0.194	11 out of 11
**Top Features for negative contribution**				
**Fingerprint**	**Bit/Smiles**	**Feature Structure**	**Score**	**Irritant in training set**
FCFP_12	203677720	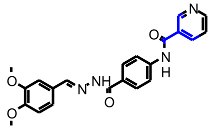<br>[*]C(=[*])[c](:[cH]:[*]):[cH]:[*]	0	319 out of 382
FCFP_12	-1925475824	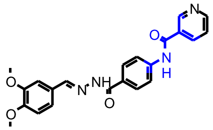<br>[*]:[cH]:[c](:[cH]:[*])C(=O)N[c](:[*]):[*]	0	4 out of 5
FCFP_12	1175232969	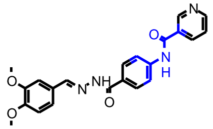<br>[*]:[cH]:[c](NC(=O)[c](:[*]):[*]):[cH]:[*]	0	4 out of 5

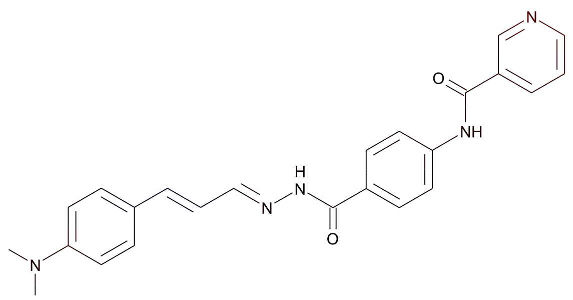

$C_{24}H_{23}N_5O_2$

Molecular Weight: 413.47172

ALogP: 2.839

Rotatable Bonds: 7

Acceptors: 5

Donors: 2

Structural Similar Compounds

Name	5-NORBORNENE-2;3-DICARBOXYLIC ACID; 1;4;5;6;7;7-HEXACHLORO-	1-AMINO-4-BENZOYLAMINO-ANTHRAQUINONE	ANTHRAQUINONE; 1-AMINO-4-HYDROXY-2-PHENOXY-
Structure	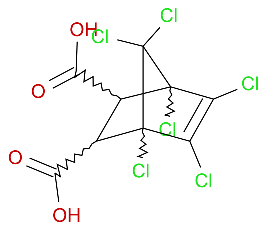	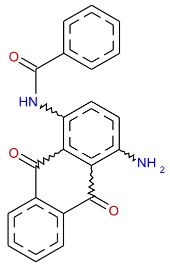	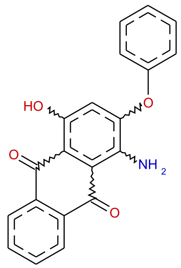
Actual Endpoint	Irritant	Irritant	Irritant
Predicted Endpoint	Irritant	Irritant	Irritant
Distance	0.671	0.674	0.699
Reference	28ZPAK-;92;72	28ZPAK-;124;72	28ZPAK 239;72

Model Prediction

Prediction: Irritant

Probability: 1

Enrichment: 1.18

Bayesian Score: 1.91

Mahalanobis Distance: 7.32

Mahalanobis Distance p-value: 0.993

Prediction: Positive if the Bayesian score is above the estimated best cutoff value from minimizing the false positive and false negative rate.
Probability: The esimated probability that the sample is in the positive category. This assumes that the Bayesian score follows a normal distribution and is different from the prediction using a cutoff.
Enrichment: An estimate of enrichment, that is, the increased likelihood (versus random) of this sample being in the category.
Bayesian Score: The standard Laplacian-modified Bayesian score.
Mahalanobis Distance: The Mahalanobis distance (MD) is the distance to the center of the training data. The larger the MD, the less trustworthy the prediction.
Mahalanobis Distance p-value: The p-value gives the fraction of training data with an MD greater than or equal to the one for the given sample, assuming normally distributed data. The smaller the p-value, the less trustworthy the prediciton. For highly non-normal X properties (e.g., fingerprints), the MD p-value is wildly inaccurate.

Model Applicability

Unknown features are fingerprint features in the query molecule, but not found or appearing too infrequently in the training set.

1. All properties and OPS components are within expected ranges.
2. Unknown FCFP_2 feature: 581019816: [*]N\N=C\[*]

Feature Contribution

Top features for positive contribution				
**Fingerprint**	**Bit/Smiles**	**Feature Structure**	**Score**	**Irritant in training set**
FCFP_12	1747237384	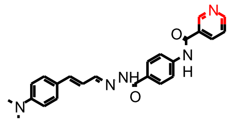 [*]:[cH]:n:[cH]:[*]	0.208	44 out of 44

FCFP_12	-124655670	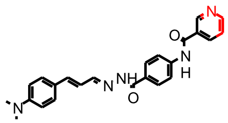 [*]:[cH]:[cH]:n:[*]	0.2	16 out of 16
FCFP_12	-1695756380	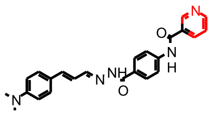 [*]1:[cH]:[cH]:[cH]:n:[cH]:1	0.194	11 out of 11
**Top Features for negative contribution**				
**Fingerprint**	**Bit/Smiles**	**Feature Structure**	**Score**	**Irritant in training set**
FCFP_12	-1078052987	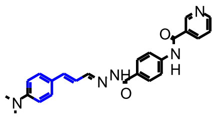 [*]\C=C\[c]1:[cH]:[cH]:[*]:[cH]:[cH]:1	-0.344	2 out of 4
FCFP_12	-146015125	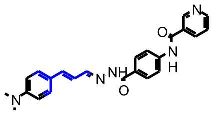 [*]=C\C=C\[c](:[cH]:[*]):[cH]:[*]	-0.268	1 out of 2
FCFP_12	451371068	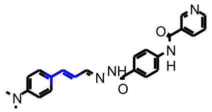 [*]\C=C\[c](:[*]):[*]	-0.167	6 out of 9

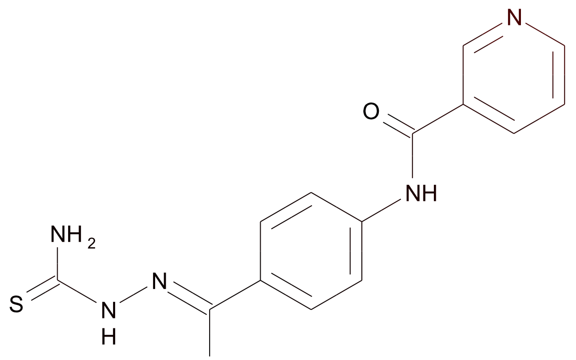

$C_{15}H_{15}N_5OS$
Molecular Weight: 313.3775
ALogP: 1.414
Rotatable Bonds: 5
Acceptors: 4
Donors: 3

Model Prediction

Prediction: Irritant
Probability: 1
Enrichment: 1.18
Bayesian Score: 2.19
Mahalanobis Distance: 5.99
Mahalanobis Distance p-value: 1

Prediction: Positive if the Bayesian score is above the estimated best cutoff value from minimizing the false positive and false negative rate.
Probability: The esimated probability that the sample is in the positive category. This assumes that the Bayesian score follows a normal distribution and is different from the prediction using a cutoff.
Enrichment: An estimate of enrichment, that is, the increased likelihood (versus random) of this sample being in the category.
Bayesian Score: The standard Laplacian-modified Bayesian score.
Mahalanobis Distance: The Mahalanobis distance (MD) is the distance to the center of the training data. The larger the MD, the less trustworthy the prediction.
Mahalanobis Distance p-value: The p-value gives the fraction of training data with an MD greater than or equal to the one for the given sample, assuming normally distributed data. The smaller the p-value, the less trustworthy the prediciton. For highly non-normal X properties (e.g., fingerprints), the MD p-value is wildly inaccurate.

Structural Similar Compounds

Name	2-NAPHTHALENESULFONIC ACID; 4-HYDROXY-7-(METHYLAMINO)-	ANTHRAQUINONE; 1-((2-HYDROXYETHYL)AMINO)-4-(METHYLAMINO)-	O-TOLUENESULFONAMIDE; 4-AMINO-N-(2-HYDROXYETHYL)-
Structure	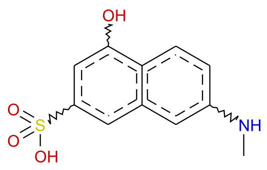	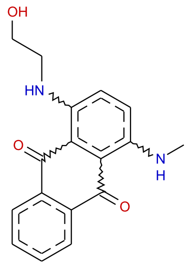	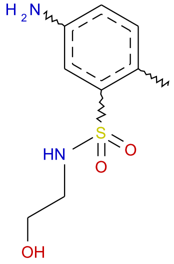
Actual Endpoint	Irritant	Irritant	Irritant
Predicted Endpoint	Irritant	Irritant	Irritant
Distance	0.599	0.638	0.641
Reference	28ZPAK 190;72	28ZPAK 245;72	28ZPAK-;200;72

Model Applicability

Unknown features are fingerprint features in the query molecule, but not found or appearing too infrequently in the training set.

1. All properties and OPS components are within expected ranges.
2. Unknown FCFP_2 feature: 581019816: [*]N\N=C\[*]

Feature Contribution

Top features for positive contribution				
**Fingerprint**	**Bit/Smiles**	**Feature Structure**	**Score**	**Irritant in training set**
FCFP_12	1747237384	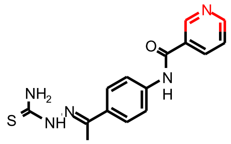 [*]:[cH]:n:[cH]:[*]	0.208	44 out of 44

FCFP_12	-124655670	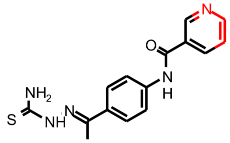 [*]:[cH]:[cH]:n:[*]	0.2	16 out of 16
FCFP_12	-1695756380	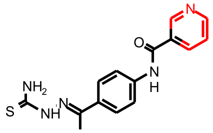 [*]1:[cH]:[cH]:[cH]:n :[cH]:1	0.194	11 out of 11

Top Features for negative contribution

Fingerprint	Bit/Smiles	Feature Structure	Score	Irritant in training set
FCFP_12	-581162801	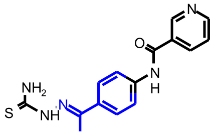 [*]\N=C(/C)\[c]1:[cH] :[cH]:[*]:[cH]:[cH]: 1	0	7 out of 9
FCFP_12	-773983804	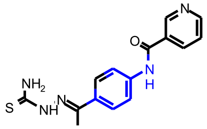 [*]N[c]1:[cH]:[*]:[c] ([*]):[cH]:[cH]:1	0	102 out of 121
FCFP_12	1175232969	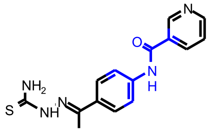 [*]:[cH]:[c](NC(=O)[c](:[*]):[*]):[cH]:[*]	0	4 out of 5

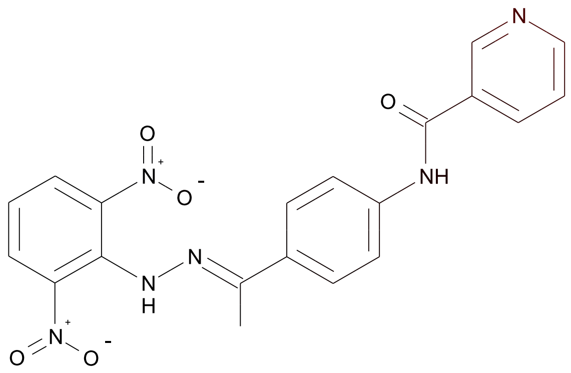

$C_{20}H_{16}N_6O_5$
Molecular Weight: 420.37824
ALogP: 2.852
Rotatable Bonds: 7
Acceptors: 8
Donors: 2

Structural Similar Compounds

Name	4;4'-DIAMINO-1;1'-DIANTHRIMIDE	2;2'-Stilbenedisulfonic acid; 4;4'-dinitro-	ANTHRAQUINONE; 1-AMINO-4-HYDROXY-2-PHENOXY-
Structure	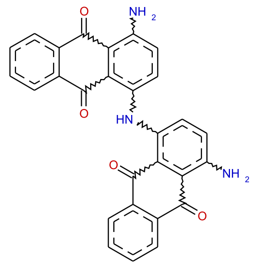	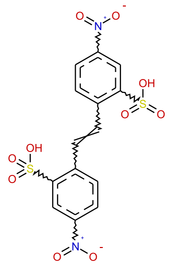	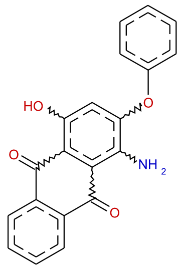
Actual Endpoint	Irritant	Irritant	Irritant
Predicted Endpoint	Irritant	Irritant	Irritant
Distance	0.767	0.797	0.822
Reference	28ZPAK-;125;72	Prehled Prumyslove Toxikologie; Organicke Latky; Marhold; J. pp 1062;86	28ZPAK 239;72

Model Prediction

Prediction: Irritant
Probability: 1
Enrichment: 1.18
Bayesian Score: 1.86
Mahalanobis Distance: 5.27
Mahalanobis Distance p-value: 1

Prediction: Positive if the Bayesian score is above the estimated best cutoff value from minimizing the false positive and false negative rate.
Probability: The esimated probability that the sample is in the positive category. This assumes that the Bayesian score follows a normal distribution and is different from the prediction using a cutoff.
Enrichment: An estimate of enrichment, that is, the increased likelihood (versus random) of this sample being in the category.
Bayesian Score: The standard Laplacian-modified Bayesian score.
Mahalanobis Distance: The Mahalanobis distance (MD) is the distance to the center of the training data. The larger the MD, the less trustworthy the prediction.
Mahalanobis Distance p-value: The p-value gives the fraction of training data with an MD greater than or equal to the one for the given sample, assuming normally distributed data. The smaller the p-value, the less trustworthy the prediciton. For highly non-normal X properties (e.g., fingerprints), the MD p-value is wildly inaccurate.

Model Applicability

Unknown features are fingerprint features in the query molecule, but not found or appearing too infrequently in the training set.

1. All properties and OPS components are within expected ranges.
2. Unknown FCFP_2 feature: 581019816: [*]N\N=C\[*]
3. Unknown FCFP_2 feature: 1294285001: [*]=NN[c](:[*]):[*]
4. Unknown FCFP_2 feature: -828984032: [*][c](:[*]):[c]([N+](=[*])[*]):c:[*]
5. Unknown FCFP_2 feature: -1338588315: [*]:[c](:[*])[N+](=O)[O-]
6. Unknown FCFP_2 feature: 1872392852: [*][N+](=O)[*]
7. Unknown FCFP_2 feature: 260476081: [*][N+](=[*])[O-]

Feature Contribution

Top features for positive contribution

Fingerprint	Bit/Smiles	Feature Structure	Score	Irritant in training set

FCFP_12	1747237384	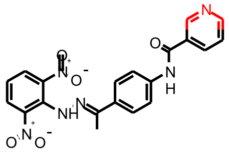 [*]:[cH]:n:[cH]:[*]	0.208	44 out of 44
FCFP_12	-124655670	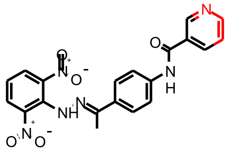 [*]:[cH]:[cH]:n:[*]	0.2	16 out of 16
FCFP_12	-1695756380	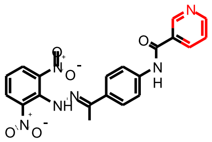 [*]1:[cH]:[cH]:[cH]:n :[cH]:1	0.194	11 out of 11
**Top Features for negative contribution**				
**Fingerprint**	**Bit/Smiles**	**Feature Structure**	**Score**	**Irritant in training set**
FCFP_12	8	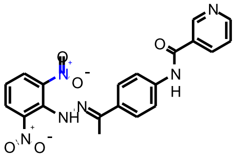 [*][N+](=[*])[*]	-0.0561	3 out of 4
FCFP_12	991735244	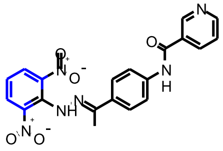 [*][c]1:[*]:[c]([*]): [cH]:[cH]:[cH]:1	0	237 out of 291

FCFP_12	-581162801	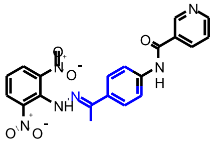 [*]\N=C(/C)\[c]1:[cH]:[cH]:[*]:[cH]:[cH]:1	0	7 out of 9

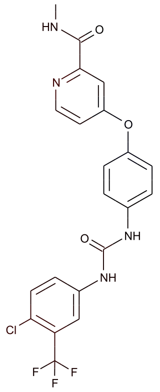

$C_{21}H_{16}ClF_{3}N_{4}O_{3}$
Molecular Weight: 464.82494
ALogP: 4.175
Rotatable Bonds: 6
Acceptors: 4
Donors: 3

Structural Similar Compounds

Name	BENZANILIDE;2';2'''-DITHIOBIS-	4;4'-DIAMINO-1;1'-DIANTHRIMIDE	5-NORBORNENE-2;3-DICARBOXYLIC ACID; 1;4;5;6;7;7-HEXACHLORO-
Structure	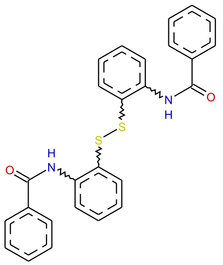	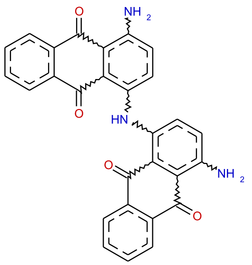	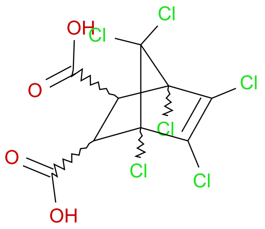
Actual Endpoint	Non-Irritant	Irritant	Irritant
Predicted Endpoint	Non-Irritant	Irritant	Irritant
Distance	0.743	0.791	0.801
Reference	28ZPAK-;173;72	28ZPAK-;125;72	28ZPAK-;92;72

Model Prediction

Prediction: Irritant
Probability: 1
Enrichment: 1.18
Bayesian Score: 3.04
Mahalanobis Distance: 6.28
Mahalanobis Distance p-value: 1

Prediction: Positive if the Bayesian score is above the estimated best cutoff value from minimizing the false positive and false negative rate.
Probability: The esimated probability that the sample is in the positive category. This assumes that the Bayesian score follows a normal distribution and is different from the prediction using a cutoff.
Enrichment: An estimate of enrichment, that is, the increased likelihood (versus random) of this sample being in the category.
Bayesian Score: The standard Laplacian-modified Bayesian score.
Mahalanobis Distance: The Mahalanobis distance (MD) is the distance to the center of the training data. The larger the MD, the less trustworthy the prediction.
Mahalanobis Distance p-value: The p-value gives the fraction of training data with an MD greater than or equal to the one for the given sample, assuming normally distributed data. The smaller the p-value, the less trustworthy the prediciton. For highly non-normal X properties (e.g., fingerprints), the MD p-value is wildly inaccurate.

Model Applicability

Unknown features are fingerprint features in the query molecule, but not found or appearing too infrequently in the training set.

1. All properties and OPS components are within expected ranges.

Feature Contribution

Top features for positive contribution

Fingerprint	Bit/Smiles	Feature Structure	Score	Irritant in training set
FCFP_12	1747237384	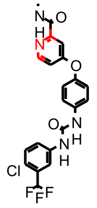 [*]:[cH]:n:[cH]:[*]	0.208	44 out of 44

FCFP_12	-124655670	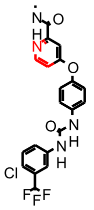 [*]:[cH]:[cH]:n:[*]	0.2	16 out of 16
FCFP_12	-1539132615	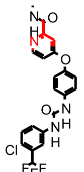 [*]C(=[*])[c](:[cH]:[*]):n:[*]	0.197	13 out of 13

Top Features for negative contribution

**Fingerprint**	**Bit/Smiles**	**Feature Structure**	**Score**	**Irritant in training set**
FCFP_12	-747629521	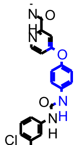 [*]N[c]1:[cH]:[cH]:[c](O[c](:[*]):[*]):[cH]:[cH]:1	-0.268	1 out of 2
FCFP_12	702861189	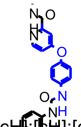 [*]N[c]1:[cH]:[cH]:[c](O[c]2:[cH]:[cH]:[*]:[c]([*]):[cH]:2):[cH]:[cH]:1	-0.268	1 out of 2
FCFP_12	859018953	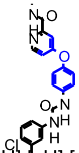 [*][c]1:[cH]:[cH]:[c](O[c](:[cH]:[*]):[cH]:[*]):[cH]:[cH]:1	0	7 out of 9

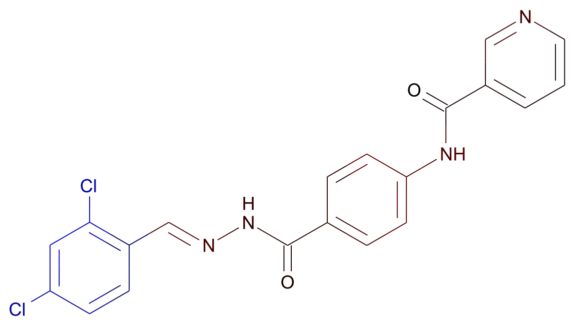

$C_{20}H_{14}Cl_2N_4O_2$
Molecular Weight: 413.25676
ALogP: 3.646
Rotatable Bonds: 5
Acceptors: 4
Donors: 2

Model Prediction

Prediction: Carcinogen
Probability: 0.291
Enrichment: 0.903
Bayesian Score: -0.399
Mahalanobis Distance: 12.8
Mahalanobis Distance p-value: 0.000265

Prediction: Positive if the Bayesian score is above the estimated best cutoff value from minimizing the false positive and false negative rate.
Probability: The esimated probability that the sample is in the positive category. This assumes that the Bayesian score follows a normal distribution and is different from the prediction using a cutoff.
Enrichment: An estimate of enrichment, that is, the increased likelihood (versus random) of this sample being in the category.
Bayesian Score: The standard Laplacian-modified Bayesian score.
Mahalanobis Distance: The Mahalanobis distance (MD) is the distance to the center of the training data. The larger the MD, the less trustworthy the prediction.
Mahalanobis Distance p-value: The p-value gives the fraction of training data with an MD greater than or equal to the one for the given sample, assuming normally distributed data. The smaller the p-value, the less trustworthy the prediciton. For highly non-normal X properties (e.g., fingerprints), the MD p-value is wildly inaccurate.

Structural Similar Compounds

Name	Bicalutamide	Doxefazepam	Indomethacin
Structure	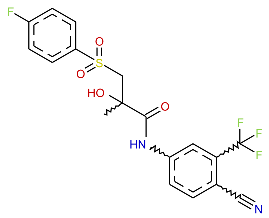	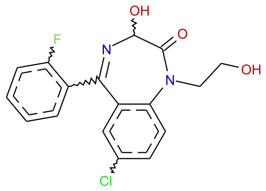	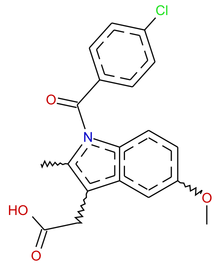
Actual Endpoint	Carcinogen	Carcinogen	Non-Carcinogen
Predicted Endpoint	Carcinogen	Carcinogen	Non-Carcinogen
Distance	0.627	0.652	0.654
Reference	US FDA (Centre for Drug Eval.& Res./Off. Testing & Res.) Sept. 1997	US FDA (Centre for Drug Eval.& Res./Off. Testing & Res.) Sept. 1997	US FDA (Centre for Drug Eval.& Res./Off. Testing & Res.) Sept. 1997

Model Applicability

Unknown features are fingerprint features in the query molecule, but not found or appearing too infrequently in the training set.

1. All properties and OPS components are within expected ranges.
2. Unknown ECFP_2 feature: 1335702447: [*][c](:[*]):[c](C=[*]):c:[*]

Feature Contribution

Top features for positive contribution

Fingerprint	Bit/Smiles	Feature Structure	Score	Carcinogen in training set
ECFP_12	-1832102709	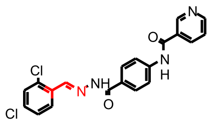 [*]\N=C\[c](:[*]):[*]	0.693	6 out of 8

ECFP_12	-223149939	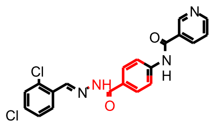 [*]NC(=O)[c]1:[cH]:[cH]:[*]:[cH]:[cH]:1	0.613	2 out of 2
ECFP_12	-177077903	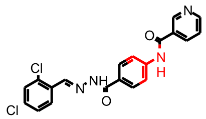 [*]N[c](:[cH]:[*]):[cH]:[*]	0.529	6 out of 10

Top Features for negative contribution

Fingerprint	Bit/Smiles	Feature Structure	Score	Carcinogen in training set
ECFP_12	1335691903	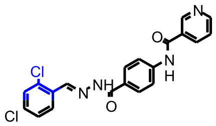 [*][c](:[*]):[c](Cl):[cH]:[*]	-1.11	2 out of 26
ECFP_12	99947387	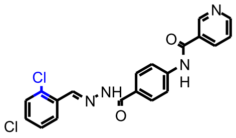 [*]:[c](:[*])Cl	-0.817	8 out of 62
ECFP_12	1854732111	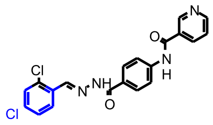 [*][c]1:[*]:[cH]:[c](Cl):[cH]:[cH]:1	-0.816	4 out of 33

TOPKAT_Rat_Female_FDA_None_vs_Carcinogen

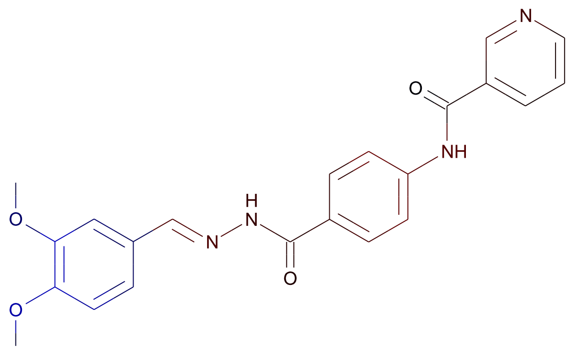

$C_{22}H_{20}N_4O_4$
Molecular Weight: 404.4186
ALogP: 2.284
Rotatable Bonds: 7
Acceptors: 6
Donors: 2

Model Prediction

Prediction: Carcinogen
Probability: 0.289
Enrichment: 0.896
Bayesian Score: -0.502
Mahalanobis Distance: 13.3
Mahalanobis Distance p-value: 4.06e-005

Prediction: Positive if the Bayesian score is above the estimated best cutoff value from minimizing the false positive and false negative rate.
Probability: The esimated probability that the sample is in the positive category. This assumes that the Bayesian score follows a normal distribution and is different from the prediction using a cutoff.
Enrichment: An estimate of enrichment, that is, the increased likelihood (versus random) of this sample being in the category.
Bayesian Score: The standard Laplacian-modified Bayesian score.
Mahalanobis Distance: The Mahalanobis distance (MD) is the distance to the center of the training data. The larger the MD, the less trustworthy the prediction.
Mahalanobis Distance p-value: The p-value gives the fraction of training data with an MD greater than or equal to the one for the given sample, assuming normally distributed data. The smaller the p-value, the less trustworthy the prediciton. For highly non-normal X properties (e.g., fingerprints), the MD p-value is wildly inaccurate.

Structural Similar Compounds

Name	Bicalutamide	Amlodipine	Moricizine
Structure	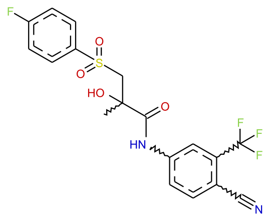	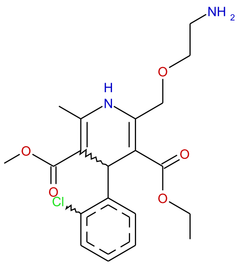	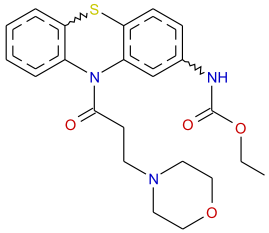
Actual Endpoint	Carcinogen	Non-Carcinogen	Carcinogen
Predicted Endpoint	Carcinogen	Non-Carcinogen	Carcinogen
Distance	0.609	0.622	0.644
Reference	US FDA (Centre for Drug Eval.& Res./Off. Testing & Res.) Sept. 1997	US FDA (Centre for Drug Eval.& Res./Off. Testing & Res.) Sept. 1997	US FDA (Centre for Drug Eval.& Res./Off. Testing & Res.) Sept. 1997

Model Applicability

Unknown features are fingerprint features in the query molecule, but not found or appearing too infrequently in the training set.

1. All properties and OPS components are within expected ranges.
2. Unknown ECFP_2 feature: -176483725: [*]=C[c](:c:[*]):c:[*]

Feature Contribution

Top features for positive contribution

Fingerprint	Bit/Smiles	Feature Structure	Score	Carcinogen in training set
ECFP_12	-1832102709	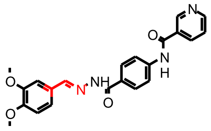 [*]\N=C\[c](:[*]):[*]	0.693	6 out of 8

ECFP_12	-223149939	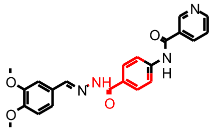 [*]NC(=O)[c]1:[cH]:[c H]:[*]:[cH]:[cH]:1	0.613	2 out of 2
ECFP_12	-177077903	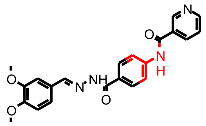 [*]N[c](:[cH]:[*]):[c H]:[*]	0.529	6 out of 10
**Top Features for negative contribution**				
**Fingerprint**	**Bit/Smiles**	**Feature Structure**	**Score**	**Carcinogen in training set**
ECFP_12	2077607946	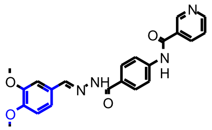 [*]O[c]1:[cH]:[cH]:[c]([*]):[*]:[c]:1[*]	-1.25	0 out of 8
ECFP_12	-468366781	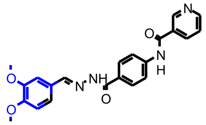 [*]O[c]1:[cH]:[*]:[c] ([*]):[cH]:[c]:1OC	-0.941	0 out of 5
ECFP_12	1408898974	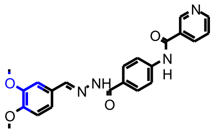 [*]O[c](:[cH]:[*]):[c]([*]):[*]	-0.517	5 out of 29

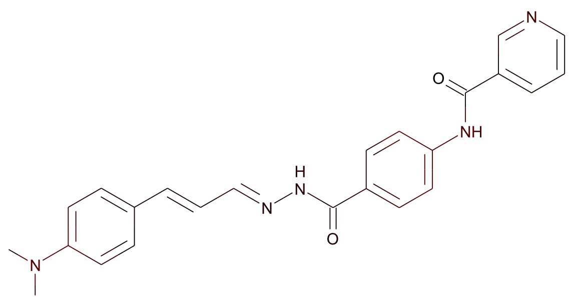

$C_{24}H_{23}N_5O_2$
Molecular Weight: 413.47172
ALogP: 2.839
Rotatable Bonds: 7
Acceptors: 5
Donors: 2

Model Prediction

Prediction: Carcinogen
Probability: 0.443
Enrichment: 1.38
Bayesian Score: 5.5
Mahalanobis Distance: 13.1
Mahalanobis Distance p-value: 0.000107

Prediction: Positive if the Bayesian score is above the estimated best cutoff value from minimizing the false positive and false negative rate.
Probability: The esimated probability that the sample is in the positive category. This assumes that the Bayesian score follows a normal distribution and is different from the prediction using a cutoff.
Enrichment: An estimate of enrichment, that is, the increased likelihood (versus random) of this sample being in the category.
Bayesian Score: The standard Laplacian-modified Bayesian score.
Mahalanobis Distance: The Mahalanobis distance (MD) is the distance to the center of the training data. The larger the MD, the less trustworthy the prediction.
Mahalanobis Distance p-value: The p-value gives the fraction of training data with an MD greater than or equal to the one for the given sample, assuming normally distributed data. The smaller the p-value, the less trustworthy the prediciton. For highly non-normal X properties (e.g., fingerprints), the MD p-value is wildly inaccurate.

Structural Similar Compounds

Name	Bicalutamide	Flecainide	Moricizine
Structure	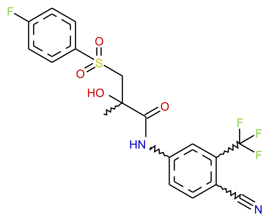	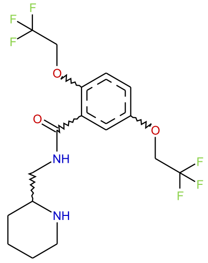	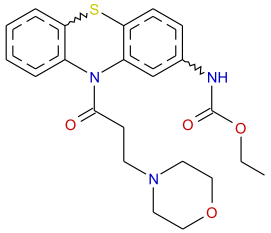
Actual Endpoint	Carcinogen	Non-Carcinogen	Carcinogen
Predicted Endpoint	Carcinogen	Non-Carcinogen	Carcinogen
Distance	0.602	0.655	0.657
Reference	US FDA (Centre for Drug Eval.& Res./Off. Testing & Res.) Sept. 1997	US FDA (Centre for Drug Eval.& Res./Off. Testing & Res.) Sept. 1997	US FDA (Centre for Drug Eval.& Res./Off. Testing & Res.) Sept. 1997

Model Applicability

Unknown features are fingerprint features in the query molecule, but not found or appearing too infrequently in the training set.

1. All properties and OPS components are within expected ranges.
2. Unknown ECFP_2 feature: -984423246: [*]\N=C\C=[*]
3. Unknown ECFP_2 feature: -176483725: [*]=C[c](:c:[*]):c:[*]

Feature Contribution

Top features for positive contribution				
**Fingerprint**	**Bit/Smiles**	**Feature Structure**	**Score**	**Carcinogen in training set**
ECFP_12	-223149939	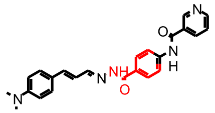 [*]NC(=O)[c]1:[cH]:[cH]:[*]:[cH]:[cH]:1	0.613	2 out of 2

ECFP_12	-1328426694	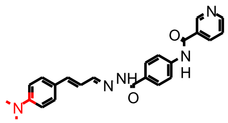<br>[*]:[c](:[*])N(C)C	0.613	2 out of 2
ECFP_12	-1140900255	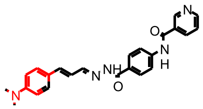<br>[*]N([*])[c]1:[cH]:[*]:[c]([*]):[cH]:[cH]:1	0.575	3 out of 4

Top Features for negative contribution

Fingerprint	Bit/Smiles	Feature Structure	Score	Carcinogen in training set
ECFP_12	1997021792	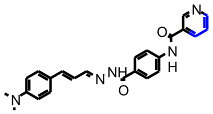<br>[*]:[cH]:[cH]:[cH]:[*]	-0.296	36 out of 156
ECFP_12	1451403962	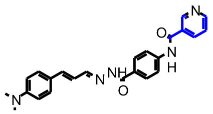<br>[*]C(=[*])[c]1:[cH]:[*]:[cH]:[cH]:[cH]:1	-0.248	1 out of 5
ECFP_12	1430169877	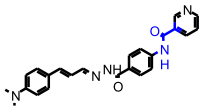<br>[*]NC(=O)[c](:[*]):[*]	-0.172	3 out of 12

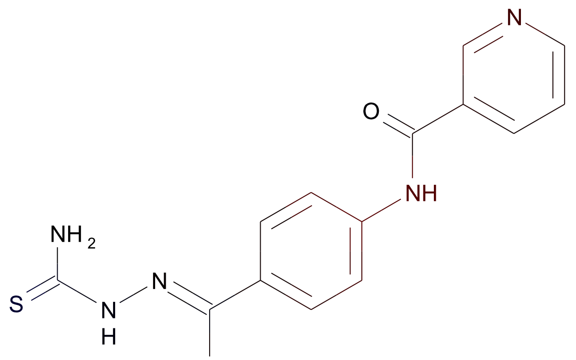

$C_{15}H_{15}N_5OS$
Molecular Weight: 313.3775
ALogP: 1.414
Rotatable Bonds: 5
Acceptors: 4
Donors: 3

Model Prediction

Prediction: Carcinogen
Probability: 0.328
Enrichment: 1.02
Bayesian Score: 1.36
Mahalanobis Distance: 12.9
Mahalanobis Distance p-value: 0.00024

Prediction: Positive if the Bayesian score is above the estimated best cutoff value from minimizing the false positive and false negative rate.
Probability: The esimated probability that the sample is in the positive category. This assumes that the Bayesian score follows a normal distribution and is different from the prediction using a cutoff.
Enrichment: An estimate of enrichment, that is, the increased likelihood (versus random) of this sample being in the category.
Bayesian Score: The standard Laplacian-modified Bayesian score.
Mahalanobis Distance: The Mahalanobis distance (MD) is the distance to the center of the training data. The larger the MD, the less trustworthy the prediction.
Mahalanobis Distance p-value: The p-value gives the fraction of training data with an MD greater than or equal to the one for the given sample, assuming normally distributed data. The smaller the p-value, the less trustworthy the prediciton. For highly non-normal X properties (e.g., fingerprints), the MD p-value is wildly inaccurate.

Structural Similar Compounds

Name	Furosemide	Tolbutamide	Chloramphenicol
Structure	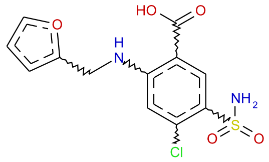	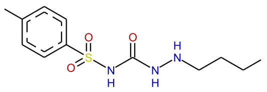	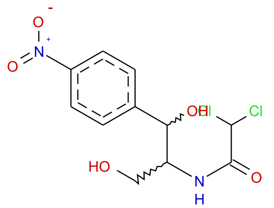
Actual Endpoint	Non-Carcinogen	Non-Carcinogen	Non-Carcinogen
Predicted Endpoint	Non-Carcinogen	Non-Carcinogen	Non-Carcinogen
Distance	0.582	0.586	0.587
Reference	US FDA (Centre for Drug Eval.& Res./Off. Testing & Res.) Sept. 1997	US FDA (Centre for Drug Eval.& Res./Off. Testing & Res.) Sept. 1997	US FDA (Centre for Drug Eval.& Res./Off. Testing & Res.) Sept. 1997

Model Applicability

Unknown features are fingerprint features in the query molecule, but not found or appearing too infrequently in the training set.

1. OPS PC22 out of range. Value: -3.728. Training min, max, SD, explained variance: -3.7128, 3.0259, 1.157, 0.0141.
2. Unknown ECFP_2 feature: 128986386: [*]\N=C(/C)\[c](:[*]):[*]
3. Unknown ECFP_2 feature: -571028867: [*]NC(=S)N

Feature Contribution

Top features for positive contribution

Fingerprint	Bit/Smiles	Feature Structure	Score	Carcinogen in training set
ECFP_12	-177077903	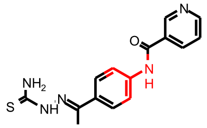 [*]N[c](:[cH]:[*]):[cH]:[*]	0.529	6 out of 10

ECFP_12	-1236483485	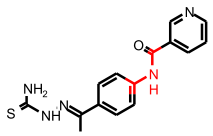 [*]C(=[*])N[c](:[*]):[*]	0.46	9 out of 17
ECFP_12	888054369	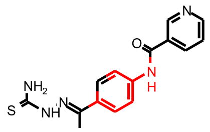 [*]N[c]1:[cH]:[*]:[c]([*]):[cH]:[cH]:1	0.454	5 out of 9
**Top Features for negative contribution**				
**Fingerprint**	**Bit/Smiles**	**Feature Structure**	**Score**	**Carcinogen in training set**
ECFP_12	1979182050	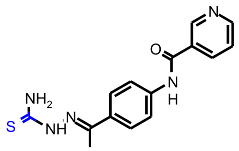 [*]C(=S)[*]	-0.485	0 out of 2
ECFP_12	-845108448	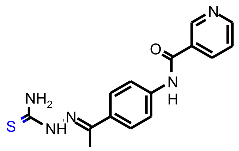 [*]=S	-0.485	0 out of 2
ECFP_12	1997021792	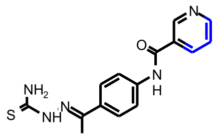 [*]:[cH]:[cH]:[cH]:[*]	-0.296	36 out of 156

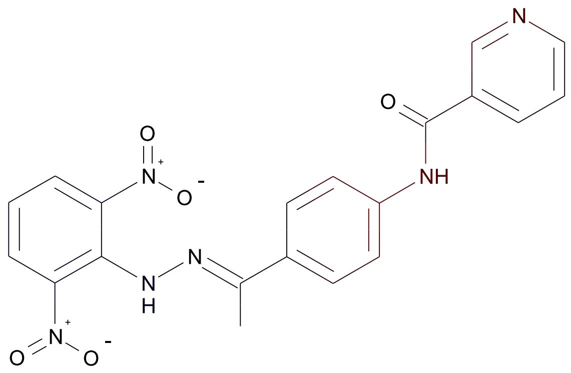

$C_{20}H_{16}N_6O_5$
Molecular Weight: 420.37824
ALogP: 2.852
Rotatable Bonds: 7
Acceptors: 8
Donors: 2

Model Prediction

Prediction: Carcinogen
Probability: 0.314
Enrichment: 0.976
Bayesian Score: 0.748
Mahalanobis Distance: 12.3
Mahalanobis Distance p-value: 0.00179

Prediction: Positive if the Bayesian score is above the estimated best cutoff value from minimizing the false positive and false negative rate.
Probability: The esimated probability that the sample is in the positive category. This assumes that the Bayesian score follows a normal distribution and is different from the prediction using a cutoff.
Enrichment: An estimate of enrichment, that is, the increased likelihood (versus random) of this sample being in the category.
Bayesian Score: The standard Laplacian-modified Bayesian score.
Mahalanobis Distance: The Mahalanobis distance (MD) is the distance to the center of the training data. The larger the MD, the less trustworthy the prediction.
Mahalanobis Distance p-value: The p-value gives the fraction of training data with an MD greater than or equal to the one for the given sample, assuming normally distributed data. The smaller the p-value, the less trustworthy the prediciton. For highly non-normal X properties (e.g., fingerprints), the MD p-value is wildly inaccurate.

Structural Similar Compounds

Name	Sulfasalazine	Nedocromil	Carbenicillin
Structure	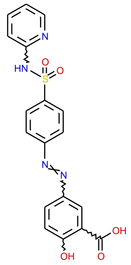	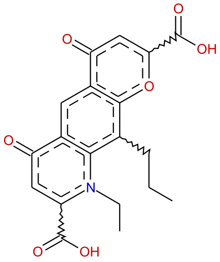	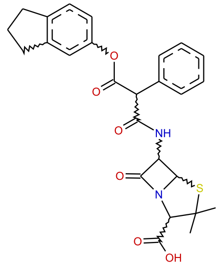
Actual Endpoint	Non-Carcinogen	Non-Carcinogen	Non-Carcinogen
Predicted Endpoint	Non-Carcinogen	Non-Carcinogen	Non-Carcinogen
Distance	0.641	0.643	0.683
Reference	US FDA (Centre for Drug Eval.& Res./Off. Testing & Res.) Sept. 1997	US FDA (Centre for Drug Eval.& Res./Off. Testing & Res.) Sept. 1997	US FDA (Centre for Drug Eval.& Res./Off. Testing & Res.) Sept. 1997

Model Applicability

Unknown features are fingerprint features in the query molecule, but not found or appearing too infrequently in the training set.

1. All properties and OPS components are within expected ranges.
2. Unknown ECFP_2 feature: 1043790491: [*][N+](=[*])[*]
3. Unknown ECFP_2 feature: 781519895: [*][O-]
4. Unknown ECFP_2 feature: 128986386: [*]\N=C(/C)\[c](:[*]):[*]
5. Unknown ECFP_2 feature: -1236714312: [*]=NN[c](:[*]):[*]
6. Unknown ECFP_2 feature: -1956535100: [*][c](:[*]):[c]([N+](=[*])[*]):c:[*]
7. Unknown ECFP_2 feature: -215026467: [*]:[c](:[*])[N+](=O)[O-]
8. Unknown ECFP_2 feature: 2104376220: [*][N+](=O)[*]
9. Unknown ECFP_2 feature: -659271057: [*][N+](=[*])[O-]

Feature Contribution

Top features for positive contribution				
**Fingerprint**	**Bit/Smiles**	**Feature Structure**	**Score**	**Carcinogen in training set**

ECFP_12	-177077903	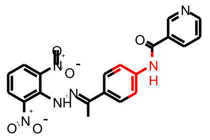 [*]N[c](:[cH]:[*]):[cH]:[*]	0.529	6 out of 10
ECFP_12	-1236483485	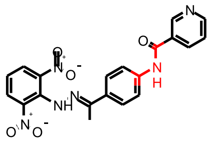 [*]C(=[*])N[c](:[*]):[*]	0.46	9 out of 17
ECFP_12	888054369	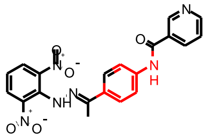 [*]N[c]1:[cH]:[*]:[c]([*]):[cH]:[cH]:1	0.454	5 out of 9
**Top Features for negative contribution**				
**Fingerprint**	**Bit/Smiles**	**Feature Structure**	**Score**	**Carcinogen in training set**
ECFP_12	2007300961	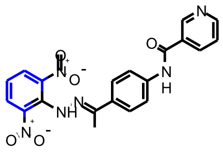 [*][c]1:[*]:[c]([*]):[cH]:[cH]:[cH]:1	-0.426	7 out of 36
ECFP_12	1997021792	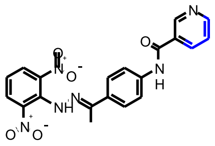 [*]:[cH]:[cH]:[cH]:[*]	-0.296	36 out of 156

ECFP_12	560380707	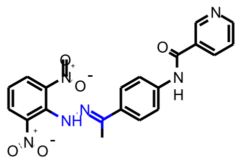<br>[*]NN=C([*])[*]	-0.272	0 out of 1

Sorafenib

TOPKAT_Rat_Female_FDA_None_vs_Carcinogen

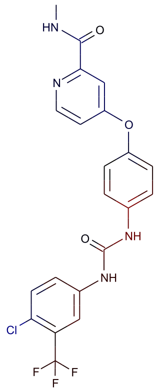

$C_{21}H_{16}ClF_3N_4O_3$
Molecular Weight: 464.82494
ALogP: 4.175
Rotatable Bonds: 6
Acceptors: 4
Donors: 3

Structural Similar Compounds

Name	Glimepride	Glyburide	Fluvastatin
Structure	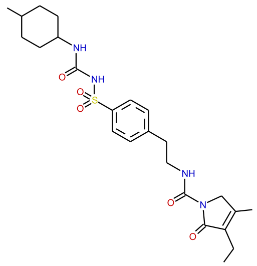	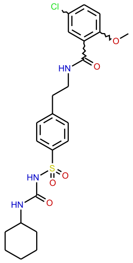	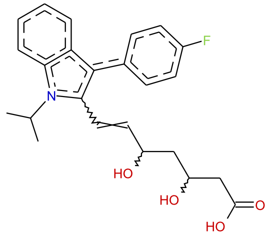
Actual Endpoint	Non-Carcinogen	Non-Carcinogen	Non-Carcinogen
Predicted Endpoint	Non-Carcinogen	Non-Carcinogen	Non-Carcinogen
Distance	0.620	0.635	0.635
Reference	US FDA (Centre for Drug Eval.& Res./Off. Testing & Res.) Sept. 1997	US FDA (Centre for Drug Eval.& Res./Off. Testing & Res.) Sept. 1997	US FDA (Centre for Drug Eval.& Res./Off. Testing & Res.) Sept. 1997

Model Prediction

Prediction: Non-Carcinogen
Probability: 0.236
Enrichment: 0.734
Bayesian Score: -3.76
Mahalanobis Distance: 12.2
Mahalanobis Distance p-value: 0.00229

Prediction: Positive if the Bayesian score is above the estimated best cutoff value from minimizing the false positive and false negative rate.
Probability: The esimated probability that the sample is in the positive category. This assumes that the Bayesian score follows a normal distribution and is different from the prediction using a cutoff.
Enrichment: An estimate of enrichment, that is, the increased likelihood (versus random) of this sample being in the category.
Bayesian Score: The standard Laplacian-modified Bayesian score.
Mahalanobis Distance: The Mahalanobis distance (MD) is the distance to the center of the training data. The larger the MD, the less trustworthy the prediction.
Mahalanobis Distance p-value: The p-value gives the fraction of training data with an MD greater than or equal to the one for the given sample, assuming normally distributed data. The smaller the p-value, the less trustworthy the prediciton. For highly non-normal X properties (e.g., fingerprints), the MD p-value is wildly inaccurate.

Model Applicability

Unknown features are fingerprint features in the query molecule, but not found or appearing too infrequently in the training set.

1. All properties and OPS components are within expected ranges.

Feature Contribution

Top features for positive contribution

Fingerprint	Bit/Smiles	Feature Structure	Score	Carcinogen in training set
ECFP_12	-970385855	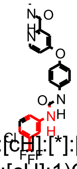 [*]N[c]1:[cH]:[*]:[c]([*]):[c](:[cH]:1)C([*])([*])[*]	0.613	2 out of 2

ECFP_12	-177077903	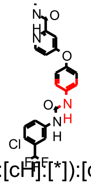 [*]N[c](:[cH]:[*]):[cH]:[*]	0.529	6 out of 10
ECFP_12	-1236483485	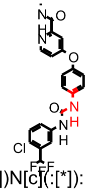 [*]C(=[*])N[c](:[*]):[*]	0.46	9 out of 17

Top Features for negative contribution

Fingerprint	Bit/Smiles	Feature Structure	Score	Carcinogen in training set
ECFP_12	1335691903	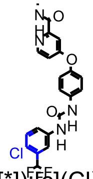 [*][c](:[*]):[c](Cl):[cH]:[*]	-1.11	2 out of 26
ECFP_12	99947387	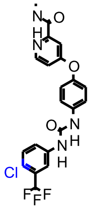 [*]:[c](:[*])Cl	-0.817	8 out of 62
ECFP_12	1413420509	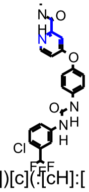 [*]C(=[*])[c](:[cH]:[*]):n:[*]	-0.661	0 out of 3

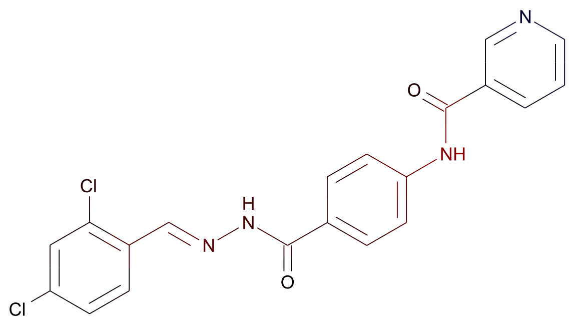

$C_{20}H_{14}Cl_2N_4O_2$
Molecular Weight: 413.25676
ALogP: 3.646
Rotatable Bonds: 5
Acceptors: 4
Donors: 2

Model Prediction

Prediction: Multiple-Carcinogen
Probability: 0.706
Enrichment: 1.89
Bayesian Score: 5.65
Mahalanobis Distance: 12.5
Mahalanobis Distance p-value: 0.000613

Prediction: Positive if the Bayesian score is above the estimated best cutoff value from minimizing the false positive and false negative rate.
Probability: The esimated probability that the sample is in the positive category. This assumes that the Bayesian score follows a normal distribution and is different from the prediction using a cutoff.
Enrichment: An estimate of enrichment, that is, the increased likelihood (versus random) of this sample being in the category.
Bayesian Score: The standard Laplacian-modified Bayesian score.
Mahalanobis Distance: The Mahalanobis distance (MD) is the distance to the center of the training data. The larger the MD, the less trustworthy the prediction.
Mahalanobis Distance p-value: The p-value gives the fraction of training data with an MD greater than or equal to the one for the given sample, assuming normally distributed data. The smaller the p-value, the less trustworthy the prediciton. For highly non-normal X properties (e.g., fingerprints), the MD p-value is wildly inaccurate.

Structural Similar Compounds

Name	Doxefazepam	Bicalutamide	Lansoprazole
Structure	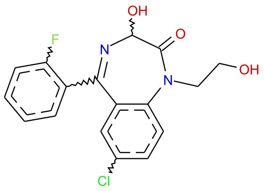	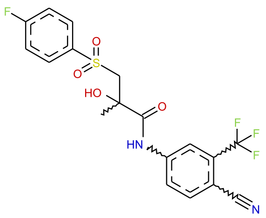	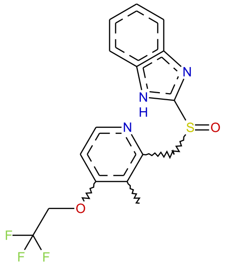
Actual Endpoint	Multiple-Carcinogen	Multiple-Carcinogen	Single-Carcinogen
Predicted Endpoint	Multiple-Carcinogen	Multiple-Carcinogen	Single-Carcinogen
Distance	0.564	0.595	0.659
Reference	US FDA (Centre for Drug Eval.& Res./Off. Testing & Res.) Sept. 1997	US FDA (Centre for Drug Eval.& Res./Off. Testing & Res.) Sept. 1997	US FDA (Centre for Drug Eval.& Res./Off. Testing & Res.) Sept. 1997

Model Applicability

Unknown features are fingerprint features in the query molecule, but not found or appearing too infrequently in the training set.

1. All properties and OPS components are within expected ranges.

Feature Contribution

Top features for positive contribution

Fingerprint	Bit/Smiles	Feature Structure	Score	Multiple-Carcinogen in training set
SCFP_4	1631845520	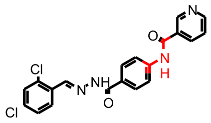 [*]C(=[*])N[c](:[*]):[*]	0.601	6 out of 9

SCFP_4	-1375926917	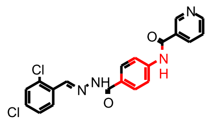 [*]N[c]1:[cH]:[*]:[c]([*]):[cH]:[cH]:1	0.522	6 out of 10
SCFP_4	1205586762	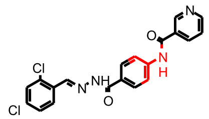 [*]N[c](:[cH]:[*]):[cH]:[*]	0.451	7 out of 13

Top Features for negative contribution

Fingerprint	Bit/Smiles	Feature Structure	Score	Multiple-Carcinogen in training set
SCFP_4	1188429584	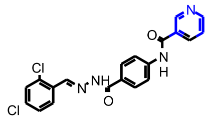 [*][c]1:[*]:[cH]:[cH]:n:[cH]:1	-0.666	0 out of 3
SCFP_4	-758850909	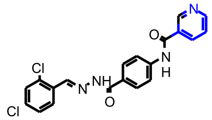 [*][c]1:[*]:n:[cH]:[cH]:[cH]:1	-0.489	0 out of 2
SCFP_4	-937094999	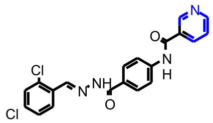 [*]1:[cH]:[cH]:[cH]:n:[cH]:1	-0.368	1 out of 6

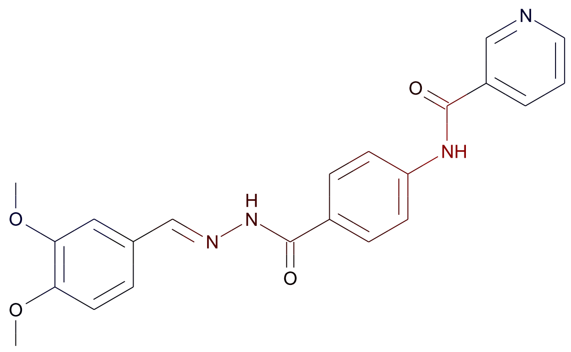

$C_{22}H_{20}N_4O_4$
Molecular Weight: 404.4186
ALogP: 2.284
Rotatable Bonds: 7
Acceptors: 6
Donors: 2

Model Prediction

Prediction: Multiple-Carcinogen
Probability: 0.579
Enrichment: 1.55
Bayesian Score: 2.77
Mahalanobis Distance: 19.3
Mahalanobis Distance p-value: 2.43e-010

Prediction: Positive if the Bayesian score is above the estimated best cutoff value from minimizing the false positive and false negative rate.
Probability: The esimated probability that the sample is in the positive category. This assumes that the Bayesian score follows a normal distribution and is different from the prediction using a cutoff.
Enrichment: An estimate of enrichment, that is, the increased likelihood (versus random) of this sample being in the category.
Bayesian Score: The standard Laplacian-modified Bayesian score.
Mahalanobis Distance: The Mahalanobis distance (MD) is the distance to the center of the training data. The larger the MD, the less trustworthy the prediction.
Mahalanobis Distance p-value: The p-value gives the fraction of training data with an MD greater than or equal to the one for the given sample, assuming normally distributed data. The smaller the p-value, the less trustworthy the prediciton. For highly non-normal X properties (e.g., fingerprints), the MD p-value is wildly inaccurate.

Structural Similar Compounds

Name	Bicalutamide	Moricizine	Omeprazole
Structure	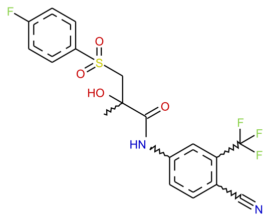	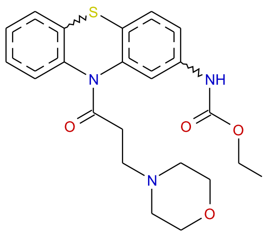	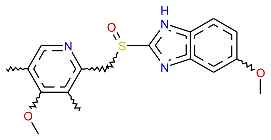
Actual Endpoint	Multiple-Carcinogen	Single-Carcinogen	Multiple-Carcinogen
Predicted Endpoint	Multiple-Carcinogen	Single-Carcinogen	Multiple-Carcinogen
Distance	0.568	0.626	0.665
Reference	US FDA (Centre for Drug Eval.& Res./Off. Testing & Res.) Sept. 1997	US FDA (Centre for Drug Eval.& Res./Off. Testing & Res.) Sept. 1997	US FDA (Centre for Drug Eval.& Res./Off. Testing & Res.) Sept. 1997

Model Applicability

Unknown features are fingerprint features in the query molecule, but not found or appearing too infrequently in the training set.

1. All properties and OPS components are within expected ranges.

Feature Contribution

Top features for positive contribution

Fingerprint	Bit/Smiles	Feature Structure	Score	Multiple-Carcinogen in training set
SCFP_4	1631845520	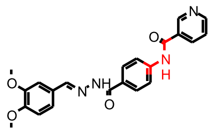 [*]C(=[*])N[c](:[*]):[*]	0.601	6 out of 9

SCFP_4	-1375926917	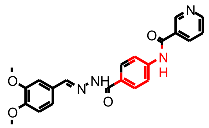 [*]N[c]1:[cH]:[*]:[c]([*]):[cH]:[cH]:1	0.522	6 out of 10
SCFP_4	1205586762	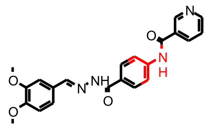 [*]N[c](:[cH]:[*]):[cH]:[*]	0.451	7 out of 13

Top Features for negative contribution

Fingerprint	Bit/Smiles	Feature Structure	Score	Multiple-Carcinogen in training set
SCFP_4	1188429584	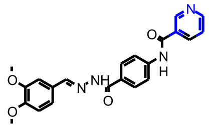 [*][c]1:[*]:[cH]:[cH]:n:[cH]:1	-0.666	0 out of 3
SCFP_4	-758850909	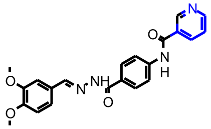 [*][c]1:[*]:n:[cH]:[cH]:[cH]:1	-0.489	0 out of 2
SCFP_4	392579710	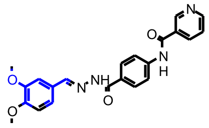 [*]O[c]1:[cH]:[c](C=[*]):[cH]:[*]:[c]:1[*]	-0.489	0 out of 2

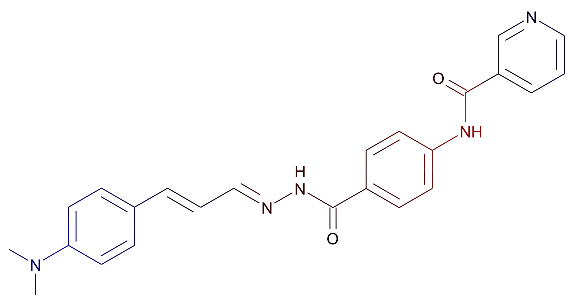

$C_{24}H_{23}N_5O_2$
Molecular Weight: 413.47172
ALogP: 2.839
Rotatable Bonds: 7
Acceptors: 5
Donors: 2

Model Prediction

Prediction: Multiple-Carcinogen
Probability: 0.532
Enrichment: 1.42
Bayesian Score: 1.52
Mahalanobis Distance: 14.2
Mahalanobis Distance p-value: 1.84e-005

Prediction: Positive if the Bayesian score is above the estimated best cutoff value from minimizing the false positive and false negative rate.
Probability: The esimated probability that the sample is in the positive category. This assumes that the Bayesian score follows a normal distribution and is different from the prediction using a cutoff.
Enrichment: An estimate of enrichment, that is, the increased likelihood (versus random) of this sample being in the category.
Bayesian Score: The standard Laplacian-modified Bayesian score.
Mahalanobis Distance: The Mahalanobis distance (MD) is the distance to the center of the training data. The larger the MD, the less trustworthy the prediction.
Mahalanobis Distance p-value: The p-value gives the fraction of training data with an MD greater than or equal to the one for the given sample, assuming normally distributed data. The smaller the p-value, the less trustworthy the prediciton. For highly non-normal X properties (e.g., fingerprints), the MD p-value is wildly inaccurate.

Structural Similar Compounds

Name	Bicalutamide	Moricizine	Lansoprazole
Structure	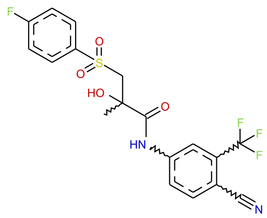	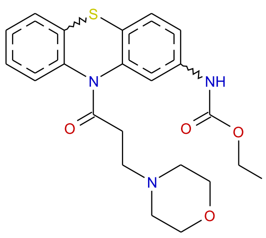	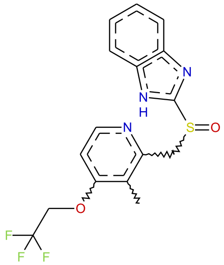
Actual Endpoint	Multiple-Carcinogen	Single-Carcinogen	Single-Carcinogen
Predicted Endpoint	Multiple-Carcinogen	Single-Carcinogen	Single-Carcinogen
Distance	0.556	0.649	0.688
Reference	US FDA (Centre for Drug Eval.& Res./Off. Testing & Res.) Sept. 1997	US FDA (Centre for Drug Eval.& Res./Off. Testing & Res.) Sept. 1997	US FDA (Centre for Drug Eval.& Res./Off. Testing & Res.) Sept. 1997

Model Applicability

Unknown features are fingerprint features in the query molecule, but not found or appearing too infrequently in the training set.

1. OPS PC16 out of range. Value: 2.8516. Training min, max, SD, explained variance: -2.5301, 2.7699, 1.075, 0.0171.

Feature Contribution

Top features for positive contribution

Fingerprint	Bit/Smiles	Feature Structure	Score	Multiple-Carcinogen in training set
SCFP_4	1631845520	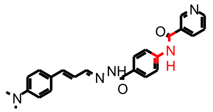 [*]C(=[*])N[c](:[*]):[*]	0.601	6 out of 9

SCFP_4	-1375926917	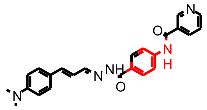 [*]N[c]1:[cH]:[*]:[c]([*]):[cH]:[cH]:1	0.522	6 out of 10
SCFP_4	1205586762	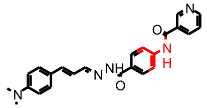 [*]N[c](:[cH]:[*]):[cH]:[*]	0.451	7 out of 13

Top Features for negative contribution

Fingerprint	Bit/Smiles	Feature Structure	Score	Multiple-Carcinogen in training set
SCFP_4	-299436502	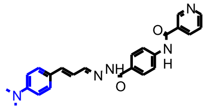 CN(C)[c]1:[cH]:[cH]:[*]:[cH]:[cH]:1	-0.946	0 out of 5
SCFP_4	1188429584	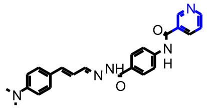 [*][c]1:[*]:[cH]:[cH]:n:[cH]:1	-0.666	0 out of 3
SCFP_4	-1853624961	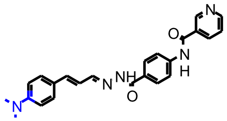 [*]:[c](:[*])N(C)C	-0.651	1 out of 9

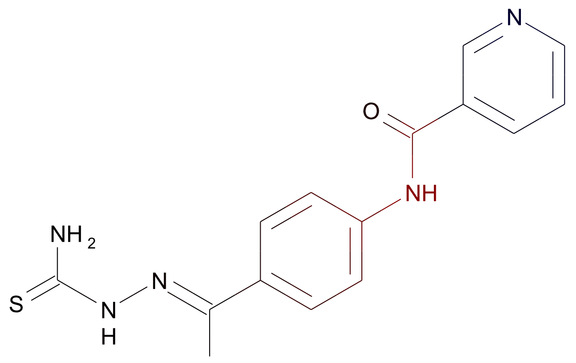

$C_{15}H_{15}N_5OS$
Molecular Weight: 313.3775
ALogP: 1.414
Rotatable Bonds: 5
Acceptors: 4
Donors: 3

Model Prediction

Prediction: Multiple-Carcinogen
Probability: 0.567
Enrichment: 1.52
Bayesian Score: 2.52
Mahalanobis Distance: 13.4
Mahalanobis Distance p-value: 9.86e-005

Prediction: Positive if the Bayesian score is above the estimated best cutoff value from minimizing the false positive and false negative rate.
Probability: The esimated probability that the sample is in the positive category. This assumes that the Bayesian score follows a normal distribution and is different from the prediction using a cutoff.
Enrichment: An estimate of enrichment, that is, the increased likelihood (versus random) of this sample being in the category.
Bayesian Score: The standard Laplacian-modified Bayesian score.
Mahalanobis Distance: The Mahalanobis distance (MD) is the distance to the center of the training data. The larger the MD, the less trustworthy the prediction.
Mahalanobis Distance p-value: The p-value gives the fraction of training data with an MD greater than or equal to the one for the given sample, assuming normally distributed data. The smaller the p-value, the less trustworthy the prediciton. For highly non-normal X properties (e.g., fingerprints), the MD p-value is wildly inaccurate.

Structural Similar Compounds

Name	Torsemide	Sulfamethazine	Atenolol
Structure	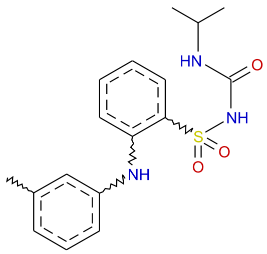	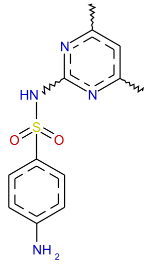	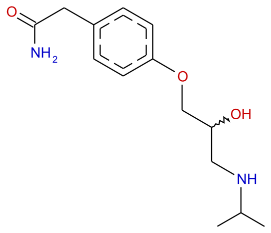
Actual Endpoint	Single-Carcinogen	Single-Carcinogen	Multiple-Carcinogen
Predicted Endpoint	Single-Carcinogen	Single-Carcinogen	Multiple-Carcinogen
Distance	0.665	0.682	0.689
Reference	US FDA (Centre for Drug Eval.& Res./Off. Testing & Res.) Sept. 1997	US FDA (Centre for Drug Eval.& Res./Off. Testing & Res.) Sept. 1997	US FDA (Centre for Drug Eval.& Res./Off. Testing & Res.) Sept. 1997

Model Applicability

Unknown features are fingerprint features in the query molecule, but not found or appearing too infrequently in the training set.

1. All properties and OPS components are within expected ranges.

Feature Contribution

Top features for positive contribution				
**Fingerprint**	**Bit/Smiles**	**Feature Structure**	**Score**	**Multiple-Carcinogen in training set**
SCFP_4	1631845520	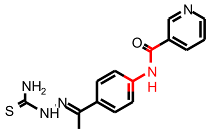 [*]C(=[*])N[c](:[*]):[*]	0.601	6 out of 9

SCFP_4	-1375926917	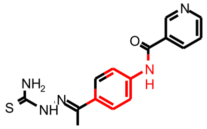 [*]N[c]1:[cH]:[*]:[c]([*]):[cH]:[cH]:1	0.522	6 out of 10
SCFP_4	1205586762	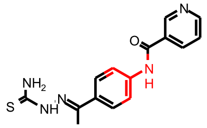 [*]N[c](:[cH]:[*]):[cH]:[*]	0.451	7 out of 13
**Top Features for negative contribution**				
**Fingerprint**	**Bit/Smiles**	**Feature Structure**	**Score**	**Multiple-Carcinogen in training set**
SCFP_4	1188429584	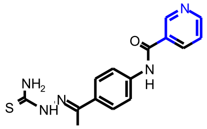 [*][c]1:[*]:[cH]:[cH]:n:[cH]:1	-0.666	0 out of 3
SCFP_4	-758850909	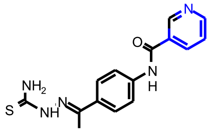 [*][c]1:[*]:n:[cH]:[cH]:[cH]:1	-0.489	0 out of 2
SCFP_4	-937094999	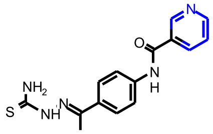 [*]1:[cH]:[cH]:[cH]:n:[cH]:1	-0.368	1 out of 6

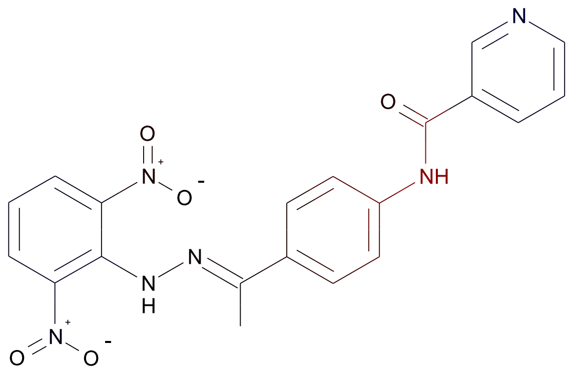

$C_{20}H_{16}N_6O_5$
Molecular Weight: 420.37824
ALogP: 2.852
Rotatable Bonds: 7
Acceptors: 8
Donors: 2

Model Prediction

Prediction: Multiple-Carcinogen
Probability: 0.561
Enrichment: 1.5
Bayesian Score: 2.38
Mahalanobis Distance: 22.2
Mahalanobis Distance p-value: 5.66e-013

Prediction: Positive if the Bayesian score is above the estimated best cutoff value from minimizing the false positive and false negative rate.
Probability: The esimated probability that the sample is in the positive category. This assumes that the Bayesian score follows a normal distribution and is different from the prediction using a cutoff.
Enrichment: An estimate of enrichment, that is, the increased likelihood (versus random) of this sample being in the category.
Bayesian Score: The standard Laplacian-modified Bayesian score.
Mahalanobis Distance: The Mahalanobis distance (MD) is the distance to the center of the training data. The larger the MD, the less trustworthy the prediction.
Mahalanobis Distance p-value: The p-value gives the fraction of training data with an MD greater than or equal to the one for the given sample, assuming normally distributed data. The smaller the p-value, the less trustworthy the prediciton. For highly non-normal X properties (e.g., fingerprints), the MD p-value is wildly inaccurate.

Structural Similar Compounds

Name	Timolol	Moricizine	Lasiocarpine
Structure	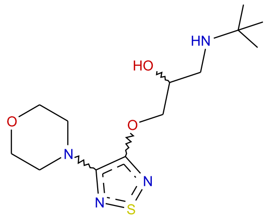	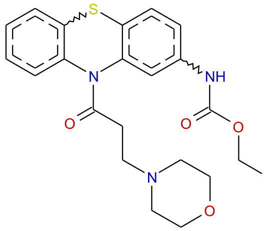	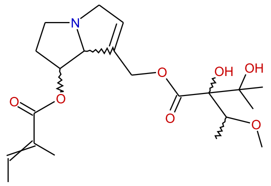
Actual Endpoint	Single-Carcinogen	Single-Carcinogen	Single-Carcinogen
Predicted Endpoint	Single-Carcinogen	Single-Carcinogen	Single-Carcinogen
Distance	0.746	0.752	0.757
Reference	US FDA (Centre for Drug Eval.& Res./Off. Testing & Res.) Sept. 1997	US FDA (Centre for Drug Eval.& Res./Off. Testing & Res.) Sept. 1997	US FDA (Centre for Drug Eval.& Res./Off. Testing & Res.) Sept. 1997

Model Applicability

Unknown features are fingerprint features in the query molecule, but not found or appearing too infrequently in the training set.

1. All properties and OPS components are within expected ranges.

Feature Contribution

Top features for positive contribution				
**Fingerprint**	**Bit/Smiles**	**Feature Structure**	**Score**	**Multiple-Carcinogen in training set**
SCFP_4	1631845520	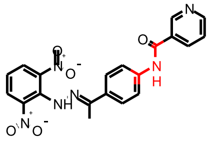 [*]C(=[*])N[c](:[*]):[*]	0.601	6 out of 9

SCFP_4	-1375926917	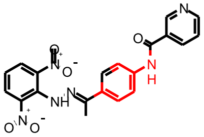 [*]N[c]1:[cH]:[*]:[c]([*]):[cH]:[cH]:1	0.522	6 out of 10
SCFP_4	1311339974	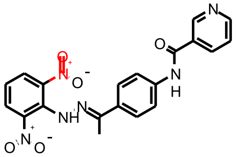 [*][N+](=O)[*]	0.504	9 out of 16
**Top Features for negative contribution**				
**Fingerprint**	**Bit/Smiles**	**Feature Structure**	**Score**	**Multiple-Carcinogen in training set**
SCFP_4	1188429584	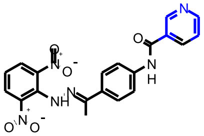 [*][c]1:[*]:[cH]:[cH]:n:[cH]:1	-0.666	0 out of 3
SCFP_4	-758850909	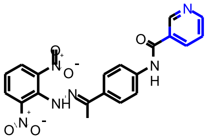 [*][c]1:[*]:n:[cH]:[cH]:[cH]:1	-0.489	0 out of 2
SCFP_4	-1380909229	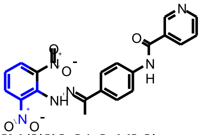 [*]N([*])[c]1:[cH]:[*]:[c]([*]):[cH]:[cH]:1	-0.413	3 out of 16

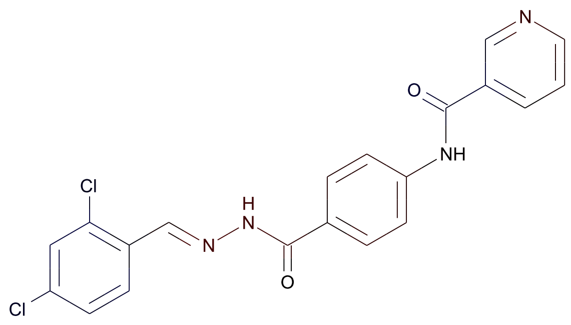

$C_{20}H_{14}Cl_2N_4O_2$

Molecular Weight: 413.25676

ALogP: 3.646

Rotatable Bonds: 5

Acceptors: 4

Donors: 2

Model Prediction

Prediction: Carcinogen

Probability: 0.38

Enrichment: 1.14

Bayesian Score: 0.793

Mahalanobis Distance: 13.1

Mahalanobis Distance p-value: 0.00126

Prediction: Positive if the Bayesian score is above the estimated best cutoff value from minimizing the false positive and false negative rate.
Probability: The esimated probability that the sample is in the positive category. This assumes that the Bayesian score follows a normal distribution and is different from the prediction using a cutoff.
Enrichment: An estimate of enrichment, that is, the increased likelihood (versus random) of this sample being in the category.
Bayesian Score: The standard Laplacian-modified Bayesian score.
Mahalanobis Distance: The Mahalanobis distance (MD) is the distance to the center of the training data. The larger the MD, the less trustworthy the prediction.
Mahalanobis Distance p-value: The p-value gives the fraction of training data with an MD greater than or equal to the one for the given sample, assuming normally distributed data. The smaller the p-value, the less trustworthy the prediciton. For highly non-normal X properties (e.g., fingerprints), the MD p-value is wildly inaccurate.

Structural Similar Compounds

Name	Doxefazepam	Bicalutamide	Indomethacin
Structure	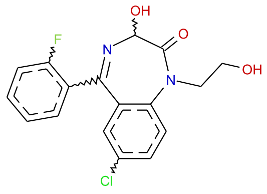	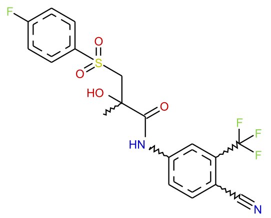	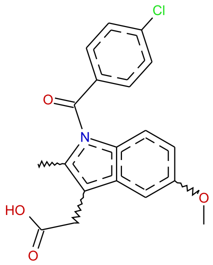
Actual Endpoint	Carcinogen	Carcinogen	Non-Carcinogen
Predicted Endpoint	Carcinogen	Carcinogen	Non-Carcinogen
Distance	0.611	0.611	0.622
Reference	US FDA (Centre for Drug Eval.& Res./Off. Testing & Res.) Sept. 1997	US FDA (Centre for Drug Eval.& Res./Off. Testing & Res.) Sept. 1997	US FDA (Centre for Drug Eval.& Res./Off. Testing & Res.) Sept. 1997

Model Applicability

Unknown features are fingerprint features in the query molecule, but not found or appearing too infrequently in the training set.

1. OPS PC1 out of range. Value: -5.8741. Training min, max, SD, explained variance: -5.694, 8.348, 2.949, 0.0811.

Feature Contribution

Top features for positive contribution

Fingerprint	Bit/Smiles	Feature Structure	Score	Carcinogen in training set
SCFP_6	-347048986	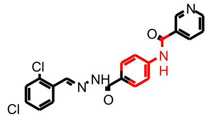 [*]C(=[*])N[c]1:[cH]:[cH]:[*]:[cH]:[cH]:1	0.615	5 out of 7

SCFP_6	1926385222	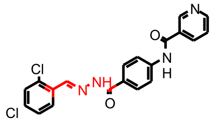 [*]C(=[*])N\N=C\[c](:[*]):[*]	0.415	1 out of 1
SCFP_6	698322229	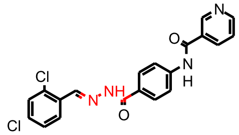 [*]C(=[*])NN=[*]	0.415	1 out of 1

Top Features for negative contribution

Fingerprint	Bit/Smiles	Feature Structure	Score	Carcinogen in training set
SCFP_6	1915307678	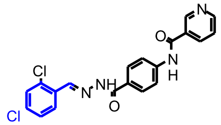 [*][c]1:[cH]:[c](Cl):[cH]:[cH]:[c]:1C=[*]	-0.496	0 out of 2
SCFP_6	1257084377	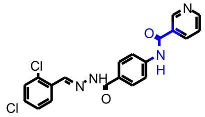 [*]NC(=O)[c](:[*]):[*]	-0.436	4 out of 21
SCFP_6	-52074512	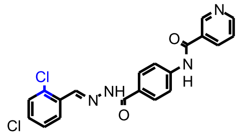 [*]:[c](:[*])Cl	-0.315	14 out of 61

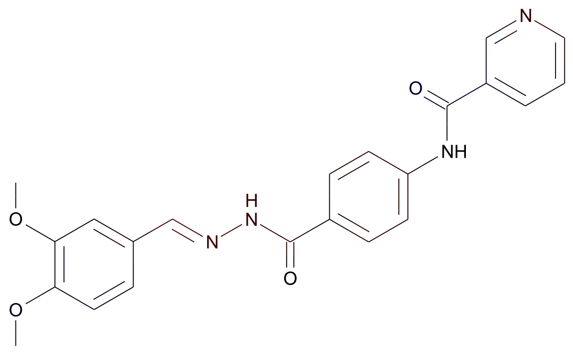

$C_{22}H_{20}N_4O_4$
Molecular Weight: 404.4186
ALogP: 2.284
Rotatable Bonds: 7
Acceptors: 6
Donors: 2

Model Prediction

Prediction: Carcinogen
Probability: 0.374
Enrichment: 1.12
Bayesian Score: 0.587
Mahalanobis Distance: 15
Mahalanobis Distance p-value: 5.58e-007

Prediction: Positive if the Bayesian score is above the estimated best cutoff value from minimizing the false positive and false negative rate.
Probability: The esimated probability that the sample is in the positive category. This assumes that the Bayesian score follows a normal distribution and is different from the prediction using a cutoff.
Enrichment: An estimate of enrichment, that is, the increased likelihood (versus random) of this sample being in the category.
Bayesian Score: The standard Laplacian-modified Bayesian score.
Mahalanobis Distance: The Mahalanobis distance (MD) is the distance to the center of the training data. The larger the MD, the less trustworthy the prediction.
Mahalanobis Distance p-value: The p-value gives the fraction of training data with an MD greater than or equal to the one for the given sample, assuming normally distributed data. The smaller the p-value, the less trustworthy the prediciton. For highly non-normal X properties (e.g., fingerprints), the MD p-value is wildly inaccurate.

Structural Similar Compounds

Name	Bicalutamide	Moricizine	Cisapride
Structure	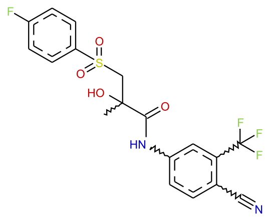	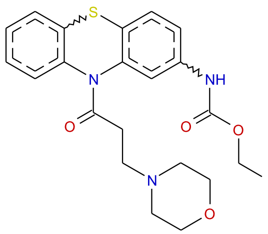	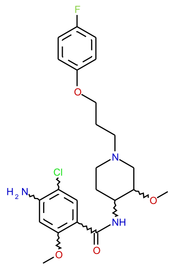
Actual Endpoint	Carcinogen	Carcinogen	Non-Carcinogen
Predicted Endpoint	Carcinogen	Carcinogen	Non-Carcinogen
Distance	0.586	0.624	0.627
Reference	US FDA (Centre for Drug Eval.& Res./Off. Testing & Res.) Sept. 1997	US FDA (Centre for Drug Eval.& Res./Off. Testing & Res.) Sept. 1997	US FDA (Centre for Drug Eval.& Res./Off. Testing & Res.) Sept. 1997

Model Applicability

Unknown features are fingerprint features in the query molecule, but not found or appearing too infrequently in the training set.

1. All properties and OPS components are within expected ranges.

Feature Contribution

Top features for positive contribution				
**Fingerprint**	**Bit/Smiles**	**Feature Structure**	**Score**	**Carcinogen in training set**
SCFP_6	-347048986	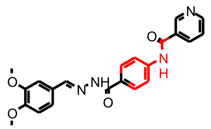 [*]C(=[*])N[c]1:[cH]:[cH]:[*]:[cH]:[cH]:1	0.615	5 out of 7

SCFP_6	392579710	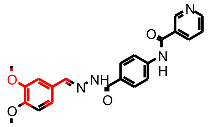 [*]O[c]1:[cH]:[c](C=[*]):[cH]:[*]:[c]:1[*]	0.425	2 out of 3
SCFP_6	-105808146	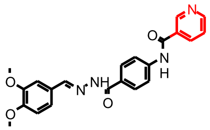 [*][c]1:[cH]:[cH]:[cH]:n:[cH]:1	0.415	1 out of 1
**Top Features for negative contribution**				
**Fingerprint**	**Bit/Smiles**	**Feature Structure**	**Score**	**Carcinogen in training set**
SCFP_6	1257084377	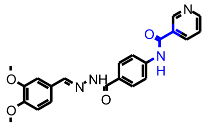 [*]NC(=O)[c](:[*]):[*]	-0.436	4 out of 21
SCFP_6	124026986	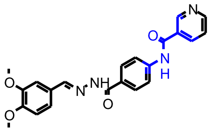 [*]:[cH]:[c](:[cH]:[*])C(=O)N[c](:[*]):[*]	-0.278	0 out of 1
SCFP_6	-1503458502	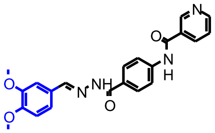 [*][c]1:[cH]:[cH]:[c] (OC):[c](OC):[cH]:1	-0.278	0 out of 1

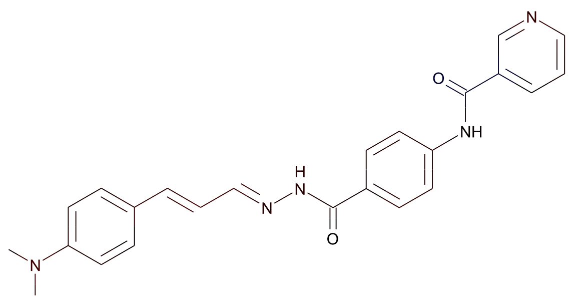

$C_{24}H_{23}N_5O_2$
Molecular Weight: 413.47172
ALogP: 2.839
Rotatable Bonds: 7
Acceptors: 5
Donors: 2

Model Prediction

Prediction: Carcinogen
Probability: 0.486
Enrichment: 1.45
Bayesian Score: 3.97
Mahalanobis Distance: 16.9
Mahalanobis Distance p-value: 5.5e-011

Prediction: Positive if the Bayesian score is above the estimated best cutoff value from minimizing the false positive and false negative rate.
Probability: The esimated probability that the sample is in the positive category. This assumes that the Bayesian score follows a normal distribution and is different from the prediction using a cutoff.
Enrichment: An estimate of enrichment, that is, the increased likelihood (versus random) of this sample being in the category.
Bayesian Score: The standard Laplacian-modified Bayesian score.
Mahalanobis Distance: The Mahalanobis distance (MD) is the distance to the center of the training data. The larger the MD, the less trustworthy the prediction.
Mahalanobis Distance p-value: The p-value gives the fraction of training data with an MD greater than or equal to the one for the given sample, assuming normally distributed data. The smaller the p-value, the less trustworthy the prediciton. For highly non-normal X properties (e.g., fingerprints), the MD p-value is wildly inaccurate.

Structural Similar Compounds

Name	Bicalutamide	Flecainide	Cisapride
Structure	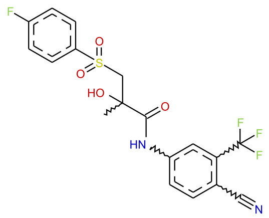	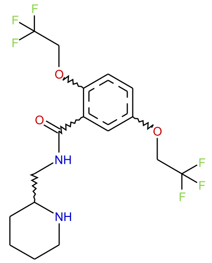	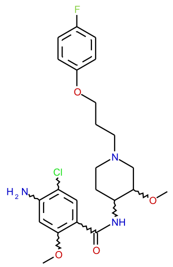
Actual Endpoint	Carcinogen	Non-Carcinogen	Non-Carcinogen
Predicted Endpoint	Carcinogen	Non-Carcinogen	Non-Carcinogen
Distance	0.583	0.634	0.635
Reference	US FDA (Centre for Drug Eval.& Res./Off. Testing & Res.) Sept. 1997	US FDA (Centre for Drug Eval.& Res./Off. Testing & Res.) Sept. 1997	US FDA (Centre for Drug Eval.& Res./Off. Testing & Res.) Sept. 1997

Model Applicability

Unknown features are fingerprint features in the query molecule, but not found or appearing too infrequently in the training set.

1. OPS PC12 out of range. Value: -4.5678. Training min, max, SD, explained variance: -3.9196, 6.4101, 1.581, 0.0233.

Feature Contribution

Top features for positive contribution

Fingerprint	Bit/Smiles	Feature Structure	Score	Carcinogen in training set
SCFP_6	-347048986	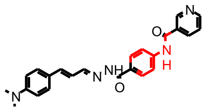 [*]C(=[*])N[c]1:[cH]:[cH]:[*]:[cH]:[cH]:1	0.615	5 out of 7

SCFP_6	-1971137145	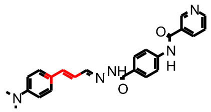 [*]\C=C\[c](:[*]):[*]	0.434	5 out of 9
SCFP_6	-1977229858	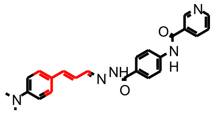 [*]=C\C=C\[c](:[cH]:[*]):[cH]:[*]	0.425	2 out of 3
**Top Features for negative contribution**				
**Fingerprint**	**Bit/Smiles**	**Feature Structure**	**Score**	**Carcinogen in training set**
SCFP_6	1257084377	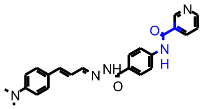 [*]NC(=O)[c](:[*]):[*]	-0.436	4 out of 21
SCFP_6	2096901122	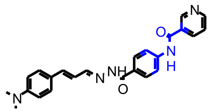 [*]:[cH]:[c](NC(=O)[c](:[*]):[*]):[cH]:[*]	-0.278	0 out of 1
SCFP_6	124026986	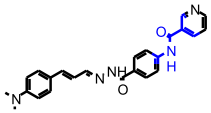 [*]:[cH]:[c](:[cH]:[*])C(=O)N[c](:[*]):[*]	-0.278	0 out of 1

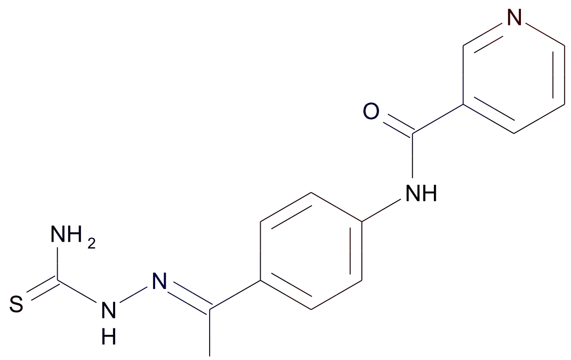

$C_{15}H_{15}N_5OS$
Molecular Weight: 313.3775
ALogP: 1.414
Rotatable Bonds: 5
Acceptors: 4
Donors: 3

Model Prediction

Prediction: Carcinogen
Probability: 0.361
Enrichment: 1.08
Bayesian Score: 0.149
Mahalanobis Distance: 15.1
Mahalanobis Distance p-value: 3.35e-007

Prediction: Positive if the Bayesian score is above the estimated best cutoff value from minimizing the false positive and false negative rate.
Probability: The esimated probability that the sample is in the positive category. This assumes that the Bayesian score follows a normal distribution and is different from the prediction using a cutoff.
Enrichment: An estimate of enrichment, that is, the increased likelihood (versus random) of this sample being in the category.
Bayesian Score: The standard Laplacian-modified Bayesian score.
Mahalanobis Distance: The Mahalanobis distance (MD) is the distance to the center of the training data. The larger the MD, the less trustworthy the prediction.
Mahalanobis Distance p-value: The p-value gives the fraction of training data with an MD greater than or equal to the one for the given sample, assuming normally distributed data. The smaller the p-value, the less trustworthy the prediciton. For highly non-normal X properties (e.g., fingerprints), the MD p-value is wildly inaccurate.

Structural Similar Compounds

Name	Chloramphenicol	Furosemide	Tolbutamide
Structure	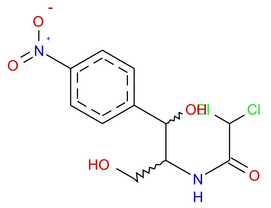	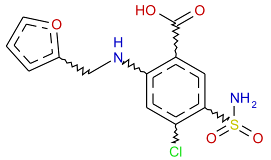	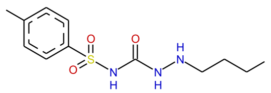
Actual Endpoint	Non-Carcinogen	Carcinogen	Non-Carcinogen
Predicted Endpoint	Non-Carcinogen	Carcinogen	Non-Carcinogen
Distance	0.567	0.567	0.576
Reference	US FDA (Centre for Drug Eval.& Res./Off. Testing & Res.) Sept. 1997	US FDA (Centre for Drug Eval.& Res./Off. Testing & Res.) Sept. 1997	US FDA (Centre for Drug Eval.& Res./Off. Testing & Res.) Sept. 1997

Model Applicability

Unknown features are fingerprint features in the query molecule, but not found or appearing too infrequently in the training set.

1. All properties and OPS components are within expected ranges.

Feature Contribution

Top features for positive contribution

Fingerprint	Bit/Smiles	Feature Structure	Score	Carcinogen in training set
SCFP_6	-347048986	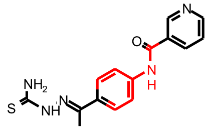 [*]C(=[*])N[c]1:[cH]:[cH]:[*]:[cH]:[cH]:1	0.615	5 out of 7

SCFP_6	-105808146	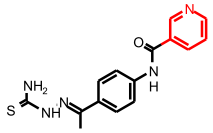 [*][c]1:[cH]:[cH]:[cH]:n:[cH]:1	0.415	1 out of 1
SCFP_6	-758850909	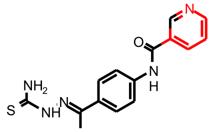 [*][c]1:[*]:n:[cH]:[cH]:[cH]:1	0.355	5 out of 10
**Top Features for negative contribution**				
**Fingerprint**	**Bit/Smiles**	**Feature Structure**	**Score**	**Carcinogen in training set**
SCFP_6	1257084377	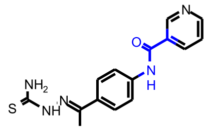 [*]NC(=O)[c](:[*]):[*]	-0.436	4 out of 21
SCFP_6	384861283	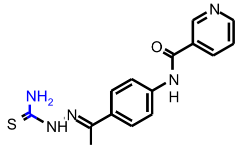 [*]C(=[*])N	-0.38	1 out of 6
SCFP_6	827404948	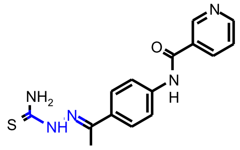 [*]C(=[*])NN=[*]	-0.278	0 out of 1

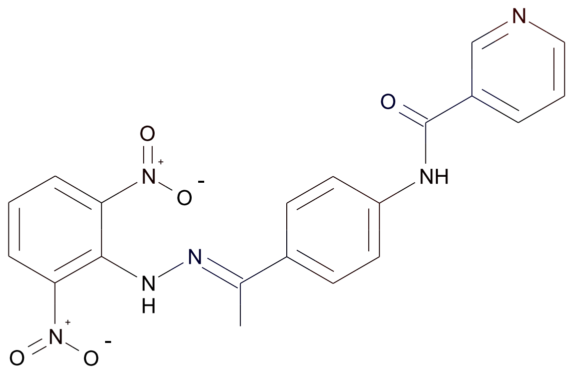

$C_{20}H_{16}N_6O_5$
Molecular Weight: 420.37824
ALogP: 2.852
Rotatable Bonds: 7
Acceptors: 8
Donors: 2

Model Prediction

Prediction: Carcinogen
Probability: 0.386
Enrichment: 1.16
Bayesian Score: 0.995
Mahalanobis Distance: 20
Mahalanobis Distance p-value: 1.22e-018

Prediction: Positive if the Bayesian score is above the estimated best cutoff value from minimizing the false positive and false negative rate.
Probability: The esimated probability that the sample is in the positive category. This assumes that the Bayesian score follows a normal distribution and is different from the prediction using a cutoff.
Enrichment: An estimate of enrichment, that is, the increased likelihood (versus random) of this sample being in the category.
Bayesian Score: The standard Laplacian-modified Bayesian score.
Mahalanobis Distance: The Mahalanobis distance (MD) is the distance to the center of the training data. The larger the MD, the less trustworthy the prediction.
Mahalanobis Distance p-value: The p-value gives the fraction of training data with an MD greater than or equal to the one for the given sample, assuming normally distributed data. The smaller the p-value, the less trustworthy the prediciton. For highly non-normal X properties (e.g., fingerprints), the MD p-value is wildly inaccurate.

Structural Similar Compounds

Name	Sulfasalazine	Nedocromil	Nisoldipine
Structure	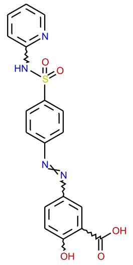	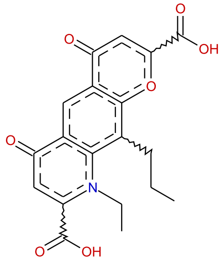	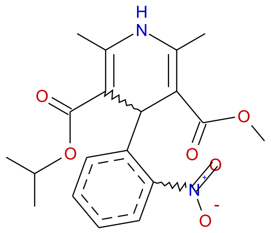
Actual Endpoint	Carcinogen	Non-Carcinogen	Non-Carcinogen
Predicted Endpoint	Carcinogen	Non-Carcinogen	Carcinogen
Distance	0.604	0.619	0.666
Reference	US FDA (Centre for Drug Eval.& Res./Off. Testing & Res.) Sept. 1997	US FDA (Centre for Drug Eval.& Res./Off. Testing & Res.) Sept. 1997	US FDA (Centre for Drug Eval.& Res./Off. Testing & Res.) Sept. 1997

Model Applicability

Unknown features are fingerprint features in the query molecule, but not found or appearing too infrequently in the training set.

1. OPS PC12 out of range. Value: -4.5928. Training min, max, SD, explained variance: -3.9196, 6.4101, 1.581, 0.0233.

Feature Contribution

Top features for positive contribution

Fingerprint	Bit/Smiles	Feature Structure	Score	Carcinogen in training set
SCFP_6	-347048986	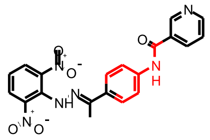 [*]C(=[*])N[c]1:[cH]:[cH]:[*]:[cH]:[cH]:1	0.615	5 out of 7

SCFP_6	-105808146	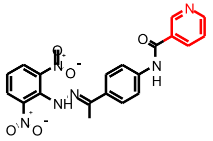 [*][c]1:[cH]:[cH]:[cH]:n:[cH]:1	0.415	1 out of 1
SCFP_6	1311339974	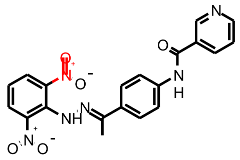 [*][N+](=O)[*]	0.405	13 out of 26
**Top Features for negative contribution**				
**Fingerprint**	**Bit/Smiles**	**Feature Structure**	**Score**	**Carcinogen in training set**
SCFP_6	1257084377	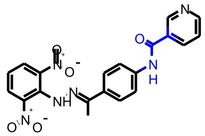 [*]NC(=O)[c](:[*]):[*]	-0.436	4 out of 21
SCFP_6	-331724199	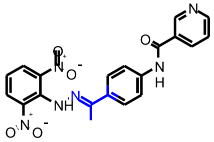 [*]\N=C(/C)\[c](:[*]):[*]	-0.278	0 out of 1
SCFP_6	124026986	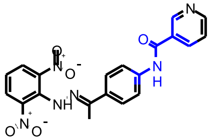 [*]:[cH]:[c](:[cH]:[*])C(=O)N[c](:[*]):[*]	-0.278	0 out of 1

Sorafenib

TOPKAT_Rat_Male_FDA_None_vs_Carcinogen

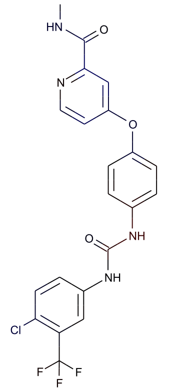

$C_{21}H_{16}ClF_3N_4O_3$
Molecular Weight: 464.82494
ALogP: 4.175
Rotatable Bonds: 6
Acceptors: 4
Donors: 3

Model Prediction

Prediction: Non-Carcinogen

Probability: 0.293

Enrichment: 0.878

Bayesian Score: -2.4

Mahalanobis Distance: 17.6

Mahalanobis Distance p-value: 1.1e-012

Prediction: Positive if the Bayesian score is above the estimated best cutoff value from minimizing the false positive and false negative rate.
Probability: The esimated probability that the sample is in the positive category. This assumes that the Bayesian score follows a normal distribution and is different from the prediction using a cutoff.
Enrichment: An estimate of enrichment, that is, the increased likelihood (versus random) of this sample being in the category.
Bayesian Score: The standard Laplacian-modified Bayesian score.
Mahalanobis Distance: The Mahalanobis distance (MD) is the distance to the center of the training data. The larger the MD, the less trustworthy the prediction.
Mahalanobis Distance p-value: The p-value gives the fraction of training data with an MD greater than or equal to the one for the given sample, assuming normally distributed data. The smaller the p-value, the less trustworthy the prediciton. For highly non-normal X properties (e.g., fingerprints), the MD p-value is wildly inaccurate.

Structural Similar Compounds

Name	Glyburide	Glimepride	Fluvastatin
Structure	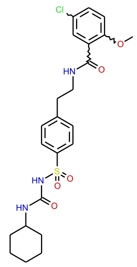	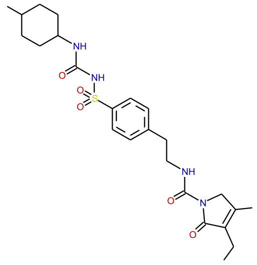	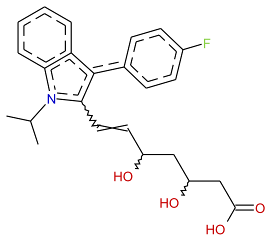
Actual Endpoint	Non-Carcinogen	Non-Carcinogen	Carcinogen
Predicted Endpoint	Non-Carcinogen	Non-Carcinogen	Carcinogen
Distance	0.593	0.600	0.615
Reference	US FDA (Centre for Drug Eval.& Res./Off. Testing & Res.) Sept. 1997	US FDA (Centre for Drug Eval.& Res./Off. Testing & Res.) Sept. 1997	US FDA (Centre for Drug Eval.& Res./Off. Testing & Res.) Sept. 1997

Model Applicability

Unknown features are fingerprint features in the query molecule, but not found or appearing too infrequently in the training set.

1. All properties and OPS components are within expected ranges.

Feature Contribution

Top features for positive contribution

Fingerprint	Bit/Smiles	Feature Structure	Score	Carcinogen in training set
SCFP_6	-347048986	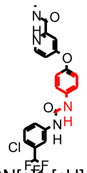 [*]C(=[*])N[c]1:[cH]:[cH]:[*]:[cH]:[cH]:1	0.615	5 out of 7

SCFP_6	-754059116	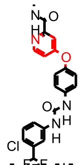 [*]O[c]1:[cH]:[*]:n:[cH]:[cH]:1	0.415	1 out of 1
SCFP_6	-347281112	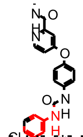 [*]N[c]1:[cH]:[*]:[c]([*]):[c](:[cH]:1)C([*])([*])[*]	0.273	2 out of 4
**Top Features for negative contribution**				
**Fingerprint**	**Bit/Smiles**	**Feature Structure**	**Score**	**Carcinogen in training set**
SCFP_6	-827073191	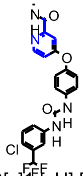 [*]C(=[*])[c]1:[cH]:[*]:[cH]:[cH]:n:1	-0.674	0 out of 3
SCFP_6	-488587948	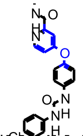 [*][c]1:[*]:[cH]:[cH]:[c](O[c](:[*]):[*]):[cH]:1	-0.496	0 out of 2
SCFP_6	-975241316	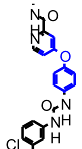 [*][c]1:[cH]:[cH]:[c](O[c](:[cH]:[*]):[cH]:[*]):[cH]:[cH]:1	-0.496	0 out of 2

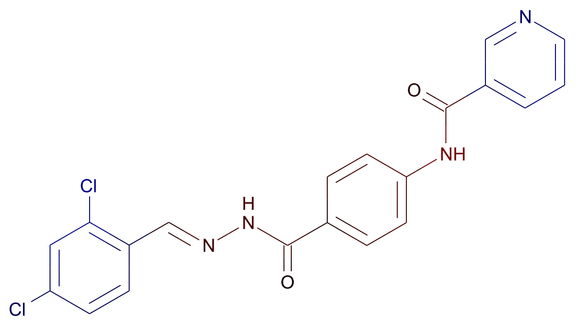

$C_{20}H_{14}Cl_2N_4O_2$
Molecular Weight: 413.25676
ALogP: 3.646
Rotatable Bonds: 5
Acceptors: 4
Donors: 2

Model Prediction

Prediction: Single-Carcinogen
Probability: 0.535
Enrichment: 1.29
Bayesian Score: -2.02
Mahalanobis Distance: 11.7
Mahalanobis Distance p-value: 0.00711

Prediction: Positive if the Bayesian score is above the estimated best cutoff value from minimizing the false positive and false negative rate.
Probability: The esimated probability that the sample is in the positive category. This assumes that the Bayesian score follows a normal distribution and is different from the prediction using a cutoff.
Enrichment: An estimate of enrichment, that is, the increased likelihood (versus random) of this sample being in the category.
Bayesian Score: The standard Laplacian-modified Bayesian score.
Mahalanobis Distance: The Mahalanobis distance (MD) is the distance to the center of the training data. The larger the MD, the less trustworthy the prediction.
Mahalanobis Distance p-value: The p-value gives the fraction of training data with an MD greater than or equal to the one for the given sample, assuming normally distributed data. The smaller the p-value, the less trustworthy the prediciton. For highly non-normal X properties (e.g., fingerprints), the MD p-value is wildly inaccurate.

Structural Similar Compounds

Name	Bicalutamide	Doxefazepam	Lansoprazole
Structure	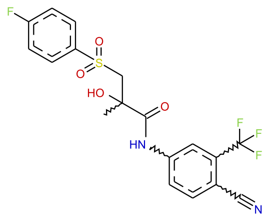	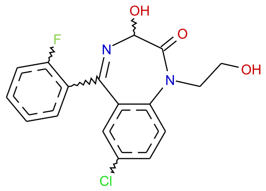	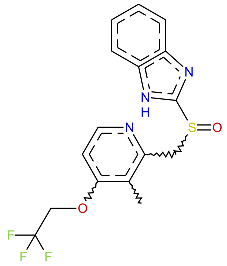
Actual Endpoint	Multiple-Carcinogen	Single-Carcinogen	Multiple-Carcinogen
Predicted Endpoint	Multiple-Carcinogen	Single-Carcinogen	Multiple-Carcinogen
Distance	0.625	0.636	0.688
Reference	US FDA (Centre for Drug Eval.& Res./Off. Testing & Res.) Sept. 1997	US FDA (Centre for Drug Eval.& Res./Off. Testing & Res.) Sept. 1997	US FDA (Centre for Drug Eval.& Res./Off. Testing & Res.) Sept. 1997

Model Applicability

Unknown features are fingerprint features in the query molecule, but not found or appearing too infrequently in the training set.

1. All properties and OPS components are within expected ranges.

Feature Contribution

Top features for positive contribution				
**Fingerprint**	**Bit/Smiles**	**Feature Structure**	**Score**	**Multiple-Carcinogen in training set**
SCFP_8	-347048986	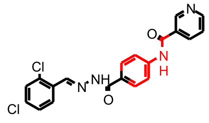 [*]C(=[*])N[c]1:[cH]:[cH]:[*]:[cH]:[cH]:1	0.574	4 out of 5

SCFP_8	1631845520	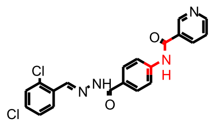 [*]C(=[*])N[c](:[*]):[*]	0.495	6 out of 9
SCFP_8	1257084377	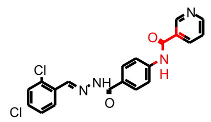 [*]NC(=O)[c](:[*]):[*]	0.489	3 out of 4

Top Features for negative contribution

Fingerprint	Bit/Smiles	Feature Structure	Score	Multiple-Carcinogen in training set
SCFP_8	-758850909	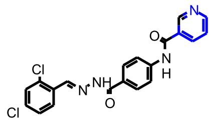 [*][c]1:[*]:n:[cH]:[cH]:[cH]:1	-1.04	0 out of 5
SCFP_8	-601571304	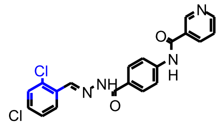 [*][c](:[*]):[c](Cl):[cH]:[*]	-0.707	2 out of 14
SCFP_8	-52074512	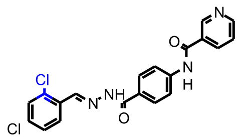 [*]:[c](:[*])Cl	-0.707	2 out of 14

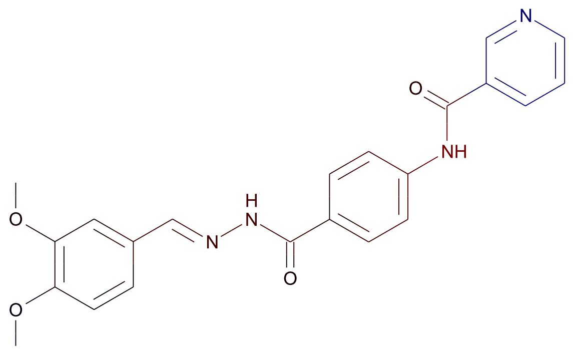

$C_{22}H_{20}N_4O_4$
Molecular Weight: 404.4186
ALogP: 2.284
Rotatable Bonds: 7
Acceptors: 6
Donors: 2

Model Prediction

Prediction: Single-Carcinogen
Probability: 0.557
Enrichment: 1.35
Bayesian Score: -0.609
Mahalanobis Distance: 20.1
Mahalanobis Distance p-value: 2.4e-010

Prediction: Positive if the Bayesian score is above the estimated best cutoff value from minimizing the false positive and false negative rate.
Probability: The esimated probability that the sample is in the positive category. This assumes that the Bayesian score follows a normal distribution and is different from the prediction using a cutoff.
Enrichment: An estimate of enrichment, that is, the increased likelihood (versus random) of this sample being in the category.
Bayesian Score: The standard Laplacian-modified Bayesian score.
Mahalanobis Distance: The Mahalanobis distance (MD) is the distance to the center of the training data. The larger the MD, the less trustworthy the prediction.
Mahalanobis Distance p-value: The p-value gives the fraction of training data with an MD greater than or equal to the one for the given sample, assuming normally distributed data. The smaller the p-value, the less trustworthy the prediciton. For highly non-normal X properties (e.g., fingerprints), the MD p-value is wildly inaccurate.

Structural Similar Compounds

Name	Bicalutamide	Moricizine	Isradipine
Structure	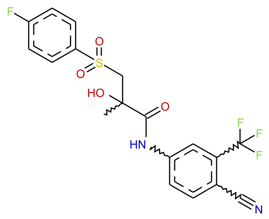	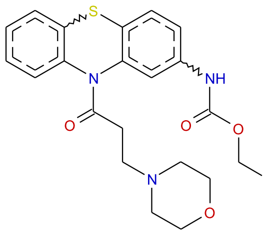	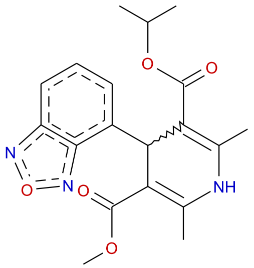
Actual Endpoint	Multiple-Carcinogen	Single-Carcinogen	Single-Carcinogen
Predicted Endpoint	Multiple-Carcinogen	Single-Carcinogen	Single-Carcinogen
Distance	0.605	0.675	0.693
Reference	US FDA (Centre for Drug Eval.& Res./Off. Testing & Res.) Sept. 1997	US FDA (Centre for Drug Eval.& Res./Off. Testing & Res.) Sept. 1997	US FDA (Centre for Drug Eval.& Res./Off. Testing & Res.) Sept. 1997

Model Applicability

Unknown features are fingerprint features in the query molecule, but not found or appearing too infrequently in the training set.

1. OPS PC4 out of range. Value: 6.2282. Training min, max, SD, explained variance: -6.1092, 5.1042, 2.173, 0.0642.

Feature Contribution

Top features for positive contribution

Fingerprint	Bit/Smiles	Feature Structure	Score	Multiple-Carcinogen in training set
SCFP_8	-347048986	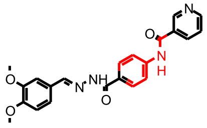 [*]C(=[*])N[c]1:[cH]:[cH]:[*]:[cH]:[cH]:1	0.574	4 out of 5

SCFP_8	1631845520	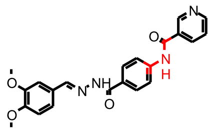 [*]C(=[*])N[c](:[*]):[*]	0.495	6 out of 9
SCFP_8	1257084377	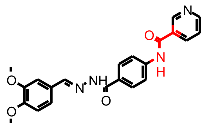 [*]NC(=O)[c](:[*]):[*]	0.489	3 out of 4

Top Features for negative contribution

Fingerprint	Bit/Smiles	Feature Structure	Score	Multiple-Carcinogen in training set
SCFP_8	-758850909	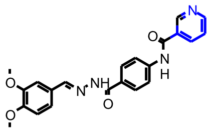 [*][c]1:[*]:n:[cH]:[cH]:[cH]:1	-1.04	0 out of 5
SCFP_8	-937094999	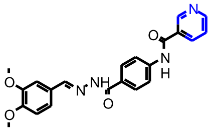 [*]1:[cH]:[cH]:[cH]:n:[cH]:1	-0.463	1 out of 6
SCFP_8	136239834	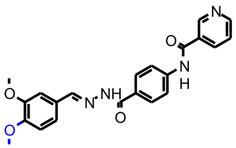 [*]OC	-0.358	3 out of 13

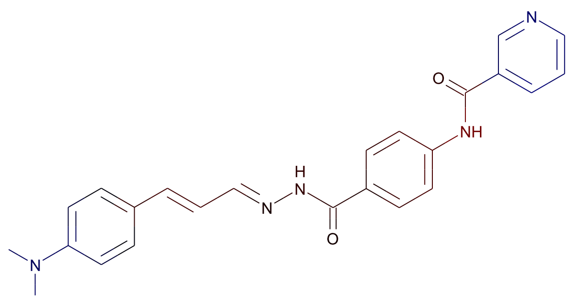

$C_{24}H_{23}N_5O_2$

Molecular Weight: 413.47172

ALogP: 2.839

Rotatable Bonds: 7

Acceptors: 5

Donors: 2

Model Prediction

Prediction: Single-Carcinogen

Probability: 0.553

Enrichment: 1.34

Bayesian Score: -0.946

Mahalanobis Distance: 14.1

Mahalanobis Distance p-value: 7.2e-005

Prediction: Positive if the Bayesian score is above the estimated best cutoff value from minimizing the false positive and false negative rate.
Probability: The esimated probability that the sample is in the positive category. This assumes that the Bayesian score follows a normal distribution and is different from the prediction using a cutoff.
Enrichment: An estimate of enrichment, that is, the increased likelihood (versus random) of this sample being in the category.
Bayesian Score: The standard Laplacian-modified Bayesian score.
Mahalanobis Distance: The Mahalanobis distance (MD) is the distance to the center of the training data. The larger the MD, the less trustworthy the prediction.
Mahalanobis Distance p-value: The p-value gives the fraction of training data with an MD greater than or equal to the one for the given sample, assuming normally distributed data. The smaller the p-value, the less trustworthy the prediciton. For highly non-normal X properties (e.g., fingerprints), the MD p-value is wildly inaccurate.

Structural Similar Compounds

Name	Bicalutamide	Moricizine	Felodipine
Structure	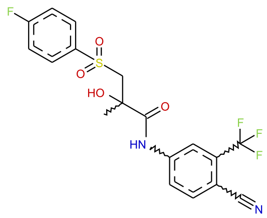	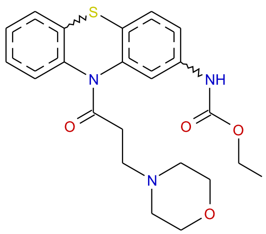	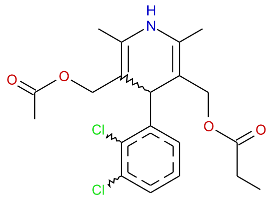
Actual Endpoint	Multiple-Carcinogen	Single-Carcinogen	Single-Carcinogen
Predicted Endpoint	Multiple-Carcinogen	Single-Carcinogen	Single-Carcinogen
Distance	0.590	0.686	0.698
Reference	US FDA (Centre for Drug Eval.& Res./Off. Testing & Res.) Sept. 1997	US FDA (Centre for Drug Eval.& Res./Off. Testing & Res.) Sept. 1997	US FDA (Centre for Drug Eval.& Res./Off. Testing & Res.) Sept. 1997

Model Applicability

Unknown features are fingerprint features in the query molecule, but not found or appearing too infrequently in the training set.

1. OPS PC14 out of range. Value: 3.4917. Training min, max, SD, explained variance: -3.4626, 3.2359, 1.286, 0.0225.

Feature Contribution

Top features for positive contribution				
**Fingerprint**	**Bit/Smiles**	**Feature Structure**	**Score**	**Multiple-Carcinogen in training set**
SCFP_8	-347048986	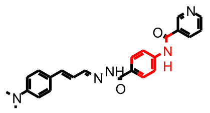 [*]C(=[*])N[c]1:[cH]:[cH]:[*]:[cH]:[cH]:1	0.574	4 out of 5

SCFP_8	-1977229858	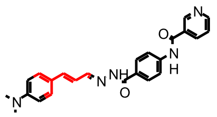<br>[*]=C\C=C\[c](:[cH]:[*]):[cH]:[*]	0.553	2 out of 2
SCFP_8	1631845520	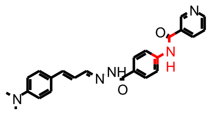<br>[*]C(=[*])N[c](:[*]): [*]	0.495	6 out of 9
**Top Features for negative contribution**				
**Fingerprint**	**Bit/Smiles**	**Feature Structure**	**Score**	**Multiple-Carcinogen in training set**
SCFP_8	-758850909	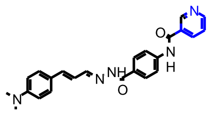<br>[*][c]1:[*]:n:[cH]:[c H]:[cH]:1	-1.04	0 out of 5
SCFP_8	-299436502	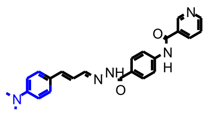<br>CN(C)[c]1:[cH]:[cH]:[*]:[cH]:[cH]:1	-0.737	0 out of 3
SCFP_8	-1853624961	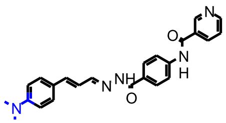<br>[*]:[c](:[*])N(C)C	-0.669	1 out of 8

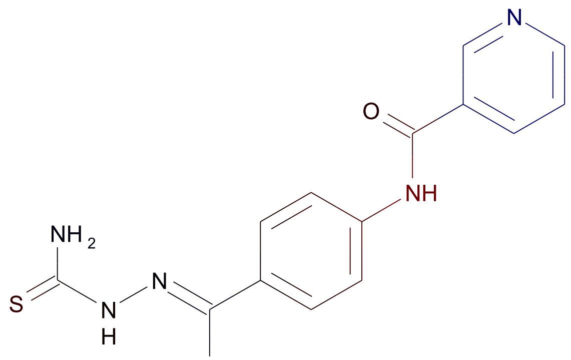

$C_{15}H_{15}N_5OS$
Molecular Weight: 313.3775
ALogP: 1.414
Rotatable Bonds: 5
Acceptors: 4
Donors: 3

Model Prediction

Prediction: Multiple-Carcinogen
Probability: 0.568
Enrichment: 1.37
Bayesian Score: 0.852
Mahalanobis Distance: 14.1
Mahalanobis Distance p-value: 6.91e-005

Prediction: Positive if the Bayesian score is above the estimated best cutoff value from minimizing the false positive and false negative rate.
Probability: The esimated probability that the sample is in the positive category. This assumes that the Bayesian score follows a normal distribution and is different from the prediction using a cutoff.
Enrichment: An estimate of enrichment, that is, the increased likelihood (versus random) of this sample being in the category.
Bayesian Score: The standard Laplacian-modified Bayesian score.
Mahalanobis Distance: The Mahalanobis distance (MD) is the distance to the center of the training data. The larger the MD, the less trustworthy the prediction.
Mahalanobis Distance p-value: The p-value gives the fraction of training data with an MD greater than or equal to the one for the given sample, assuming normally distributed data. The smaller the p-value, the less trustworthy the prediciton. For highly non-normal X properties (e.g., fingerprints), the MD p-value is wildly inaccurate.

Structural Similar Compounds

Name	Furosemide	Torsemide	Sulfamethazine
Structure	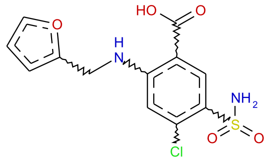	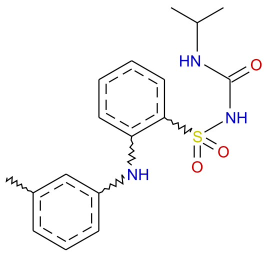	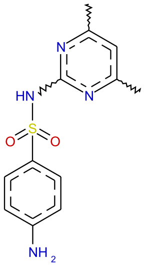
Actual Endpoint	Single-Carcinogen	Multiple-Carcinogen	Single-Carcinogen
Predicted Endpoint	Single-Carcinogen	Multiple-Carcinogen	Single-Carcinogen
Distance	0.577	0.707	0.717
Reference	US FDA (Centre for Drug Eval.& Res./Off. Testing & Res.) Sept. 1997	US FDA (Centre for Drug Eval.& Res./Off. Testing & Res.) Sept. 1997	US FDA (Centre for Drug Eval.& Res./Off. Testing & Res.) Sept. 1997

Model Applicability

Unknown features are fingerprint features in the query molecule, but not found or appearing too infrequently in the training set.

1. All properties and OPS components are within expected ranges.

Feature Contribution

Top features for positive contribution

Fingerprint	Bit/Smiles	Feature Structure	Score	Multiple-Carcinogen in training set
SCFP_8	-347048986	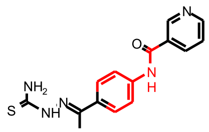 [*]C(=[*])N[c]1:[cH]:[cH]:[*]:[cH]:[cH]:1	0.574	4 out of 5

SCFP_8	1435188938	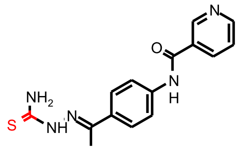 [*]C(=S)[*]	0.553	2 out of 2
SCFP_8	18	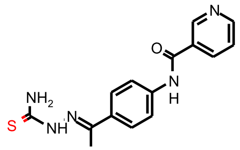 [*]=S	0.553	2 out of 2
**Top Features for negative contribution**				
**Fingerprint**	**Bit/Smiles**	**Feature Structure**	**Score**	**Multiple-Carcinogen in training set**
SCFP_8	-758850909	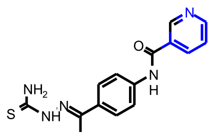 [*][c]1:[*]:n:[cH]:[cH]:[cH]:1	-1.04	0 out of 5
SCFP_8	-937094999	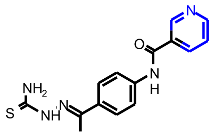 [*]1:[cH]:[cH]:[cH]:n:[cH]:1	-0.463	1 out of 6
SCFP_8	136627117	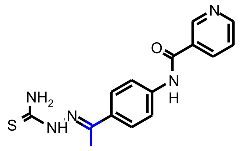 [*]C(=[*])C	-0.41	4 out of 18

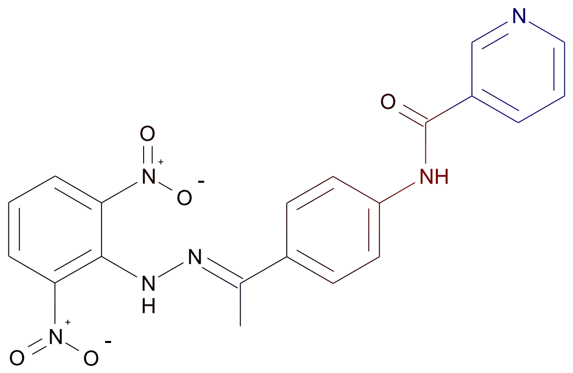

$C_{20}H_{16}N_6O_5$
Molecular Weight: 420.37824
ALogP: 2.852
Rotatable Bonds: 7
Acceptors: 8
Donors: 2

Model Prediction

Prediction: Single-Carcinogen
Probability: 0.542
Enrichment: 1.31
Bayesian Score: -1.65
Mahalanobis Distance: 13.1
Mahalanobis Distance p-value: 0.000507

Prediction: Positive if the Bayesian score is above the estimated best cutoff value from minimizing the false positive and false negative rate.
Probability: The esimated probability that the sample is in the positive category. This assumes that the Bayesian score follows a normal distribution and is different from the prediction using a cutoff.
Enrichment: An estimate of enrichment, that is, the increased likelihood (versus random) of this sample being in the category.
Bayesian Score: The standard Laplacian-modified Bayesian score.
Mahalanobis Distance: The Mahalanobis distance (MD) is the distance to the center of the training data. The larger the MD, the less trustworthy the prediction.
Mahalanobis Distance p-value: The p-value gives the fraction of training data with an MD greater than or equal to the one for the given sample, assuming normally distributed data. The smaller the p-value, the less trustworthy the prediciton. For highly non-normal X properties (e.g., fingerprints), the MD p-value is wildly inaccurate.

Structural Similar Compounds

Name	Sulfasalazine	Isradipine	Nimodipine
Structure	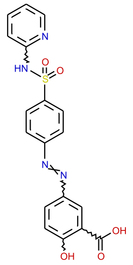	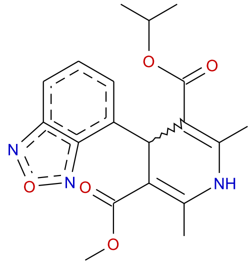	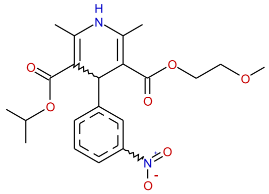
Actual Endpoint	Single-Carcinogen	Single-Carcinogen	Single-Carcinogen
Predicted Endpoint	Single-Carcinogen	Single-Carcinogen	Single-Carcinogen
Distance	0.656	0.743	0.760
Reference	US FDA (Centre for Drug Eval.& Res./Off. Testing & Res.) Sept. 1997	US FDA (Centre for Drug Eval.& Res./Off. Testing & Res.) Sept. 1997	US FDA (Centre for Drug Eval.& Res./Off. Testing & Res.) Sept. 1997

Model Applicability

Unknown features are fingerprint features in the query molecule, but not found or appearing too infrequently in the training set.

1. OPS PC14 out of range. Value: 3.3935. Training min, max, SD, explained variance: -3.4626, 3.2359, 1.286, 0.0225.

Feature Contribution

Top features for positive contribution

Fingerprint	Bit/Smiles	Feature Structure	Score	Multiple-Carcinogen in training set
SCFP_8	-347048986	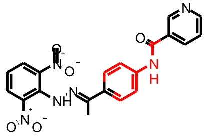 [*]C(=[*])N[c]1:[cH]:[cH]:[*]:[cH]:[cH]:1	0.574	4 out of 5

SCFP_8	1631845520	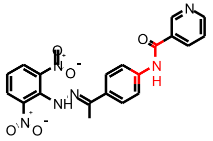 [*]C(=[*])N[c](:[*]):[*]	0.495	6 out of 9
SCFP_8	1257084377	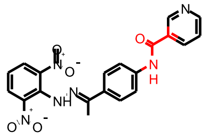 [*]NC(=O)[c](:[*]):[*]	0.489	3 out of 4

Top Features for negative contribution

Fingerprint	Bit/Smiles	Feature Structure	Score	Multiple-Carcinogen in training set
SCFP_8	-758850909	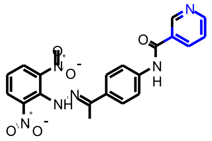 [*][c]1:[*]:n:[cH]:[cH]:[cH]:1	-1.04	0 out of 5
SCFP_8	-937094999	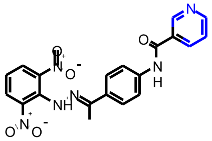 [*]1:[cH]:[cH]:[cH]:n:[cH]:1	-0.463	1 out of 6
SCFP_8	136627117	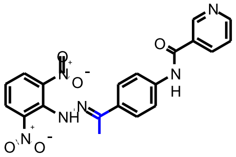 [*]C(=[*])C	-0.41	4 out of 18

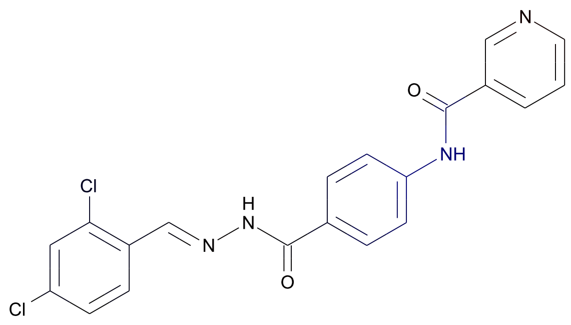

$C_{20}H_{14}Cl_2N_4O_2$
Molecular Weight: 413.25676
ALogP: 3.646
Rotatable Bonds: 5
Acceptors: 4
Donors: 2

Model Prediction

Prediction: Non-Irritant
Probability: 0.844
Enrichment: 0.916
Bayesian Score: -3.25
Mahalanobis Distance: 6.36
Mahalanobis Distance p-value: 1

Prediction: Positive if the Bayesian score is above the estimated best cutoff value from minimizing the false positive and false negative rate.
Probability: The esimated probability that the sample is in the positive category. This assumes that the Bayesian score follows a normal distribution and is different from the prediction using a cutoff.
Enrichment: An estimate of enrichment, that is, the increased likelihood (versus random) of this sample being in the category.
Bayesian Score: The standard Laplacian-modified Bayesian score.
Mahalanobis Distance: The Mahalanobis distance (MD) is the distance to the center of the training data. The larger the MD, the less trustworthy the prediction.
Mahalanobis Distance p-value: The p-value gives the fraction of training data with an MD greater than or equal to the one for the given sample, assuming normally distributed data. The smaller the p-value, the less trustworthy the prediciton. For highly non-normal X properties (e.g., fingerprints), the MD p-value is wildly inaccurate.

Structural Similar Compounds

Name	5-Norbornene-2,3-dicarboxylic acid, 1,4,5,6,7,7-hexachloro-	Anthraquinone, 1,1'-iminodi-	1-Piperazineacetic acid, 4-(2-hydroxyethyl)-alpha-phenyl-, 2,6-xyly l ester, monohydrochloride
Structure	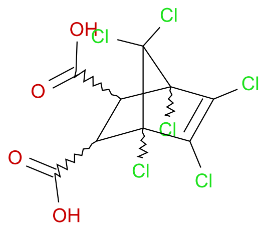	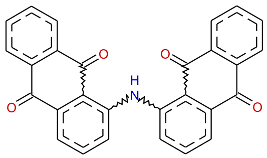	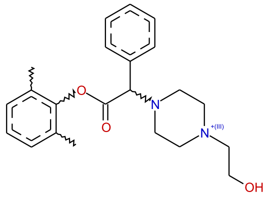
Actual Endpoint	Irritant	Irritant	Irritant
Predicted Endpoint	Irritant	Non-Irritant	Irritant
Distance	0.673	0.768	0.774
Reference	85JCAE "Prehled Prumyslove Toxikologie; Organicke Latky," Marhold, J., Prague , Czechoslovakia, Avicenum, 1986 Volume(issue)/page/year: -,581,1986	85JCAE "Prehled Prumyslove Toxikologie; Organicke Latky," Marhold, J., Prague , Czechoslovakia, Avicenum, 1986 Volume(issue)/page/year: -,735,1986	BCFAAI Bollettino Chimico Farmaceutico. (Societa Editoriale Farmaceutica, Vi a Ausonio 12, 20123 Milan, Italy) V.33- 1894- Volume(issue)/page/year: 107,3 10,1968

Model Applicability

Unknown features are fingerprint features in the query molecule, but not found or appearing too infrequently in the training set.

1. All properties and OPS components are within expected ranges.

Feature Contribution

Top features for positive contribution				
Fingerprint	Bit/Smiles	Feature Structure	Score	Irritant in training set

FCFP_12	-124655670	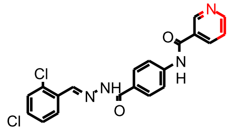 [*]:[cH]:[cH]:n:[*]	0.0821	13 out of 13
FCFP_12	-2100785893	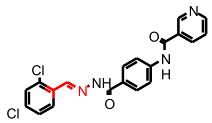 [*]\N=C\[c](:[*]):[*]	0.081	11 out of 11
FCFP_12	-1695756380	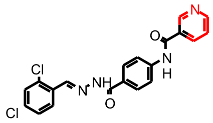 [*]1:[cH]:[cH]:[cH]:n :[cH]:1	0.0772	7 out of 7
**Top Features for negative contribution**				
**Fingerprint**	**Bit/Smiles**	**Feature Structure**	**Score**	**Irritant in training set**
FCFP_12	-1838187238	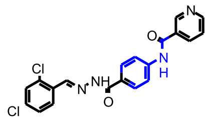 [*]C(=[*])N[c]1:[cH]: [cH]:[*]:[cH]:[cH]:1	-0.692	5 out of 12
FCFP_12	1294255210	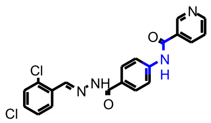 [*]C(=[*])N[c](:[*]): [*]	-0.486	12 out of 22

FCFP_12	-773983804	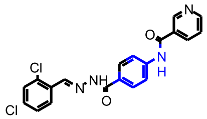 [*]N[c]1:[cH]:[*]:[c]([*]):[cH]:[cH]:1	-0.444	46 out of 79

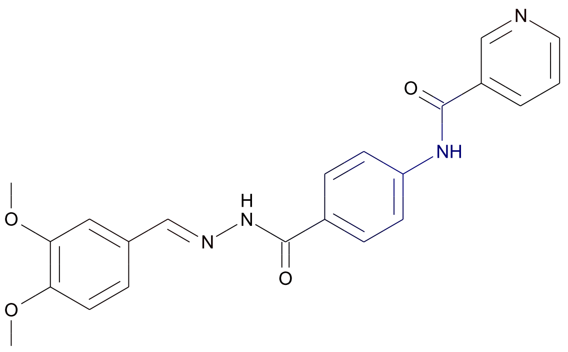

$C_{22}H_{20}N_4O_4$
Molecular Weight: 404.4186
ALogP: 2.284
Rotatable Bonds: 7
Acceptors: 6
Donors: 2

Model Prediction

Prediction: Non-Irritant
Probability: 0.931
Enrichment: 1.01
Bayesian Score: -2.31
Mahalanobis Distance: 6.34
Mahalanobis Distance p-value: 1

Prediction: Positive if the Bayesian score is above the estimated best cutoff value from minimizing the false positive and false negative rate.
Probability: The esimated probability that the sample is in the positive category. This assumes that the Bayesian score follows a normal distribution and is different from the prediction using a cutoff.
Enrichment: An estimate of enrichment, that is, the increased likelihood (versus random) of this sample being in the category.
Bayesian Score: The standard Laplacian-modified Bayesian score.
Mahalanobis Distance: The Mahalanobis distance (MD) is the distance to the center of the training data. The larger the MD, the less trustworthy the prediction.
Mahalanobis Distance p-value: The p-value gives the fraction of training data with an MD greater than or equal to the one for the given sample, assuming normally distributed data. The smaller the p-value, the less trustworthy the prediciton. For highly non-normal X properties (e.g., fingerprints), the MD p-value is wildly inaccurate.

Structural Similar Compounds

Name	Pregna-1,4-diene-3,20-dione, 21-(acetyloxy)-11-hydroxy-6-methyl-17- (1-oxopropoxy)-, (6-alpha,11-beta)-	2-Anthracenesulfonic acid, 1-amino-9,10-dihydro-9,10-dioxo-4-(2,4,6 -trimethylanilino)-, monosodium salt	Benzenesulfonamide, 4-amino-N-(5,6-dimethoxy-4-pyrimidinyl)-
Structure	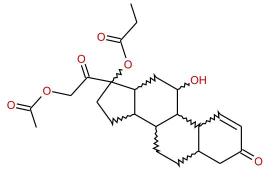	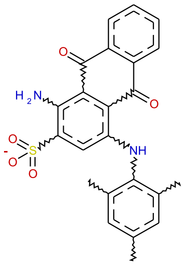	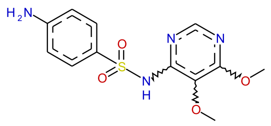
Actual Endpoint	Irritant	Irritant	Irritant
Predicted Endpoint	Irritant	Non-Irritant	Non-Irritant
Distance	0.724	0.778	0.800
Reference	YACHDS Yakuri to Chiryo. Pharmacology and Therapeutics. (Raifu Saiensu Shup pan K.K., 2-5-13, Yaesu, Chuo-ku, Tokyo 104, Japan) V.1- 1972- Volume(issue) /page/year: 19,3103,1991	85JCAE "Prehled Prumyslove Toxikologie; Organicke Latky," Marhold, J., Prague , Czechoslovakia, Avicenum, 1986 Volume(issue)/page/year: -,1327,1986	FCTXAV 14,307,76

Model Applicability

Unknown features are fingerprint features in the query molecule, but not found or appearing too infrequently in the training set.

1. All properties and OPS components are within expected ranges.

Feature Contribution

Top features for positive contribution				
**Fingerprint**	**Bit/Smiles**	**Feature Structure**	**Score**	**Irritant in training set**

FCFP_12	-124655670	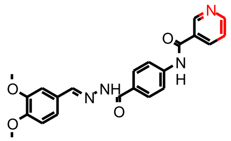<br>[*]:[cH]:[cH]:n:[*]	0.0821	13 out of 13
FCFP_12	-2100785893	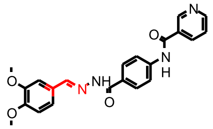<br>[*]\N=C\[c](:[*]):[*]	0.081	11 out of 11
FCFP_12	-1038421835	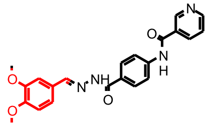<br>[*]O[c]1:[cH]:[c](C=[*]):[cH]:[cH]:[c]:1OC	0.0795	9 out of 9
**Top Features for negative contribution**				
**Fingerprint**	**Bit/Smiles**	**Feature Structure**	**Score**	**Irritant in training set**
FCFP_12	-1838187238	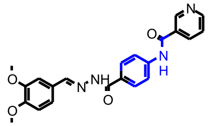<br>[*]C(=[*])N[c]1:[cH]:[cH]:[*]:[cH]:[cH]:1	-0.692	5 out of 12
FCFP_12	1294255210	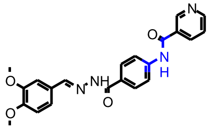<br>[*]C(=[*])N[c](:[*]):[*]	-0.486	12 out of 22

FCFP_12	-773983804	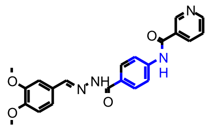 [*]N[c]1:[cH]:[*]:[c]([*]):[cH]:[cH]:1	-0.444	46 out of 79

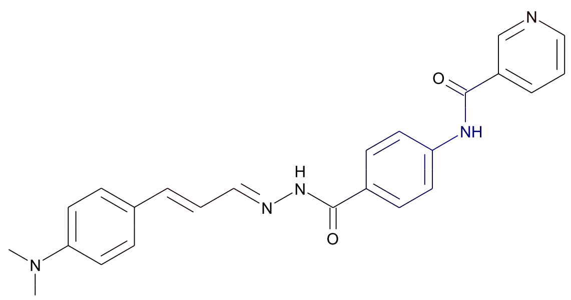

$C_{24}H_{23}N_5O_2$
Molecular Weight: 413.47172
ALogP: 2.839
Rotatable Bonds: 7
Acceptors: 5
Donors: 2

Model Prediction

Prediction: Non-Irritant

Probability: 0.935

Enrichment: 1.02

Bayesian Score: -2.23

Mahalanobis Distance: 9.15

Mahalanobis Distance p-value: 0.346

Prediction: Positive if the Bayesian score is above the estimated best cutoff value from minimizing the false positive and false negative rate.
Probability: The esimated probability that the sample is in the positive category. This assumes that the Bayesian score follows a normal distribution and is different from the prediction using a cutoff.
Enrichment: An estimate of enrichment, that is, the increased likelihood (versus random) of this sample being in the category.
Bayesian Score: The standard Laplacian-modified Bayesian score.
Mahalanobis Distance: The Mahalanobis distance (MD) is the distance to the center of the training data. The larger the MD, the less trustworthy the prediction.
Mahalanobis Distance p-value: The p-value gives the fraction of training data with an MD greater than or equal to the one for the given sample, assuming normally distributed data. The smaller the p-value, the less trustworthy the prediciton. For highly non-normal X properties (e.g., fingerprints), the MD p-value is wildly inaccurate.

Structural Similar Compounds

Name	5-Norbornene-2,3-dicarboxylic acid, 1,4,5,6,7,7-hexachloro-	1-Piperazineacetic acid, 4-(2-hydroxyethyl)-alpha-phenyl-, 2,6-xyly l ester, monohydrochloride	Pregna-1,4-diene-3,20-dione, 21-(acetyloxy)-11-hydroxy-6-methyl-17- (1-oxopropoxy)-, (6-alpha,11-beta)-
Structure	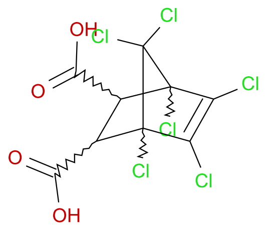	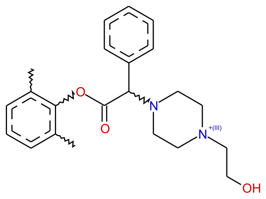	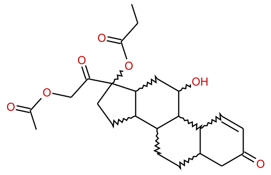
Actual Endpoint	Irritant	Irritant	Irritant
Predicted Endpoint	Irritant	Irritant	Irritant
Distance	0.756	0.788	0.808
Reference	85JCAE "Prehled Prumyslove Toxikologie; Organicke Latky," Marhold, J., Prague , Czechoslovakia, Avicenum, 1986 Volume(issue)/page/year: -,581,1986	BCFAAI Bollettino Chimico Farmaceutico. (Societa Editoriale Farmaceutica, Vi a Ausonio 12, 20123 Milan, Italy) V.33- 1894- Volume(issue)/page/year: 107,3 10,1968	YACHDS Yakuri to Chiryo. Pharmacology and Therapeutics. (Raifu Saiensu Shup pan K.K., 2-5-13, Yaesu, Chuo-ku, Tokyo 104, Japan) V.1-1972- Volume(issue) /page/year: 19,3103,1991

Model Applicability

Unknown features are fingerprint features in the query molecule, but not found or appearing too infrequently in the training set.

1. All properties and OPS components are within expected ranges.

Feature Contribution

Top features for positive contribution				
Fingerprint	Bit/Smiles	Feature Structure	Score	Irritant in training set

FCFP_12	-2100309237	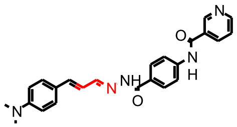 [*]\N=C\C=[*]	0.087	53 out of 53
FCFP_12	-146015125	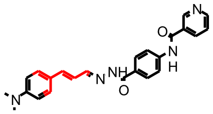 [*]=C\C=C\[c](:[cH]:[*]):[cH]:[*]	0.085	24 out of 24
FCFP_12	2011169140	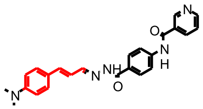 [*][c]1:[cH]:[cH]:[c](\C=C\C=[*]):[cH]:[cH]:1	0.0829	15 out of 15
**Top Features for negative contribution**				
**Fingerprint**	**Bit/Smiles**	**Feature Structure**	**Score**	**Irritant in training set**
FCFP_12	-1838187238	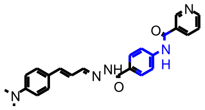 [*]C(=[*])N[c]1:[cH]:[cH]:[*]:[cH]:[cH]:1	-0.692	5 out of 12
FCFP_12	1294255210	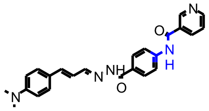 [*]C(=[*])N[c](:[*]):[*]	-0.486	12 out of 22

FCFP_12	-773983804	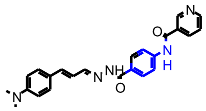 [*]N[c]1:[cH]:[*]:[c]([*]):[cH]:[cH]:1	-0.444	46 out of 79

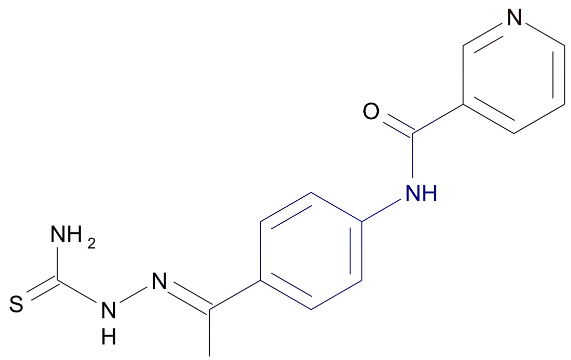

$C_{15}H_{15}N_5OS$
Molecular Weight: 313.3775
ALogP: 1.414
Rotatable Bonds: 5
Acceptors: 4
Donors: 3

Model Prediction

Prediction: Non-Irritant
Probability: 0.89
Enrichment: 0.966
Bayesian Score: -2.86
Mahalanobis Distance: 6.86
Mahalanobis Distance p-value: 0.998

Prediction: Positive if the Bayesian score is above the estimated best cutoff value from minimizing the false positive and false negative rate.
Probability: The esimated probability that the sample is in the positive category. This assumes that the Bayesian score follows a normal distribution and is different from the prediction using a cutoff.
Enrichment: An estimate of enrichment, that is, the increased likelihood (versus random) of this sample being in the category.
Bayesian Score: The standard Laplacian-modified Bayesian score.
Mahalanobis Distance: The Mahalanobis distance (MD) is the distance to the center of the training data. The larger the MD, the less trustworthy the prediction.
Mahalanobis Distance p-value: The p-value gives the fraction of training data with an MD greater than or equal to the one for the given sample, assuming normally distributed data. The smaller the p-value, the less trustworthy the prediciton. For highly non-normal X properties (e.g., fingerprints), the MD p-value is wildly inaccurate.

Structural Similar Compounds

Name	o-Toluenesulfonamide, 4-amino-N-(2-hydroxyethyl)-	8-Methylamino-4-hydroxy-2-naphthalene sulfonic acid	C.I. Fluorescent Brightening Agent 24
Structure	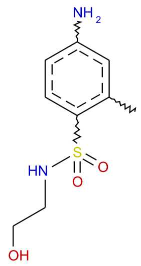	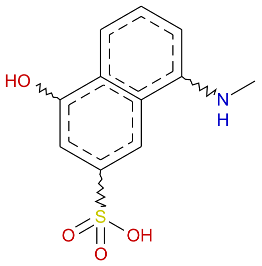	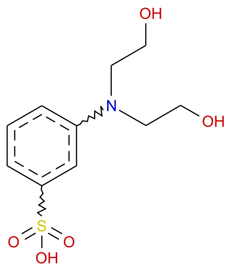
Actual Endpoint	Irritant	Non-Irritant	Irritant
Predicted Endpoint	Non-Irritant	Non-Irritant	Non-Irritant
Distance	0.705	0.707	0.723
Reference	85JCAE "Prehled Prumyslove Toxikologie; Organicke Latky," Marhold, J., Prague , Czechoslovakia, Avicenum, 1986 Volume(issue)/page/year: -,1076,1986	28ZPAK -,190,72	MVCRB3 MVC-Report. (Stockholm, Sweden) No.1-2, 1972-73. Discontinued. Volu me(issue)/page/year: 2,193,1973

Model Applicability

Unknown features are fingerprint features in the query molecule, but not found or appearing too infrequently in the training set.

1. All properties and OPS components are within expected ranges.

Feature Contribution

Top features for positive contribution				
**Fingerprint**	**Bit/Smiles**	**Feature Structure**	**Score**	**Irritant in training set**

FCFP_12	-124655670	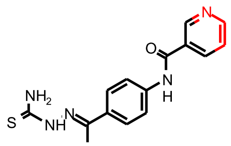 [*]:[cH]:[cH]:n:[*]	0.0821	13 out of 13
FCFP_12	-1695756380	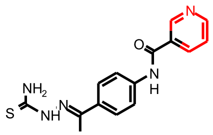 [*]1:[cH]:[cH]:[cH]:n :[cH]:1	0.0772	7 out of 7
FCFP_12	730557100	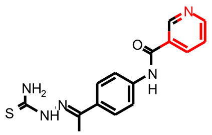 [*][c]1:[*]:n:[cH]:[c H]:[cH]:1	0.0756	6 out of 6

Top Features for negative contribution

Fingerprint	Bit/Smiles	Feature Structure	Score	Irritant in training set
FCFP_12	-1838187238	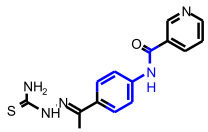 [*]C(=[*])N[c]1:[cH]: [cH]:[*]:[cH]:[cH]:1	-0.692	5 out of 12
FCFP_12	1294255210	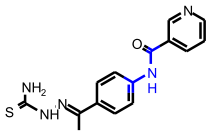 [*]C(=[*])N[c](:[*]): [*]	-0.486	12 out of 22

FCFP_12	-773983804	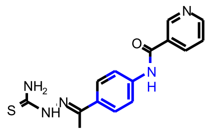 [*]N[c]1:[cH]:[*]:[c]([*]):[cH]:[cH]:1	-0.444	46 out of 79

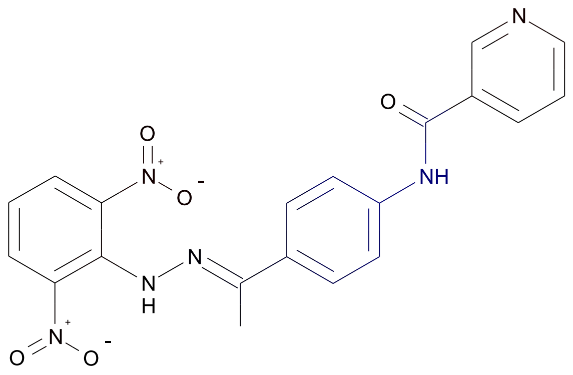

$C_{20}H_{16}N_6O_5$
Molecular Weight: 420.37824
ALogP: 2.852
Rotatable Bonds: 7
Acceptors: 8
Donors: 2

Model Prediction

Prediction: Non-Irritant
Probability: 0.932
Enrichment: 1.01
Bayesian Score: -2.3
Mahalanobis Distance: 6.31
Mahalanobis Distance p-value: 1

Prediction: Positive if the Bayesian score is above the estimated best cutoff value from minimizing the false positive and false negative rate.
Probability: The esimated probability that the sample is in the positive category. This assumes that the Bayesian score follows a normal distribution and is different from the prediction using a cutoff.
Enrichment: An estimate of enrichment, that is, the increased likelihood (versus random) of this sample being in the category.
Bayesian Score: The standard Laplacian-modified Bayesian score.
Mahalanobis Distance: The Mahalanobis distance (MD) is the distance to the center of the training data. The larger the MD, the less trustworthy the prediction.
Mahalanobis Distance p-value: The p-value gives the fraction of training data with an MD greater than or equal to the one for the given sample, assuming normally distributed data. The smaller the p-value, the less trustworthy the prediciton. For highly non-normal X properties (e.g., fingerprints), the MD p-value is wildly inaccurate.

Structural Similar Compounds

Name	2,2'-Stilbenedisulfonic acid, 4,4'-dinitro-	2-Anthracenesulfonic acid, 1-amino-9,10-dihydro-9,10-dioxo-4-(2,4,6 -trimethylanilino)-, monosodium salt	Benzenesulfonamide, 4-amino-N-(5,6-dimethoxy-4-pyrimidinyl)-
Structure	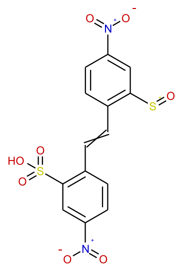	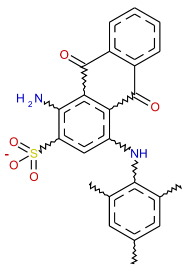	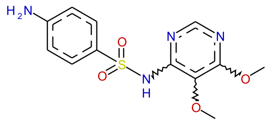
Actual Endpoint	Irritant	Irritant	Irritant
Predicted Endpoint	Irritant	Non-Irritant	Non-Irritant
Distance	0.696	0.711	0.820
Reference	85JCAE "Prehled Prumyslove Toxikologie; Organicke Latky," Marhold, J., Prague , Czechoslovakia, Avicenum, 1986 Volume(issue)/page/year: -,1062,1986	85JCAE "Prehled Prumyslove Toxikologie; Organicke Latky," Marhold, J., Prague , Czechoslovakia, Avicenum, 1986 Volume(issue)/page/year: -,1327,1986	FCTXAV 14,307,76

Model Applicability

Unknown features are fingerprint features in the query molecule, but not found or appearing too infrequently in the training set.

1. All properties and OPS components are within expected ranges.

Feature Contribution

Top features for positive contribution				
**Fingerprint**	**Bit/Smiles**	**Feature Structure**	**Score**	**Irritant in training set**

FCFP_12	5	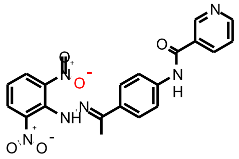<br>[*][O-]	0.0854	27 out of 27
FCFP_12	8	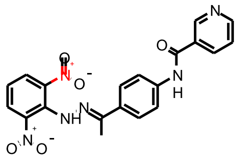<br>[*][N+](=[*])[*]	0.0843	20 out of 20
FCFP_12	-124655670	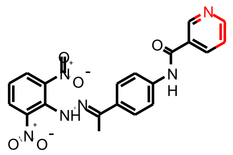<br>[*]:[cH]:[cH]:n:[*]	0.0821	13 out of 13
**Top Features for negative contribution**				
**Fingerprint**	**Bit/Smiles**	**Feature Structure**	**Score**	**Irritant in training set**
FCFP_12	-1838187238	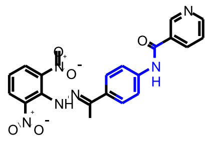<br>[*]C(=[*])N[c]1:[cH]:<br>[cH]:[*]:[cH]:[cH]:1	-0.692	5 out of 12
FCFP_12	1294255210	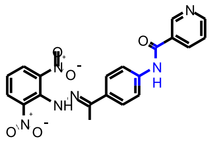<br>[*]C(=[*])N[c](:[*]):<br>[*]	-0.486	12 out of 22

FCFP_12	-773983804	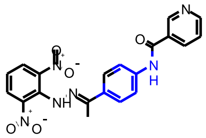 [*]N[c]1:[cH]:[*]:[c]([*]):[cH]:[cH]:1	-0.444	46 out of 79

Sorafenib

TOPKAT_Skin_Irritancy_None_vs_Irritant

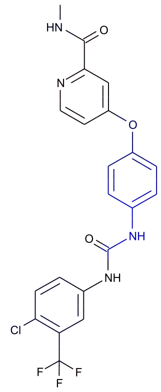

$C_{21}H_{16}ClF_3N_4O_3$
Molecular Weight: 464.82494
ALogP: 4.175
Rotatable Bonds: 6
Acceptors: 4
Donors: 3

Model Prediction

Prediction: Non-Irritant

Probability: 0.264

Enrichment: 0.287

Bayesian Score: -5.23

Mahalanobis Distance: 8.27

Mahalanobis Distance p-value: 0.791

Prediction: Positive if the Bayesian score is above the estimated best cutoff value from minimizing the false positive and false negative rate.
Probability: The esimated probability that the sample is in the positive category. This assumes that the Bayesian score follows a normal distribution and is different from the prediction using a cutoff.
Enrichment: An estimate of enrichment, that is, the increased likelihood (versus random) of this sample being in the category.
Bayesian Score: The standard Laplacian-modified Bayesian score.
Mahalanobis Distance: The Mahalanobis distance (MD) is the distance to the center of the training data. The larger the MD, the less trustworthy the prediction.
Mahalanobis Distance p-value: The p-value gives the fraction of training data with an MD greater than or equal to the one for the given sample, assuming normally distributed data. The smaller the p-value, the less trustworthy the prediciton. For highly non-normal X properties (e.g., fingerprints), the MD p-value is wildly inaccurate.

Structural Similar Compounds

Name	5-Norbornene-2,3-dicarboxylic acid, 1,4,5,6,7,7-hexachloro-	Benzenesulfonic acid, 2,2'-(4,4'-biphenylylenedivinylene)di-, disod ium salt	Sulfide, bis(4-t-butyl-m-cresyl)-
Structure	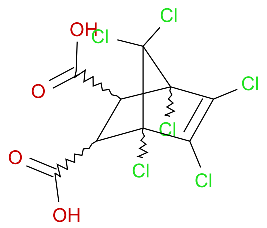	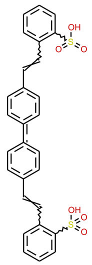	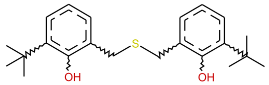
Actual Endpoint	Irritant	Irritant	Irritant
Predicted Endpoint	Irritant	Non-Irritant	Irritant
Distance	0.844	0.871	0.884
Reference	85JCAE "Prehled Prumyslove Toxikologie; Organicke Latky," Marhold, J., Prague , Czechoslovakia, Avicenum, 1986 Volume(issue)/page/year: -,581,1986	MVCRB3 MVC-Report. (Stockholm, Sweden) No.1-2, 1972-73. Discontinued. Volu me(issue)/page/year: 2,193,1973	AMIHBC AMA Archives of Industrial Hygiene and Occupational Medicine. (Chicago , IL) V.2-10, 1950-54. For publisher information, see AEHLAU. Volume(issue)/pag e/year: 5,311,1952

Model Applicability

Unknown features are fingerprint features in the query molecule, but not found or appearing too infrequently in the training set.

1. All properties and OPS components are within expected ranges.

Feature Contribution

Top features for positive contribution				
Fingerprint	Bit/Smiles	Feature Structure	Score	Irritant in training set

FCFP_12	-124655670	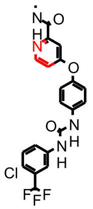 [*]:[cH]:[cH]:n:[*]	0.0821	13 out of 13
FCFP_12	-1539132615	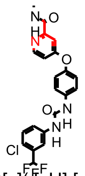 [*]C(=[*])[c](:[cH]:[*]):n:[*]	0.0795	9 out of 9
FCFP_12	-1695756380	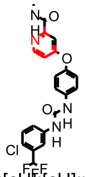 [*]1:[cH]:[cH]:[cH]:n:[cH]:1	0.0772	7 out of 7

Top Features for negative contribution

Fingerprint	Bit/Smiles	Feature Structure	Score	Irritant in training set
FCFP_12	-789307649	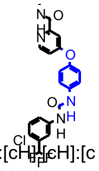 [*]O[c]1:[cH]:[cH]:[c](NC(=[*])[*]):[cH]:[cH]:1	-1.54	0 out of 4
FCFP_12	-1838187238	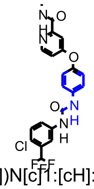 [*]C(=[*])N[c]1:[cH]:[cH]:[*]:[cH]:[cH]:1	-0.692	5 out of 12

FCFP_12	1294255210	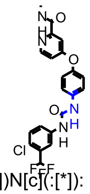<br>[*]C(=[*])N[c](:[*]):[*]	-0.486	12 out of 22

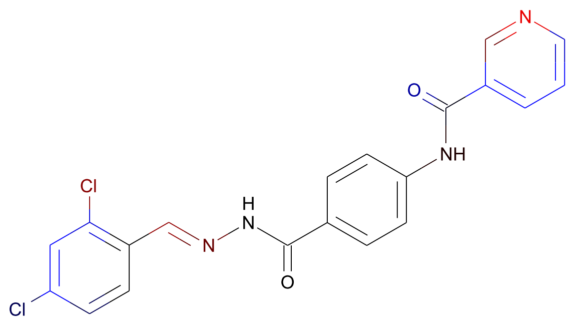

$C_{20}H_{14}Cl_2N_4O_2$
Molecular Weight: 413.25676
ALogP: 3.646
Rotatable Bonds: 5
Acceptors: 4
Donors: 2

Model Prediction

Prediction: 14.6
Unit: mg/kg_body_weight/day
Mahalanobis Distance: 12.9
Mahalanobis Distance p-value: 2.71e-007

Mahalanobis Distance: The Mahalanobis distance (MD) is a generalization of the Euclidean distance that accounts for correlations among the X properties. It is calculated as the distance to the center of the training data. The larger the MD, the less trustworthy the prediction.
Mahalanobis Distance p-value: The p-value gives the fraction of training data with an MD greater than or equal to the one for the given sample, assuming normally distributed data. The smaller the p-value, the less trustworthy the prediciton. For highly non-normal X properties (e.g., fingerprints), the MD p-value is wildly inaccurate.

Structural Similar Compounds

Name	455	422	Phenolphthalein
Structure	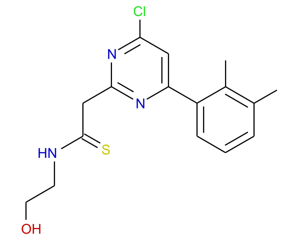	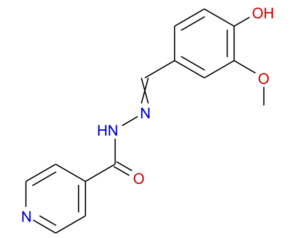	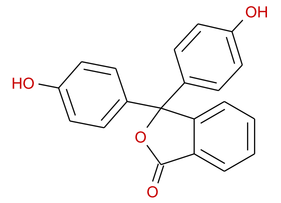
Actual Endpoint (-log C)	3.87681	3.99565	2.43468
Predicted Endpoint (-log C)	3.77582	3.22211	3.66084
Distance	0.690	0.696	0.697
Reference	CPDB	CPDB	CPDB

Model Applicability

Unknown features are fingerprint features in the query molecule, but not found or appearing too infrequently in the training set.

1. All properties and OPS components are within expected ranges.

Feature Contribution

Top features for positive contribution			
Fingerprint	Bit/Smiles	Feature Structure	Score
ECFP_6	655739385	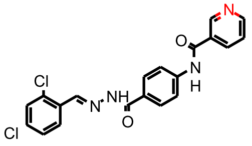 [*]:n:[*]	0.229

ECFP_6	-1925046727	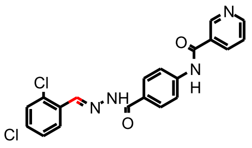 [*]C=[*]	0.145
ECFP_6	-817402818	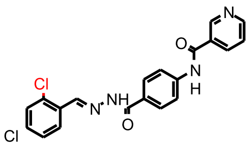 [*]Cl	0.129

Top Features for negative contribution

Fingerprint	Bit/Smiles	Feature Structure	Score
ECFP_6	1996767644	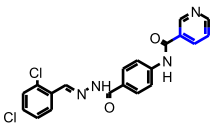 [*][c](:[*]):[cH]:[cH]:[*]	-0.251
ECFP_6	642810091	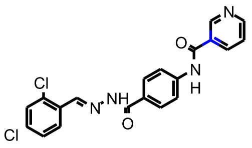 [*][c](:[*]):[*]	-0.247
ECFP_6	-182236392	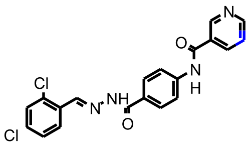 [*]:[cH]:[*]	-0.232

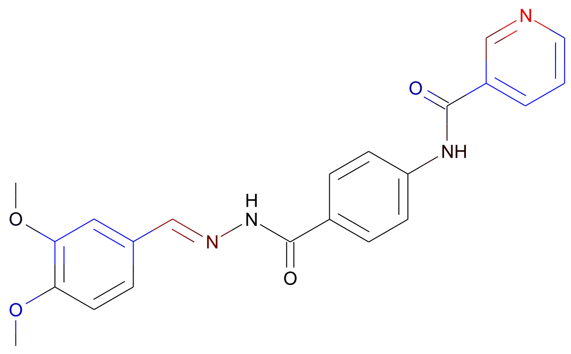

$C_{22}H_{20}N_4O_4$
Molecular Weight: 404.4186
ALogP: 2.284
Rotatable Bonds: 7
Acceptors: 6
Donors: 2

Model Prediction

Prediction: 41.1
Unit: mg/kg_body_weight/day
Mahalanobis Distance: 11.9
Mahalanobis Distance p-value: 2.76e-005

Mahalanobis Distance: The Mahalanobis distance (MD) is a generalization of the Euclidean distance that accounts for correlations among the X properties. It is calculated as the distance to the center of the training data. The larger the MD, the less trustworthy the prediction.
Mahalanobis Distance p-value: The p-value gives the fraction of training data with an MD greater than or equal to the one for the given sample, assuming normally distributed data. The smaller the p-value, the less trustworthy the prediciton. For highly non-normal X properties (e.g., fingerprints), the MD p-value is wildly inaccurate.

Structural Similar Compounds

Name	422	832	Compound LY171883
Structure	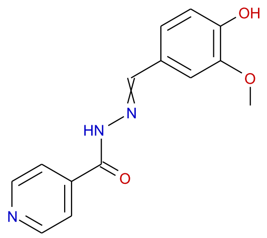	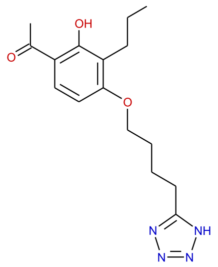	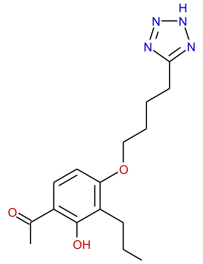
Actual Endpoint (-log C)	3.99565	3.45372	3.45372
Predicted Endpoint (-log C)	3.22211	2.80429	2.84749
Distance	0.596	0.697	0.702
Reference	CPDB	CPDB	CPDB

Model Applicability

Unknown features are fingerprint features in the query molecule, but not found or appearing too infrequently in the training set.

1. All properties and OPS components are within expected ranges.

Feature Contribution

Top features for positive contribution

Fingerprint	Bit/Smiles	Feature Structure	Score
ECFP_6	655739385	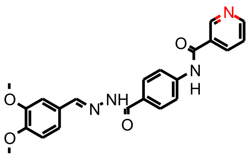 [*]:n:[*]	0.229

ECFP_6	-1925046727	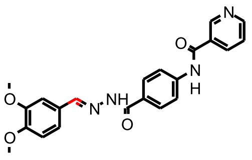 [*]C=[*]	0.145
ECFP_6	-175146122	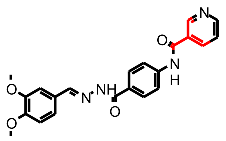 [*]C(=[*])[c](:[cH]:[*]):[cH]:[*]	0.107

Top Features for negative contribution

Fingerprint	Bit/Smiles	Feature Structure	Score
ECFP_6	1996767644	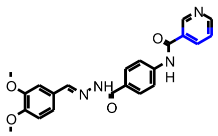 [*][c](:[*]):[cH]:[cH]:[*]	-0.251
ECFP_6	642810091	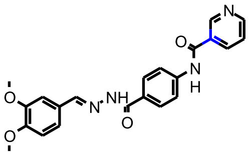 [*][c](:[*]):[*]	-0.247
ECFP_6	-182236392	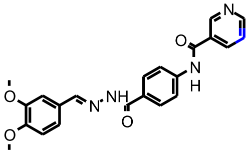 [*]:[cH]:[*]	-0.232

7c **TOPKAT_Carcinogenic_Potency_TD50_Mouse**

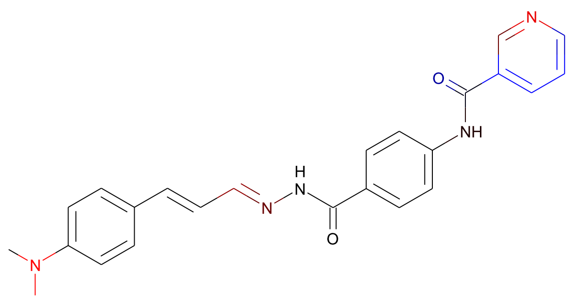

$C_{24}H_{23}N_5O_2$
Molecular Weight: 413.47172
ALogP: 2.839
Rotatable Bonds: 7
Acceptors: 5
Donors: 2

Model Prediction

Prediction: 3.01
Unit: mg/kg_body_weight/day
Mahalanobis Distance: 11.7
Mahalanobis Distance p-value: 7.15e-005

Mahalanobis Distance: The Mahalanobis distance (MD) is a generalization of the Euclidean distance that accounts for correlations among the X properties. It is calculated as the distance to the center of the training data. The larger the MD, the less trustworthy the prediction.
Mahalanobis Distance p-value: The p-value gives the fraction of training data with an MD greater than or equal to the one for the given sample, assuming normally distributed data. The smaller the p-value, the less trustworthy the prediciton. For highly non-normal X properties (e.g., fingerprints), the MD p-value is wildly inaccurate.

Structural Similar Compounds

Name	422	455	Compound LY171883
Structure	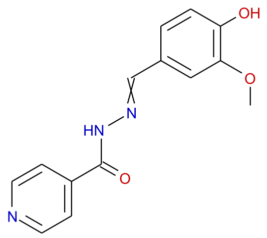	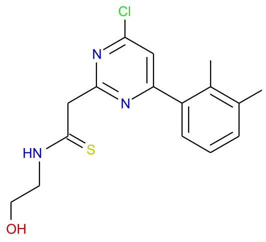	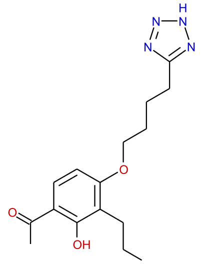
Actual Endpoint (-log C)	3.99565	3.87681	3.45372
Predicted Endpoint (-log C)	3.22211	3.77582	2.84749
Distance	0.704	0.720	0.733
Reference	CPDB	CPDB	CPDB

Model Applicability

Unknown features are fingerprint features in the query molecule, but not found or appearing too infrequently in the training set.

1. All properties and OPS components are within expected ranges.
2. Unknown ECFP_2 feature: -984423246: [*]\N=C\C=[*]

Feature Contribution

Top features for positive contribution

Fingerprint	Bit/Smiles	Feature Structure	Score
ECFP_6	-1072294614	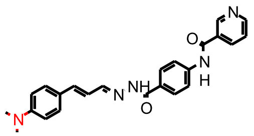 [*]N([*])[*]	0.428

ECFP_6	655739385	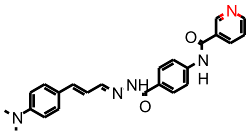 [*]:n:[*]	0.229
ECFP_6	865379614	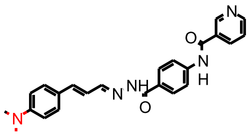 [*]N([*])C	0.219
**Top Features for negative contribution**			
**Fingerprint**	**Bit/Smiles**	**Feature Structure**	**Score**
ECFP_6	1996767644	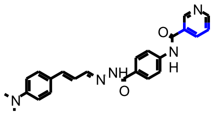 [*][c](:[*]):[cH]:[cH]:[*]	-0.251
ECFP_6	642810091	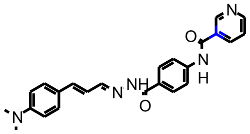 [*][c](:[*]):[*]	-0.247
ECFP_6	-182236392	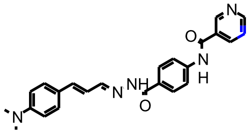 [*]:[cH]:[*]	-0.232

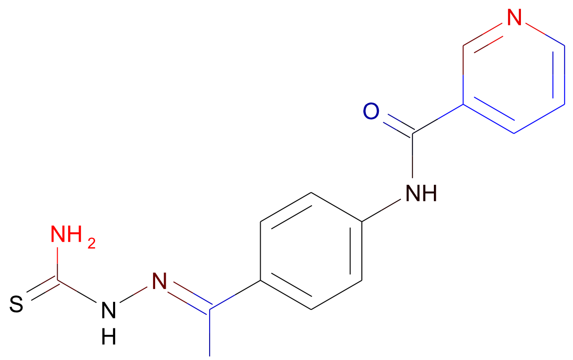

$C_{15}H_{15}N_5OS$

Molecular Weight: 313.3775

ALogP: 1.414

Rotatable Bonds: 5

Acceptors: 4

Donors: 3

Model Prediction

Prediction: 38.5

Unit: mg/kg_body_weight/day

Mahalanobis Distance: 12.4

Mahalanobis Distance p-value: 3.58e-006

Mahalanobis Distance: The Mahalanobis distance (MD) is a generalization of the Euclidean distance that accounts for correlations among the X properties. It is calculated as the distance to the center of the training data. The larger the MD, the less trustworthy the prediction.
Mahalanobis Distance p-value: The p-value gives the fraction of training data with an MD greater than or equal to the one for the given sample, assuming normally distributed data. The smaller the p-value, the less trustworthy the prediciton. For highly non-normal X properties (e.g., fingerprints), the MD p-value is wildly inaccurate.

Structural Similar Compounds

Name	Furosemide	Formic acid 2-[4-(5-nitro-2-furyl)-2-thiazolyl]hydrazide s	Sulfamethazine
Structure	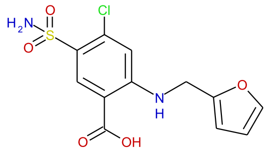	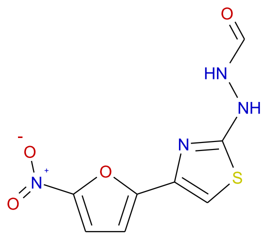	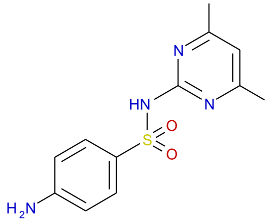
Actual Endpoint (-log C)	2.65498	4.37179	2.26558
Predicted Endpoint (-log C)	3.60472	4.32851	3.67808
Distance	0.539	0.642	0.653
Reference	CPDB	CPDB	CPDB

Model Applicability

Unknown features are fingerprint features in the query molecule, but not found or appearing too infrequently in the training set.

1. All properties and OPS components are within expected ranges.
2. Unknown ECFP_2 feature: 128986386: [*]\N=C(/C)\[c](:[*]):[*]
3. Unknown ECFP_2 feature: 560380707: [*]NN=C([*])[*]
4. Unknown ECFP_2 feature: -571028867: [*]NC(=S)N

Feature Contribution

Top features for positive contribution

Fingerprint	Bit/Smiles	Feature Structure	Score
ECFP_6	655739385	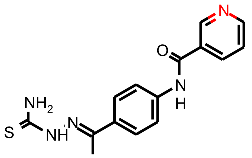 [*]:n:[*]	0.229

ECFP_6	1572579716	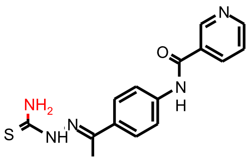 [*]N	0.225
ECFP_6	-175146122	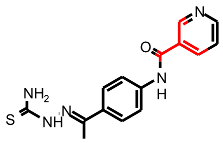 [*]C(=[*])[c](:[cH]:[*]):[cH]:[*]	0.107
**Top Features for negative contribution**			
**Fingerprint**	**Bit/Smiles**	**Feature Structure**	**Score**
ECFP_6	1996767644	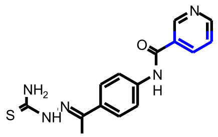 [*][c](:[*]):[cH]:[cH]:[*]	-0.251
ECFP_6	642810091	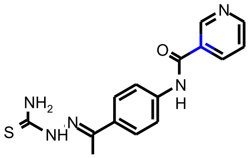 [*][c](:[*]):[*]	-0.247
ECFP_6	-182236392	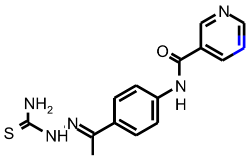 [*]:[cH]:[*]	-0.232

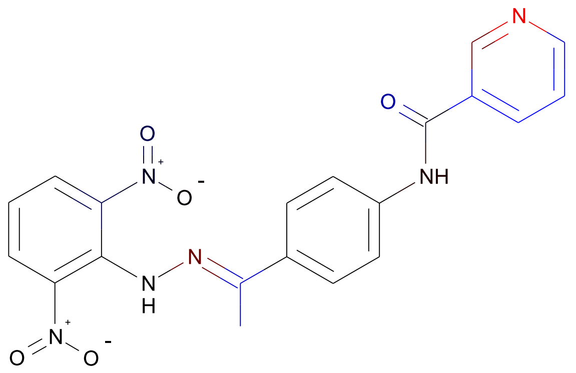

$C_{20}H_{16}N_6O_5$

Molecular Weight: 420.37824

ALogP: 2.852

Rotatable Bonds: 7

Acceptors: 8

Donors: 2

Model Prediction

Prediction: 33.1

Unit: mg/kg_body_weight/day

Mahalanobis Distance: 12.1

Mahalanobis Distance p-value: 1.46e-005

Mahalanobis Distance: The Mahalanobis distance (MD) is a generalization of the Euclidean distance that accounts for correlations among the X properties. It is calculated as the distance to the center of the training data. The larger the MD, the less trustworthy the prediction.
Mahalanobis Distance p-value: The p-value gives the fraction of training data with an MD greater than or equal to the one for the given sample, assuming normally distributed data. The smaller the p-value, the less trustworthy the prediciton. For highly non-normal X properties (e.g., fingerprints), the MD p-value is wildly inaccurate.

Structural Similar Compounds

Name	Salicylazosulfapyridine	470	420
Structure	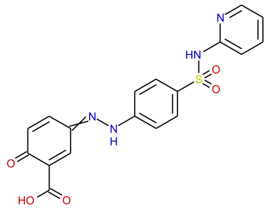	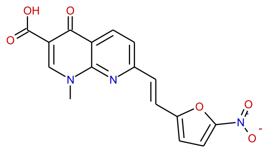	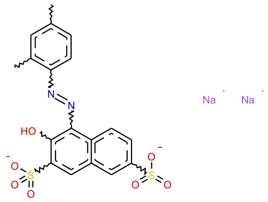
Actual Endpoint (-log C)	2.5034	4.62839	2.78302
Predicted Endpoint (-log C)	3.54214	3.93264	3.31546
Distance	0.663	0.745	0.765
Reference	CPDB	CPDB	CPDB

Model Applicability

Unknown features are fingerprint features in the query molecule, but not found or appearing too infrequently in the training set.

1. All properties and OPS components are within expected ranges.
2. Unknown ECFP_2 feature: 128986386: [*]\N=C(/C)\[c](:[*]):[*]
3. Unknown ECFP_2 feature: 560380707: [*]NN=C([*])[*]
4. Unknown ECFP_2 feature: -1660205591: [*]N[c](:[c]([*]):[*]):[c]([*]):[*]

Feature Contribution

Top features for positive contribution			
Fingerprint	Bit/Smiles	Feature Structure	Score
ECFP_6	655739385	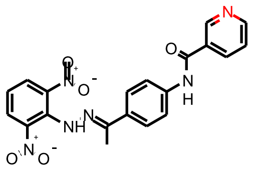 [*]:n:[*]	0.229

ECFP_6	-175146122	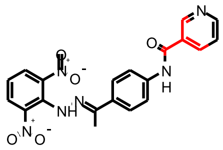<br>[*]C(=[*])[c](:[cH]:[*]):[cH]:[*]	0.107
ECFP_6	-1087070950	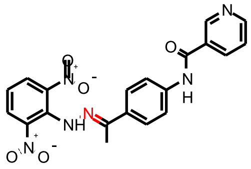<br>[*]N=[*]	0.104
**Top Features for negative contribution**			
**Fingerprint**	**Bit/Smiles**	**Feature Structure**	**Score**
ECFP_6	1996767644	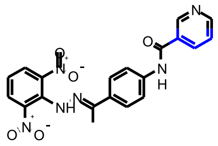<br>[*][c](:[*]):[cH]:[cH]:[*]	-0.251
ECFP_6	642810091	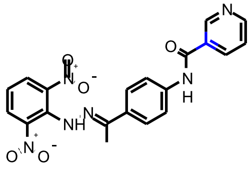<br>[*][c](:[*]):[*]	-0.247
ECFP_6	-182236392	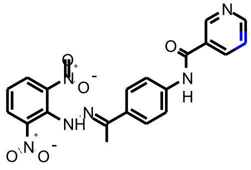<br>[*]:[cH]:[*]	-0.232

Sorafenib

TOPKAT_Carcinogenic_Potency_TD50_Mouse

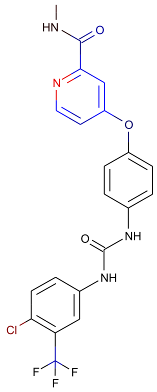

$C_{21}H_{16}ClF_3N_4O_3$

Molecular Weight: 464.82494

ALogP: 4.175

Rotatable Bonds: 6

Acceptors: 4

Donors: 3

Model Prediction

Prediction: 19.2

Unit: mg/kg_body_weight/day

Mahalanobis Distance: 12.4

Mahalanobis Distance p-value: 2.94e-006

Mahalanobis Distance: The Mahalanobis distance (MD) is a generalization of the Euclidean distance that accounts for correlations among the X properties. It is calculated as the distance to the center of the training data. The larger the MD, the less trustworthy the prediction.
Mahalanobis Distance p-value: The p-value gives the fraction of training data with an MD greater than or equal to the one for the given sample, assuming normally distributed data. The smaller the p-value, the less trustworthy the prediciton. For highly non-normal X properties (e.g., fingerprints), the MD p-value is wildly inaccurate.

Structural Similar Compounds

Name	Ochratoxin A	542	4-Chloro-6-(2,3-xylidino)-2-pyri-mi-dinylthio(N-b-hydroxy-ethyl) acetamide
Structure	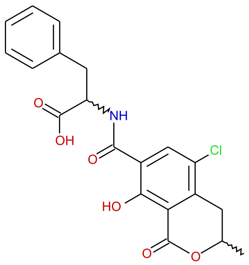	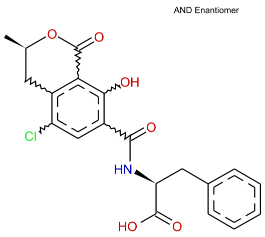	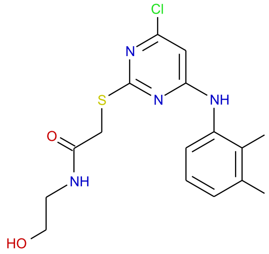
Actual Endpoint (-log C)	4.79932	4.79932	3.91517
Predicted Endpoint (-log C)	3.6353	3.6353	3.92186
Distance	0.718	0.718	0.738
Reference	CPDB	CPDB	CPDB

Model Applicability

Unknown features are fingerprint features in the query molecule, but not found or appearing too infrequently in the training set.

1. All properties and OPS components are within expected ranges.
2. Unknown ECFP_2 feature: 1413420509: [*]C(=[*])[c](:n:[*]):c:[*]
3. Unknown ECFP_2 feature: 1338334141: [*]C(=[*])NC

Feature Contribution

Top features for positive contribution			
Fingerprint	Bit/Smiles	Feature Structure	Score
ECFP_6	655739385	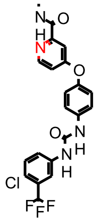 [*]:n:[*]	0.229

ECFP_6	-817402818	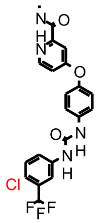 [*]Cl	0.129
ECFP_6	-176455838	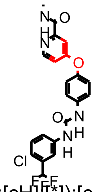 [*]O[c](:[cH]:[*]):[cH]:[*]	0.0818
**Top Features for negative contribution**			
**Fingerprint**	**Bit/Smiles**	**Feature Structure**	**Score**
ECFP_6	1996767644	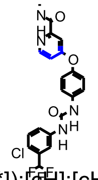 [*][c](:[*]):[cH]:[cH]:[*]	-0.251
ECFP_6	642810091	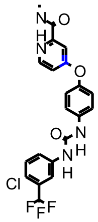 [*][c](:[*]):[*]	-0.247
ECFP_6	-182236392	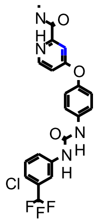 [*]:[cH]:[*]	-0.232

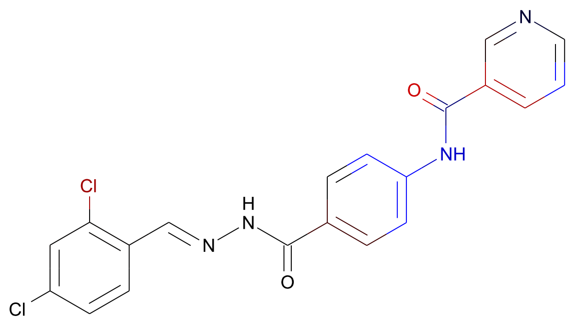

$C_{20}H_{14}Cl_2N_4O_2$
Molecular Weight: 413.25676
ALogP: 3.646
Rotatable Bonds: 5
Acceptors: 4
Donors: 2

Model Prediction

Prediction: 11.3
Unit: mg/kg_body_weight/day
Mahalanobis Distance: 11.9
Mahalanobis Distance p-value: 0.000962

Mahalanobis Distance: The Mahalanobis distance (MD) is a generalization of the Euclidean distance that accounts for correlations among the X properties. It is calculated as the distance to the center of the training data. The larger the MD, the less trustworthy the prediction.
Mahalanobis Distance p-value: The p-value gives the fraction of training data with an MD greater than or equal to the one for the given sample, assuming normally distributed data. The smaller the p-value, the less trustworthy the prediciton. For highly non-normal X properties (e.g., fingerprints), the MD p-value is wildly inaccurate.

Structural Similar Compounds

Name	Indomethacin	3-(Cyclopentyloxy)-N-(3,5-di-chloro-4-pyridyl)-4-methoxy-benzamide	Omeprazole
Structure	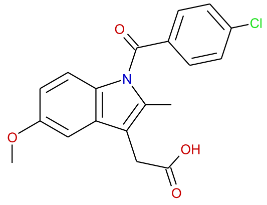	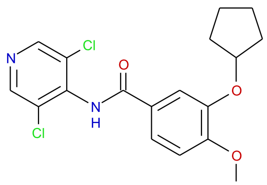	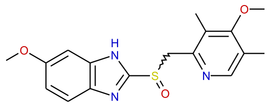
Actual Endpoint (-log C)	5.49293	5.39369	3.4628
Predicted Endpoint (-log C)	4.9569	4.27874	4.7324
Distance	0.590	0.594	0.622
Reference	CPDB	CPDB	CPDB

Model Applicability

Unknown features are fingerprint features in the query molecule, but not found or appearing too infrequently in the training set.

1. OPS PC6 out of range. Value: 6.7455. Training min, max, SD, explained variance: -5.5832, 6.4847, 1.973, 0.0374.

Feature Contribution

Top features for positive contribution

Fingerprint	Bit/Smiles	Feature Structure	Score
FCFP_6	1	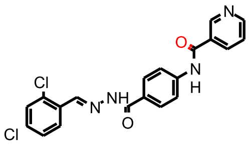 [*]=O	0.234

FCFP_6	32	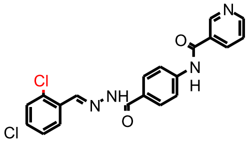 [*]Cl	0.154
FCFP_6	730557100	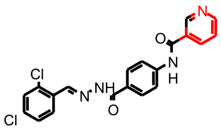 [*][c]1:[*]:n:[cH]:[cH]:[cH]:1	0.141
**Top Features for negative contribution**			
**Fingerprint**	**Bit/Smiles**	**Feature Structure**	**Score**
FCFP_6	16	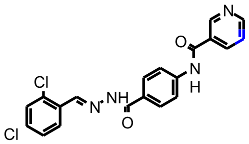 [*]:[cH]:[*]	-0.354
FCFP_6	590925877	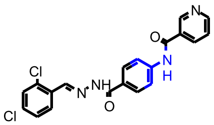 [*]N[c](:[cH]:[*]):[cH]:[*]	-0.323
FCFP_6	17	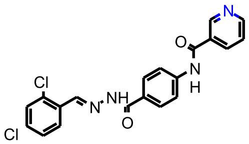 [*]:n:[*]	-0.149

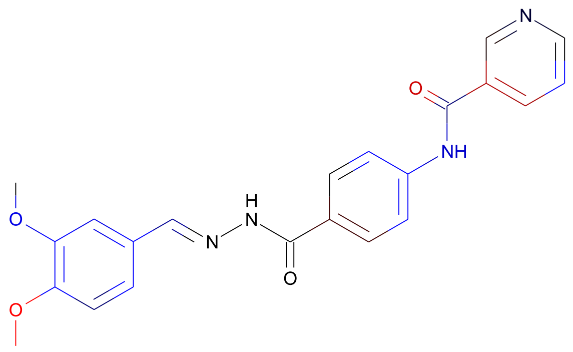

$C_{22}H_{20}N_4O_4$

Molecular Weight: 404.4186

ALogP: 2.284

Rotatable Bonds: 7

Acceptors: 6

Donors: 2

Model Prediction

Prediction: 10.9

Unit: mg/kg_body_weight/day

Mahalanobis Distance: 13.7

Mahalanobis Distance p-value: 1.54e-007

Mahalanobis Distance: The Mahalanobis distance (MD) is a generalization of the Euclidean distance that accounts for correlations among the X properties. It is calculated as the distance to the center of the training data. The larger the MD, the less trustworthy the prediction.
Mahalanobis Distance p-value: The p-value gives the fraction of training data with an MD greater than or equal to the one for the given sample, assuming normally distributed data. The smaller the p-value, the less trustworthy the prediciton. For highly non-normal X properties (e.g., fingerprints), the MD p-value is wildly inaccurate.

Structural Similar Compounds

Name	Loxtidine	Omeprazole	C.I. direct brown 95
Structure	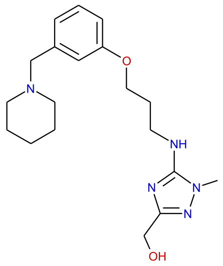	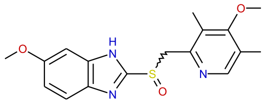	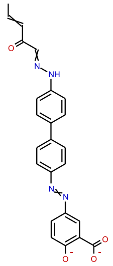
Actual Endpoint (-log C)	2.87532	3.4628	5.31387
Predicted Endpoint (-log C)	3.63996	4.7324	4.30266
Distance	0.632	0.639	0.658
Reference	CPDB	CPDB	CPDB

Model Applicability

Unknown features are fingerprint features in the query molecule, but not found or appearing too infrequently in the training set.

1. All properties and OPS components are within expected ranges.

Feature Contribution

Top features for positive contribution			
**Fingerprint**	**Bit/Smiles**	**Feature Structure**	**Score**
FCFP_6	136627117	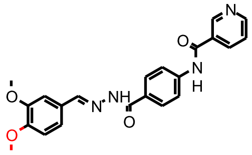 [*]OC	0.69

FCFP_6	1	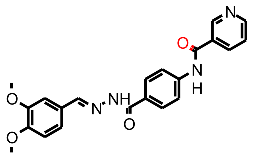 [*]=O	0.234
FCFP_6	730557100	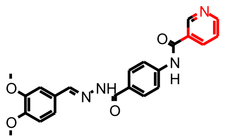 [*][c]1:[*]:n:[cH]:[cH]:[cH]:1	0.141
**Top Features for negative contribution**			
**Fingerprint**	**Bit/Smiles**	**Feature Structure**	**Score**
FCFP_6	16	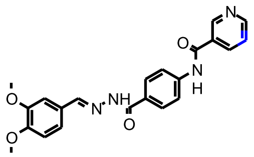 [*]:[cH]:[*]	-0.354
FCFP_6	590925877	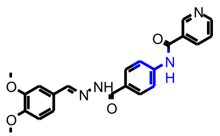 [*]N[c](:[cH]:[*]):[cH]:[*]	-0.323
FCFP_6	1674451008	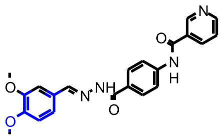 [*]O[c]1:[cH]:[cH]:[c]([*]):[*]:[c]:1[*]	-0.233

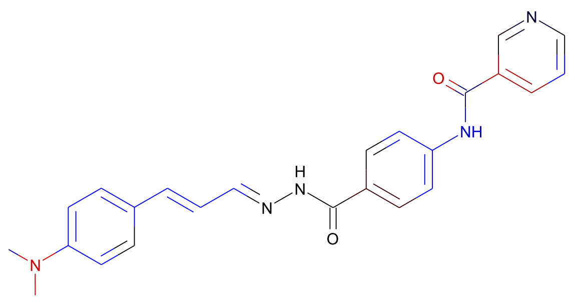

$C_{24}H_{23}N_5O_2$
Molecular Weight: 413.47172
ALogP: 2.839
Rotatable Bonds: 7
Acceptors: 5
Donors: 2

Model Prediction

Prediction: 29.1
Unit: mg/kg_body_weight/day
Mahalanobis Distance: 13.4
Mahalanobis Distance p-value: 6.93e-007

Mahalanobis Distance: The Mahalanobis distance (MD) is a generalization of the Euclidean distance that accounts for correlations among the X properties. It is calculated as the distance to the center of the training data. The larger the MD, the less trustworthy the prediction.
Mahalanobis Distance p-value: The p-value gives the fraction of training data with an MD greater than or equal to the one for the given sample, assuming normally distributed data. The smaller the p-value, the less trustworthy the prediciton. For highly non-normal X properties (e.g., fingerprints), the MD p-value is wildly inaccurate.

Structural Similar Compounds

Name	Omeprazole	Loxtidine	3-(Cyclopentyloxy)-N-(3,5-di-chloro-4-pyridyl)-4-methoxy-benzamide
Structure	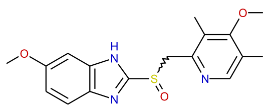	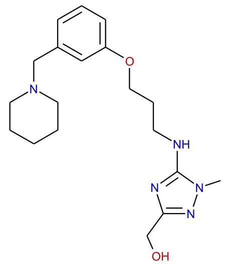	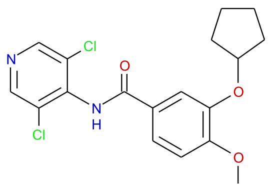
Actual Endpoint (-log C)	3.4628	2.87532	5.39369
Predicted Endpoint (-log C)	4.7324	3.63996	4.27874
Distance	0.641	0.644	0.659
Reference	CPDB	CPDB	CPDB

Model Applicability

Unknown features are fingerprint features in the query molecule, but not found or appearing too infrequently in the training set.

1. All properties and OPS components are within expected ranges.

Feature Contribution

Top features for positive contribution			
**Fingerprint**	**Bit/Smiles**	**Feature Structure**	**Score**
FCFP_6	136627117	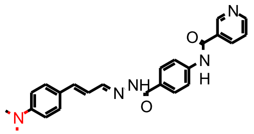 [*]OC	0.69

FCFP_6	1	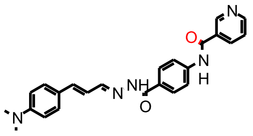 [*]=O	0.234
FCFP_6	730557100	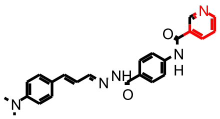 [*][c]1:[*]:n:[cH]:[cH]:[cH]:1	0.141
**Top Features for negative contribution**			
**Fingerprint**	**Bit/Smiles**	**Feature Structure**	**Score**
FCFP_6	451847724	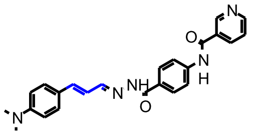 [*]\C=C\C=[*]	-0.436
FCFP_6	16	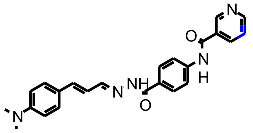 [*]:[cH]:[*]	-0.354
FCFP_6	590925877	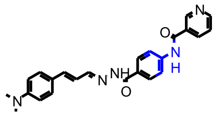 [*]N[c](:[cH]:[*]):[cH]:[*]	-0.323

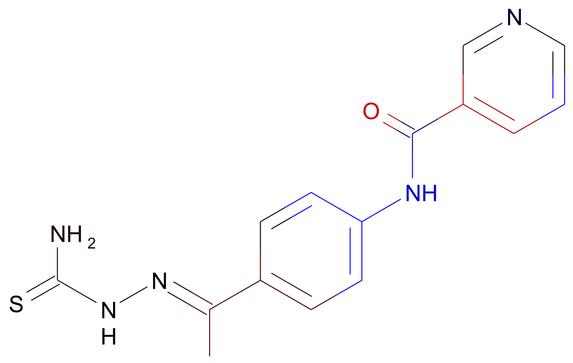

$C_{15}H_{15}N_5OS$
Molecular Weight: 313.3775
ALogP: 1.414
Rotatable Bonds: 5
Acceptors: 4
Donors: 3

Structural Similar Compounds

Name	4,4´-Sulfonylbisacetanilide	1,2-Dihydro-2-(5-nitro-2-thi-enyl) quinazolin-4(3H)-one	Formic acid 2-[4-(5-nitro-2-furyl)-2-thiazolyl]hydrazide s
Structure	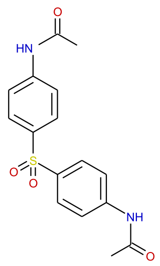	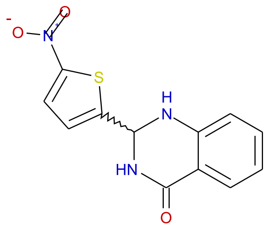	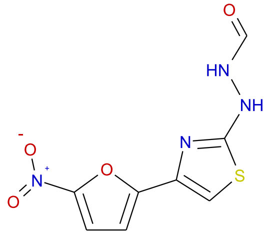
Actual Endpoint (-log C)	3.77655	5.25509	4.70106
Predicted Endpoint (-log C)	3.55337	3.89291	4.66041
Distance	0.541	0.639	0.641
Reference	CPDB	CPDB	CPDB

Model Prediction

Prediction: 71.8
Unit: mg/kg_body_weight/day
Mahalanobis Distance: 11.6
Mahalanobis Distance p-value: 0.00256

Mahalanobis Distance: The Mahalanobis distance (MD) is a generalization of the Euclidean distance that accounts for correlations among the X properties. It is calculated as the distance to the center of the training data. The larger the MD, the less trustworthy the prediction.
Mahalanobis Distance p-value: The p-value gives the fraction of training data with an MD greater than or equal to the one for the given sample, assuming normally distributed data. The smaller the p-value, the less trustworthy the prediciton. For highly non-normal X properties (e.g., fingerprints), the MD p-value is wildly inaccurate.

Model Applicability

Unknown features are fingerprint features in the query molecule, but not found or appearing too infrequently in the training set.

1. All properties and OPS components are within expected ranges.

Feature Contribution

Top features for positive contribution

Fingerprint	Bit/Smiles	Feature Structure	Score
FCFP_6	1	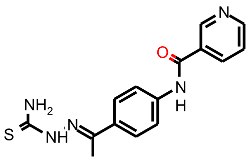 [*]=O	0.234

FCFP_6	730557100	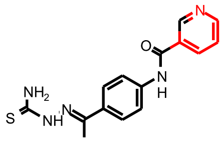 [*][c]1:[*]:n:[cH]:[cH]:[cH]:1	0.141
FCFP_6	203677720	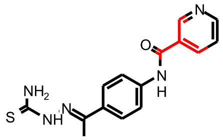 [*]C(=[*])[c](:[cH]:[*]):[cH]:[*]	0.137
**Top Features for negative contribution**			
**Fingerprint**	**Bit/Smiles**	**Feature Structure**	**Score**
FCFP_6	16	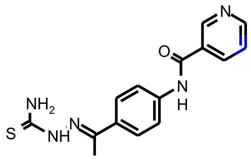 [*]:[cH]:[*]	-0.354
FCFP_6	590925877	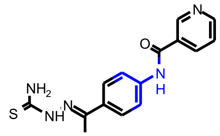 [*]N[c](:[cH]:[*]):[cH]:[*]	-0.323
FCFP_6	17	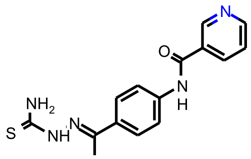 [*]:n:[*]	-0.149

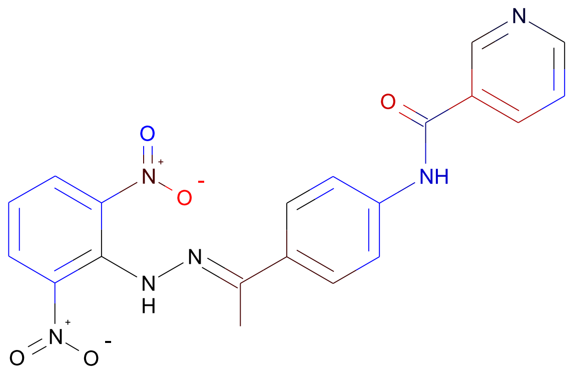

$C_{20}H_{16}N_6O_5$
Molecular Weight: 420.37824
ALogP: 2.852
Rotatable Bonds: 7
Acceptors: 8
Donors: 2

Structural Similar Compounds

Name	623	Salicylazosulfapyridine	4-Bis(2-hydroxyethyl)amino-2-(5-nitro-2-thienyl)quinazoline
Structure	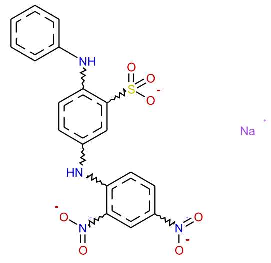	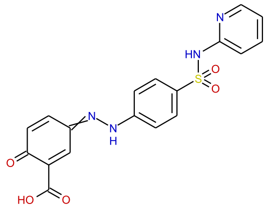	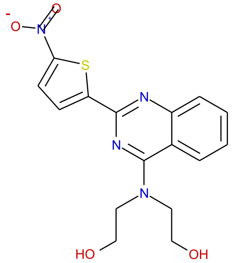
Actual Endpoint (-log C)	2.39985	2.39891	5.05984
Predicted Endpoint (-log C)	3.4177	3.17598	4.23808
Distance	0.531	0.617	0.625
Reference	CPDB	CPDB	CPDB

Model Prediction

Prediction: 23
Unit: mg/kg_body_weight/day
Mahalanobis Distance: 12.9
Mahalanobis Distance p-value: 6.99e-006

Mahalanobis Distance: The Mahalanobis distance (MD) is a generalization of the Euclidean distance that accounts for correlations among the X properties. It is calculated as the distance to the center of the training data. The larger the MD, the less trustworthy the prediction.
Mahalanobis Distance p-value: The p-value gives the fraction of training data with an MD greater than or equal to the one for the given sample, assuming normally distributed data. The smaller the p-value, the less trustworthy the prediciton. For highly non-normal X properties (e.g., fingerprints), the MD p-value is wildly inaccurate.

Model Applicability

Unknown features are fingerprint features in the query molecule, but not found or appearing too infrequently in the training set.

1. OPS PC24 out of range. Value: -3.3209. Training min, max, SD, explained variance: -3.0088, 7.4204, 1.076, 0.0111.

Feature Contribution

Top features for positive contribution

Fingerprint	Bit/Smiles	Feature Structure	Score
FCFP_6	5	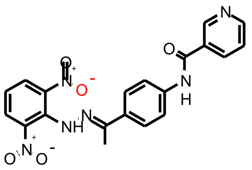 [*][O-]	0.431

FCFP_6	8	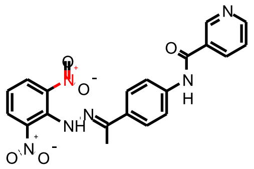 [*][N+](=[*])[*]	0.336
FCFP_6	1	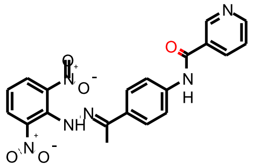 [*]=O	0.234
**Top Features for negative contribution**			
**Fingerprint**	**Bit/Smiles**	**Feature Structure**	**Score**
FCFP_6	991735244	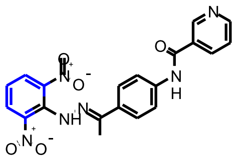 [*][c]1:[*]:[c]([*]):[cH]:[cH]:[cH]:1	-0.422
FCFP_6	16	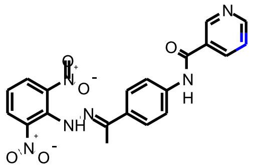 [*]:[cH]:[*]	-0.354
FCFP_6	590925877	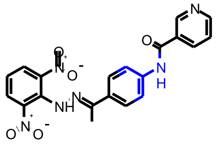 [*]N[c](:[cH]:[*]):[cH]:[*]	-0.323

Sorafenib

TOPKAT_Carcinogenic_Potency_TD50_Rat

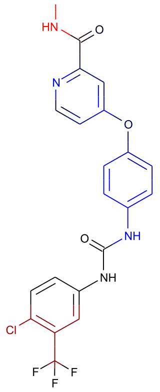

$C_{21}H_{16}ClF_3N_4O_3$

Molecular Weight: 464.82494

ALogP: 4.175

Rotatable Bonds: 6

Acceptors: 4

Donors: 3

Model Prediction

Prediction: 14.2

Unit: mg/kg_body_weight/day

Mahalanobis Distance: 20.4

Mahalanobis Distance p-value: 9.56e-031

Mahalanobis Distance: The Mahalanobis distance (MD) is a generalization of the Euclidean distance that accounts for correlations among the X properties. It is calculated as the distance to the center of the training data. The larger the MD, the less trustworthy the prediction.
Mahalanobis Distance p-value: The p-value gives the fraction of training data with an MD greater than or equal to the one for the given sample, assuming normally distributed data. The smaller the p-value, the less trustworthy the prediciton. For highly non-normal X properties (e.g., fingerprints), the MD p-value is wildly inaccurate.

Structural Similar Compounds

Name	Fluvastatin	913	Ochratoxin A
Structure	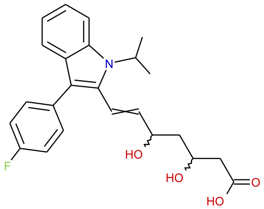	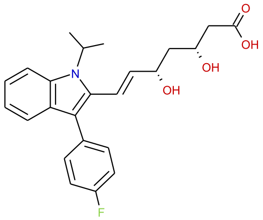	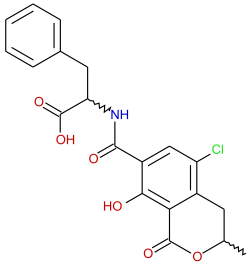
Actual Endpoint (-log C)	3.51742	3.51742	6.47264
Predicted Endpoint (-log C)	5.41573	5.41573	5.06501
Distance	0.597	0.597	0.666
Reference	CPDB	CPDB	CPDB

Model Applicability

Unknown features are fingerprint features in the query molecule, but not found or appearing too infrequently in the training set.

1. All properties and OPS components are within expected ranges.
2. Unknown FCFP_2 feature: -1029533685: [*]:[c](:[*])C(F)(F)F

Feature Contribution

Top features for positive contribution

Fingerprint	Bit/Smiles	Feature Structure	Score
FCFP_6	1	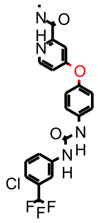 [*]=O	0.234

FCFP_6	-885550502	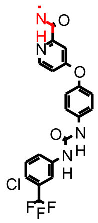 [*]C(=[*])NC	0.229
FCFP_6	32	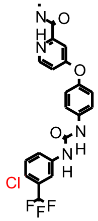 [*]Cl	0.154

Top Features for negative contribution

Fingerprint	Bit/Smiles	Feature Structure	Score
FCFP_6	16	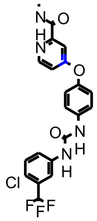 [*]:[cH]:[*]	-0.354
FCFP_6	590925877	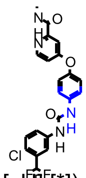 [*]N[c](:[cH]:[*]):[cH]:[*]	-0.323
FCFP_6	1674451008	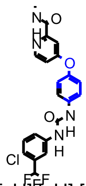 [*]O[c]1:[cH]:[cH]:[c]([*]):[*]:[c]:1[*]	-0.233

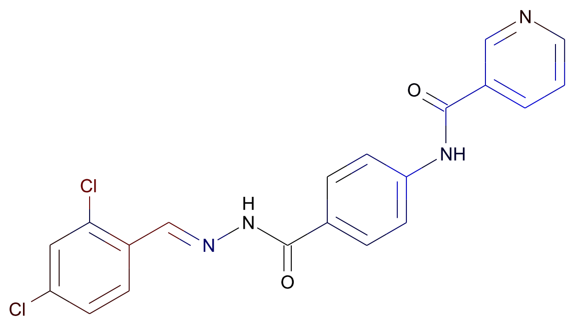

$C_{20}H_{14}Cl_2N_4O_2$
Molecular Weight: 413.25676
ALogP: 3.646
Rotatable Bonds: 5
Acceptors: 4
Donors: 2

Model Prediction

Prediction: 0.12
Unit: g/kg_body_weight
Mahalanobis Distance: 28
Mahalanobis Distance p-value: 4.66e-021

Mahalanobis Distance: The Mahalanobis distance (MD) is a generalization of the Euclidean distance that accounts for correlations among the X properties. It is calculated as the distance to the center of the training data. The larger the MD, the less trustworthy the prediction.
Mahalanobis Distance p-value: The p-value gives the fraction of training data with an MD greater than or equal to the one for the given sample, assuming normally distributed data. The smaller the p-value, the less trustworthy the prediciton. For highly non-normal X properties (e.g., fingerprints), the MD p-value is wildly inaccurate.

Structural Similar Compounds

Name	D & C RED 9	SODIUM ACIFLUORFEN	ISOXABEN
Structure	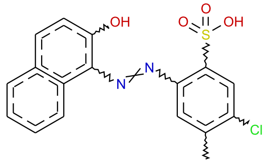	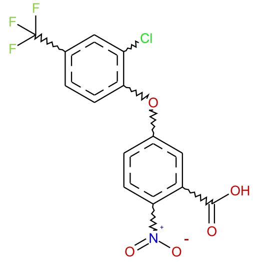	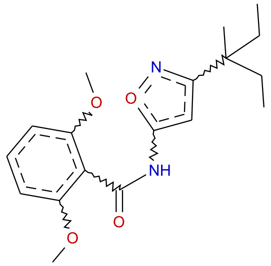
Actual Endpoint (-log C)	3.87715	4.16036	3.81665
Predicted Endpoint (-log C)	3.6546	4.65915	4.42315
Distance	0.666	0.667	0.668
Reference	NTP REPORT # 225	EPA COVER SHEET 0192;891101;(1)	EPA COVER SHEET 0339;881201;(1)

Model Applicability

Unknown features are fingerprint features in the query molecule, but not found or appearing too infrequently in the training set.

1. All properties and OPS components are within expected ranges.
2. Unknown ECFP_6 feature: 1997021792: [*]:[cH]:[cH]:[cH]:[*]
3. Unknown ECFP_6 feature: 1996163143: [*]:[cH]:[cH]:n:[*]
4. Unknown ECFP_6 feature: -677055651: [*]:[cH]:n:[cH]:[*]
5. Unknown ECFP_6 feature: -709633021: [*][c](:[*]):[cH]:n:[*]
6. Unknown ECFP_6 feature: -175146122: [*]C(=[*])[c](:[cH]:[*]):[cH]:[*]
7. Unknown ECFP_6 feature: 1430169877: [*]NC(=O)[c](:[*]):[*]
8. Unknown ECFP_6 feature: -177077903: [*]N[c](:[cH]:[*]):[cH]:[*]
9. Unknown ECFP_6 feature: 544048674: [*]C(=[*])NN=[*]
10. Unknown ECFP_6 feature: 1814278164: [*]N\N=C\[*]
11. Unknown ECFP_6 feature: -1832102709: [*]\N=C\[c](:[*]):[*]
12. Unknown ECFP_6 feature: 1335702447: [*][c](:[*]):[c](C=[*]):[cH]:[*]
13. Unknown ECFP_6 feature: -176494269: [*]:[cH]:[c](Cl):[cH]:[*]
14. Unknown ECFP_6 feature: 99947387: [*]:[c](:[*])Cl

Feature Contribution

Top features for positive contribution			
**Fingerprint**	**Bit/Smiles**	**Feature Structure**	**Score**

FCFP_6	32	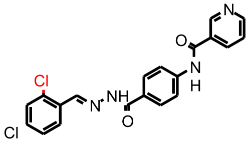<br>[*]Cl	0.101
FCFP_6	3	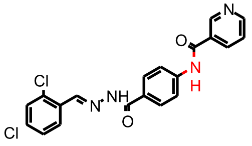<br>[*]N[*]	0.0924
ECFP_6	-1925046727	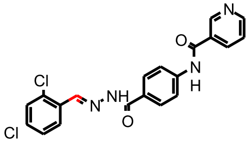<br>[*]C=[*]	0.0915
**Top Features for negative contribution**			
**Fingerprint**	**Bit/Smiles**	**Feature Structure**	**Score**
FCFP_6	1	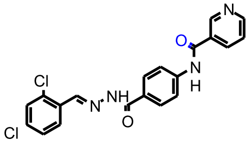<br>[*]=O	-0.102
ECFP_6	-1087070950	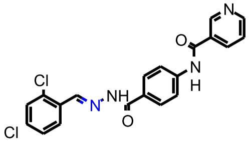<br>[*]N=[*]	-0.102

FCFP_6	-453677277	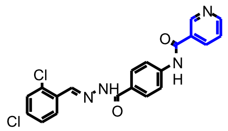<br>[*]C(=[*])[c]1:[cH]:[<br>*]:[cH]:[cH]:[cH]:1	-0.0906

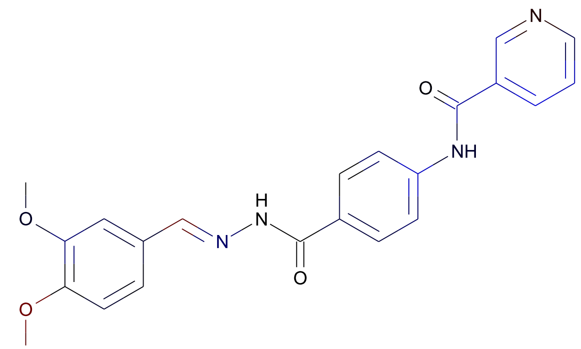

$C_{22}H_{20}N_4O_4$
Molecular Weight: 404.4186
ALogP: 2.284
Rotatable Bonds: 7
Acceptors: 6
Donors: 2

Model Prediction

Prediction: 0.103
Unit: g/kg_body_weight
Mahalanobis Distance: 29.4
Mahalanobis Distance p-value: 1.73e-023

Mahalanobis Distance: The Mahalanobis distance (MD) is a generalization of the Euclidean distance that accounts for correlations among the X properties. It is calculated as the distance to the center of the training data. The larger the MD, the less trustworthy the prediction.
Mahalanobis Distance p-value: The p-value gives the fraction of training data with an MD greater than or equal to the one for the given sample, assuming normally distributed data. The smaller the p-value, the less trustworthy the prediciton. For highly non-normal X properties (e.g., fingerprints), the MD p-value is wildly inaccurate.

Structural Similar Compounds

Name	ISOXABEN	CHLORSULFURON	GLIPIZIDE
Structure	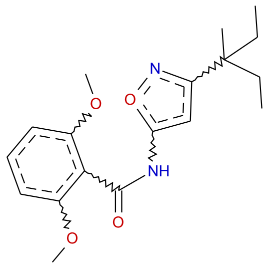	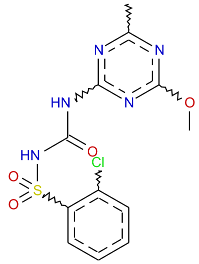	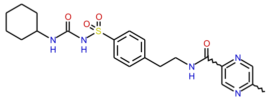
Actual Endpoint (-log C)	3.81665	4.15566	3.94991
Predicted Endpoint (-log C)	4.42315	3.79771	3.95594
Distance	0.688	0.694	0.703
Reference	EPA COVER SHEET 0339;881201;(1)	EPA COVER SHEET 0027;880301;(1)	NDA-17583

Model Applicability

Unknown features are fingerprint features in the query molecule, but not found or appearing too infrequently in the training set.

1. OPS PC18 out of range. Value: -5.3082. Training min, max, SD, explained variance: -4.7991, 6.1674, 1.831, 0.0147.
2. Unknown ECFP_6 feature: 1997021792: [*]:[cH]:[cH]:[cH]:[*]
3. Unknown ECFP_6 feature: 1996163143: [*]:[cH]:[cH]:n:[*]
4. Unknown ECFP_6 feature: -677055651: [*]:[cH]:n:[cH]:[*]
5. Unknown ECFP_6 feature: -709633021: [*][c](:[*]):[cH]:n:[*]
6. Unknown ECFP_6 feature: -175146122: [*]C(=[*])[c](:[cH]:[*]):[cH]:[*]
7. Unknown ECFP_6 feature: 1430169877: [*]NC(=O)[c](:[*]):[*]
8. Unknown ECFP_6 feature: -177077903: [*]N[c](:[cH]:[*]):[cH]:[*]
9. Unknown ECFP_6 feature: 544048674: [*]C(=[*])NN=[*]
10. Unknown ECFP_6 feature: 1814278164: [*]N\N=C\[*]
11. Unknown ECFP_6 feature: -1832102709: [*]\N=C\[c](:[*]):[*]
12. Unknown ECFP_6 feature: -176483725: [*]=C[c](:[cH]:[*]):[cH]:[*]
13. Unknown ECFP_6 feature: 1307307440: [*]:[c](:[*])OC

Feature Contribution

Top features for positive contribution			
Fingerprint	Bit/Smiles	Feature Structure	Score

FCFP_6	3	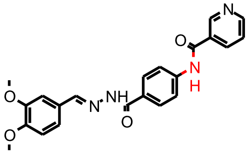 [*]N[*]	0.0924
ECFP_6	-1925046727	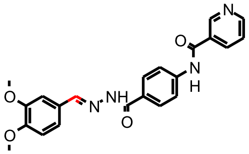 [*]C=[*]	0.0915
ECFP_6	2099970318	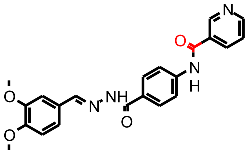 [*]C(=O)[*]	0.0766

Top Features for negative contribution

Fingerprint	Bit/Smiles	Feature Structure	Score
FCFP_6	1	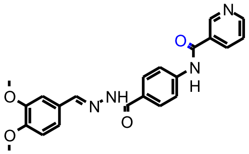 [*]=O	-0.102
ECFP_6	-1087070950	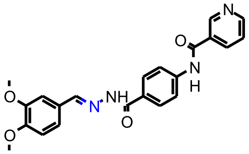 [*]N=[*]	-0.102

FCFP_6	-453677277	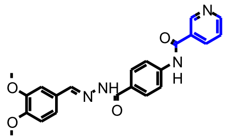 [*]C(=[*])[c]1:[cH]:[*]:[cH]:[cH]:[cH]:1	-0.0906

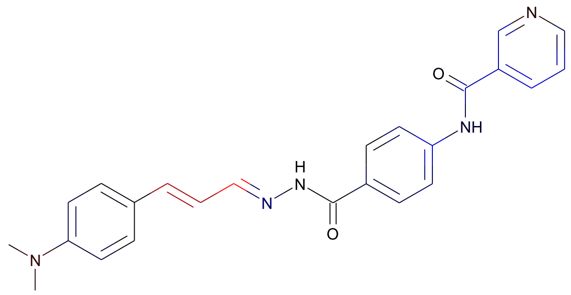

$C_{24}H_{23}N_5O_2$
Molecular Weight: 413.47172
ALogP: 2.839
Rotatable Bonds: 7
Acceptors: 5
Donors: 2

Structural Similar Compounds

Name	ISOXABEN	DILTIAZEM	DISOPYRAMIDE.PHOSPHATE
Structure	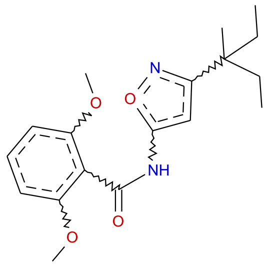	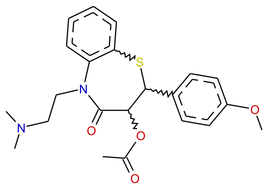	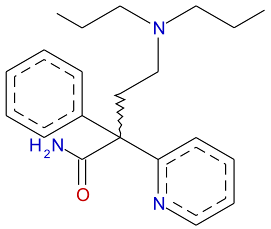
Actual Endpoint (-log C)	3.81665	4.21961	3.22979
Predicted Endpoint (-log C)	4.42315	4.005	3.38887
Distance	0.690	0.719	0.738
Reference	EPA COVER SHEET 0339;881201;(1)	NDA-18602	NDA-17447

Model Prediction

Prediction: 0.055
Unit: g/kg_body_weight
Mahalanobis Distance: 29.8
Mahalanobis Distance p-value: 2.44e-024

Mahalanobis Distance: The Mahalanobis distance (MD) is a generalization of the Euclidean distance that accounts for correlations among the X properties. It is calculated as the distance to the center of the training data. The larger the MD, the less trustworthy the prediction.
Mahalanobis Distance p-value: The p-value gives the fraction of training data with an MD greater than or equal to the one for the given sample, assuming normally distributed data. The smaller the p-value, the less trustworthy the prediciton. For highly non-normal X properties (e.g., fingerprints), the MD p-value is wildly inaccurate.

Model Applicability

Unknown features are fingerprint features in the query molecule, but not found or appearing too infrequently in the training set.

1. All properties and OPS components are within expected ranges.
2. Unknown ECFP_6 feature: 1997021792: [*]:[cH]:[cH]:[cH]:[*]
3. Unknown ECFP_6 feature: 1996163143: [*]:[cH]:[cH]:n:[*]
4. Unknown ECFP_6 feature: -677055651: [*]:[cH]:n:[cH]:[*]
5. Unknown ECFP_6 feature: -709633021: [*][c](:[*]):[cH]:n:[*]
6. Unknown ECFP_6 feature: -175146122: [*]C(=[*])[c](:[cH]:[*]):[cH]:[*]
7. Unknown ECFP_6 feature: 1430169877: [*]NC(=O)[c](:[*]):[*]
8. Unknown ECFP_6 feature: -177077903: [*]N[c](:[cH]:[*]):[cH]:[*]
9. Unknown ECFP_6 feature: 544048674: [*]C(=[*])NN=[*]
10. Unknown ECFP_6 feature: 1814278164: [*]N\N=C\[*]
11. Unknown ECFP_6 feature: -984423246: [*]\N=C\C=[*]
12. Unknown ECFP_6 feature: -471753896: [*]\C=C\C=[*]
13. Unknown ECFP_6 feature: -1831055759: [*]\C=C\[c](:[*]):[*]
14. Unknown ECFP_6 feature: -176483725: [*]=C[c](:[cH]:[*]):[cH]:[*]
15. Unknown ECFP_6 feature: -175985444: [*]N([*])[c](:[cH]:[*]):[cH]:[*]
16. Unknown ECFP_6 feature: -1328426694: [*]:[c](:[*])N(C)C

Feature Contribution

Top features for positive contribution

Fingerprint	Bit/Smiles	Feature Structure	Score
FCFP_6	451847724	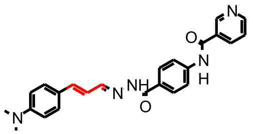 [*]\C=C\C=[*]	0.16
FCFP_6	3	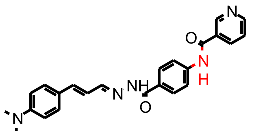 [*]N[*]	0.0924
ECFP_6	-1925046727	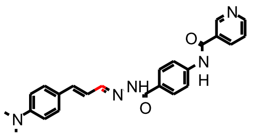 [*]C=[*]	0.0915

Top Features for negative contribution

Fingerprint	Bit/Smiles	Feature Structure	Score
FCFP_6	1	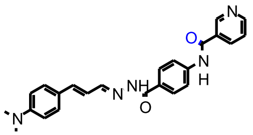 [*]=O	-0.102

ECFP_6	-1087070950	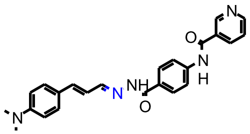 [*]N=[*]	-0.102
FCFP_6	-453677277	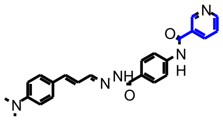 [*]C(=[*])[c]1:[cH]:[*]:[cH]:[cH]:[cH]:1	-0.0906

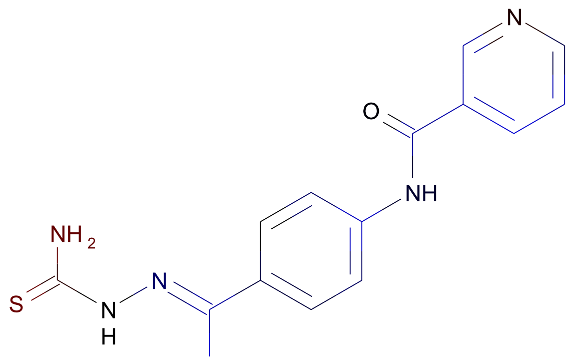

$C_{15}H_{15}N_5OS$
Molecular Weight: 313.3775
ALogP: 1.414
Rotatable Bonds: 5
Acceptors: 4
Donors: 3

Structural Similar Compounds

Name	FUROSEMIDE	PIROXICAM	CHLORSULFURON
Structure	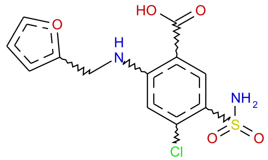	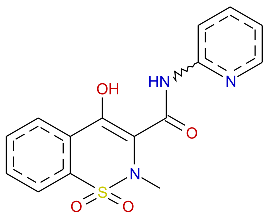	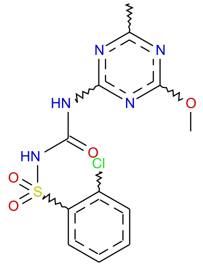
Actual Endpoint (-log C)	4.27645	5.52028	4.15566
Predicted Endpoint (-log C)	4.40005	4.06087	3.79771
Distance	0.482	0.624	0.642
Reference	NTP REPORT # 356	NDA-18147	EPA COVER SHEET 0027;880301;(1)

Model Prediction

Prediction: 0.131
Unit: g/kg_body_weight
Mahalanobis Distance: 29.4
Mahalanobis Distance p-value: 1.4e-023

Mahalanobis Distance: The Mahalanobis distance (MD) is a generalization of the Euclidean distance that accounts for correlations among the X properties. It is calculated as the distance to the center of the training data. The larger the MD, the less trustworthy the prediction.
Mahalanobis Distance p-value: The p-value gives the fraction of training data with an MD greater than or equal to the one for the given sample, assuming normally distributed data. The smaller the p-value, the less trustworthy the prediciton. For highly non-normal X properties (e.g., fingerprints), the MD p-value is wildly inaccurate.

Model Applicability

Unknown features are fingerprint features in the query molecule, but not found or appearing too infrequently in the training set.

1. All properties and OPS components are within expected ranges.
2. Unknown ECFP_6 feature: 1997021792: [*]:[cH]:[cH]:[cH]:[*]
3. Unknown ECFP_6 feature: 1996163143: [*]:[cH]:[cH]:n:[*]
4. Unknown ECFP_6 feature: -677055651: [*]:[cH]:n:[cH]:[*]
5. Unknown ECFP_6 feature: -709633021: [*][c](:[*]):[cH]:n:[*]
6. Unknown ECFP_6 feature: -175146122: [*]C(=[*])[c](:[cH]:[*]):[cH]:[*]
7. Unknown ECFP_6 feature: 1430169877: [*]NC(=O)[c](:[*]):[*]
8. Unknown ECFP_6 feature: -177077903: [*]N[c](:[cH]:[*]):[cH]:[*]
9. Unknown ECFP_6 feature: 128986386: [*]\N=C(/C)\[c](:[*]):[*]
10. Unknown ECFP_6 feature: 560380707: [*]NN=C([*])[*]
11. Unknown ECFP_6 feature: 544048674: [*]C(=[*])NN=[*]
12. Unknown ECFP_6 feature: -571028867: [*]NC(=S)N
13. Unknown ECFP_6 feature: -932108170: [*]C(=[*])N
14. Unknown ECFP_6 feature: 1979182050: [*]C(=S)[*]

Feature Contribution

Top features for positive contribution			
**Fingerprint**	**Bit/Smiles**	**Feature Structure**	**Score**

ECFP_6	-845108448	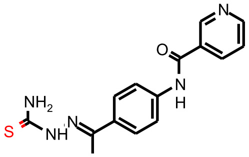 [*]=S	0.105
FCFP_6	3	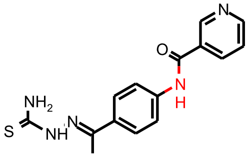 [*]N[*]	0.0924
ECFP_6	2099970318	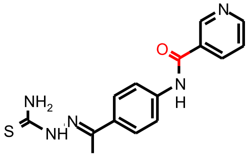 [*]C(=O)[*]	0.0766

Top Features for negative contribution

**Fingerprint**	**Bit/Smiles**	**Feature Structure**	**Score**
FCFP_6	1	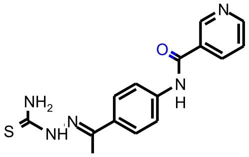 [*]=O	-0.102
ECFP_6	-1087070950	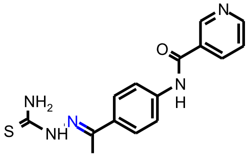 [*]N=[*]	-0.102

FCFP_6	-453677277	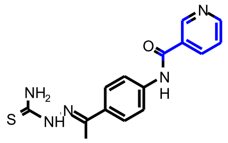 [*]C(=[*])[c]1:[cH]:[*]:[cH]:[cH]:[cH]:1	-0.0906

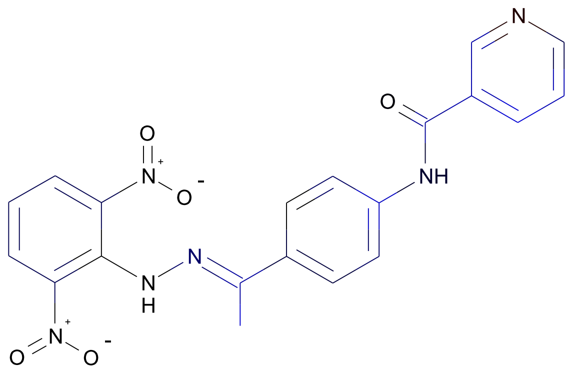

$C_{20}H_{16}N_6O_5$
Molecular Weight: 420.37824
ALogP: 2.852
Rotatable Bonds: 7
Acceptors: 8
Donors: 2

Model Prediction

Prediction: 0.231
Unit: g/kg_body_weight
Mahalanobis Distance: 29.6
Mahalanobis Distance p-value: 6.28e-024

Mahalanobis Distance: The Mahalanobis distance (MD) is a generalization of the Euclidean distance that accounts for correlations among the X properties. It is calculated as the distance to the center of the training data. The larger the MD, the less trustworthy the prediction.
Mahalanobis Distance p-value: The p-value gives the fraction of training data with an MD greater than or equal to the one for the given sample, assuming normally distributed data. The smaller the p-value, the less trustworthy the prediciton. For highly non-normal X properties (e.g., fingerprints), the MD p-value is wildly inaccurate.

Structural Similar Compounds

Name	C.I. ACID ORANGE 3	ALLY	C.I. ACID ORANGE 10
Structure	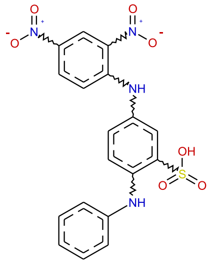	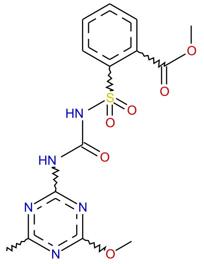	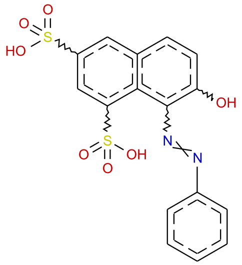
Actual Endpoint (-log C)	3.20573	3.1834	3.435
Predicted Endpoint (-log C)	3.55956	3.59541	3.28295
Distance	0.600	0.654	0.656
Reference	NTP REPORT # 335	EPA COVER SHEET 0288;891101;(1)	NTP REPORT # 211

Model Applicability

Unknown features are fingerprint features in the query molecule, but not found or appearing too infrequently in the training set.

1. All properties and OPS components are within expected ranges.
2. Unknown FCFP_2 feature: 5: [*][O-]
3. Unknown FCFP_2 feature: 1294285001: [*]=NN[c](:[*]):[*]
4. Unknown FCFP_2 feature: -828984032: [*][c](:[*]):[c]([N+](=[*])[*]):c:[*]
5. Unknown FCFP_2 feature: -1338588315: [*]:[c](:[*])[N+](=O)[O-]
6. Unknown FCFP_2 feature: 1872392852: [*][N+](=O)[*]
7. Unknown FCFP_2 feature: 260476081: [*][N+](=[*])[O-]
8. Unknown ECFP_6 feature: 1043790491: [*][N+](=[*])[*]
9. Unknown ECFP_6 feature: 781519895: [*][O-]
10. Unknown ECFP_6 feature: 1997021792: [*]:[cH]:[cH]:[cH]:[*]
11. Unknown ECFP_6 feature: 1996163143: [*]:[cH]:[cH]:n:[*]
12. Unknown ECFP_6 feature: -677055651: [*]:[cH]:n:[cH]:[*]
13. Unknown ECFP_6 feature: -709633021: [*][c](:[*]):[cH]:n:[*]
14. Unknown ECFP_6 feature: -175146122: [*]C(=[*])[c](:[cH]:[*]):[cH]:[*]
15. Unknown ECFP_6 feature: 1430169877: [*]NC(=O)[c](:[*]):[*]
16. Unknown ECFP_6 feature: -177077903: [*]N[c](:[cH]:[*]):[cH]:[*]
17. Unknown ECFP_6 feature: 128986386: [*]\N=C(/C)\[c](:[*]):[*]
18. Unknown ECFP_6 feature: 560380707: [*]NN=C([*])[*]
19. Unknown ECFP_6 feature: -1236714312: [*]=NN[c](:[*]):[*]

20. Unknown ECFP_6 feature: -1660205591: [*]N[c](:[c]([*]):[*]):[c]([*]):[*]
21. Unknown ECFP_6 feature: -1956535100: [*][c](:[*]):[c](:[cH]:[*])[N+](=[*])[*]
22. Unknown ECFP_6 feature: -215026467: [*]:[c](:[*])[N+](=O)[O-]
23. Unknown ECFP_6 feature: 2104376220: [*][N+](=O)[*]
24. Unknown ECFP_6 feature: -659271057: [*][N+](=[*])[O-]

Feature Contribution

Top features for positive contribution

Fingerprint	Bit/Smiles	Feature Structure	Score
FCFP_6	3	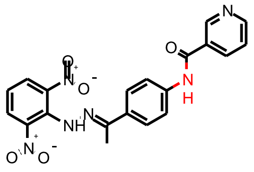 [*]N[*]	0.0924
ECFP_6	2099970318	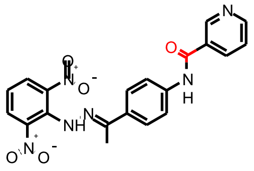 [*]C(=O)[*]	0.0766
ECFP_6	2007300961	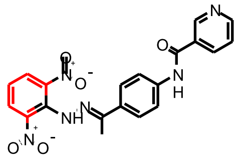 [*][c]1:[*]:[c]([*]):[cH]:[cH]:[cH]:1	0.0564

Top Features for negative contribution

Fingerprint	Bit/Smiles	Feature Structure	Score

FCFP_6	991735244	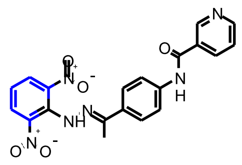 [*][c]1:[*]:[c]([*]):[cH]:[cH]:[cH]:1	-0.134
FCFP_6	1	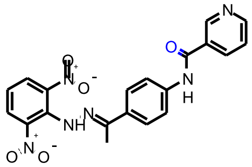 [*]=O	-0.102
ECFP_6	-1087070950	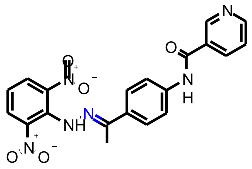 [*]N=[*]	-0.102

Sorafenib

TOPKAT_Chronic_LOAEL

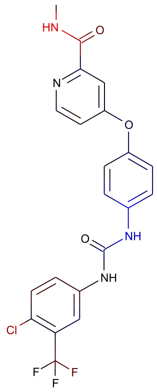

$C_{21}H_{16}ClF_3N_4O_3$

Molecular Weight: 464.82494

ALogP: 4.175

Rotatable Bonds: 6

Acceptors: 4

Donors: 3

Model Prediction

Prediction: 0.00483

Unit: g/kg_body_weight

Mahalanobis Distance: 30

Mahalanobis Distance p-value: 1.21e-024

Mahalanobis Distance: The Mahalanobis distance (MD) is a generalization of the Euclidean distance that accounts for correlations among the X properties. It is calculated as the distance to the center of the training data. The larger the MD, the less trustworthy the prediction.

Mahalanobis Distance p-value: The p-value gives the fraction of training data with an MD greater than or equal to the one for the given sample, assuming normally distributed data. The smaller the p-value, the less trustworthy the prediciton. For highly non-normal X properties (e.g., fingerprints), the MD p-value is wildly inaccurate.

Structural Similar Compounds

Name	GLYBURIDE	D & C RED 9	SODIUM ACIFLUORFEN
Structure	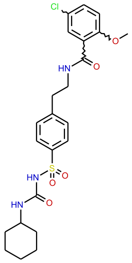	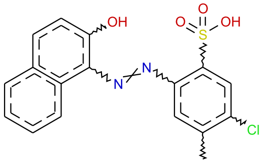	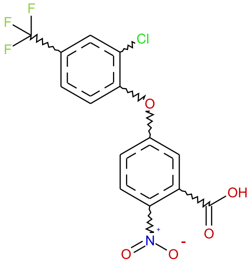
Actual Endpoint (-log C)	4.21661	3.87715	4.16036
Predicted Endpoint (-log C)	4.21035	3.6546	4.65915
Distance	0.636	0.722	0.736
Reference	UPJ-26452	NTP REPORT # 225	EPA COVER SHEET 0192;891101;(1)

Model Applicability

Unknown features are fingerprint features in the query molecule, but not found or appearing too infrequently in the training set.

1. All properties and OPS components are within expected ranges.
2. Unknown ECFP_6 feature: -1046436026: [*]F
3. Unknown ECFP_6 feature: 1305253718: [*]:[c](:[*])O[c](:[*]):[*]
4. Unknown ECFP_6 feature: 1413420509: [*]C(=[*])[c](:[cH]:[*]):n:[*]
5. Unknown ECFP_6 feature: -677309799: [*][c](:[*]):n:[cH]:[*]
6. Unknown ECFP_6 feature: 1996163143: [*]:[cH]:[cH]:n:[*]
7. Unknown ECFP_6 feature: 1430169877: [*]NC(=O)[c](:[*]):[*]
8. Unknown ECFP_6 feature: 1338334141: [*]C(=[*])NC
9. Unknown ECFP_6 feature: 864287155: [*]NC
10. Unknown ECFP_6 feature: -177077903: [*]N[c](:[cH]:[*]):[cH]:[*]
11. Unknown ECFP_6 feature: -649580166: [*]NC(=O)N[*]
12. Unknown ECFP_6 feature: 1336678434: [*][c](:[*]):[c](:[cH]:[*])C([*])([*])[*]
13. Unknown ECFP_6 feature: 99947387: [*]:[c](:[*])Cl
14. Unknown ECFP_6 feature: -1952889961: [*]:[c](:[*])C(F)(F)F
15. Unknown ECFP_6 feature: 226796801: [*]C([*])([*])F

Feature Contribution

Top features for positive contribution

Fingerprint	Bit/Smiles	Feature Structure	Score
ECFP_6	-176455838	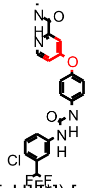 [*]O[c](:[cH]:[*]):[cH]:[*]	0.106
FCFP_6	32	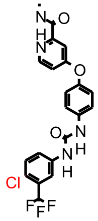 [*]Cl	0.101
FCFP_6	3	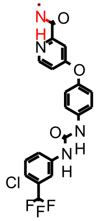 [*]N[*]	0.0924

Top Features for negative contribution

Fingerprint	Bit/Smiles	Feature Structure	Score
FCFP_6	1	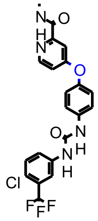 [*]=O	-0.102

ECFP_6	-1236483485	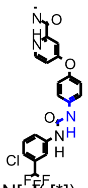 [*]C(=[*])N[c](:[*]):[*]	-0.0747
FCFP_6	203677720	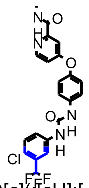 [*]C(=[*])[c](:[cH]:[*]):[cH]:[*]	-0.0713

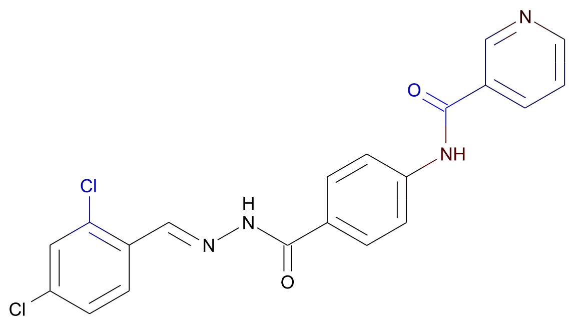

$C_{20}H_{14}Cl_2N_4O_2$

Molecular Weight: 413.25676

ALogP: 3.646

Rotatable Bonds: 5

Acceptors: 4

Donors: 2

Model Prediction

Prediction: 0.125

Unit: g/kg_body_weight

Mahalanobis Distance: 7.9

Mahalanobis Distance p-value: 0.0309

Mahalanobis Distance: The Mahalanobis distance (MD) is a generalization of the Euclidean distance that accounts for correlations among the X properties. It is calculated as the distance to the center of the training data. The larger the MD, the less trustworthy the prediction.

Mahalanobis Distance p-value: The p-value gives the fraction of training data with an MD greater than or equal to the one for the given sample, assuming normally distributed data. The smaller the p-value, the less trustworthy the prediciton. For highly non-normal X properties (e.g., fingerprints), the MD p-value is wildly inaccurate.

Structural Similar Compounds

Name	PHENOLPHTHALEIN	1-AMINO-2,4-DIBROMOANTHRAQUINONE	OXAZEPAM
Structure	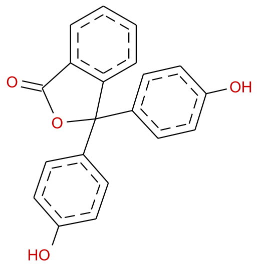	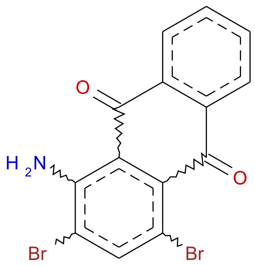	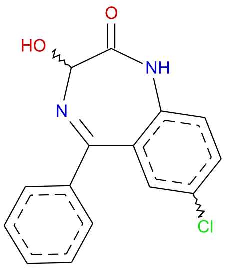
Actual Endpoint (-log C)	2.20184	2.82966	3.05262
Predicted Endpoint (-log C)	2.8857	3.92444	3.13073
Distance	0.623	0.640	0.651
Reference	NCI/NTP TR-465	NCI/NTP TR-383	NCI/NTP TR-468

Model Applicability

Unknown features are fingerprint features in the query molecule, but not found or appearing too infrequently in the training set.

1. All properties and OPS components are within expected ranges.

Feature Contribution

Top features for positive contribution

Fingerprint	Bit/Smiles	Feature Structure	Score
FCFP_2	3	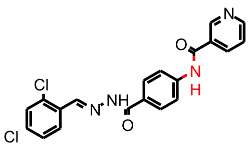 [*]N[*]	0.0737

FCFP_2	17	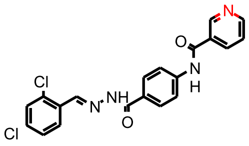 [*]:n:[*]	0.0441
FCFP_2	590925877	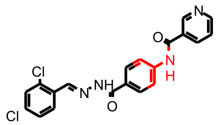 [*]N[c](:[cH]:[*]):[cH]:[*]	0.00762

Top Features for negative contribution

Fingerprint	Bit/Smiles	Feature Structure	Score
FCFP_2	71476542	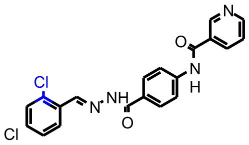 [*]:[c](:[*])Cl	-0.134
FCFP_2	1872154524	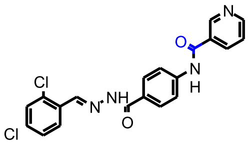 [*]C(=O)[*]	-0.105
FCFP_2	203677720	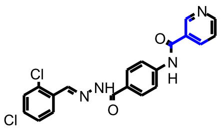 [*]C(=[*])[c](:[cH]:[*]):[cH]:[*]	-0.0829

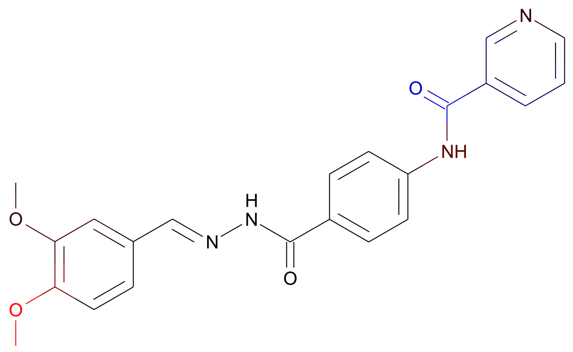

$C_{22}H_{20}N_4O_4$

Molecular Weight: 404.4186

ALogP: 2.284

Rotatable Bonds: 7

Acceptors: 6

Donors: 2

Model Prediction

Prediction: 0.0578

Unit: g/kg_body_weight

Mahalanobis Distance: 7.81

Mahalanobis Distance p-value: 0.039

Mahalanobis Distance: The Mahalanobis distance (MD) is a generalization of the Euclidean distance that accounts for correlations among the X properties. It is calculated as the distance to the center of the training data. The larger the MD, the less trustworthy the prediction.
Mahalanobis Distance p-value: The p-value gives the fraction of training data with an MD greater than or equal to the one for the given sample, assuming normally distributed data. The smaller the p-value, the less trustworthy the prediciton. For highly non-normal X properties (e.g., fingerprints), the MD p-value is wildly inaccurate.

Structural Similar Compounds

Name	SALICYLAZOSULFAPYRIDINE	FUROSEMIDE	BENZIDINE,3,3'-DIMETHOXY-
Structure	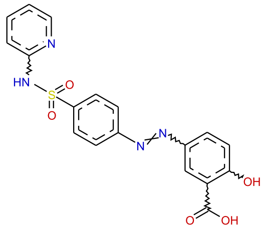	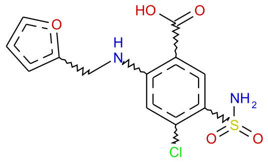	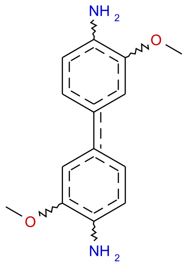
Actual Endpoint (-log C)	3.375	4.04236	4.06569
Predicted Endpoint (-log C)	2.80292	2.8614	3.57405
Distance	0.687	0.724	0.745
Reference	NCI/NTP TR-457	NCI/NTP TR-356	NCI/NTP Report 10, Nov. 1987

Model Applicability

Unknown features are fingerprint features in the query molecule, but not found or appearing too infrequently in the training set.

1. OPS PC9 out of range. Value: 3.9949. Training min, max, SD, explained variance: -2.8548, 3.3954, 1.263, 0.0360.

Feature Contribution

Top features for positive contribution

Fingerprint	Bit/Smiles	Feature Structure	Score
FCFP_2	136627117	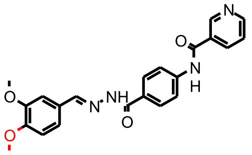 [*]OC	0.173

FCFP_2	1036089772	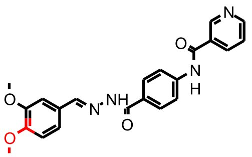 [*]:[c](:[*])OC	0.0749
FCFP_2	3	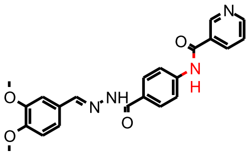 [*]N[*]	0.0737
**Top Features for negative contribution**			
**Fingerprint**	**Bit/Smiles**	**Feature Structure**	**Score**
FCFP_2	1872154524	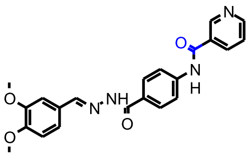 [*]C(=O)[*]	-0.105
FCFP_2	203677720	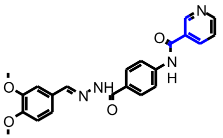 [*]C(=[*])[c](:[cH]:[*]):[cH]:[*]	-0.0829
FCFP_2	1	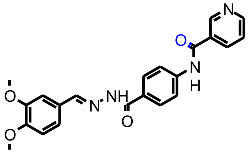 [*]=O	-0.0796

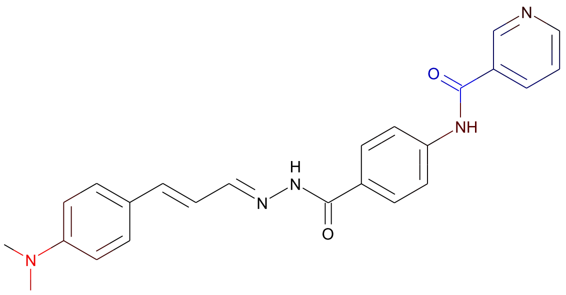

$C_{24}H_{23}N_5O_2$

Molecular Weight: 413.47172

ALogP: 2.839

Rotatable Bonds: 7

Acceptors: 5

Donors: 2

Model Prediction

Prediction: 0.0637

Unit: g/kg_body_weight

Mahalanobis Distance: 8.71

Mahalanobis Distance p-value: 0.00351

Mahalanobis Distance: The Mahalanobis distance (MD) is a generalization of the Euclidean distance that accounts for correlations among the X properties. It is calculated as the distance to the center of the training data. The larger the MD, the less trustworthy the prediction.
Mahalanobis Distance p-value: The p-value gives the fraction of training data with an MD greater than or equal to the one for the given sample, assuming normally distributed data. The smaller the p-value, the less trustworthy the prediciton. For highly non-normal X properties (e.g., fingerprints), the MD p-value is wildly inaccurate.

Structural Similar Compounds

Name	DISPERSE YELLOW 3	PHENOLPHTHALEIN	CINNAMYL ANTHRANILATE
Structure	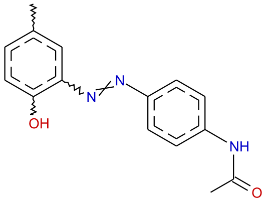	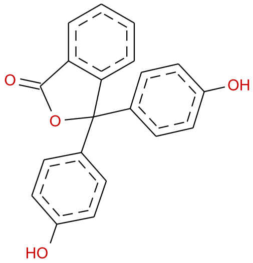	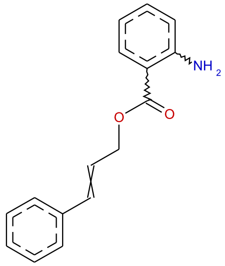
Actual Endpoint (-log C)	2.77703	2.20184	2.2733
Predicted Endpoint (-log C)	2.80195	2.8857	3.25473
Distance	0.727	0.745	0.746
Reference	NCI/NTP TR-222	NCI/NTP TR-465	NCI/NTP TR-196

Model Applicability

Unknown features are fingerprint features in the query molecule, but not found or appearing too infrequently in the training set.

1. All properties and OPS components are within expected ranges.

Feature Contribution

Top features for positive contribution

Fingerprint	Bit/Smiles	Feature Structure	Score
FCFP_2	136627117	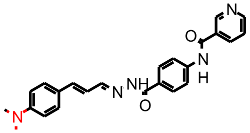 [*]OC	0.173

FCFP_2	3	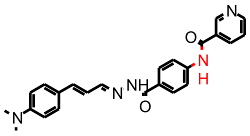<br>[*]N[*]	0.0737
FCFP_2	332760439	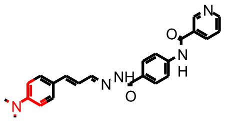<br>[*]O[c](:[cH]:[*]):[c]([*]):[*]	0.0611

Top Features for negative contribution

Fingerprint	Bit/Smiles	Feature Structure	Score
FCFP_2	1872154524	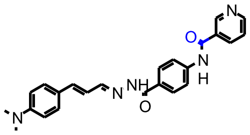<br>[*]C(=O)[*]	-0.105
FCFP_2	203677720	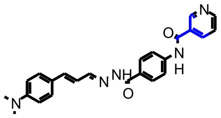<br>[*]C(=[*])[c](:[cH]:[*]):[cH]:[*]	-0.0829
FCFP_2	1	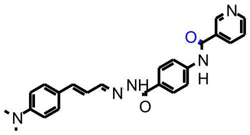<br>[*]=O	-0.0796

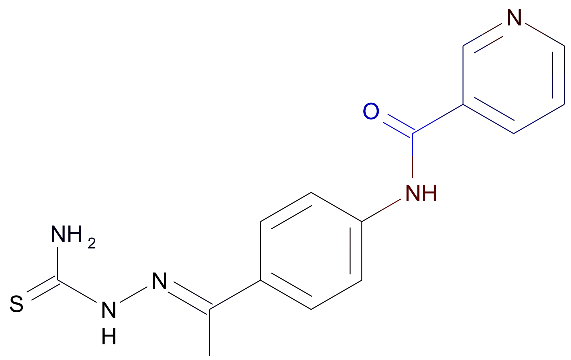

$C_{15}H_{15}N_5OS$
Molecular Weight: 313.3775
ALogP: 1.414
Rotatable Bonds: 5
Acceptors: 4
Donors: 3

Model Prediction

Prediction: 0.146
Unit: g/kg_body_weight
Mahalanobis Distance: 8.01
Mahalanobis Distance p-value: 0.024

Mahalanobis Distance: The Mahalanobis distance (MD) is a generalization of the Euclidean distance that accounts for correlations among the X properties. It is calculated as the distance to the center of the training data. The larger the MD, the less trustworthy the prediction.
Mahalanobis Distance p-value: The p-value gives the fraction of training data with an MD greater than or equal to the one for the given sample, assuming normally distributed data. The smaller the p-value, the less trustworthy the prediciton. For highly non-normal X properties (e.g., fingerprints), the MD p-value is wildly inaccurate.

Structural Similar Compounds

Name	FUROSEMIDE	NITROFURAZONE	DAPSONE
Structure	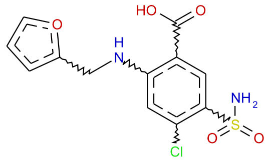	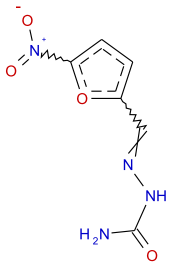	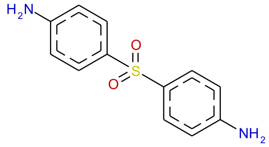
Actual Endpoint (-log C)	4.04236	4.21779	3.66258
Predicted Endpoint (-log C)	2.8614	3.40885	3.26993
Distance	0.371	0.582	0.595
Reference	NCI/NTP TR-356	NCI/NTP TR-337	NCI/NTP TR-20

Model Applicability

Unknown features are fingerprint features in the query molecule, but not found or appearing too infrequently in the training set.

1. All properties and OPS components are within expected ranges.

Feature Contribution

Top features for positive contribution			
**Fingerprint**	**Bit/Smiles**	**Feature Structure**	**Score**
FCFP_2	3	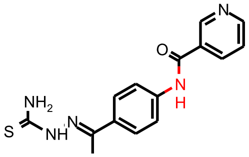 [*]N[*]	0.0737

FCFP_2	17	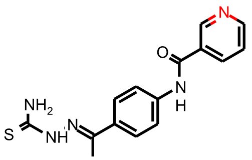 [*]:n:[*]	0.0441
FCFP_2	590925877	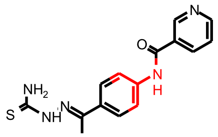 [*]N[c](:[cH]:[*]):[cH]:[*]	0.00762
**Top Features for negative contribution**			
**Fingerprint**	**Bit/Smiles**	**Feature Structure**	**Score**
FCFP_2	1872154524	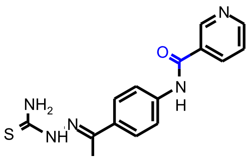 [*]C(=O)[*]	-0.105
FCFP_2	203677720	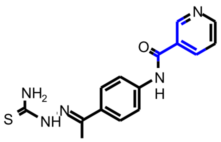 [*]C(=[*])[c](:[cH]:[*]):[cH]:[*]	-0.0829
FCFP_2	1	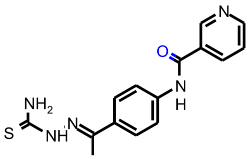 [*]=O	-0.0796

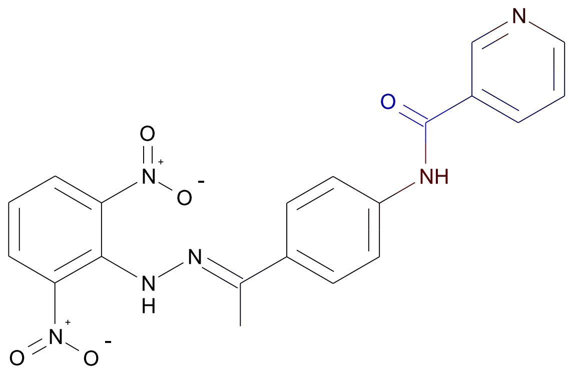

$C_{20}H_{16}N_6O_5$
Molecular Weight: 420.37824
ALogP: 2.852
Rotatable Bonds: 7
Acceptors: 8
Donors: 2

Model Prediction

Prediction: 0.111
Unit: g/kg_body_weight
Mahalanobis Distance: 7.54
Mahalanobis Distance p-value: 0.0702

Mahalanobis Distance: The Mahalanobis distance (MD) is a generalization of the Euclidean distance that accounts for correlations among the X properties. It is calculated as the distance to the center of the training data. The larger the MD, the less trustworthy the prediction.
Mahalanobis Distance p-value: The p-value gives the fraction of training data with an MD greater than or equal to the one for the given sample, assuming normally distributed data. The smaller the p-value, the less trustworthy the prediciton. For highly non-normal X properties (e.g., fingerprints), the MD p-value is wildly inaccurate.

Structural Similar Compounds

Name	SALICYLAZOSULFAPYRIDINE	C.I.PIGMENT RED 23	FUROSEMIDE
Structure	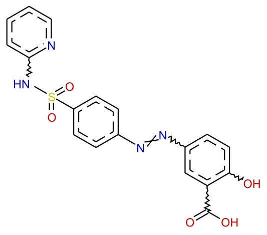	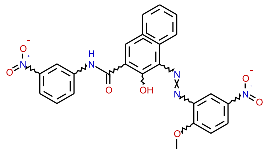	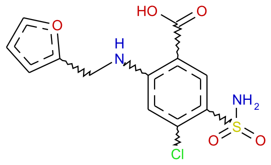
Actual Endpoint (-log C)	3.375	2.30052	4.04236
Predicted Endpoint (-log C)	2.80292	3.55333	2.8614
Distance	0.506	0.697	0.754
Reference	NCI/NTP TR-457	NCI/NTP TR-411	NCI/NTP TR-356

Model Applicability

Unknown features are fingerprint features in the query molecule, but not found or appearing too infrequently in the training set.

1. All properties and OPS components are within expected ranges.
2. Unknown FCFP_2 feature: 8: [*][N+](=[*])[*]
3. Unknown FCFP_2 feature: 5: [*][O-]
4. Unknown FCFP_2 feature: 1294285001: [*]=NN[c](:[*]):[*]
5. Unknown FCFP_2 feature: -828984032: [*][c](:[*]):[c]([N+](=[*])[*]):c:[*]
6. Unknown FCFP_2 feature: -1338588315: [*]:[c](:[*])[N+](=O)[O-]
7. Unknown FCFP_2 feature: 1872392852: [*][N+](=O)[*]
8. Unknown FCFP_2 feature: 260476081: [*][N+](=[*])[O-]

Feature Contribution

Top features for positive contribution

Fingerprint	Bit/Smiles	Feature Structure	Score

FCFP_2	3	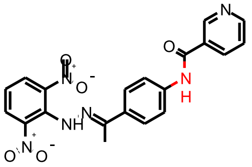 [*]N[*]	0.0737
FCFP_2	17	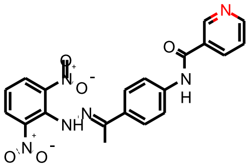 [*]:n:[*]	0.0441
FCFP_2	590925877	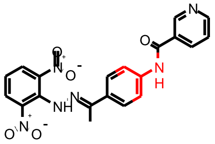 [*]N[c](:[cH]:[*]):[cH]:[*]	0.00762
**Top Features for negative contribution**			
**Fingerprint**	**Bit/Smiles**	**Feature Structure**	**Score**
FCFP_2	1872154524	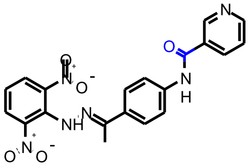 [*]C(=O)[*]	-0.105
FCFP_2	203677720	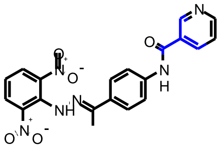 [*]C(=[*])[c](:[cH]:[*]):[cH]:[*]	-0.0829

FCFP_2	1	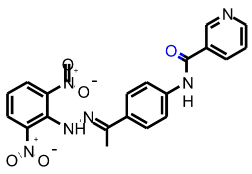 [*]=O	-0.0796

Sorafenib

TOPKAT_Rat_Maximum_Tolerated_Dose_Feed

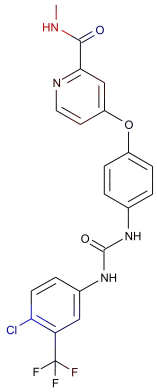

$C_{21}H_{16}ClF_3N_4O_3$

Molecular Weight: 464.82494

ALogP: 4.175

Rotatable Bonds: 6

Acceptors: 4

Donors: 3

Model Prediction

Prediction: 0.0885

Unit: g/kg_body_weight

Mahalanobis Distance: 12.4

Mahalanobis Distance p-value: 1.76e-009

Mahalanobis Distance: The Mahalanobis distance (MD) is a generalization of the Euclidean distance that accounts for correlations among the X properties. It is calculated as the distance to the center of the training data. The larger the MD, the less trustworthy the prediction.
Mahalanobis Distance p-value: The p-value gives the fraction of training data with an MD greater than or equal to the one for the given sample, assuming normally distributed data. The smaller the p-value, the less trustworthy the prediciton. For highly non-normal X properties (e.g., fingerprints), the MD p-value is wildly inaccurate.

Structural Similar Compounds

Name	FUROSEMIDE	PHENOLPHTHALEIN	DISPERSE YELLOW 3
Structure	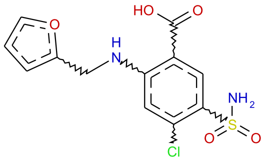	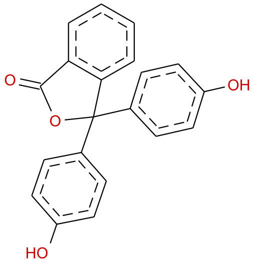	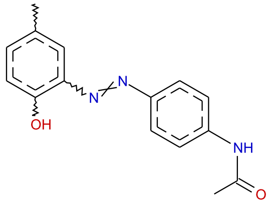
Actual Endpoint (-log C)	4.04236	2.20184	2.77703
Predicted Endpoint (-log C)	2.8614	2.8857	2.80195
Distance	0.741	0.780	0.799
Reference	NCI/NTP TR-356	NCI/NTP TR-465	NCI/NTP TR-222

Model Applicability

Unknown features are fingerprint features in the query molecule, but not found or appearing too infrequently in the training set.

1. All properties and OPS components are within expected ranges.

Feature Contribution

Top features for positive contribution			
**Fingerprint**	**Bit/Smiles**	**Feature Structure**	**Score**
FCFP_2	-885550502	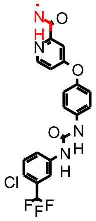 [*]C(=[*])NC	0.115

FCFP_2	3	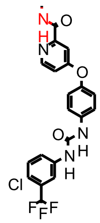 [*]N[*]	0.0737
FCFP_2	332760439	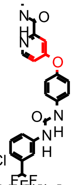 [*]O[c](:[cH]:[*]):[c]([*]):[*]	0.0611
**Top Features for negative contribution**			
**Fingerprint**	**Bit/Smiles**	**Feature Structure**	**Score**
FCFP_2	71476542	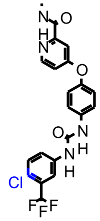 [*]:[c](:[*])Cl	-0.134
FCFP_2	1872154524	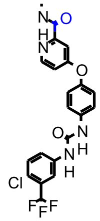 [*]C(=O)[*]	-0.105
FCFP_2	203677720	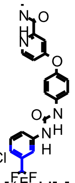 [*]C(=[*])[c](:[cH]:[*]):[cH]:[*]	-0.0829

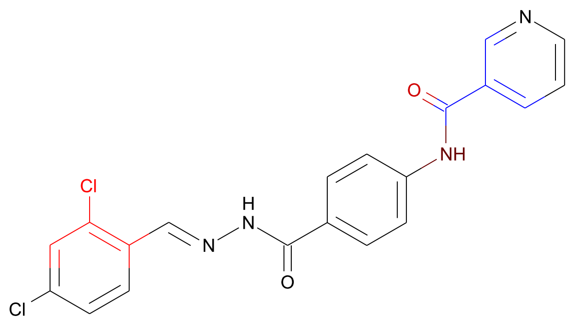

$C_{20}H_{14}Cl_2N_4O_2$

Molecular Weight: 413.25676

ALogP: 3.646

Rotatable Bonds: 5

Acceptors: 4

Donors: 2

Model Prediction

Prediction: 0.00502

Unit: g/kg_body_weight

Mahalanobis Distance: 7.75

Mahalanobis Distance p-value: 0.00403

Mahalanobis Distance: The Mahalanobis distance (MD) is a generalization of the Euclidean distance that accounts for correlations among the X properties. It is calculated as the distance to the center of the training data. The larger the MD, the less trustworthy the prediction.
Mahalanobis Distance p-value: The p-value gives the fraction of training data with an MD greater than or equal to the one for the given sample, assuming normally distributed data. The smaller the p-value, the less trustworthy the prediciton. For highly non-normal X properties (e.g., fingerprints), the MD p-value is wildly inaccurate.

Structural Similar Compounds

Name	OCHRATOXIN	SULFISOOXAZOLE	PROBENECID
Structure	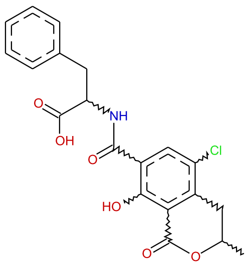	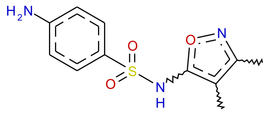	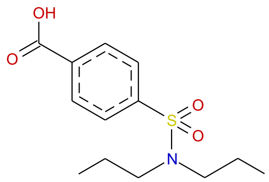
Actual Endpoint (-log C)	6.28396	2.82494	2.85333
Predicted Endpoint (-log C)	5.12358	3.0705	2.4258
Distance	0.803	0.860	1.041
Reference	NCI/NTP TR-358	NCI/NTP TR-138	NCI/NTP TR-395

Model Applicability

Unknown features are fingerprint features in the query molecule, but not found or appearing too infrequently in the training set.

1. Num_AromaticRings out of range. Value: 3. Training min, max, mean, SD: 0, 2, 0.5625, 0.693.
2. OPS PC7 out of range. Value: -3.7431. Training min, max, SD, explained variance: -2.8003, 2.9332, 1.16, 0.0416.
3. Unknown FCFP_2 feature: -885520711: [*]C(=[*])NN=[*]
4. Unknown FCFP_2 feature: 581019816: [*]N\N=C\[*]

Feature Contribution

Top features for positive contribution

Fingerprint	Bit/Smiles	Feature Structure	Score
FCFP_2	32	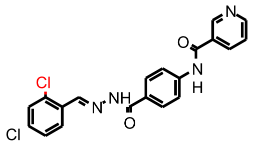 [*]Cl	0.526

FCFP_2	1	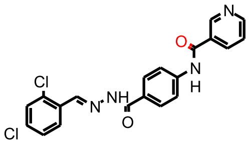 [*]=O	0.511
FCFP_2	367998008	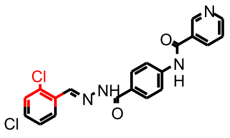 [*][c](:[*]):[c](Cl):[cH]:[*]	0.413
**Top Features for negative contribution**			
**Fingerprint**	**Bit/Smiles**	**Feature Structure**	**Score**
FCFP_2	203677720	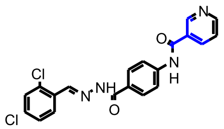 [*]C(=[*])[c](:[cH]:[*]):[cH]:[*]	-0.406
FCFP_2	1872154524	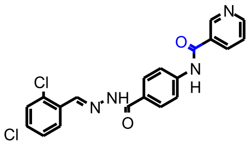 [*]C(=O)[*]	-0.307
FCFP_2	0	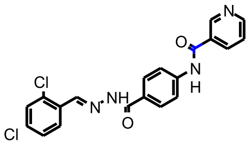 [*]C(=[*])[*]	-0.29

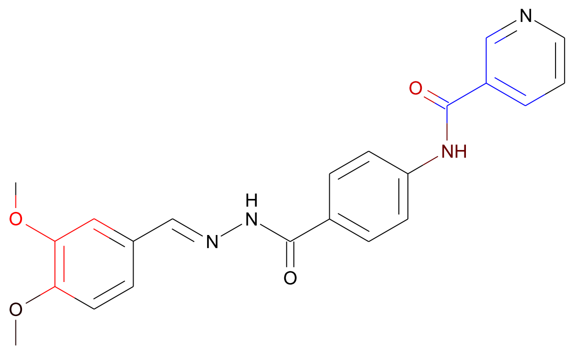

$C_{22}H_{20}N_4O_4$

Molecular Weight: 404.4186

ALogP: 2.284

Rotatable Bonds: 7

Acceptors: 6

Donors: 2

Structural Similar Compounds

Name	OCHRATOXIN	SULFISOOXAZOLE	PENICILLIN VK
Structure	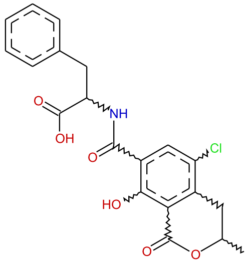	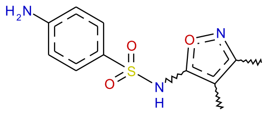	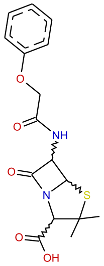
Actual Endpoint (-log C)	6.28396	2.82494	2.54455
Predicted Endpoint (-log C)	5.12358	3.0705	3.9702
Distance	0.798	0.851	0.965
Reference	NCI/NTP TR-358	NCI/NTP TR-138	NCI/NTP TR-336

Model Prediction

Prediction: 0.00276

Unit: g/kg_body_weight

Mahalanobis Distance: 8.67

Mahalanobis Distance p-value: 0.000324

Mahalanobis Distance: The Mahalanobis distance (MD) is a generalization of the Euclidean distance that accounts for correlations among the X properties. It is calculated as the distance to the center of the training data. The larger the MD, the less trustworthy the prediction.
Mahalanobis Distance p-value: The p-value gives the fraction of training data with an MD greater than or equal to the one for the given sample, assuming normally distributed data. The smaller the p-value, the less trustworthy the prediciton. For highly non-normal X properties (e.g., fingerprints), the MD p-value is wildly inaccurate.

Model Applicability

Unknown features are fingerprint features in the query molecule, but not found or appearing too infrequently in the training set.

1. Num_AromaticRings out of range. Value: 3. Training min, max, mean, SD: 0, 2, 0.5625, 0.693.
2. OPS PC6 out of range. Value: -3.1581. Training min, max, SD, explained variance: -2.4321, 2.9885, 1.256, 0.0488.
3. Unknown FCFP_2 feature: -885520711: [*]C(=[*])NN=[*]
4. Unknown FCFP_2 feature: 581019816: [*]N\N=C\[*]

Feature Contribution

Top features for positive contribution

Fingerprint	Bit/Smiles	Feature Structure	Score
FCFP_2	332760439	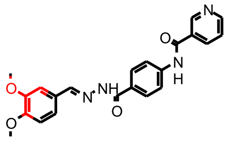 [*]O[c](:[cH]:[*]):[c]([*]):[*]	0.672

FCFP_2	1	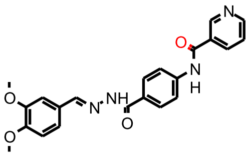 [*]=O	0.511
FCFP_2	3	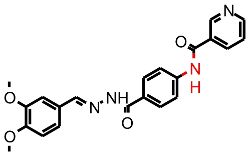 [*]N[*]	0.104

Top Features for negative contribution

Fingerprint	Bit/Smiles	Feature Structure	Score
FCFP_2	203677720	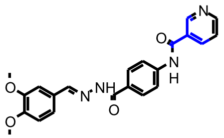 [*]C(=[*])[c](:[cH]:[*]):[cH]:[*]	-0.406
FCFP_2	1872154524	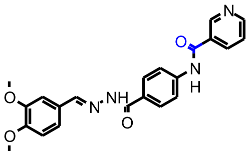 [*]C(=O)[*]	-0.307
FCFP_2	0	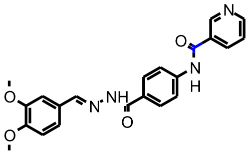 [*]C(=[*])[*]	-0.29

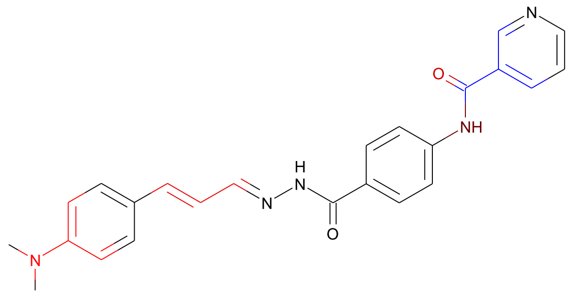

$C_{24}H_{23}N_5O_2$
Molecular Weight: 413.47172
ALogP: 2.839
Rotatable Bonds: 7
Acceptors: 5
Donors: 2

Model Prediction

Prediction: 0.00385
Unit: g/kg_body_weight
Mahalanobis Distance: 9.86
Mahalanobis Distance p-value: 8.78e-006

Mahalanobis Distance: The Mahalanobis distance (MD) is a generalization of the Euclidean distance that accounts for correlations among the X properties. It is calculated as the distance to the center of the training data. The larger the MD, the less trustworthy the prediction.
Mahalanobis Distance p-value: The p-value gives the fraction of training data with an MD greater than or equal to the one for the given sample, assuming normally distributed data. The smaller the p-value, the less trustworthy the prediciton. For highly non-normal X properties (e.g., fingerprints), the MD p-value is wildly inaccurate.

Structural Similar Compounds

Name	OCHRATOXIN	SULFISOOXAZOLE	PROBENECID
Structure	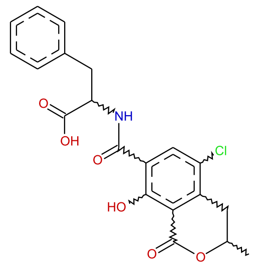	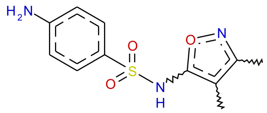	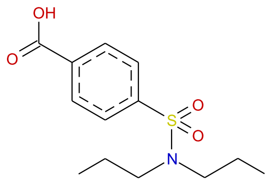
Actual Endpoint (-log C)	6.28396	2.82494	2.85333
Predicted Endpoint (-log C)	5.12358	3.0705	2.4258
Distance	0.852	0.891	1.033
Reference	NCI/NTP TR-358	NCI/NTP TR-138	NCI/NTP TR-395

Model Applicability

Unknown features are fingerprint features in the query molecule, but not found or appearing too infrequently in the training set.

1. Num_AromaticRings out of range. Value: 3. Training min, max, mean, SD: 0, 2, 0.5625, 0.693.
2. OPS PC6 out of range. Value: -2.8672. Training min, max, SD, explained variance: -2.4321, 2.9885, 1.256, 0.0488.
3. OPS PC7 out of range. Value: -3.0478. Training min, max, SD, explained variance: -2.8003, 2.9332, 1.16, 0.0416.
4. Unknown FCFP_2 feature: -885520711: [*]C(=[*])NN=[*]
5. Unknown FCFP_2 feature: 581019816: [*]N\N=C\[*]
6. Unknown FCFP_2 feature: -2100309237: [*]\N=C\C=[*]

Feature Contribution

Top features for positive contribution			
Fingerprint	Bit/Smiles	Feature Structure	Score

FCFP_2	332760439	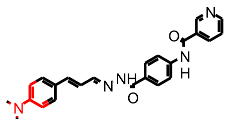<br>[*]O[c](:[cH]:[*]):[c]([*]):[*]	0.672
FCFP_2	1	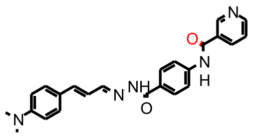<br>[*]=O	0.511
FCFP_2	451847724	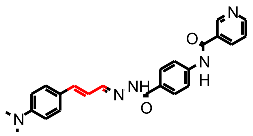<br>[*]\C=C\C=[*]	0.225
**Top Features for negative contribution**			
**Fingerprint**	**Bit/Smiles**	**Feature Structure**	**Score**
FCFP_2	203677720	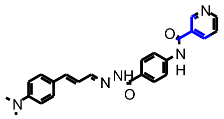<br>[*]C(=[*])[c](:[cH]:[*]):[cH]:[*]	-0.406
FCFP_2	1872154524	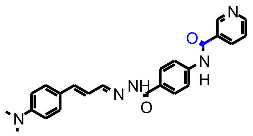<br>[*]C(=O)[*]	-0.307

FCFP_2	0	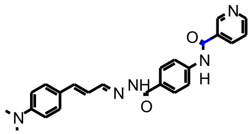 [*]C(=[*])[*]	-0.29

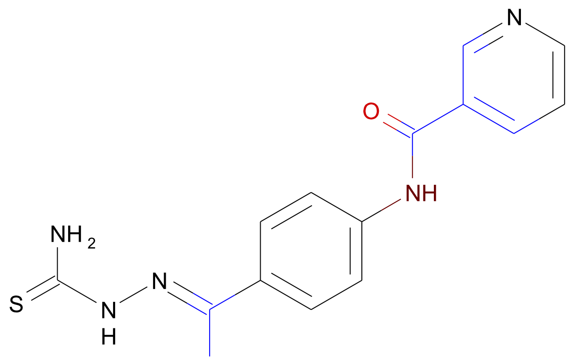

$C_{15}H_{15}N_5OS$
Molecular Weight: 313.3775
ALogP: 1.414
Rotatable Bonds: 5
Acceptors: 4
Donors: 3

Structural Similar Compounds

Name	SULFISOOXAZOLE	HC RED 3	OCHRATOXIN
Structure	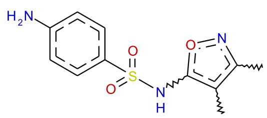	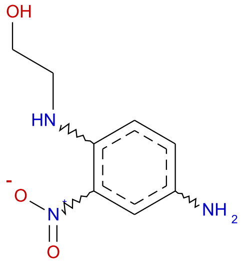	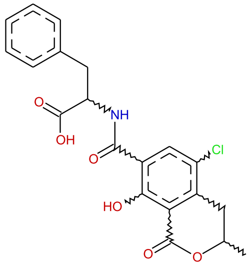
Actual Endpoint (-log C)	2.82494	2.59592	6.28396
Predicted Endpoint (-log C)	3.0705	3.285	5.12358
Distance	0.605	0.689	0.729
Reference	NCI/NTP TR-138	NCI/NTP TR-281	NCI/NTP TR-358

Model Prediction

Prediction: 1.66
Unit: g/kg_body_weight
Mahalanobis Distance: 7.96
Mahalanobis Distance p-value: 0.00229

Mahalanobis Distance: The Mahalanobis distance (MD) is a generalization of the Euclidean distance that accounts for correlations among the X properties. It is calculated as the distance to the center of the training data. The larger the MD, the less trustworthy the prediction.
Mahalanobis Distance p-value: The p-value gives the fraction of training data with an MD greater than or equal to the one for the given sample, assuming normally distributed data. The smaller the p-value, the less trustworthy the prediciton. For highly non-normal X properties (e.g., fingerprints), the MD p-value is wildly inaccurate.

Model Applicability

Unknown features are fingerprint features in the query molecule, but not found or appearing too infrequently in the training set.

1. All properties and OPS components are within expected ranges.
2. Unknown FCFP_2 feature: -1549192822: [*]\N=C(/C)\[c](:[*]):[*]
3. Unknown FCFP_2 feature: 581019816: [*]N\N=C\[*]
4. Unknown FCFP_2 feature: -885520711: [*]C(=[*])NN=[*]
5. Unknown FCFP_2 feature: 1499521844: [*]NC(=S)N

Feature Contribution

Top features for positive contribution			
**Fingerprint**	**Bit/Smiles**	**Feature Structure**	**Score**
FCFP_2	1	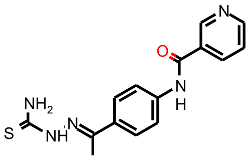 [*]=O	0.511

FCFP_2	3	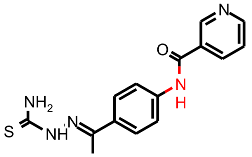 [*]N[*]	0.104

Top Features for negative contribution

Fingerprint	Bit/Smiles	Feature Structure	Score
FCFP_2	136597326	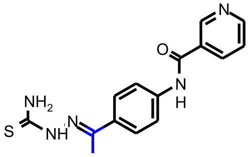 [*]C(=[*])C	-0.489
FCFP_2	203677720	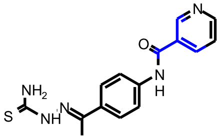 [*]C(=[*])[c](:[cH]:[*]):[cH]:[*]	-0.406
FCFP_2	1872154524	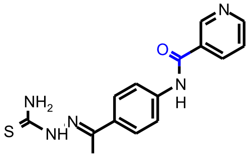 [*]C(=O)[*]	-0.307

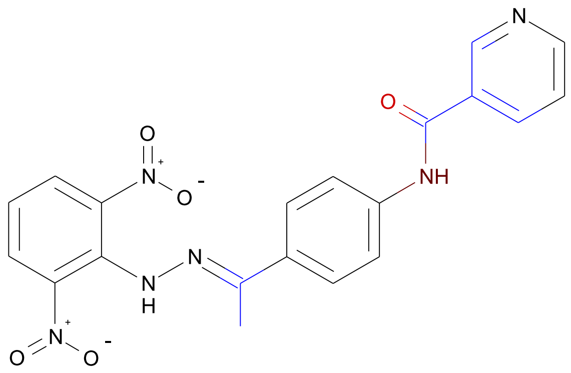

$C_{20}H_{16}N_6O_5$

Molecular Weight: 420.37824

ALogP: 2.852

Rotatable Bonds: 7

Acceptors: 8

Donors: 2

Model Prediction

Prediction: 0.0104

Unit: g/kg_body_weight

Mahalanobis Distance: 10.5

Mahalanobis Distance p-value: 1.1e-006

Mahalanobis Distance: The Mahalanobis distance (MD) is a generalization of the Euclidean distance that accounts for correlations among the X properties. It is calculated as the distance to the center of the training data. The larger the MD, the less trustworthy the prediction.
Mahalanobis Distance p-value: The p-value gives the fraction of training data with an MD greater than or equal to the one for the given sample, assuming normally distributed data. The smaller the p-value, the less trustworthy the prediciton. For highly non-normal X properties (e.g., fingerprints), the MD p-value is wildly inaccurate.

Structural Similar Compounds

Name	OCHRATOXIN	SULFISOOXAZOLE	PENICILLIN VK
Structure	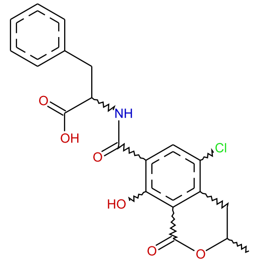	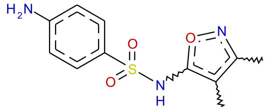	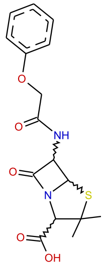
Actual Endpoint (-log C)	6.28396	2.82494	2.54455
Predicted Endpoint (-log C)	5.12358	3.0705	3.9702
Distance	0.898	1.092	1.099
Reference	NCI/NTP TR-358	NCI/NTP TR-138	NCI/NTP TR-336

Model Applicability

Unknown features are fingerprint features in the query molecule, but not found or appearing too infrequently in the training set.

1. Num_H_Acceptors out of range. Value: 8. Training min, max, mean, SD: 0, 6, 1.6146, 1.644.
2. Num_AromaticRings out of range. Value: 3. Training min, max, mean, SD: 0, 2, 0.5625, 0.693.
3. Molecular_PolarSASA out of range. Value: 243.04. Training min, max, mean, SD: 0, 223.97, 50.816, 55.15.
4. Molecular_PolarSurfaceArea out of range. Value: 158.01. Training min, max, mean, SD: 0, 138.03, 28.978, 32.1.
5. Unknown FCFP_2 feature: 8: [*][N+](=[*])[*]
6. Unknown FCFP_2 feature: 5: [*][O-]
7. Unknown FCFP_2 feature: -1549192822: [*]\N=C(/C)\[c](:[*]):[*]
8. Unknown FCFP_2 feature: 581019816: [*]N\N=C\[*]
9. Unknown FCFP_2 feature: 1294285001: [*]=NN[c](:[*]):[*]
10. Unknown FCFP_2 feature: -828984032: [*][c](:[*]):[c]([N+](=[*])[*]):c:[*]
11. Unknown FCFP_2 feature: -1338588315: [*]:[c](:[*])[N+](=O)[O-]
12. Unknown FCFP_2 feature: 1872392852: [*][N+](=O)[*]
13. Unknown FCFP_2 feature: 260476081: [*][N+](=[*])[O-]

Feature Contribution

Top features for positive contribution			
**Fingerprint**	**Bit/Smiles**	**Feature Structure**	**Score**

FCFP_2	1	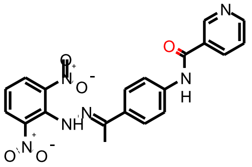 [*]=O	0.511
FCFP_2	3	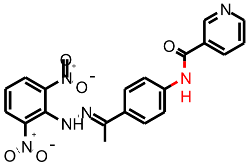 [*]N[*]	0.104
**Top Features for negative contribution**			
**Fingerprint**	**Bit/Smiles**	**Feature Structure**	**Score**
FCFP_2	136597326	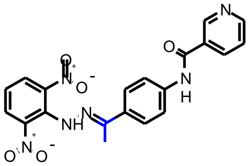 [*]C(=[*])C	-0.489
FCFP_2	203677720	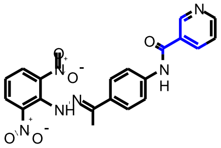 [*]C(=[*])[c](:[cH]:[*]):[cH]:[*]	-0.406
FCFP_2	1872154524	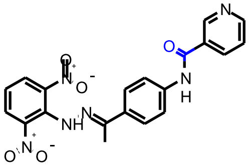 [*]C(=O)[*]	-0.307

Sorafenib

TOPKAT_Rat_Maximum_Tolerated_Dose_Gavage

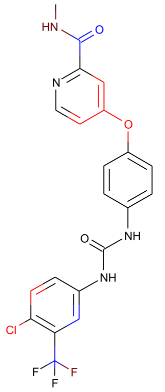

$C_{21}H_{16}ClF_3N_4O_3$

Molecular Weight: 464.82494

ALogP: 4.175

Rotatable Bonds: 6

Acceptors: 4

Donors: 3

Model Prediction

Prediction: 0.000918

Unit: g/kg_body_weight

Mahalanobis Distance: 12.2

Mahalanobis Distance p-value: 4.69e-009

Mahalanobis Distance: The Mahalanobis distance (MD) is a generalization of the Euclidean distance that accounts for correlations among the X properties. It is calculated as the distance to the center of the training data. The larger the MD, the less trustworthy the prediction.

Mahalanobis Distance p-value: The p-value gives the fraction of training data with an MD greater than or equal to the one for the given sample, assuming normally distributed data. The smaller the p-value, the less trustworthy the prediciton. For highly non-normal X properties (e.g., fingerprints), the MD p-value is wildly inaccurate.

Structural Similar Compounds

Name	OCHRATOXIN	SULFISOOXAZOLE	PENICILLIN VK
Structure	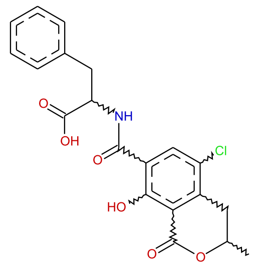	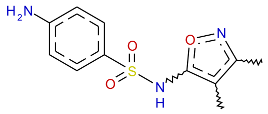	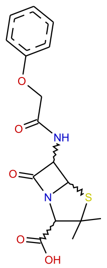
Actual Endpoint (-log C)	6.28396	2.82494	2.54455
Predicted Endpoint (-log C)	5.12358	3.0705	3.9702
Distance	0.758	0.997	1.159
Reference	NCI/NTP TR-358	NCI/NTP TR-138	NCI/NTP TR-336

Model Applicability

Unknown features are fingerprint features in the query molecule, but not found or appearing too infrequently in the training set.

1. Molecular_Weight out of range. Value: 464.82. Training min, max, mean, SD: 68.074, 434.63, 171.13, 85.06.
2. Num_AromaticRings out of range. Value: 3. Training min, max, mean, SD: 0, 2, 0.5625, 0.693.
3. OPS PC5 out of range. Value: -3.5737. Training min, max, SD, explained variance: -3.4, 4.1587, 1.489, 0.0686.
4. OPS PC7 out of range. Value: -3.8342. Training min, max, SD, explained variance: -2.8003, 2.9332, 1.16, 0.0416.
5. Unknown FCFP_2 feature: 136686699: [*]NC
6. Unknown FCFP_2 feature: 1499521844: [*]NC(=S)N
7. Unknown FCFP_2 feature: -1029533685: [*]:[c](:[*])C(F)(F)F

Feature Contribution

Top features for positive contribution

Fingerprint	Bit/Smiles	Feature Structure	Score

FCFP_2	332760439	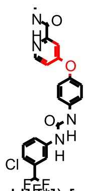 [*]O[c](:[cH]:[*]):[c]([*]):[*]	0.672
FCFP_2	32	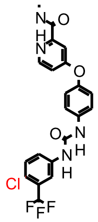 [*]Cl	0.526
FCFP_2	1	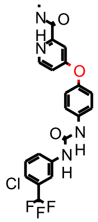 [*]=O	0.511

Top Features for negative contribution

**Fingerprint**	**Bit/Smiles**	**Feature Structure**	**Score**
FCFP_2	203677720	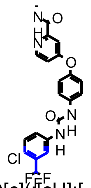 [*]C(=[*])[c](:[cH]:[*]):[cH]:[*]	-0.406
FCFP_2	1872154524	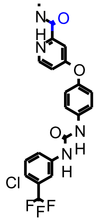 [*]C(=O)[*]	-0.307

FCFP_2	0	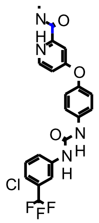 [*]C(=[*])[*]	-0.29

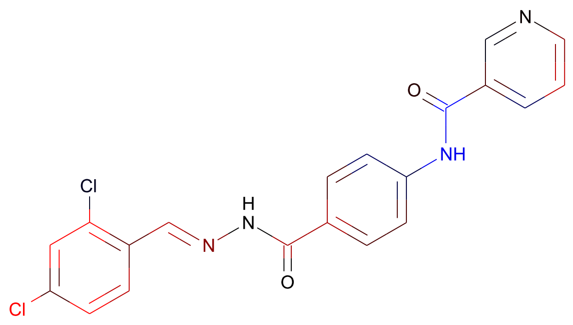

$C_{20}H_{14}Cl_2N_4O_2$

Molecular Weight: 413.25676

ALogP: 3.646

Rotatable Bonds: 5

Acceptors: 4

Donors: 2

Model Prediction

Prediction: 1.49

Unit: g/kg_body_weight

Mahalanobis Distance: 18.5

Mahalanobis Distance p-value: 7.07e-005

Mahalanobis Distance: The Mahalanobis distance (MD) is a generalization of the Euclidean distance that accounts for correlations among the X properties. It is calculated as the distance to the center of the training data. The larger the MD, the less trustworthy the prediction.
Mahalanobis Distance p-value: The p-value gives the fraction of training data with an MD greater than or equal to the one for the given sample, assuming normally distributed data. The smaller the p-value, the less trustworthy the prediciton. For highly non-normal X properties (e.g., fingerprints), the MD p-value is wildly inaccurate.

Structural Similar Compounds

Name	FLUBENDAZOLE	CARBAMIC ACID; N-(5-BENZOYLBENZIMIDAZOL-2-YL)-; METHYL ESTER	INDOMETHAZINE
Structure	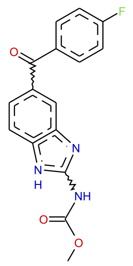	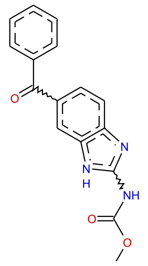	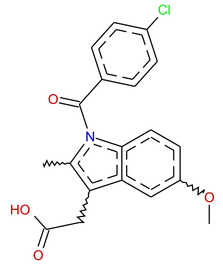
Actual Endpoint (-log C)	2.088	2.617	5.17
Predicted Endpoint (-log C)	2.69288	2.2368	3.33605
Distance	0.567	0.616	0.620
Reference	YRTMA6 9;11;78	IYKEDH 19;735;88	ARZNAD 25;1526;75

Model Applicability

Unknown features are fingerprint features in the query molecule, but not found or appearing too infrequently in the training set.

1. All properties and OPS components are within expected ranges.
2. Unknown FCFP_6 feature: 16: [*]:[cH]:[*]
3. Unknown FCFP_6 feature: 1618154665: [*]:[cH]:[cH]:[cH]:[*]
4. Unknown FCFP_6 feature: 1747237384: [*]:[cH]:n:[cH]:[*]
5. Unknown FCFP_6 feature: -885520711: [*]C(=[*])NN=[*]
6. Unknown FCFP_6 feature: 581019816: [*]N\N=C\[*]
7. Unknown FCFP_6 feature: -2100785893: [*]\N=C\[c](:[*]):[*]
8. Unknown FCFP_6 feature: 71476542: [*]:[c](:[*])Cl

Feature Contribution

Top features for positive contribution			
Fingerprint	Bit/Smiles	Feature Structure	Score

ECFP_6	642810091	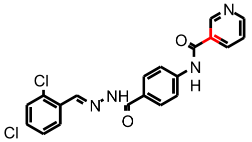 [*][c](:[*]):[*]	0.281
ECFP_6	-1897341097	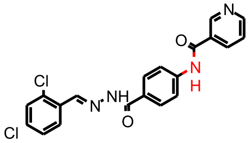 [*]N[*]	0.216
ECFP_6	577592657	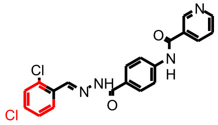 [*][c]1:[*]:[cH]:[cH]:[c](Cl):[cH]:1	0.194
**Top Features for negative contribution**			
**Fingerprint**	**Bit/Smiles**	**Feature Structure**	**Score**
ECFP_6	-817402818	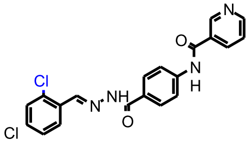 [*]Cl	-0.263
ECFP_6	655739385	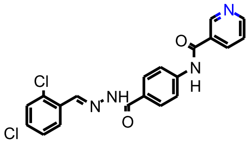 [*]:n:[*]	-0.239

FCFP_6	555188808	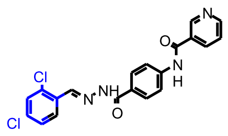<br>[*][c]1:[*]:[cH]:[c](Cl):[cH]:[c]:1Cl	-0.119

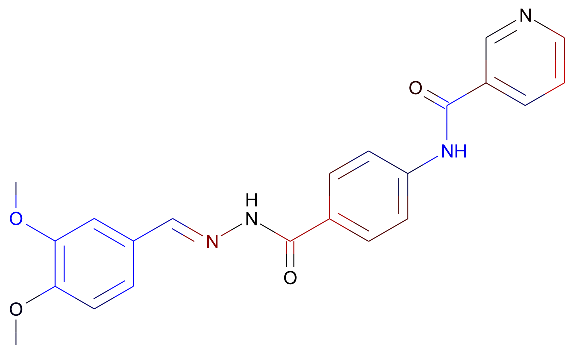

$C_{22}H_{20}N_4O_4$

Molecular Weight: 404.4186

ALogP: 2.284

Rotatable Bonds: 7

Acceptors: 6

Donors: 2

Model Prediction

Prediction: 4.51

Unit: g/kg_body_weight

Mahalanobis Distance: 19.1

Mahalanobis Distance p-value: 3.1e-006

Mahalanobis Distance: The Mahalanobis distance (MD) is a generalization of the Euclidean distance that accounts for correlations among the X properties. It is calculated as the distance to the center of the training data. The larger the MD, the less trustworthy the prediction.
Mahalanobis Distance p-value: The p-value gives the fraction of training data with an MD greater than or equal to the one for the given sample, assuming normally distributed data. The smaller the p-value, the less trustworthy the prediciton. For highly non-normal X properties (e.g., fingerprints), the MD p-value is wildly inaccurate.

Structural Similar Compounds

Name	bis-OXATIN ACETATE	PRASOZIN .HCl (HCl STRIPPED)	ACEMETACIN
Structure	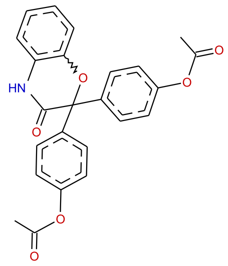	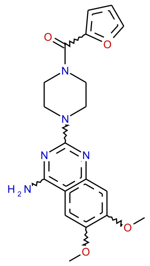	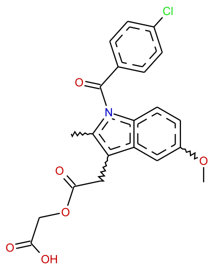
Actual Endpoint (-log C)	1.717	2.294	4.235
Predicted Endpoint (-log C)	2.40947	3.00765	3.39415
Distance	0.582	0.617	0.638
Reference	NIIRDN 6;609;82	NIIRDN 6;688;82	ARZNAD 30;1398;80

Model Applicability

Unknown features are fingerprint features in the query molecule, but not found or appearing too infrequently in the training set.

1. All properties and OPS components are within expected ranges.
2. Unknown FCFP_6 feature: 16: [*]:[cH]:[*]
3. Unknown FCFP_6 feature: 1618154665: [*]:[cH]:[cH]:[cH]:[*]
4. Unknown FCFP_6 feature: 1747237384: [*]:[cH]:n:[cH]:[*]
5. Unknown FCFP_6 feature: -885520711: [*]C(=[*])NN=[*]
6. Unknown FCFP_6 feature: 581019816: [*]N\N=C\[*]
7. Unknown FCFP_6 feature: -2100785893: [*]\N=C\[c](:[*]):[*]

Feature Contribution

Top features for positive contribution			
Fingerprint	Bit/Smiles	Feature Structure	Score

ECFP_6	642810091	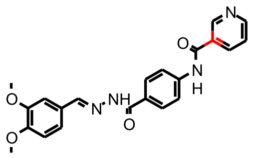<br>[*][c](:[*]):[*]	0.281
ECFP_6	-1897341097	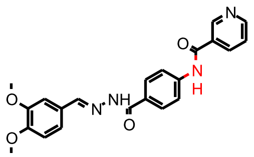<br>[*]N[*]	0.216
FCFP_6	136627117	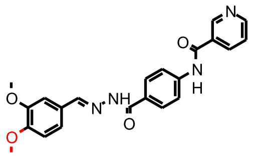<br>[*]OC	0.17

Top Features for negative contribution

Fingerprint	Bit/Smiles	Feature Structure	Score
FCFP_6	1676877079	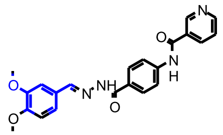<br>[*]O[c]1:[cH]:[c](C=[<br>*]):[cH]:[*]:[c]:1[*<br>]	-0.254
ECFP_6	2077607946	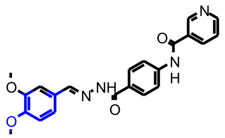<br>[*]O[c]1:[cH]:[cH]:[c<br>]([*]):[*]:[c]:1[*]	-0.252

ECFP_6	655739385	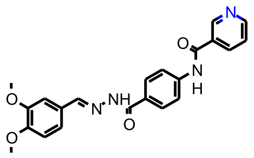<br>[*]:n:[*]	-0.239

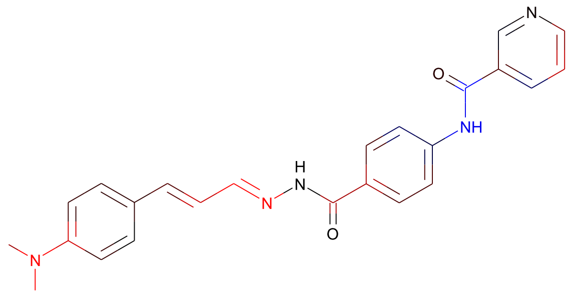

$C_{24}H_{23}N_5O_2$

Molecular Weight: 413.47172

ALogP: 2.839

Rotatable Bonds: 7

Acceptors: 5

Donors: 2

Structural Similar Compounds

Name	bis-OXATIN ACETATE	ACRIDINE; 9-[3-(DIMETHYLAMINO)PROPYLAMINO]-1-NITRO-	FLUBENDAZOLE
Structure	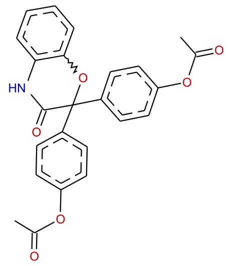	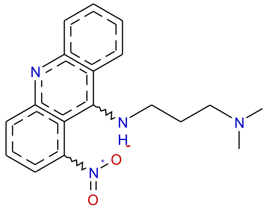	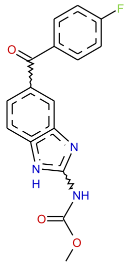
Actual Endpoint (-log C)	1.717	4.101	2.088
Predicted Endpoint (-log C)	2.40947	3.3633	2.69288
Distance	0.616	0.635	0.653
Reference	NIIRDN 6;609;82	MMDPA6 8;252;76	YRTMA6 9;11;78

Model Prediction

Prediction: 1.28

Unit: g/kg_body_weight

Mahalanobis Distance: 22.7

Mahalanobis Distance p-value: 1.46e-019

Mahalanobis Distance: The Mahalanobis distance (MD) is a generalization of the Euclidean distance that accounts for correlations among the X properties. It is calculated as the distance to the center of the training data. The larger the MD, the less trustworthy the prediction.
Mahalanobis Distance p-value: The p-value gives the fraction of training data with an MD greater than or equal to the one for the given sample, assuming normally distributed data. The smaller the p-value, the less trustworthy the prediciton. For highly non-normal X properties (e.g., fingerprints), the MD p-value is wildly inaccurate.

Model Applicability

Unknown features are fingerprint features in the query molecule, but not found or appearing too infrequently in the training set.

1. All properties and OPS components are within expected ranges.
2. Unknown FCFP_6 feature: 16: [*]:[cH]:[*]
3. Unknown FCFP_6 feature: 1618154665: [*]:[cH]:[cH]:[cH]:[*]
4. Unknown FCFP_6 feature: 1747237384: [*]:[cH]:n:[cH]:[*]
5. Unknown FCFP_6 feature: -885520711: [*]C(=[*])NN=[*]
6. Unknown FCFP_6 feature: 581019816: [*]N\N=C\[*]
7. Unknown FCFP_6 feature: 451371068: [*]\C=C\[c](:[*]):[*]

Feature Contribution

Top features for positive contribution			
**Fingerprint**	**Bit/Smiles**	**Feature Structure**	**Score**

ECFP_6	865379614	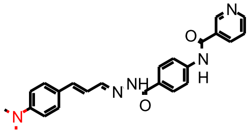 [*]N([*])C	0.322
ECFP_6	642810091	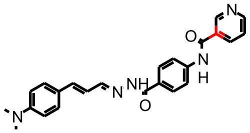 [*][c](:[*]):[*]	0.281
ECFP_6	-1897341097	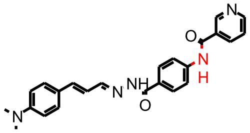 [*]N[*]	0.216
**Top Features for negative contribution**			
**Fingerprint**	**Bit/Smiles**	**Feature Structure**	**Score**
ECFP_6	655739385	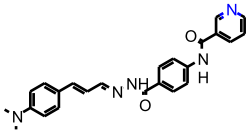 [*]:n:[*]	-0.239
ECFP_6	734603939	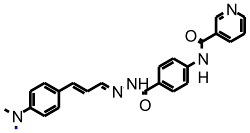 [*]C	-0.201

FCFP_6	-1549103449	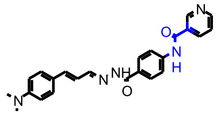<br>[*]NC(=O)[c](:[*]):[*<br>]	-0.117

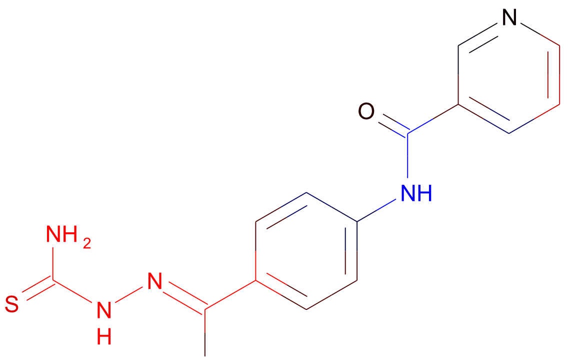

$C_{15}H_{15}N_5OS$
Molecular Weight: 313.3775
ALogP: 1.414
Rotatable Bonds: 5
Acceptors: 4
Donors: 3

Model Prediction

Prediction: 0.477
Unit: g/kg_body_weight
Mahalanobis Distance: 22.3
Mahalanobis Distance p-value: 1.71e-017

Mahalanobis Distance: The Mahalanobis distance (MD) is a generalization of the Euclidean distance that accounts for correlations among the X properties. It is calculated as the distance to the center of the training data. The larger the MD, the less trustworthy the prediction.
Mahalanobis Distance p-value: The p-value gives the fraction of training data with an MD greater than or equal to the one for the given sample, assuming normally distributed data. The smaller the p-value, the less trustworthy the prediciton. For highly non-normal X properties (e.g., fingerprints), the MD p-value is wildly inaccurate.

Structural Similar Compounds

Name	SULFAMETHIZOLE	SULFAFURAZOLE	CARBUTAMIDE
Structure	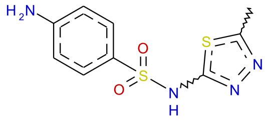	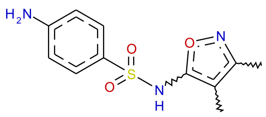	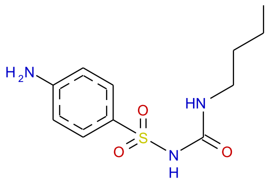
Actual Endpoint (-log C)	1.687	1.427	1.541
Predicted Endpoint (-log C)	1.85826	1.37011	1.48568
Distance	0.628	0.633	0.636
Reference	NIIRDN 6;388;82	NIIRDN 6;391;82	FATOAO 25;93;62

Model Applicability

Unknown features are fingerprint features in the query molecule, but not found or appearing too infrequently in the training set.

1. All properties and OPS components are within expected ranges.
2. Unknown ECFP_2 feature: 128986386: [*]\N=C(/C)\[c](:[*]):[*]
3. Unknown FCFP_6 feature: 16: [*]:[cH]:[*]
4. Unknown FCFP_6 feature: 1618154665: [*]:[cH]:[cH]:[cH]:[*]
5. Unknown FCFP_6 feature: 1747237384: [*]:[cH]:n:[cH]:[*]
6. Unknown FCFP_6 feature: 581019816: [*]N\N=C\[*]
7. Unknown FCFP_6 feature: -885520711: [*]C(=[*])NN=[*]

Feature Contribution

Top features for positive contribution			
**Fingerprint**	**Bit/Smiles**	**Feature Structure**	**Score**

ECFP_6	642810091	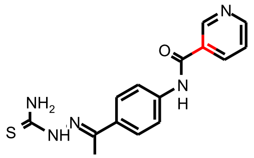 [*][c](:[*]):[*]	0.281
FCFP_6	1499521844	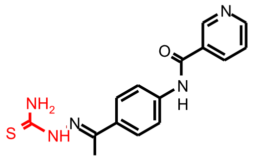 [*]NC(=S)N	0.258
ECFP_6	-1897341097	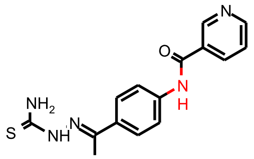 [*]N[*]	0.216

Top Features for negative contribution

Fingerprint	Bit/Smiles	Feature Structure	Score
ECFP_6	655739385	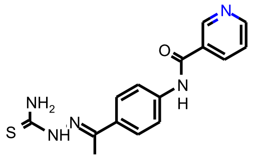 [*]:n:[*]	-0.239
ECFP_6	734603939	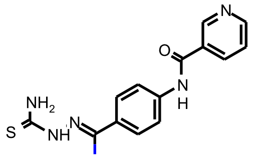 [*]C	-0.201

FCFP_6	-1549103449	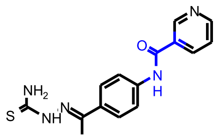 [*]NC(=O)[c](:[*]):[*]]	-0.117

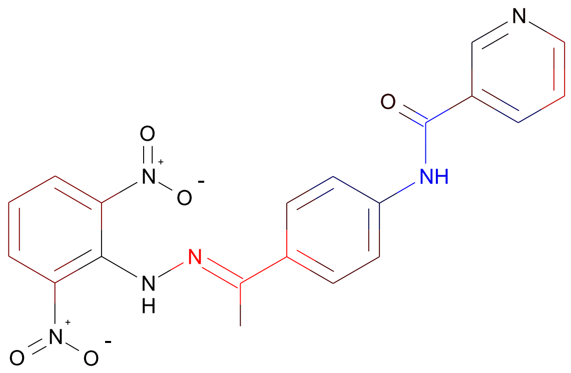

$C_{20}H_{16}N_6O_5$
Molecular Weight: 420.37824
ALogP: 2.852
Rotatable Bonds: 7
Acceptors: 8
Donors: 2

Model Prediction

Prediction: 2.14
Unit: g/kg_body_weight
Mahalanobis Distance: 22.3
Mahalanobis Distance p-value: 1.04e-017

Mahalanobis Distance: The Mahalanobis distance (MD) is a generalization of the Euclidean distance that accounts for correlations among the X properties. It is calculated as the distance to the center of the training data. The larger the MD, the less trustworthy the prediction.
Mahalanobis Distance p-value: The p-value gives the fraction of training data with an MD greater than or equal to the one for the given sample, assuming normally distributed data. The smaller the p-value, the less trustworthy the prediciton. For highly non-normal X properties (e.g., fingerprints), the MD p-value is wildly inaccurate.

Structural Similar Compounds

Name	BENZOTHIAZOLE; 6-NITRO-2-(p-NITROBENZOYLAMINO)-	AZOSEMIDE	FEBANTEL
Structure	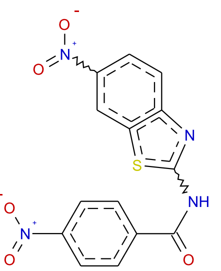	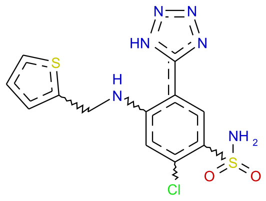	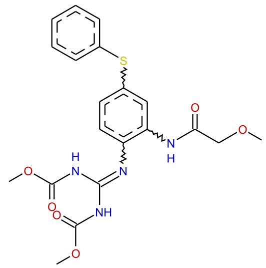
Actual Endpoint (-log C)	2.361	2.163	1.624
Predicted Endpoint (-log C)	2.96257	2.21052	2.37098
Distance	0.679	0.726	0.751
Reference	JPETAB 90;260;47	IYKEDH 18;666;87	ARZNAD 28;2193;78

Model Applicability

Unknown features are fingerprint features in the query molecule, but not found or appearing too infrequently in the training set.

1. All properties and OPS components are within expected ranges.
2. Unknown ECFP_2 feature: 1043790491: [*][N+](=[*])[*]
3. Unknown ECFP_2 feature: 781519895: [*][O-]
4. Unknown ECFP_2 feature: 128986386: [*]\N=C(/C)\[c](:[*]):[*]
5. Unknown ECFP_2 feature: -1956535100: [*][c](:[*]):[c]([N+](=[*])[*]):c:[*]
6. Unknown ECFP_2 feature: -215026467: [*]:[c](:[*])[N+](=O)[O-]
7. Unknown ECFP_2 feature: 2104376220: [*][N+](=O)[*]
8. Unknown ECFP_2 feature: -659271057: [*][N+](=[*])[O-]
9. Unknown FCFP_6 feature: 16: [*]:[cH]:[*]
10. Unknown FCFP_6 feature: 8: [*][N+](=[*])[*]
11. Unknown FCFP_6 feature: 5: [*][O-]
12. Unknown FCFP_6 feature: 1618154665: [*]:[cH]:[cH]:[cH]:[*]
13. Unknown FCFP_6 feature: 1747237384: [*]:[cH]:n:[cH]:[*]
14. Unknown FCFP_6 feature: 581019816: [*]N\N=C\[*]
15. Unknown FCFP_6 feature: 1294285001: [*]=NN[c](:[*]):[*]
16. Unknown FCFP_6 feature: -828984032: [*][c](:[*]):[c](:[cH]:[*])[N+](=[*])[*]
17. Unknown FCFP_6 feature: -1338588315: [*]:[c](:[*])[N+](=O)[O-]
18. Unknown FCFP_6 feature: 1872392852: [*][N+](=O)[*]
19. Unknown FCFP_6 feature: 260476081: [*][N+](=[*])[O-]

Feature Contribution

Top features for positive contribution			
**Fingerprint**	**Bit/Smiles**	**Feature Structure**	**Score**
ECFP_6	642810091	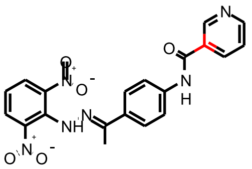 [*][c](:[*]):[*]	0.281
ECFP_6	-1897341097	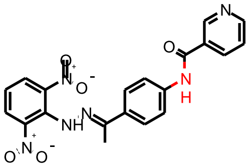 [*]N[*]	0.216
FCFP_6	-1549192822	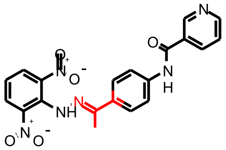 [*]\N=C(/C)\[c](:[*]) :[*]	0.168

Top Features for negative contribution			
**Fingerprint**	**Bit/Smiles**	**Feature Structure**	**Score**
ECFP_6	655739385	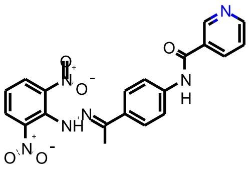 [*]:n:[*]	-0.239

ECFP_6	734603939	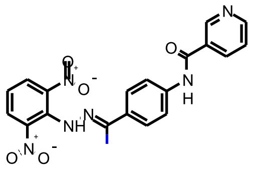 [*]C	-0.201
FCFP_6	-1549103449	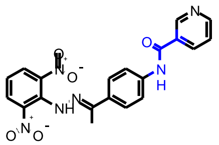 [*]NC(=O)[c](:[*]):[*]	-0.117

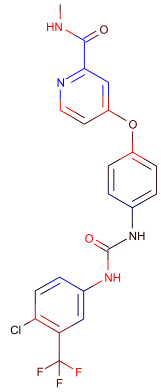

$C_{21}H_{16}ClF_3N_4O_3$
Molecular Weight: 464.82494
ALogP: 4.175
Rotatable Bonds: 6
Acceptors: 4
Donors: 3

Structural Similar Compounds

Name	FLUBENDAZOLE	PHOSPHORAMIDOTHIOIC ACID; ACETIMIDOYL-; O;O-bis-(p-CHLOROPHENYL)ESTER	BEZAFIBRATE
Structure	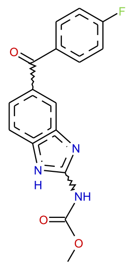	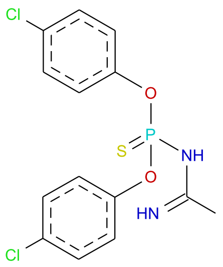	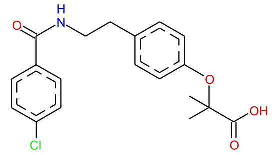
Actual Endpoint (-log C)	2.088	5.006	1.946
Predicted Endpoint (-log C)	2.69288	3.23989	2.54395
Distance	0.697	0.703	0.721
Reference	YRTMA6 9;11;78	FMCHA2 -;C149;89	ARZNAD 30;2023;80

Model Prediction

Prediction: 0.823
Unit: g/kg_body_weight
Mahalanobis Distance: 21
Mahalanobis Distance p-value: 1.93e-012

Mahalanobis Distance: The Mahalanobis distance (MD) is a generalization of the Euclidean distance that accounts for correlations among the X properties. It is calculated as the distance to the center of the training data. The larger the MD, the less trustworthy the prediction.
Mahalanobis Distance p-value: The p-value gives the fraction of training data with an MD greater than or equal to the one for the given sample, assuming normally distributed data. The smaller the p-value, the less trustworthy the prediciton. For highly non-normal X properties (e.g., fingerprints), the MD p-value is wildly inaccurate.

Model Applicability

Unknown features are fingerprint features in the query molecule, but not found or appearing too infrequently in the training set.

1. All properties and OPS components are within expected ranges.
2. Unknown FCFP_6 feature: 16: [*]:[cH]:[*]
3. Unknown FCFP_6 feature: 1618154665: [*]:[cH]:[cH]:[cH]:[*]
4. Unknown FCFP_6 feature: 1747237384: [*]:[cH]:n:[cH]:[*]
5. Unknown FCFP_6 feature: 136686699: [*]NC
6. Unknown FCFP_6 feature: 71476542: [*]:[c](:[*])Cl

Feature Contribution

Top features for positive contribution

Fingerprint	Bit/Smiles	Feature Structure	Score

FCFP_6	71953198	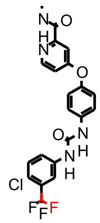 [*]C([*])([*])F	0.392
ECFP_6	-1046436026	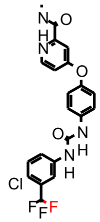 [*]F	0.349
ECFP_6	642810091	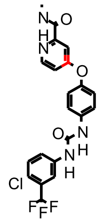 [*][c](:[*]):[*]	0.281

Top Features for negative contribution

Fingerprint	Bit/Smiles	Feature Structure	Score
ECFP_6	226796801	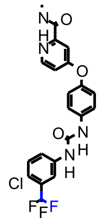 [*]C([*])([*])F	-0.32
ECFP_6	-817402818	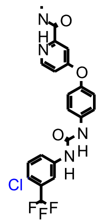 [*]Cl	-0.263

ECFP_6	-176455838	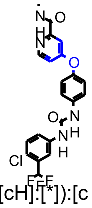 [*]O[c](:[cH]:[*]):[cH]:[*]	-0.257
